# Chemical Probes for the Functionalization of Polyketide Intermediates**

**DOI:** 10.1002/anie.201407448

**Published:** 2014-09-11

**Authors:** Elena Riva, Ina Wilkening, Silvia Gazzola, W M Ariel Li, Luke Smith, Peter F Leadlay, Manuela Tosin

**Affiliations:** Department of Chemistry, University of WarwickLibrary Road, Coventry CV4 7AL (UK); Department of Chemistry, University of CambridgeLensfield Road, Cambridge CB2 1EW (UK); Department of Biochemistry, University of Cambridge80 Tennis Court Road, Cambridge CB2 1GA (UK)

**Keywords:** biosynthesis, enzymes, drug discovery, polyketides, synthetic methods

## Abstract

A library of functionalized chemical probes capable of reacting with ketosynthase-bound biosynthetic intermediates was prepared and utilized to explore in vivo polyketide diversification. Fermentation of ACP mutants of *S. lasaliensis* in the presence of the probes generated a range of unnatural polyketide derivatives, including novel putative lasalocid A derivatives characterized by variable aryl ketone moieties and linear polyketide chains (bearing alkyne/azide handles and fluorine) flanking the polyether scaffold. By providing direct information on microorganism tolerance and enzyme processing of unnatural malonyl-ACP analogues, as well as on the amenability of unnatural polyketides to further structural modifications, the chemical probes constitute invaluable tools for the development of novel mutasynthesis and synthetic biology.

As direct outcomes of millions of years of structural and functional evolution, natural products remain at the forefront of drug discovery and development.^1^ Polyketides in particular constitute a highly diverse family including important pharmaceuticals and agrochemicals such as the antibiotic erythromycin A, the cholesterol-lowering agent lovastatin, the antiparasitic ivermectin, and the immunosuppressant rapamycin.^2^ Polyketides are biosynthesized in microorganisms and plants by the polyketide synthase (PKS) multienzymes. PKSs utilize a wide range of bioavailable acyl building blocks (e.g. acetate, propionate, butyrate, etc.) and iterative decarboxylative Claisen condensation to generate stereoenriched enzyme-bound polyketide chains, which are eventually released from PKSs and can be further enzymatically tailored to yield the final bioactive products.^2^ Natural product structural diversification is highly desirable to obtain novel molecules of increased efficiency and/or novel bioactivity.^3^ Synthetic biology is currently seen as a highly promising avenue to generate novel polyketide derivatives, especially in the context of modular PKSs.^4^ This potential arises because of the relative amenability of these assembly lines to genetic manipulation by domain swap and other redesign.^5^–^7^ Current approaches towards the generation of unnatural polyketides (compounds which are still made by enzymatic assembly but are structurally different from the original products) include semisynthesis, precursor-directed biosynthesis, mutasynthesis, combinatorial biosynthesis, and chemogenetics.^8^ Chemical synthesis as well as enzyme and metabolic engineering can be utilized to provide polyketide machineries with unnatural building blocks. Recent efforts in these directions have highlighted both the innate and engineered chemical flexibility of PKSs in accepting a variety of non-native substrates to generate novel polyketide derivatives in vitro and in vivo, thus opening up the exploitation of novel chemical spaces and bioactivities for this class of natural products.^9^ We have recently developed synthetic chain terminators capable of capturing polyketide biosynthetic intermediates in vitro^10^ and in vivo.^11^ These chemical probes (e.g., the β-ketoacids **1 a**,**b**; Scheme 1; generated in situ from the hydrolysis of the corresponding methyl esters)^11^ compete with ACP-bound malonate units for polyketide chain extension. By reacting with enzyme-bound biosynthetic intermediates, intermediate-like species (**2 a**,**b**) are off-loaded from PKSs and become available for LC-MS characterization. Further, the use of these molecules in vivo has allowed us to reveal relatively inaccessible features of polyketide assembly, such as the timing of ring formation in the biosynthesis of the polyether antibiotic lasalocid A.11b

**Scheme 1:**
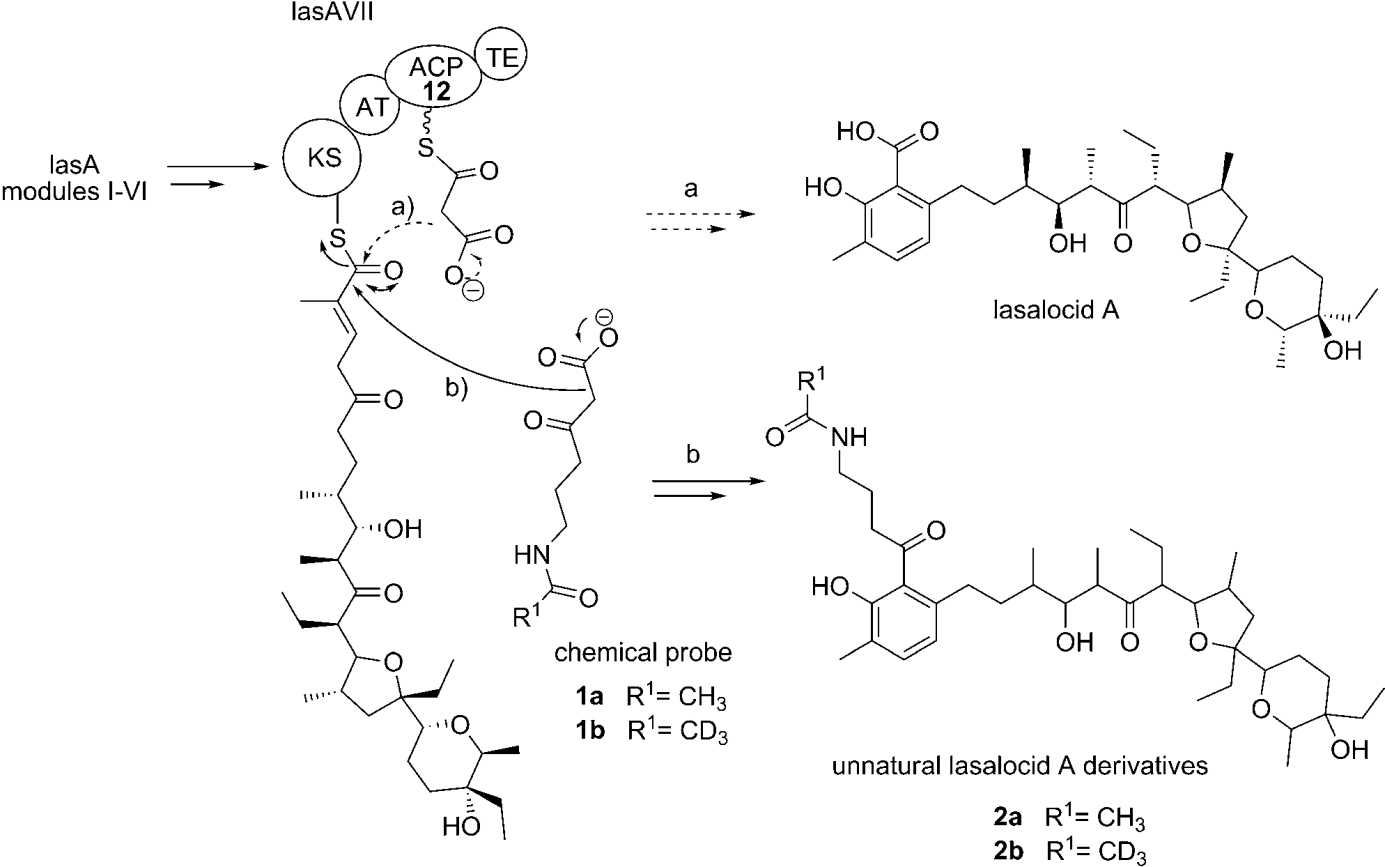
a) Late stages in the biosynthesis of lasalocid A by the *lasA* PKS: malonyl-ACP decarboxylative condensation with the last KS-bound intermediate, followed by aromatization and thioester hydrolysis, leads to natural product formation. b) The competitive decarboxylative condensation of the carba(dethia) *N*-acetyl cysteamine probes 1 a,b11b with the same advanced intermediate leads to the formation of the lasalocid A unnatural derivatives 2 a,b. ACP=acyl carrier protein, AT=acyl transferase, KS=ketosynthase, TE=thioesterase.

In characterizing the captured biosynthetic intermediate species, it occurred to us that it might be possible, by utilizing further derivatized chemical probes together with suitably engineered bacterial strains, to rapidly generate a variety of novel polyketide intermediates and products bearing bioorthogonal chemical handles for site-specific modifications, such as alkyne and azide moieties,^12^ as well as pharmaceutically relevant motifs, such as fluorine.^13^ To test our hypothesis, we prepared a small library of second-generation methyl ester probes characterized by the variation of the N-acyl moiety and 1,3-dicarbonyl functionalization (**3**–**13**; Scheme 2). We then employed these molecules as substrates in small-scale fermentations (10 mL) of ACP mutant strains of *Streptomyces lasaliensis.* The strains, constructed from *S. lasaliensis* NRRL 3382 by point mutation of the active serine (for 4′-phosphopantetheine attachment) of ACP12 and ACP5 to alanine residues as previously reported,11b do not produce the natural polyketide product lasalocid A. However they still harbour enzyme-bound biosynthetic intermediates.11b The esters **3**–**13** were hydrolyzed in situ to the corresponding β-ketoacids **1** and **14**–**23** by endogenous esterases, with the extent of the hydrolysis being substrate-dependent (see the Supporting Information).

**Scheme 2:**
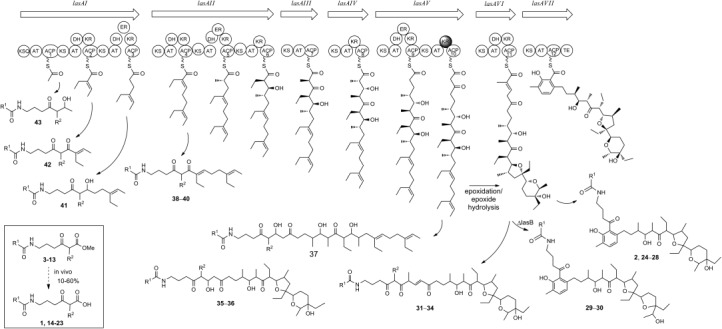
Generation of putative functionalized polyketide intermediates and the products 2 and 24–43 by fermentation of *S. lasaliensis* in the presence of 1 and 14–23 (derived from in vivo hydrolysis of the corresponding methyl esters 3–13; see boxed inset). The stereochemistry of the newly formed compounds is yet to be established.

The overall outcome of the feeding experiments is summarized in Scheme 2 and Table 1 (see the Supporting Information for details). In the ethyl acetate extracts of the ACP12 (S970A) mutant, grown in the presence of the methyl ester substrates **3**–**8** (supplemented daily to reach a 2.5–4.0 mm final concentration), the putative lasalocid A derivatives **2** and **24**–**28** were identified as the major polyketide products off-loaded from the *lasA* PKS by micro-LC-HR-MS analyses (Table 1, entries 1–7). The compounds **2** and **24**–**28** were characterized by HR-MS^2^ and displayed a conserved fragmentation pattern involving McLafferty rearrangement and subsequent formation of *m*/*z* 377, a diagnostic polyether fragment.11b In addition, for the compounds **27** and **28** the loss of the C10 acyl chain occurring during fragmentation afforded an *m*/*z* 260 fragment, which would correspond to a protonated cyclic imine (Figure 1 c and see the Supporting Information) and strongly supports the aromatic ketone nature of these derivatives. The formation of **24**–**28** indicates that variations of the N-acyl moiety of the pseudomalonate substrates are well tolerated by both *S. lasaliensis* and the *lasA* PKS in the range of concentrations utilized in these experiments. Interestingly, additional formation of short-chain products (e.g., the pentaketides **38**–**40**) was observed for long-chain substrates (**7**–**8**, and, later on, **13**; Table 1, entries 6, 7, and 19). The nature of these products was independently verified by feeding of *S. lasaliensis* ACP5 (S3799A) with **7** and **8** (entries 9 and 10). Putative iso-lasalocid derivatives (**29**–**30**) were also selectively obtained when the compounds **6** and **8** were fed to *S. lasaliensis* ΔlasB-ACP12 (S970A)11b (entries 11 and 12). These derivatives displayed a similar HR-MS^2^ fragmentation pattern to their lasalocid putative counterparts but eluted at significantly different retention times, as previously observed11b (see the Supporting Information).

**Figure 1:**
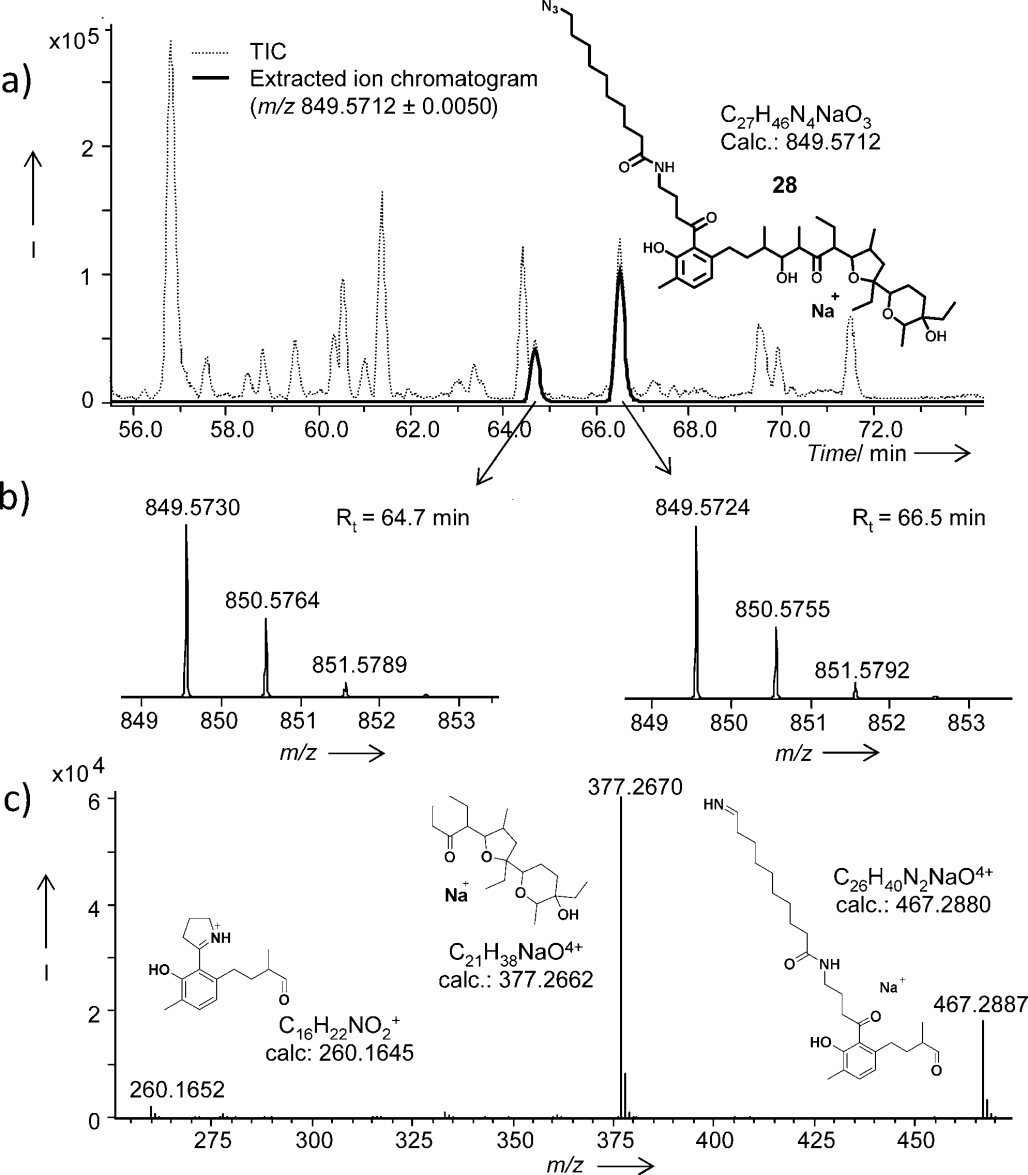
UPLC*-*HR-MS analyses of the ethyl acetate extracts of *S. lasaliensis* ACP12 (S970A) grown in the presence of substrate 8 (Table 1, entry 7). a) TIC and extracted ion chromatogram traces showing the presence of the compound 28 (Acquity HSS T3 column, 78-minute gradient). b) HR-MS and c) HR-MS^2^ of the putative lasalocid derivative 28. The double LC-MS peak for 28 and all the other putative lasalocid derivatives is under investigation.

**Table 1:** Generation of putative unnatural polyketides from the *lasA* PKS and the substrates 1 and 14–23 (generated from in vivo hydrolysis of 3–13; Scheme 2).

Entry	*S. lasaliensis* strain	Substrate[a]			Major product(s)[b]	
		Ester	acid	R^1^	R^2^	
1	ACP12 (S970A)	**3 a**	**1 a**	CH_3_	H	**2 a**[c]
2	ACP12 (S970A)	**3 b**	**1 b**	CD_3_	H	**2 b**[c]
3	ACP12 (S970A)	**4**	**14**	CF_3_	H	**24**[c]
4	ACP12 (S970A)	**5**	**15**	(CH_2_)_2_C=CH	H	**25**[c]
5	ACP12 (S970A)	**6**	**16**	(CH_2_)_3_N_3_	H	**26**[c]
6	ACP12 (S970A)	**7**	**17**	(CH_2_)_8_CH_3_	H	**27**,[c] **38**^[c,e]^
7	ACP12 (S970A)	**8**	**18**	(CH_2_)_9_N_3_	H	**28**,[c] **39**[c]
8	ACP5 (S3799A)	**5**	**15**	(CH_2_)_2_C=CH	H	not detected
9	ACP5 (S3799A)	**7**	**17**	(CH_2_)_8_CH_3_	H	**38**[c]
10	ACP5 (S3799A)	**8**	**18**	(CH_2_)_9_N_3_	H	**39**[c]
11	ΔlasB-ACP12 (S970A)	**6**	**16**	(CH_2_)_3_N_3_	H	**29**[c]
12	ΔlasB-ACP12 (S970A)	**8**	**18**	(CH_2_)_9_N_3_	H	**30**[c]
13	ACP12 (S970A)	**9**	**19**	(CH_2_)_2_C=CH	CH_3_	**31**[c]
14	WT (NRRL 3382)	**9**	**19**	(CH_2_)_2_C=CH	CH_3_	**35, 31**[d]
15	WT (NRRL 3382)	**10**	**20**	CH_3_	CH_3_	**36, 32,**[d]
16	ACP12 (S970A)	**11 a**	**21 a**	CH_3_	F	**37 a, 33 a**[d]
17	ACP12 (S970A)	**11 b**	**21 b**	CD_3_	F	**37 b, 33 b**[d]
18	ACP12 (S970A)	**12**	**22**	(CH_2_)_2_C=CH	F	not detected
19	ACP12 (S970A)	**13**	**23**	(CH_2_)_8_CH_3_	F	**40**–**43, 34**[d]

[a] Methyl ester hydrolyzed in vivo to the corresponding acid (see boxed inset in Scheme 2).

[b] Major product(s) as deduced by LC-HRMS analysis.

[c] Double peaks (for both sodium and ammonium adducts) detected, possibly arising from isomers/conformers.^16^

[d] Detected in minor amounts. [e] Products of different chain lengths and oxidations states detected in minor amounts.

1,3-dicarbonyl functionalization of the chemical probes led to further unexpected results. From the fermentation of *S. lasaliensis* ACP12 (S970A) in the presence of the *N*-pentynoyl 2*-*methyl hexanoate substrate **9**, the novel putative polyether derivative **31** was obtained and characterized by HR-MS^2^ (Table 1, entry 13). This product indicates that the last ketosynthase (KS) domain of the *lasA* PKS is capable of carrying out chain extension with a methyl malonate mimic in place of the natural malonate, followed by ketoreduction and dehydration. As a result, the normal chain aromatization does not take place and a chemically derivatized linear polyether is generated instead. Oxidized undecaketides such as **35** and **36** were initially identified as the major species generated from the *lasA* PKS in wild-type *S. lasaliensis* grown in the presence of the methylmalonate mimics **9** and **10** (entries 14 and 15). Conversely, the use of an engineered strain in conjunction with a newly optimized feeding protocol for liquid cultures has herein favored the unprecedented formation of linear oxidized dodecaketides such as **31**. Intriguingly, when the *N*-acetyl- and deuteroacetyl-2*-*fluoro hexanoates **11 a** and **11 b**, respectively, were employed as substrates for *S. lasaliensis* ACP12 (S970A), the corresponding fluorinated undecaketides **37 a** and **37 b** were identified as the main products off-loaded from the PKS (entries 16 and 17), with the oxidized linear dodecaketides **33 a**,**b** barely detectable. No obvious products were identified from the feeding of *S. lasaliensis* ACP12 (S970A) with the *N*-pentynoyl 2*-*fluoro substrate **12** (entry 18), whereas from the *N*-decanoyl 2*-*fluoro substrate **13**, the fluorinated putative polyether **34** was afforded, albeit in minor amount compared to the short-chain products **40**–**43** (entry 19; all products characterized by HR-MS^n^, see the Supporting Information).

These results show for the first time that, by directly utilizing fluoromalonate surrogate substrates in vivo, fluorine can be successfully incorporated into advanced polyketide biosynthetic intermediates and products at specific α-carbonyl positions in place of hydrogen atoms, and methyl and ethyl groups with unexpected consequences on the natural product assembly. Indeed for the *lasA* PKS the stage and the extent of fluorine incorporation is dependent on the N-acyl length of the probe (Table 1). Additionally, the absence of oxidized undecaketide species bearing fluorine seems to suggest that 2-fluorinated undecaketide dienes may not be suitable substrates for epoxidation/epoxide hydrolysis leading to polyether formation. These findings are of particular relevance in view of engineering the production of fluorinated polyketides9d, 13d for medicinal chemistry and chemical biology applications.

Most of the polyketide products arising from these experiments also bear an alkyne or an azide moiety. To show how these molecules can be creatively diversified, the putative lasalocid A derivatives **25**, **26**, and **28** were utilized as substrates for azide–alkyne Huisgen cycloaddition^14^ and Staudinger-phosphite^15^ reactions in crude and purified organic extracts of *S. lasaliensis* cultures (Figure 2). In particular, the crude putative lasalocid alkyne derivative **25** was reacted with the N-Boc-cadaverine azide **44** in the presence of copper(I) to afford the putative lasalocid conjugate **45**. The same transformation was carried out on HPLC-purified fractions of **25**, thus leading to identical click products (Figure 2 a). The putative azide polyethers **26** and **28** were converted into glucose conjugates (e.g., **47**) by click reactions with the alkyne **46**, and to phosphoramidates (e.g., **48**) by reaction with trimethyl phosphite (Figures 2 b,c).

**Figure 2:**
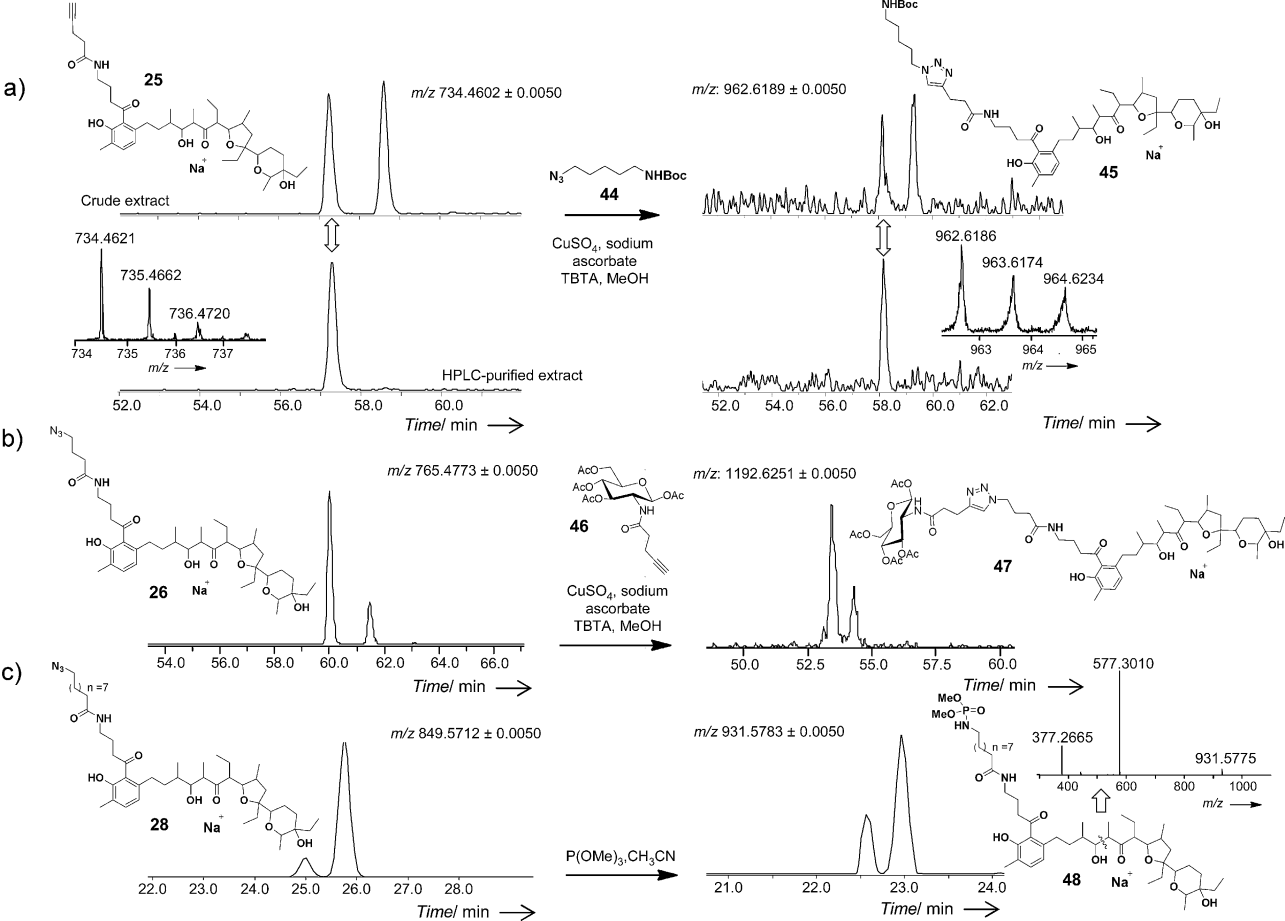
a) Conversion of the putative lasalocid alkyne derivative 25 into the cadaverine conjugate 45 by copper-catalyzed azide–alkyne cycloaddition (CuAAC) with the azide 44 (carried out in crude and purified organic extracts, as shown in upper and lower selected LC-MS ion traces, respectively (Acquity HSS T3 column, 68-minute gradient). b) The putative lasalocid azide derivative 26 is converted into the glucose conjugate 47 by CuAAC with the alkyne 46 (Acquity HSS T3 column, 68-minute gradient). c) The putative lasalocid azide derivative 28 is converted into the phosphoramidate 48 by a Staudinger-phosphite reaction (Eclipse C18 column, 35-minute gradient). The presence of a double LC-MS peak for all the newly generated putative lasalocid derivatives is under investigation.

Overall the compounds **2**, **24**–**28**, **31**–**36** and their derivatives were identified and characterized as putative lasalocid A species by HR-MS^2^, all displaying the conserved *m*/*z* 377 fragment previously mentioned11b and a consistent LC-MS double-peak pattern. This pattern is possibly due to isomerization or the adoption of different monomeric/dimeric structures of the polyethers in complex with sodium.^16^ Because of their formation in limited amounts and the complexity of the samples background, it has not always been straightforward to detect the presence of the unnatural polyketides in the bacterial crude extracts (Figure 1). From comparative LC-MS analyses with samples of lasalocid A of known titre, we have preliminarily estimated the production of the unnatural polyethers to be in the range of 100–800 μg l^−1^, depending on the probe utilized and in the conditions here reported (data not shown). Nonetheless, we have shown here that such diverse and functionalized products can be straightforwardly generated from the fermentation of a minimally engineered *Streptomyces* strain in the presence of varied synthetic malonate surrogates (**3**–**13**), they can be isolated by HPLC purification and they can be further derivatized (Figure 2).

To the best of our knowledge, **2**, **24**–**28**, **31**–**36**, **45**, **47**, and **48** together constitute the first example of an unnatural polyether library characterized by the replacement of the aryl carboxylate functionality with variable aryl ketones (**2, 24**–**28**, **45**, **47**, and **48**) and with linear polyketide chains flanking the polyether portion of the natural product (**31**–**36**). Polyether ionophores are widely utilized as antibiotics in veterinary medicine, and they are also rapidly emerging as promising bioactive molecules for cancer therapy^17^ and for the treatment of tropical diseases.^18^ Structural modification of polyethers can change the ability and the selectivity of metal cations and small-molecule binding.^19^ Therefore this approach may give access to novel molecules of therapeutic interest, as well as helping illuminate their mechanism of action.

On the basis of the results reported here, it is also appealing to envisage the generation of a library of polyketide derivatives by a novel mutasynthetic approach involving chain re-initiation of stalled biosynthetic intermediates on any ketosynthase with a variety of malonyl-ACP mimics. N-acetyl cysteamine analogues of malonate extender units and of advanced biosynthetic intermediates are widely utilized in current mutasynthetic approaches in place of the natural substrates bound to the pantetheinyl cofactor of acyl carrier proteins.^8^, ^9^, 20b It is now clear that the pantetheinyl portion of acyl carrier proteins can be replaced by a variety of N-acyl groups of variable chain length and nature to generate functionalized polyketides. This feature is of particular interest in relation to the newly available three-dimensional structure of a whole PKS module,^20^ as molecular modeling should be now employed to design unnatural malonyl-ACP analogues capable of best interacting with all the catalytic partners in the PKS reaction chamber. In light of all these considerations, we are currently exploring whether the mutasynthetic approach to the putative lasalocid precursor/derivative structures reported herein is an effective and general approach applicable to other PKSs and modular assembly lines for the generation of novel unnatural products.

In summary, the functionalized chemical probes **3**–**13** constitute first-hand chemical biology tools for exploring novel polyketide diversification by providing direct information on PKSs tolerance and processing of unnatural malonyl-ACP analogues, as well as on the amenability of the newly generated unnatural polyketides to further synthetic modifications.

Our current work is focusing on the large-scale isolation of the novel polyketide derivatives reported here, their full characterization by NMR spectroscopy, and the evaluation of their bioactivity, as well as on the development of tools to enhance their production in vivo. The application of the probes **3**–**13** for the investigation and exploitation of other PKS systems will be reported in due course.

## Notes

Supporting information for this article (synthesis of probes 3–13 and their use in the fermentation of S. lasaliensis strains, as well as detailed LC-HR-MS^n^ analysis of all the newly generated polyketide intermediates and products (24–43, 45, 47–48)) is available on the WWW under http://dx.doi.org/10.1002/anie.201407448.
