# Supplementary material for: Chemical Probes for the Functionalization of Polyketide Intermediates
Source: Angew Chem Int Ed Engl. 2014 Sep 11;53(44):11944–9. doi: 10.1002/anie.201407448 (PMC4501312; doi:10.1002/anie.201407448)

Angewandte Chemie
Eine Zeitschrift der Gesellschaft Deutscher Chemiker

Supporting Information

© Wiley-VCH 2014

69451 Weinheim, Germany

Chemical Probes for the Functionalization of Polyketide Intermediates**

*Elena Riva, Ina Wilkening, Silvia Gazzola, W. M. Ariel Li, Luke Smith, Peter F. Leadlay, and Manuela Tosin**

anie_201407448_sm_miscellaneous_information.pdf

TABLE OF CONTENTS

1 GENERAL METHODS 3

2 SYNTHESIS OF CHEMICAL PROBES AND COMPOUND CHARACTERIZATION 4

2.1 SYNTHESIS OF PROBE 4 4
2.2 SYNTHESIS OF PROBES 5, 9 AND 12 6
2.3 SYNTHESIS OF PROBE 6 10
2.4 SYNTHESIS OF PROBES 7 AND 13 13
2.5 SYNTHESIS OF PROBE 8 16
2.6 SYNTHESIS OF PROBES 11A AND 11B 19
2.7 SYNTHESIS OF *TERT*-BUTYL 5-AZIDOPENTYLCARBAMATE (44) 20
2.8 SYNTHESIS OF 1,3,4,6-TETRA-*O*-ACETYL-*N*-4-PENTYNOYLGLUCOSAMINE (46) 21

3 FEEDING EXPERIMENTS 23

3.1 MICROBIOLOGY METHODS 23
3.2 CONSTRUCTION OF MUTANT STRAINS OF *STREPTOMYCES LASALIENSIS* 23
3.3 GROWTH OF *S. LASALIENSIS* WILD-TYPE (WT), ACP12 (S970A), ACP5 (S3799A) AND ΔLASB-ACP12 (S970A) STRAINS AND MASS SPECTROMETRY ANALYSIS 24
3.4 OFF-LOADING OF FUNCTIONALIZED POLYKETIDES FROM *S. LASALIENSIS* ACP12 (S970A) AND ΔLASB-ACP12 (S970A) VIA CHEMICAL PROBES 4-8 26
3.5 LC-HRMS AND MS/MS ANALYSIS OF OFF-LOADED POLYKETIDES GENERATED FROM *S. LASALIENSIS* ACP5 (S3799A) 46
3.6 OFF-LOADING OF FUNCTIONALIZED POLYKETIDES FROM *S. LASALIENSIS* WT AND ACP12 (S970A) VIA CHEMICAL PROBES 9 48
3.7 OFF-LOADING OF FUNCTIONALIZED POLYKETIDES FROM *S. LASALIENSIS* ACP12 (S970A) VIA CHEMICAL PROBES 11A, 11B AND 13 51

4 COPPER-CATALYZED AZIDE-ALKYNE CYCLOADDITION (CUAAC) OF ALKYNE AND AZIDE INTERMEDIATES 25 AND 26 63

4.1 COPPER-CATALYZED AZIDE-ALKYNE CYCLOADDITION (CUAAC) OF ALKYNE INTERMEDIATES 25 WITH AZIDE 44 63
4.2 COPPER-CATALYZED AZIDE-ALKYNE CYCLOADDITION (CUAAC) OF ALKYNE INTERMEDIATES 26 WITH ALKYNE 46 67

5 STAUDINGER-PHOSPHITE REACTION WITH INTERMEDIATE 26 71

6 STAUDINGER-PHOSPHITE REACTION WITH INTERMEDIATE 28 74

7 NMR SPECTRA 77

7.1 ^{1}H-, ^{13}C- AND ^{19}F-NMR OF COMPOUND 51 77
7.2 ^{1}H-, ^{13}C- AND ^{19}F-NMR OF COMPOUND 52 79
7.3 ^{1}H-, ^{13}C- AND ^{19}F-NMR OF COMPOUND 4 80
7.4 ^{1}H- AND ^{13}C-NMR OF COMPOUND 55 82
7.5 ^{1}H- AND ^{13}C-NMR OF COMPOUND 56 83
7.6 ^{1}H- AND ^{13}C-NMR OF COMPOUND 5 84
7.7 ^{1}H- AND ^{13}C-NMR OF COMPOUND 9 85
7.8 ^{1}H- AND ^{13}C-NMR OF COMPOUND 12 86
7.9 ^{1}H- AND ^{13}C-NMR OF COMPOUND 59 87
7.10 ^{1}H- AND ^{13}C-NMR OF COMPOUND 60 88
7.11 ^{1}H- AND ^{13}C-NMR OF COMPOUND 61 89
7.12 ^{1}H- AND ^{13}C-NMR OF COMPOUND 6 90
7.13 ^{1}H- AND ^{13}C-NMR OF COMPOUND 63 91
7.14 ^{1}H- AND ^{13}C-NMR OF COMPOUND 64 92
7.15 ^{1}H- AND ^{13}C-NMR OF COMPOUND 7 93
7.16 ^{1}H- AND ^{13}C-NMR OF COMPOUND 13 94
7.17 ^{1}H- AND ^{13}C-NMR OF COMPOUND 66 95
7.18 ^{1}H- AND ^{13}C-NMR OF COMPOUND 67 96
7.19 ^{1}H- AND ^{13}C-NMR OF COMPOUND 68 97
7.20 ^{1}H- AND ^{13}C-NMR OF COMPOUND 8 98
7.21 ^{1}H- AND ^{13}C-NMR OF COMPOUND 11A 99
7.22 ^{1}H- AND ^{13}C-NMR OF COMPOUND 11B 100
7.23 ^{1}H- AND ^{13}C-NMR OF COMPOUND 69 101
7.24 ^{1}H- AND ^{13}C-NMR OF COMPOUND 70 102
7.25 ^{1}H- AND ^{13}C-NMR OF COMPOUND 46 103

1 General methods

Unless specified otherwise, chemicals were purchased from Sigma Aldrich, Fisher Scientific, Carbosynth and Alfa Aesar and were used without further purification. Dry dichloromethane, tetrahydrofuran and *N*,*N*-dimethylformamide were purchased from VWR International (AR grade) and dried using solvent towers. Dry methanol and dry acetonitrile were purchased from Fisher Scientific. Reagent grade dichloromethane, ethyl acetate, methanol, acetonitrile, acetone, dimethyl sulfoxide and tetrahydrofuran were purchased from Fisher Scientific.

Analytical thin-layer chromatography (TLC) was performed on aluminium sheets precoated with silica gel 60 (F_{254}, Merck) and visualized under ultra-violet light (short and long-wave) and using potassium permanganate ($KMnO_4$) or vanillin stains. Silica gel was purchased from Sigma Aldrich (Tech Grade, pore size 60Å, 230-400 mesh).

^{1}H and ^{13}C NMR spectra were recorded in d_4-MeOD, $CDCl_3$ or D_2O on the following Bruker Avance instruments: DPX-300 300 MHz, DPX-400 400 MHz, DRX-500 500 MHz, AV-600 600 MHz or AV-700 700 MHz.

High-resolution mass spectra (HRMS) were obtained using electrospray ionization (ESI) on a MaXis UHR-TOF (Bruker Daltonics) or on Bruker MaXis (ESI-HR-MS).

Compounds were purified by preparative or semipreparative HPLC on Phenomenex synergi™ Polar RP 80Å (250 x 21.2 mm, 4μm) and Phenomenex synergi™ Polar RP 80Å (250 x 10.0 mm, 4μm) columns respectively. The mobile phase consisted of a gradient of water and acetonitrile or methanol (HPLC grade, containing 0.1 % trifluoroacetic acid or 0.1% formic acid) at a flow rate of 20 or 2.5 mL/min, with UV detection at 210, 254 and 280 nm.

The syntheses of the probes **3a-b** and **10** have been previously reported.[1, 2, 12]

2 Synthesis of chemical probes and compound characterization

2.1 Synthesis of probe 4

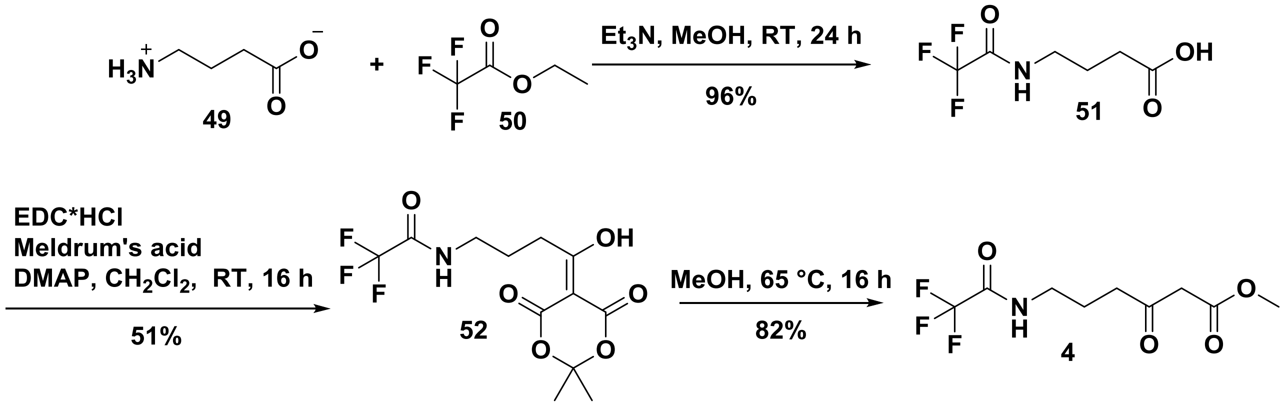

2.1.1 4-(2,2,2-trifluoroacetamido)butanoic acid (51)

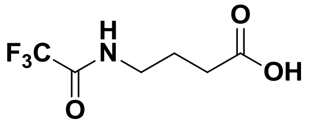

γ-Aminobutyric acid (**49**, 2.06 g, 20.0 mmol) was suspended in methanol (10 mL) and triethylamine (2.78 mL, 20.00 mmol) was added.[3] The solution was stirred at room temperature for 5 min before ethyl trifluoroacetate (**50**, 2.98 mL, 25.0 mmol) was slowly added. After 24 h the solvent was removed under reduced pressure and the residue was dissolved in water (10 mL). The aqueous solution was acidified with concentrated aqueous HCl (pH 1) and stirred for 15 minutes at room temperature. The aqueous solution was extracted with ethyl acetate (3 x 15 mL) and dried over $MgSO_4$. After filtration, ethyl acetate was removed *in vacuo* and the crude product was purified by column chromatography (MeOH : ethyl acetate 1 : 9) delivering compound **51** as white solid (3.81 g, 96%); R_f = 0.6 (EtOAc : MeOH 9 : 1).

^{1}H-NMR (400 MHz, MeOD): δ_H 4.97 (br s, 1H, OH), 3.33 (t, *J* 7.0 Hz, 2H, CH_2), 2.34 (t, *J* 7.3 Hz, 2H, CH_2), 1.85 (quint, *J* 7.2 Hz, 2H, CH_2); **^{13}C-NMR** (101 MHz, $CDCl_3$): δ_C 176.6 (RCOOH), 159.1 (q, *J* 36.9 Hz, RCONH), 117.5 (q, *J* 286.7 Hz, CF_3), 40.1 (CH_2), 31.9 (CH_2), 25.1 (CH_2); **^{19}F-NMR** (282 MHz, $CDCl_3$): δ_F -76.62; **HR-ESI-MS**: $[M+Na]^+$ calculated: 222.0348, found: 222.0357.

2.1.2 *N*-(4-(2,2-dimethyl-4,6-dioxo-1,3-dioxan-5-ylidene)-4-hydroxybutyl)-2,2,2-trifluoroacetamide (52)

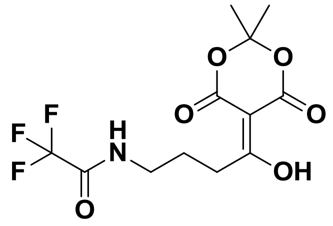

Compound **51** (2.00 g, 10.0 mmol), Meldrum's acid (1.45 g, 10.0 mmol) and *N*,*N*-dimethylamino pyridine (1.47 g, 12.1 mmol) were dissolved in anhydrous dichloromethane (70 mL). The reaction mixture was stirred for 10 minutes before *N*-(3-Dimethylaminopropyl)-*N'*-ethylcarbodiimide hydrochloride (2.16 g, 11.2 mmol) was added. The reaction mixture was stirred at room temperature for 18 hours. The organic solution was washed with aqueous HCl solution (1 M, 100 mL) and brine (100 mL). The organic phase was dried over $MgSO_4$ and filtered. The solvent was removed under reduced pressure and the residue was purified by column chromatography with a stepwise gradient from 100% dichloromethane to 100% ethyl acetate yielding product **52** (1.66 g, 51%); R_f = 0.25 (EtOAc).

^{1}H-NMR (300 MHz, $CDCl_3$): δ_H 15.32 (br s, 1H, OH), 7.29 (br s, 1H, NH), 3.42 (apt q, *J* 6.2 Hz, 2H, CH_2), 3.08 (t, *J* 7.3 Hz, 2H, CH_2), 2.08 – 1.95 (m, 2H, CH_2), 1.73 (s, 6H, CH_3); **^{13}C-NMR** (151 MHz, $CDCl_3$) : δ_C 196.2 (COH), 170.4 (RCOOR), 161.0 (RCOOR), 157.7 (q, *J* 37.0 Hz, RCONH), 115.9 (q, *J* 287.6 Hz, CF_3), 105.5 ($C(CH_3)_2$), 92.1 (R*C*COH), 38.9 (CH_2), 32.6 (CH_2), 26.9 (CH_3), 25.4 (CH_2); **^{19}F-NMR** (282 MHz, $CDCl_3$): δ_F -76.58; **HR-ESI-MS**: $[M+Na]^+$ calculated: 348.0665, found: 348.0663.

2.1.3 Methyl 3-oxo-6-(2,2,2-trifluoroacetamido)hexanoate (4)

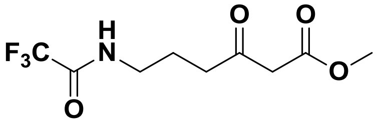

Compound **52** (1.30 g, 4.00 mmol) was dissolved in anhydrous methanol (70 mL) and the reaction mixture was refluxed for 16 h. The solvent was then evaporated under reduced pressure. The crude material was purified by semipreparative HPLC (elution gradient starting from 100% H_2O and linearly increasing to 100% MeCN over 40 minutes) to yield **4** (541 mg, 53%); R_t = 16.0 min.

^{1}H-NMR (400 MHz, $CDCl_3$): δ_H 6.91 (br s, 1H, NH), 3.74 (s, 3H, OCH_3), 3.48 (s, 2H, CH_2), 3.39 (apt q, *J* 6.3 Hz, 2H, CH_2), 2.67 (t, *J* 6.5 Hz, 2H, CH_2), 1.91 (quint, *J* 6.5 Hz, 2H, CH_2); **^{13}C-NMR** (101 MHz, $CDCl_3$) δ^C 202.7 (R_2CO), 168.0 (RCOOR), 157.6 (q, *J* 47.5 Hz, RCONH), 116.1 (q, *J* 260.3 Hz, CF_3), 52.7

(OCH_3), 48.9 (CH_2), 40.2 (CH_2), 39.3 (CH_2), 22.4 (CH_2); **^{19}F-NMR** (282 MHz, $CDCl_3$): δ_F -76.64; **HR-ESI-MS**: $[M+Na]^+$ calculated: 278.0611, found: 278.0599.

2.2 Synthesis of probes 5, 9 and 12

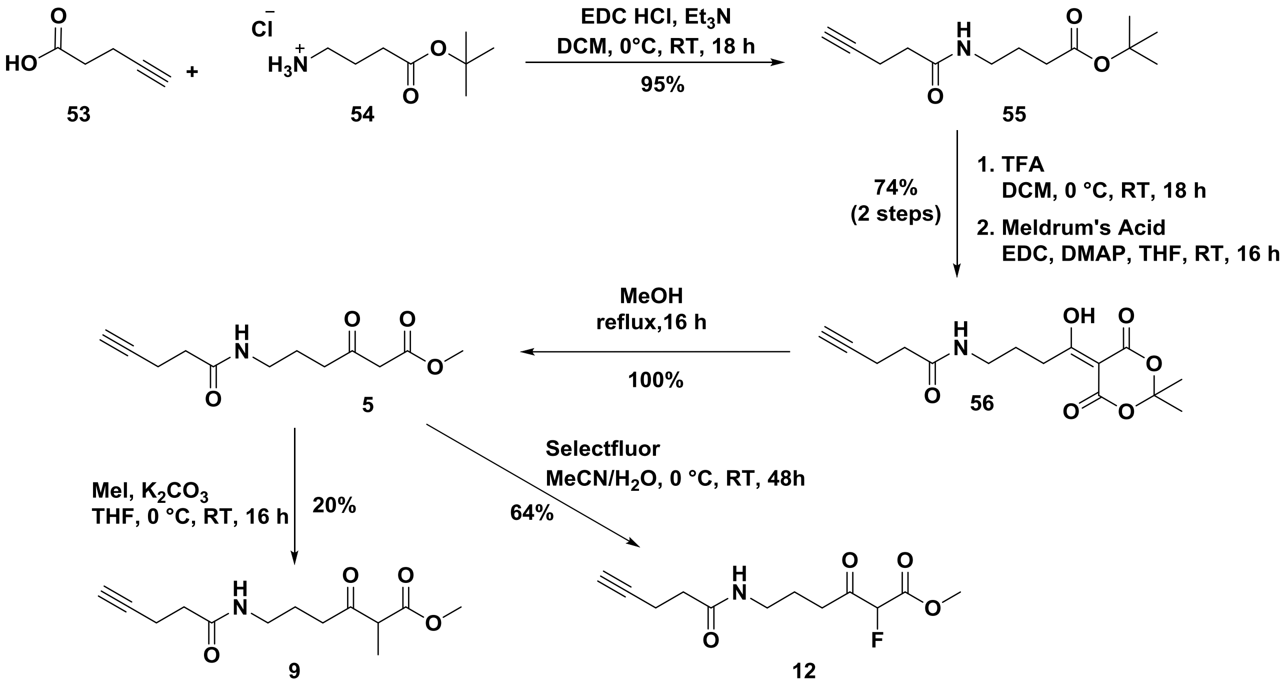

2.2.1 *Tert*-butyl 4-pent-4-ynamidobutanote (55)

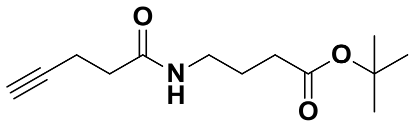

4-pentynoic acid (**53**) (1.96 g, 20.4 mmol), *tert*-butyl 4-aminobutanoate hydrochloride (**54**) (4.00 g, 20.4 mmol) and triethylamine (5.57 mL, 40.8 mmol) were dissolved in dry dichloromethane (100 mL) and cooled to 0 °C. *N*-(3-Dimethylaminopropyl)-*N′*-ethylcarbodiimide hydrochloride (3.72 g, 20.4 mmol) was added to the mixture and this was stirred at room temperature overnight. A diluted HCl solution (0.1 M, 100 mL) was then added to the reaction mixture, the phases were separated and the organic layer was further washed with saturated aqueous Na_2CO_3 (100 mL). The organic phase was dried over magnesium sulphate, filtered and concentrated to yield a crude product, which was purified by silica gel chromatography (dichloromethane : MeOH 97 : 3) to afford **55** as a colorless oil (4.60 g, 95%); R_f = 0.4 (dichloromethane : MeOH 9 : 1).

^{1}H-NMR (400 MHz, $CDCl_3$): δ_H 5.95 (br s, 1H, NH), 3.28 (apt q, *J* 6.8 Hz, 2H, CH_2), 2.55 (td, *J* 6.8, 2.8 Hz, 2H, CH_2), 2.36 (t, *J* 6.8 Hz, 2H, CH_2), 2.27 (t, *J* 7.2 Hz, 2H, CH_2), 1.98 (t, *J* 2.8 Hz, 1H, *H*CCR),

1.78 (tt, *J* 7.2, 6.8 Hz, 2H, CH_2), 1.42 (s, 9H, $C(CH_3)_3$); **^{13}C-NMR** (101 MHz, $CDCl_3$): δ_C 173.0 (RCONH), 171.1 (*C*O_2tBu), 83.1 (HC*CR*), 80.7 (*C*$(CH_3)_3$), 69.4 (H*C*CR), 39.2 (CH_2), 35.5 (CH_2), 33.1 (CH_2), 28.2 ($C(CH_3)_3$), 24.7 (CH_2), 15.0 (CH_2); **IR** (thin film): 3297 (N-H), 2977 (HC≡C), 2933 (C-H), 1726, 1646 (each C=O), 1548, 1439, 1367, 1256, 1190, 1084, 956, 845 cm^{-1}; **HR-ESI-MS**: $[M+H]^+$ calculated: 240.1600, found: 240.1603.

2.2.2 *N*-(4-(2,2-dimethyl-4,6-dioxo-1,3-dioxan-5-ylidene)-4-hydroxybutyl)pent-4-ynamide (56)

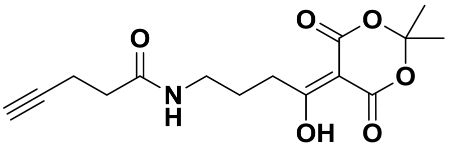

Tert-butyl 4-pent-4-ynamidobutanote (**55**) (4.60 g, 19.2 mmol) was dissolved in dry dichloromethane (40 ml) and trifluoroacetic acid (TFA) (5.75 mL, 76.8 mmol) was added dropwise at 0 °C. The mixture was stirred at room temperature for 16 hours. Dichloromethane and TFA were removed *in vacuo* (azeotropic mixture with toluene) and then freeze-dried to yield 4-pent-4-ynamidobutanoic acid as yellow oil (3.50 g, 100%).

^{1}H-NMR (400 MHz, $CDCl_3$): δ_H 9.75 (br s, 1H, COOH), 6.49 (br s, 1H, NH), 3.40 (td, *J* 6.8 , 10 Hz, 2H, CH_2), 2.54 (t, *J* 7.2 Hz, 2H, CH_2), 2.44 (td, *J* 2.8, 7.2 Hz, 2H, CH_2), 2.41 (t, *J* 7.2 Hz, 2H, CH_2), 2.04 (t, *J* 2.8 Hz, 1H, RCCH), 1.88 (tt, *J* 6.8, 7.2 Hz, 2H, CH_2).

This crude material was used for the next step without further purification: it was indeed dissolved in tetrahydrofuran (70 mL) together with Meldrum's acid (3.00 g, 21.1 mmol) and 4-(dimethylamino)pyridine (2.90 g, 24.0 mmol). *N*-(3-Dimethylaminopropyl)-*N'*-ethylcarbodiimide hydrochloride (3.27 g, 21.1 mmol) was added at 0 °C and then the reaction mixture was stirred overnight at room temperature. The solvent was removed *in vacuo* and the reaction crude was dissolved in dichloromethane (50 mL), washed with aqueous HCl (1 M, 50 mL) and deionized water (50 mL). The organic phase was dried over $MgSO_4$, filtered and concentrated. The crude residue was purified by silica gel chromatography with an isocratic elution of dichloromethane : MeOH 97 : 3). The desired product **56** was obtained as a pale yellow powder (4.40 g, 74%).

^{1}H-NMR (400 MHz, $CDCl_3$): δ_H 15.30 (br s, 1H, OH), 6.09 (br s, 1H, NH), 3.34 (apt q, *J* 6.8 Hz, 2H, CH_2), 3.07 (t, *J* 7.6 Hz, 2H, CH_2), 2.55-2.50 (m, *J* 7.6, 2.8 Hz, 2H, CH_2), 2.40 (t, *J* 7.2 Hz, CH_2, 2H,), 1.98 (t, *J* 2.8 Hz, 1H, CH), 1.92 (tt, *J* 6.8, 7.2 Hz, 2H, CH_2), 1.73 (s, 6H, $(CH_3)_2$); **^{13}C-NMR** (101 MHz, $CDCl_3$): δ_C 196.9 (COH), 171.3 (RCONH), 160.5 (RCOOR), 105.1 (*C*$(CH_3)_2$), 91.7 (HOC*C*R_2), 83.0 (CH*C*R), 69.3

(*C*HCR), 38.9 (CH_2), 36.2 (CH_2), 32.8 (CH_2), 26.8 (CH_3), 26.0 (CH_2), 14.9 (CH_2); **IR** (thin film): 3293 (N-H), 3190 (HC≡C), 2932 (C-H), 1713, 1650 (each C=O), 1549, 1359, 1280 1204, 1017, 972 cm^{-1}; **HR-ESI-MS**: $[M+H]^+$ calculated: 310.1291, found: 310.1289.

2.2.3 Methyl 3-oxo-6-pent-4-ynamidohexanoate (5)

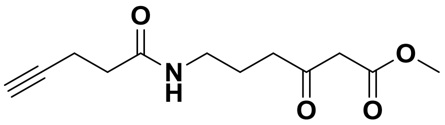

Compound **56** (4.40 g, 14.2 mmol) was dissolved in methanol (100 mL) and heated under reflux overnight under nitrogen atmosphere. The methanol was evaporated and the crude residue was purified by preparative HPLC (with an elution gradient starting from 100% H_2O and linearly increasing to 100% MeOH over 30 minutes). The desired product **5** (eluting at R_t = 11.2 min) was obtained as pale yellow powder (3.40 g, 100%).

^{1}H-NMR (400 MHz, $CDCl_3$): δ_H 5.99 (br s, 1H, NH), 3.74 (s, 3H, OCH_3), 3.45 (s, 2H, CH_2), 3.26 (apt q, *J* 6.8 Hz, 2H, CH_2), 2.61 (t, *J* 6.8 Hz, 2H, CH_2), 2.50 (td *J* 2.4, 7.2 Hz, 2H, CH_2), 2.36 (t, *J* 7.2 Hz, 1H, CH), 1.98 (t, *J* 2.4 Hz, 1H, CH), 1.77 (quint, *J* 6.8 Hz, 2H, CH_2); **^{13}C-NMR** (101 MHz, $CDCl_3$): δ_C 202.8 (R_2CO), 171.4 (RCONH), 167.7 (RCOOR), 83.1 (R*C*HC), 69.3 (RCH*C*), 52.4 (OCH_3), 48.9 (CH_2), 40.2 (CH_2), 38.5 (CH_2), 35.2 (CH_2), 23.2 (CH_2), 14.9 (CH_2); **IR** (thin film): 3340 (HC≡C), 3288 (N-H), 2954 (C-H), 1713, 1744, 1645 (each C=O), 1541, 1437, 1266, 1201, 1150, 1004, 841 cm^{-1}; **HR-ESI-MS**: $[M+H]^+$ calculated: 240.1230, found: 240.1229.

2.2.4 Methyl 2-methyl-3-oxo-6-pent-4-ynamidohexanoate (9)

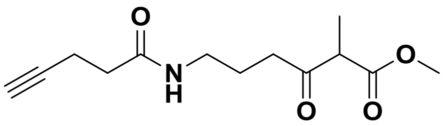

Compound **5** (1.11 g, 4.64 mmol) was dissolved in THF (150 mL) and potassium carbonate (1.66 g, 12.06 mmol) was added. Methyl iodide (0.29 mL, 4.64 mmol) was added dropwise at 0 °C. After one hour the reaction mixture was allowed to warm at room temperature and was left stirring overnight. The reaction was quenched by addition of water (20 mL). THF and water were removed by evaporation/freeze-drying. The crude residue was purified by preparative HPLC (elution gradient starting from 100% H_2O and linearly increasing to 100% MeOH over 30 minutes) and **9** was yielded as white solid (238 mg, 20%, R_t = 10.9 min).

^{1}H-NMR (400 MHz, $CDCl_3$): δ_H 5.90 (br s, 1H, NH), 3.71 (s, 3H, OCH_3), 3.53 (q, *J* 7.2 Hz, 1H, CH), 3.31-3.19 (m, 2H, CH_2), 2.56 (t, *J* 6.8 Hz, 2H, CH_2), 2.50 (td, *J* 2.4, 6.8 Hz, 2H, CH_2), 2.35 (t, *J* 6.8 Hz, 2H, CH_2), 1.98 (t, *J* 2.4 Hz, 1H, HCCR), 1.79 (tt, *J* 6.8, 6.8 Hz, 2H, CH_2), 1.31 (d, *J* 7.2, 3H, CCH_3); **^{13}C-NMR** (101 MHz, $CDCl_3$): δ_C 207.2 (R_2CO), 205.8 (RCOOR), 171.1 (RCONH), 83.1 (HC*C*R), 69.4 (H*C*CR), 52.7 (OCH_3), 52.6 (CH_3*C*H), 38.9 (CH_2), 35.5 (CH_2), 31.6 (CH_2), 23.3 (CH_2), 15.0 (CH_2), 12.9 (CH*C*H_3); **IR** (thin film): 3383 (HC≡C), 3288 (N-H), 1944 (C-H), 1740 (ketone C=O), 1712 (ester C=O), 1646 (amide C=O), 1544, 1436, 1376, 1323, 1265, 1206, 1176, 1126, 1084, 1019, 963, 865 cm^{-1}; **HR-ESI-MS**: $[M+H]^+$ calculated: 254.1387, found: 254.1385.

2.2.5 Methyl 2-fluoro-3-oxo-6-(pent-4-ynamido)hexanoate (12)

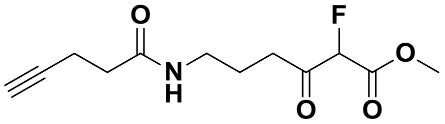

Compound **5** (300 mg, 1.25 mmol) was dissolved in a 1:1 mixture of acetonitrile and water (8 mL) and Selectfluor (578 mg, 1.63 mmol) was added portionwise at 0 °C. The reaction was stirred at room temperature for 48 h. It was quenched by addition of water (8 mL) and then extracted with dichloromethane (2 x 20 mL). The organic layer was dried over $MgSO_4$, filtered and evaporated under reduced pressure. The crude residue was purified by preparative HPLC (elution gradient starting from 100% H_2O and linearly increasing to 40% acetonitrile over 30 minutes and then to 100% MeCN over 3 minutes) and **12** was yielded as a white powder (164 mg, 64 %, R_t = 10.7 min).
^{1}H-NMR (400 MHz, $CDCl_3$): δ_H 5.58 (br s, 1H, NH), 5.27 (d, *J* 46.4 Hz, 1H, HCF), 3.68 (s, 3H, OCH_3), 3.30 (apt q, *J* 6.8 Hz, 2H, CH_2), 2.85-2.64 (m, 2H, CH_2), 2.52 (td, *J* 6.3, 2.4 Hz, 2H, CH_2), 2.36 (t, *J* 6.8 Hz, 2H, CH_2), 2.03 (t, *J* 2.4 Hz, 1H, HCCR), 1.86 (quint, *J* 6.8 Hz, 2H, CH_2); **^{13}C-NMR** (101 MHz, $CDCl_3$): δ_C 201.0 (R_2CO), 171.3 (RCONH), 164.6 (RCOOR), 91.9-90.5 (*J* 197.60 Hz, HCF), 82.9 (R*C*CH), 69.5 (RC*C*H), 53.3 (CH_3), 38.5 (CH_2), 35.7 (CH_2), 35.4 (CH_2), 22.7 (CH_2), 14.9 (CH_2); **^{19}F-NMR** (282 MHz, $CDCl_3$): δ_F -114.15: **HR-ESI-MS**: $[M+Na]^+$ calculated: 280.0961, found: 280.0963.

2.3 Synthesis of probe 6

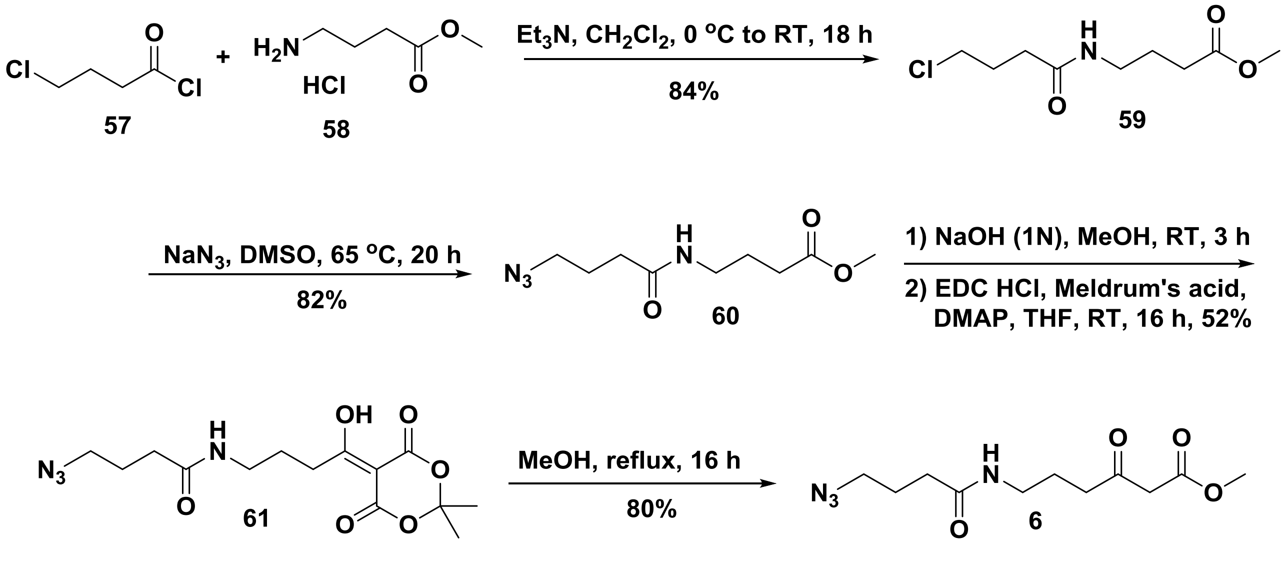

2.3.1 Methyl 4-(4-chlorobutanamido)butanoate (59)

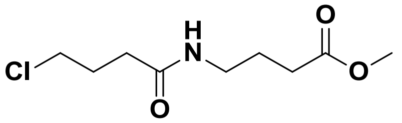

Triethylamine (8.40 mL, 30.0 mmol) was added to a solution of methyl 4-aminobutyrate hydrochloride (**58**, 1.54 g, 10.0 mmol) in dichloromethane (60 mL). The solution was cooled to 0 °C and 4-chlorobutyryl chloride (**57**, 1.23 mL, 1.55 g, 11 mmol.) was slowly added. After 15 minutes the ice-bath was removed and the reaction mixture was stirred for 16 h at room temperature. The solution was washed with 0.1 M hydrochloric acid (50 mL), water (50 mL) and brine (50 mL) and dried over $MgSO_4$. After filtration the solvent was removed under reduced pressure. Methyl 4-(4-chlorobutanamido)butanoate (**59**) was obtained as pale oil (1.81 g, 82%) and used without further purification; R_f = 0.35 (EtOAc : cyclohexane 3 : 1).

^{1}H-NMR (400 MHz, $CDCl_3$): δ_H 6.12 (s, 1H, NH), 3.65 (s, 3H, CH_3), 3.57 (t, *J* 6.2 Hz, 2H, CH_2), 3.26 (apt q, *J* 6.5 Hz, 2H, CH_2), 2.34 (t, *J* 7.2 Hz, 2H, CH_2), 2.33 (t, *J* 7.2 Hz, 2H, CH_2), 2.10 – 2.04 (m, 2H, CH_2), 1.81 (quint, *J* 7.1 Hz, 2H, CH_2); **^{13}C-NMR** (101 MHz, $CDCl_3$): δ_C 174.0 (*C*OOMe), 172.1 (RCONH), 51.8 (OCH_3), 44.6 (CH_2Cl), 39.1 (CH_2NH), 33.3 (*C*H_2RCONH), 31.5 (*C*H_2COOMe), 28.2 (CH_2), 24.6 (CH_2); **HR-ESI-MS**: $[M+H]^+$ calculated: 222.0891, found: 222.0895.

2.3.2 Methyl 4-(4-azidobutanamido)butanoate (60)

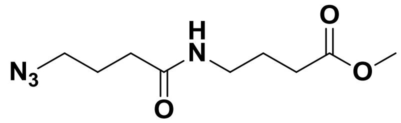

Sodium azide (1.06 g, 16.3 mmol) was added to a solution of methyl 4-(4-chlorobutanamido)butanoate (**59**) (1.81 g, 8.15 mmol) in dimethyl sulfoxide (50 mL). The reaction mixture was stirred at 70 °C for 18 h. The reaction mixture was allowed to cool to room temperature and water (100 mL) was added. The aqueous solution was extracted with ethyl acetate (4 x 50 mL). The combined organic phases were washed with water (2 x 100 mL) and brine (100 mL) and dried over $MgSO_4$. The $MgSO_4$ was filtered off and the ethyl acetate was removed in *vacuo* to yield the crude product. The crude product was purified by column chromatography (MeOH : ethyl acetate 1 : 9) delivering the azide **60** (1.52 g, 82%) as pale oil; R_f = 0.3 (EtOAc : cyclohexane 3 : 1).

^{1}H-NMR (400 MHz, $CDCl_3$) δ_H: 6.06 (br s, 1H, NH), 3.64 (s, 3H, OCH_3), 3.31 (t, *J* 6.5 Hz, 2H, CH_2), 3.25 (apt q, *J* 6.7 Hz, 2H, CH_2), 2.34 (t, *J* 7.1 Hz, 2H, CH_2), 2.23 (t, *J* 7.2 Hz, 2H, CH_2), 1.93 – 1.84 (m, 2H, CH_2), 1.80 (quint, *J* 7.0 Hz, 3H, CH_2); **^{13}C-NMR** (101 MHz, $CDCl_3$): δ_C 174.0 (*C*OOMe), 172.0 (RCONH), 51.8 (OCH_3), 50.9 (CH_2N_3), 39.0 (CH_2NH), 33.2 (*C*H_2CONH), 31.5 (*C*H_2COOMe), 24.9 (CH_2), 24.6 (CH_2); **HR-ESI-MS**: $[M+Na]^+$ calculated: 251.1115, found: 251.1104.

2.3.3 4-azido-*N*-(4-(2,2-dimethyl-4,6-dioxo-1,3-dioxan-5-ylidene)-4-hydroxybutyl)butanamide (61)

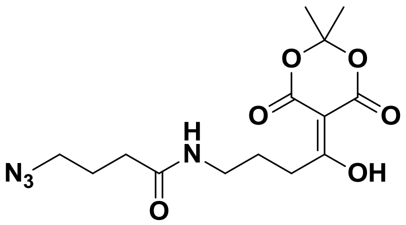

Methyl 4-(4-azidobutanamido)butanoate (**60**) (1.10 g, 4.82 mmol) was dissolved in methanol (9 mL) and aqueous sodium hydroxide solution (1 M, 15 mL) was added. The reaction mixture was stirred at room temperature for 2 h. The solvent was evaporated under reduced pressure and the aqueous solution was brought to pH 1 with concentrated hydrochloric acid. Afterwards the aqueous solution was extracted with ethyl acetate (3 x 10 mL) and the organic phase was dried over $MgSO_4$. After filtration, ethyl acetate was removed *in vacuo* to yield 4-(4-azidobutanamido)butanoic acid (836 mg, 81%). The product was used without further purification.

^{1}H-NMR (400 MHz, MeOD): δ_H 3.33 (t, *J* 6.8 Hz, 2H, CH_2), 3.21 (t, *J* 6.9 Hz, 2H, CH_2), 2.33 (t, *J* 7.4 Hz, 2H, CH_2), 2.27 (t, *J* 7.4 Hz, 2H, CH_2), 1.90 – 1.74 (m, 4H, CH_2); **^{13}C-NMR** (101 MHz, $CDCl_3$): δ_C 177.6 (COOH), 173.1 (RCONH), 50.8 (CH_2N_3), 39.1 (CH_2NH), 33.2 (*C*H_2COOH), 31.5 (*C*H_2CONH), 24.9 (CH_2), 24.5 (CH_2); **HR-ESI-MS**: $[M+Na]^+$ calculated: 237.0958, found: 237.0950.

4-(4-Azidobutanamido)butanoic acid (398 mg, 1.85 mmol), Meldrum's acid (320 mg, 2.22 mmol) and *N*,*N*-dimethylamino pyridine (340 mg, 2.78 mmol) were dissolved in anhydrous dichloromethane (30 mL). The reaction mixture was stirred for 10 min. before *N*-(3-dimethylaminopropyl)-*N'*-ethylcarbodiimide hydrochloride (468 mg, 2.44 mmol) was added. The reaction mixture was stirred at room temperature for 18 h. The organic solution was washed with HCl solution (1 M, 30 mL) and brine (30 mL). The organic phase was dried over $MgSO_4$ and filtered. The solvent was removed under reduced pressure and the residue was purified by column chromatography (ethyl acetate : cyclohexane 1 : 3 to ethyl acetate) to afford compound **61** (178 mg, 52%); R_f = 0.03 (EtOAc).

^{1}H-NMR (400 MHz, $CDCl_3$): δ_H 15.30 (br s, 1H, OH), 6.06 (s, 1H, NH), 3.35 – 3.30 (m, 4H, CH_2N_3, CH_2NH), 3.11 – 3.02 (m, 2H, CH_2C), 2.28 (t, *J* 7.2 Hz, 2H, CH_2CO), 1.90 – 1.81 (m, 4H, 2xCH_2), 1.73 (s, 6H, CH_3); **^{13}C-NMR** (101 MHz, $CDCl_3$): δ_C 197.0 (COH), 172.1 (RCONH), 160.8 (RCOOR), 105.3 ($C(CH_3)_2$), 91.9 (R_2*C*CROH), 50.9 (CH_2N_3), 38.6 (CH_2NH), 33.3 (*C*H_2COH), 32.9 (*C*H_2CO), 26.9 (2 x CH_3), 26.3 (CH_2), 24.9 (CH_2); **HR-ESI-MS**: $[M+Na]^+$ calculated: 363.1275, found: 363.1274.

2.3.4 Methyl 6-(4-azidobutanamido)-3-oxohexanoate (6)

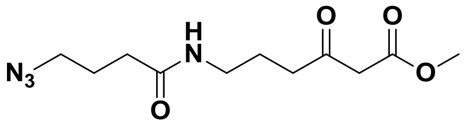

Compound **61** (178 mg, 0.52 mmol) was dissolved in anhydrous methanol (10 mL) and the reaction mixture was refluxed for 16 h. The solvent was then evaporated under reduced pressure and the crude product purified by semipreparative HPLC (elution gradient starting from 100% H_2O and linearly increasing to 100% MeCN over 40 minutes) to yield methyl 6-(4-azidobutanamido)-3-oxohexanoate (53.0 mg, 38%, R_t = 15.9 min).

^{1}H-NMR (400 MHz, $CDCl_3$): δ_H 5.78 (br s, 1H, NH), 3.74 (s, 3H, CH_3), 3.46 (s, 2H, CH_2), 3.35 (t, *J* 6.6 Hz, 2H, CH_2), 3.26 (apt q, *J* 6.6 Hz, 2H, CH_2), 2.61 (t, *J* 6.8 Hz, 2H, CH_2), 2.25 (t, *J* 7.2 Hz, 2H, CH_2), 1.91 (quint, *J* 6.9 Hz, 2H, CH_2), 1.82 (quint, *J* 6.9 Hz, 2H, CH_2); **^{13}C-NMR** (101 MHz, $CDCl_3$): δ_C 202.7

(R_2CO), 172.1 (RCONH), 167.9 (RCOOR), 52.5 (OCH_3), 50.9 (CH_2), 49.0 (CH_2), 40.4 (CH_2), 38.8 (CH_2), 33.3 (CH_2), 24.9 (CH_2), 23.3 (CH_2); **HR-ESI-MS**: $[M+Na]^+$ calculated: 293.1220, found: 293.1212.

2.4 Synthesis of probes 7 and 13

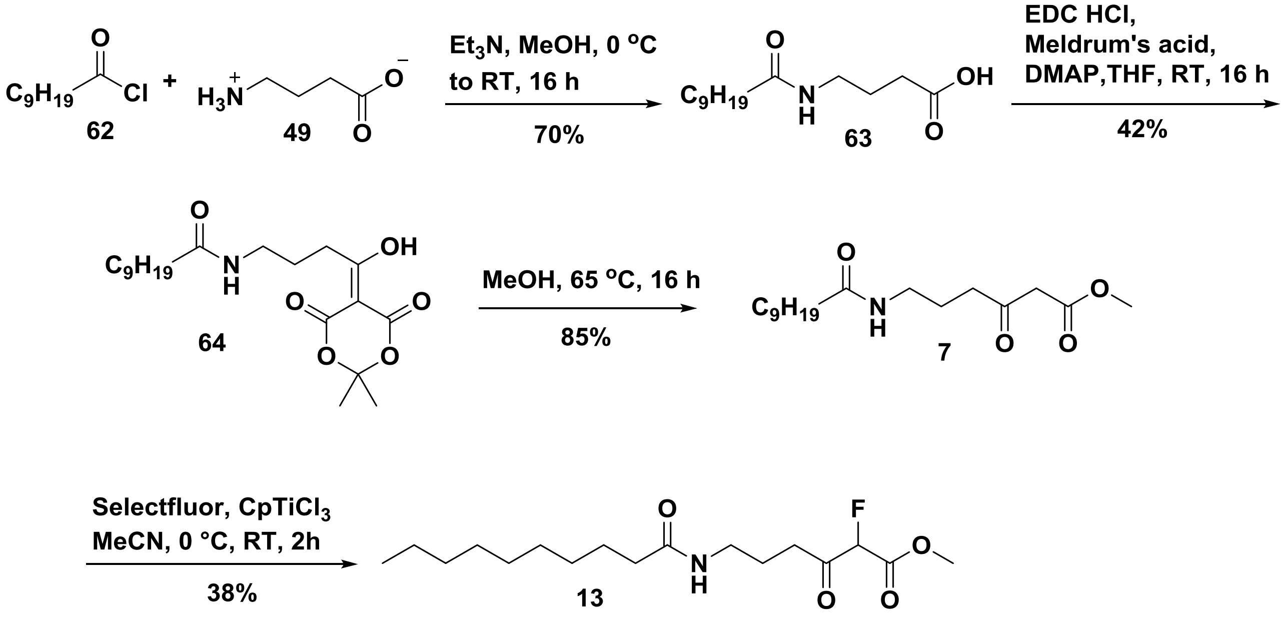

2.4.1 1 4-decanamidobutanoic acid (63)

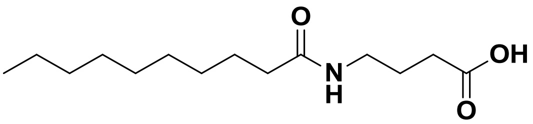

γ-Aminobutyric acid (**49**, 1.00 g, 9.7 mmol) was suspended in dry methanol (60 mL) in the presence of triethylamine (5.75 mL, 29 mmol). The mixture was cooled to 0 °C and decanoyl chloride (**62**, 2.1 mL, 9.9 mmol) was added dropwise. The reaction was stirred at 0 °C for 1 h and at room temperature for 16 h. The solvent was evaporated and the crude *N*-decanoyl-γ-butyric acid (triethylammonium salt) was suspended in water (100 mL), a solution of aqueous 1 M HCl was added to adjust the pH to 2. The mixture was then extracted with $CHCl_3$ (3 x 100 mL). The organic phase was dried over $MgSO_4$, filtered and evaporated to afford **63** as white powder (2.00 g, 80%). **^{1}H-NMR** (400 MHz, $CDCl_3$): δ_H 5.77 (br s, 1H, NH), 3.33 (apt q, *J* 6.4 Hz, 2H, $NHCH_2$), 2.40 (t, *J* 6.9 Hz, 2H, CH_2COOH), 2.18 (t, *J* 7.5 Hz, 2H, CH_2CONH), 1.85 (quint, *J* 6.8 Hz, 2H, CH_2-CH_2COOH), 1.63-1.58 (m, 2H, CH_2CH_2CONH), 1.27 (br s, 12H, $(CH_2)_6$), 0.87 (t, *J* 6.8 Hz, 3H, CH_3); **^{13}C-NMR** (101 MHz, $CDCl_3$): δ_C 176.8 (RCONH), 174.3 (RCOOR), 38.9 ($NHCH_2$), 36.9 (CH_2CONH), 32.0 (CH_2), 31.9

(CH_2COOH), 29.8, 29.6, 29.5, 29.4 (each CH_2), 25.9 (CH_2-CH_2COOH), 25.2 (CH_2), 22.8 (CH_2CH_3), 14.2 (CH_3); **HR-ESI-MS**: $[M+H]^+$ calculated: 258.2064, found: 258.2064.

2.4.2 *N*-(4-(2,2-dimethyl-4,6-dioxo-1,3-dioxan-5-ylidene)-4-hydroxybutyl)decanamide (64)

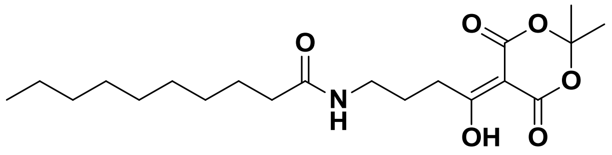

Compound **63** (2.00 g, 7.76 mmol), Meldrum's acid (1.12 g, 7.76 mmol) and 4-(dimethylamino)pyridine (1.05 g, 9.62 mmol) were dissolved in tetrahydrofuran (150 ml). *N*-(3-Dimethylaminopropyl)-*N'*-ethylcarbodiimide hydrochloride (1.63 g, 8.54 mmol) was added at 0 °C and the reaction mixture stirred overnight at room temperature. The solvent was removed *in vacuo* and the reaction crude was dissolved in dichloromethane (50 mL), washed with 1 M HCl solution (50 mL) and water (50 mL). The organic phase was dried over $MgSO_4$ and evaporated. The crude was purified by chromatography column using a stepwise gradient from 100% cyclohexane to 100% of EtOAc and **64** was obtained as a pale yellow powder (1.02 g, 37%); R_f = 0.04 (EtOAc). **^{1}H-NMR** (400 MHz, $CDCl_3$): δ_H 15.2 (br s, 1H, OH), 6.09 (br s, 1H, NH), 3.29 (apt q, *J* 6.2 Hz, 2H, NHCH_2), 3.04 (t, *J* 7.4 Hz, 2H, CH_2COH), 2.11 (t, *J* 7.4 Hz, 2H, CH_2CONH), 1.88 (quint, *J* 6.9 Hz, 2H, CH_2-CH_2COH), 1.68 (s, 6H, $(CH_3)_2$), 1.61-1.54 (m, 2H, CH_2-CH_2CONH), 1.24 (br s, 12H, $(CH_2)_6$), 0.83 (t, *J* 6.6 Hz, 3H, CH_3); **^{13}C-NMR** (101 MHz, $CDCl_3$): δ_C 197.0 (RCOH), 173.5 (RCONH), 170.4, 160.6 (RCOOR), 105.1 (*C*$(CH_3)_2$), 91.7 (*C*$(CO)_2$), 41.1 ($NHCH_2$), 39.0 (*C*H_2CONH), 38.3 (CH_2), 36.8 (CH_2), 32.8 (CH_2), 31.8 (CH_2), 29.3 (CH_2), 29.2 (CH_2), 26.8 ($(CH_3)_2$), 26.3 (*C*H_2COH), 25.7 (*C*H_2-CH_2COH), 22.7 (*C*H_2CH_3), 14.1 (CH_3); **HR-ESI-MS**: $[M-H]^-$ calculated: 382.2230, found: 382.2235.

2.4.3 *N*-(4,6-dioxoheptyl)decanamide (7)

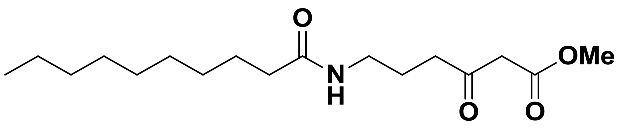

Compound **64** (1.02 g, 2.66 mmol) was dissolved in methanol (45 mL) and heated under reflux overnight under argon atmosphere. The solvent was evaporated and the crude was purified using preparative HPLC (elution gradient starting from 85% H_2O and linearly increasing to 65% MeOH

over 10 minutes and then to 80% MeOH over 30 minutes). Compound **7** was obtained as white powder (800 mg, 96%); R_t = 20.1 min.

^{1}**H-NMR** (400 MHz, $CDCl_3$): δ_H 5.83 (br s, 1H, NH), 3.73 (s, 3H, OCH_3), 3.46 (s, 2H, $COCH_2CO$), 3.26 (apt q, *J* 6.6 Hz, 2H, $NHCH_2$), 2.61 (t, *J* 6.8 Hz, 2H, CH_2CO), 2.16 (t, *J* 7.6 Hz, 2H, CH_2CONH), 1.77-1.82 (m, 2H, CH_2-CH_2CO), 1.60-1.56 (m, 2H, CH_2-CH_2CONH), 1.27 (br s, 12H, $(CH_2)_6$), 0.87 (t, *J* 6.1 Hz, 3H, CH_3); ^{13}**C-NMR** (101 MHz, $CDCl_3$): δ_C 202.7 (R_2CO), 173.5 (RCONH), 167.8 (RCOOR), 52.6 (OCH_3), 49.1 ($COCH_2CO$), 40.5 (CH_2CO), 38.7 ($NHCH_2$), 36.9 (CH_2CONH), 31.8 (CH_2-CH_2CO), 29.6, 29.5, 25.9, 25.8, 23.4, 22.8 (each CH_2), 22.7 (CH_2CH_3), 14.2 (CH_3); **HR-ESI-MS**: $[M-H]^-$ calculated: 312.2175, found: 312.2179.

2.4.4 Methyl 6-decanamido-2-fluoro-3-oxohexanoate (13)

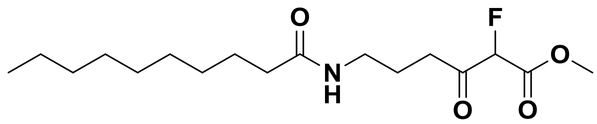

Selectfluor (123 mg, 0.34 mmol) was added to **7** (100 mg, 0.31 mmol) and cyclopentadienyltitanium(IV) trichloride (3.39 mg, 0.015 mmol) dissolved in MeCN (3 mL).[4] After stirring the reaction at room temperature for 2 hours, the reaction mixture was filtered and the solvent evaporated. The crude was purified by HPLC (elution gradient starting from 85% H_2O and linearly increasing to 65% MeOH over 10 minutes and then to 80% MeOH over 30 minutes). Compound **13** was obtained as a white powder (42.8 mg, 38%); R_t = 20.3 min.

^{1}**H-NMR** (400 MHz, $CDCl_3$): δ_H 5.56 (br s, 1H, NH), 5.28 (d, *J* 48.5 Hz, 1H, HCF), 3.87 (s, 3H, OCH_3), 3.30-3.20 (m, 2H, $NHCH_2$), 2.80-2.67 (m, 2H, CH_2), 2.16 (t, *J* 7.8 Hz, 2H, CH_2CONH), 1.87-1.82 (m, 2H, CH_2), 1.62-1.57 (m, 2H, CH_2), 1.35-1.21 (m, 12H, $(CH_2)_6$), 0.90-0.86 (m, *J* 6.8, 3H, CH_3); **HR-ESI-MS**: $[M-H]^-$ calculated: 354.2051, found: 354.2047.

2.5 Synthesis of probe 8

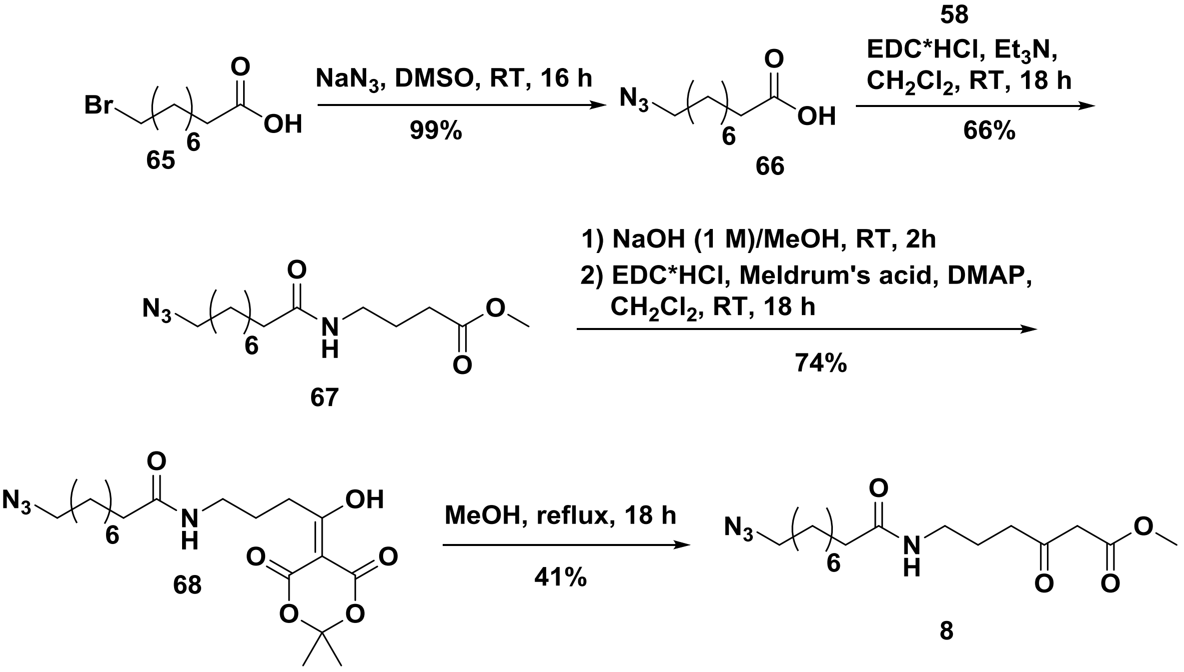

2.5.1 Synthesis of 10-azidodecanoic acid (66)

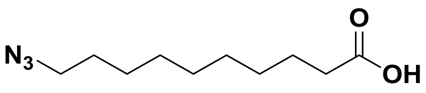

Sodium azide (602 mg, 9.27 mmol) was added to a solution of 10-bromodecanoic acid (**65**, 2.00 g, 8.43 mmol) in dimethyl sulfoxide (50 mL). The reaction mixture was stirred at room temperature for 18 h and afterwards water (100 mL) was added. The aqueous solution was extracted with ethyl acetate (4 x 50 mL). The combined organic phases were washed with water (2 x 100 mL) and brine (100 mL) and dried over $MgSO_4$. The $MgSO_4$ was filtered off and the ethyl acetate was removed *in vacuo* to yield azide **66** (1.67 g, 99%).[5]

^{1}H-NMR (400 MHz, $CDCl_3$): δ_H 10.33 (br s, 1H, COOH), 3.28 (t, *J* 6.90 Hz, 2H, CH_2N_3), 2.37 (t, *J* 7.40 Hz, 2H, CH_2COOH), 1.74 - 1.58 (m, 4H, CH_2), 1.45 - 1.27 (m, 10H, CH_2); **^{13}C-NMR** (101 MHz, $CDCl_3$): δ_C 179.9 (COOH), 51.6 (CH_2), 34.2 (CH_2), 29.4 (CH_2), 29.2 (CH_2), 29.2 (CH_2), 28.9 (CH_2), 26.8 (CH_2), 24.8 (CH_2) ; **HR-ESI-MS**: $[M+Na]^+$ calculated: 236.1369, found: 236.1370.

2.5.2 Synthesis of methyl 4-(10-azidodecanamido)butanoate (67)

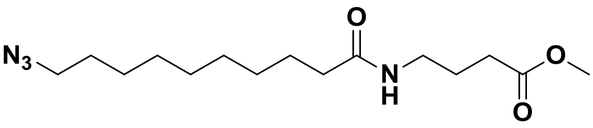

A solution of crude **66** (1.67 g, 7.83 mmol), methyl 4-aminobutyrate hydrochloride (**58**) (1.57 g, 10.2 mmol) and triethylamine (4.00 mL, 28.2 mmol) in dichloromethane (50 mL) was cooled to 0 ^{o}C before 1-(3-dimethylaminopropyl)-3-ethylcarbodiimide hydrochloride (1.96 g, 10.2 mmol) was added. The reaction mixture was stirred for 1 h at 0 ^{o}C and then 16 h at room temperature. The reaction mixture was extracted with hydrochloric acid (1 M, 30 mL) and water (30 mL) and dried over $MgSO_4$. The solvent was removed under reduced pressure to obtain the crude product. The crude was purified by column chromatography (cyclohexane : ethyl acetate gradient from 2 : 1 to 1 : 1). The product **67** was obtained as a white solid in a yield of 66% (1.61 g); R_f = 0.2 (EtOAc: cyclohexane 1 : 1).

^{1}H-NMR (400 MHz, $CDCl_3$): δ_H 5.75 (br s, 1H, NH), 3.66 (s, 3H, OCH_3), 3.28 - 3.21 (m, 4H, 2xCH_2), 2.35 (t, *J* 7.2 Hz, 2H, CH_2), 2.13 (t, *J* 7.3 Hz, 2H, CH_2), 1.84 (quint, *J* 7.0 Hz, 2H, CH_2), 1.66 - 1.55 (m, 4H, CH_2), 1.41 - 1.26 (m, 10 H, CH_2); **^{13}C-NMR** (101 MHz, $CDCl_3$): δ_C 174.1 (RCONH), 173.3 (RCOOR), 51.8 (OCH_3), 51.6 (CH_2N_3), 39.0 (CH_2), 36.9 (CH_2), 31.6 (CH_2), 29.4 (CH_2), 29.4 (CH_2), 29.2 (CH_2), 28.2 (CH_2), 26.8 (CH_2), 25.8 (CH_2), 24.8 (CH_2); **HR-ESI-MS**: $[M+Na]^+$ calculated: 335.2054, found: 335.2057.

2.5.3 Synthesis of 10-azido-*N*-(4-(2,2-dimethyl-4,6-dioxo-1,3-dioxan-5-ylidene)-4-hydroxybutyl)decanamide (68)

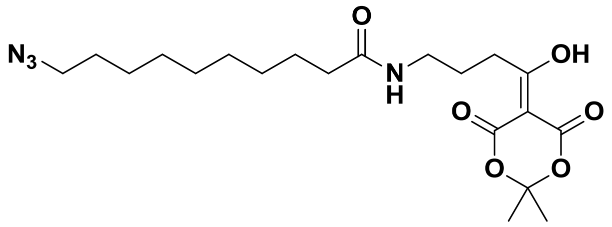

Compound **67** (1.54 g, 4.93 mmol) was dissolved in MeOH (10 mL) and aqueous sodium hydroxide solution (1M, 15 mL) was added.[6] The reaction mixture was stirred for 2 h at room temperature. The organic solvent was removed under reduced pressure and the remaining solution was acidified to pH 1 with hydrochloric acid (1M). The obtained suspension was extracted with ethyl acetate (3 x 20 mL). The organic phase was dried over $MgSO_4$, filtered and the solvent was removed under reduced pressure. 4-(10-azidodecanamido)butanoic acid was obtained as white solid and used without further purification for the next step (1.24 g).

^{1}H-NMR (400 MHz, $CDCl_3$): δ_H 5.77 (s, 1H, NH), 3.35 (apt q, *J* 6.5 Hz, 2H, CH_2), 3.25 (t, *J* 7.0 Hz, 2H, CH_2), 2.40 (t, *J* 7.0 Hz, 2H, CH_2), 2.18 (t, *J* 7.5 Hz, 2H, CH_2), 1.86 (quint, *J* 6.9 Hz, 2H, CH_2), 1.70 - 1.52 (m, 4H, CH_2), 1.45 - 1.21 (m, 10H, CH_2); **^{13}C-NMR** (101 MHz, $CDCl_3$): δ_C 176.7 (RCONH), 174.5

(RCOOH), 51.4 (CH_2N_3), 38.9 (CH_2), 36.5 (CH_2), 31.5 (CH_2), 29.2 (CH_2), 29.1 (CH_2), 29.0 (CH_2), 28.7 (CH_2), 26.6 (CH_2), 25.7 (CH_2) 24.5 (CH_2); **HR-ESI-MS**: $[M+Na]^+$ calculated: 321.1897, found: 321.1891.

4-(10-azidodecanamido)butanoic acid (1.00 g, 3.35 mmol), Meldrum's acid (580 mg, 4.02 mmol) and *N*,*N*-dimethylamino pyridine (614 mg, 5.02 mmol) in dichloromethane (80 mL) were stirred at room temperature for 20 min. Then 1-(3-dimethylaminopropyl)-3-ethylcarbodiimide hydrochloride (822 mg, 4.29 mmol) was added. The reaction mixture was stirred for 16 h at room temperature and washed with hydrochloric acid (1 M, 50 mL) and water (50 mL). The phases were separated and dried over $MgSO_4$, filtered and concentrated. The crude product was purified by column chromatography (gradient from CH_2Cl_2 to CH_2Cl_2 : ethyl acetate 3 : 1). **68** was obtained as a light yellow solid in (915 mg, 65%); R_f = 0.04 (EtOAc).

^{1}H-NMR (400 MHz, $CDCl_3$): δ_H 15.30 (br s, 1H, COOH), 5.94 (br s, 1H, NH), 3.32 (apt q, *J* 6.2 Hz, 2H), 3.24 (t, *J* 6.9 Hz, 2H, CH_2), 3.07 (t, *J* 7.4 Hz, 2H, CH_2), 2.17 (t, *J* 7.6 Hz, 2H, CH_2), 1.92 (quint, *J* 7.0 Hz, 2H, CH_2), 1.74 (s, 6H, CH_3), 1.68 - 1.52 (m, 4H, CH_2), 1.28 (br s, 10H, CH_2); **^{13}C-NMR** (101 MHz, $CDCl_3$): δ_C 197.1 (R_2CO) 173.5 (RCONH) 160.7 (RCOH) 105.3 (R_2*C*COH) 91.9 ($C(CH_3)_2$) 51.6 (CH_2N_3), 38.5 (CH_2), 36.9 (CH_2), 32.9 (CH_2), 29.3 (CH_2), 29.2 (CH_2), 28.9 (CH_2), 26.9 (CH_2), 26.8 (CH_2), 26.4 (CH_2), 25.8 (CH_2); **HR-ESI-MS**: $[M+Na]^+$ calculated: 447.2214, found: 447.2190.

2.5.4 Synthesis of methyl 6-(10-azidodecanamido)-3-oxohexanoate (8)

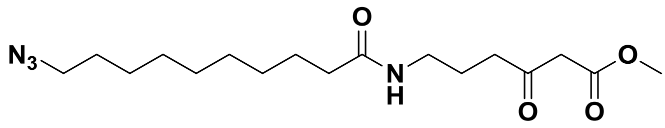

68 (915 mg, 2.15 mmol) was dissolved in dry methanol (20 mL) and the solution was refluxed for 16 h. The solvent was removed under reduced pressure and the crude product was purified by semipreparative HPLC (elution gradient starting from 100% H_2O and linearly increasing to 100% MeCN over 30 minutes) to yield **8** (487 mg, 41%); R_t = 18.3 minutes.

^{1}H-NMR (400 MHz, $CDCl_3$, MeOD): δ_H 3.67 (s, 3H, CH_3), 3.19 – 3.14 (m, 4H, CH_2), 2.54 (t, *J* 6.9 Hz, 2H, CH_2), 2.08 (t, *J* 7.6 Hz, 2H, CH_2), 1.73 (quint, *J* 6.8 Hz, 2H, CH_2), 1.59 – 1.48 (m, 4H, CH_2), 1.34 – 1.19 (m, 10H, CH_2); **^{13}C-NMR** (126 MHz, $CDCl_3$, MeOD): δ_C 203.1 (R_2CO), 174.0 (RCONH), 168.0 (RCOOR), 52.5 (OCH_3), 51.5 (CH_2N_3), 48.8 (CH_2), 40.4 (CH_2), 38.5 (CH_2), 36.7 (CH_2), 29.3 (CH_2), 29.3 (CH_2), 29.3 (CH_2), 29.1 (CH_2), 28.9 (CH_2), 26.7 (CH_2), 25.8 (CH_2), 23.2 (CH_2); **HR-ESI-MS**: $[M+Na]^+$ calculated: 377.2159, found: 377.2156.

2.6 Synthesis of probes 11a and 11b

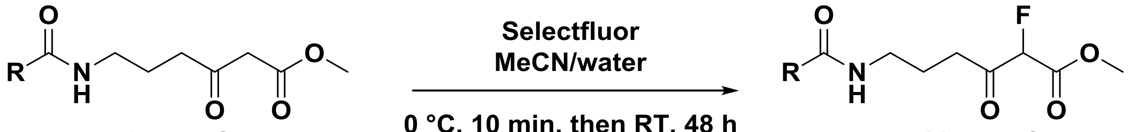

3a: R= CH_3
3b: R= CD_3

11a: R= CH_3
11b: R= CD_3

Methyl 6-acetamido-2-fluoro-3-oxohexanoate (11a)

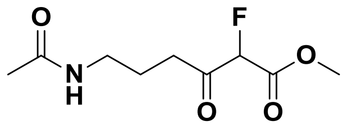

Methyl 6-acetamido-3-oxohexanoate (**3a**)[1] (257 mg, 1.27 mmol) was dissolved in a 1:1 mixture of acetonitrile and water (6 mL) and Selectfluor (540 mg, 1.52 mmol) was added portionwise at 0 °C. The reaction was stirred at room temperature for 48 h. The reaction was quenched by addition of water (6 mL) and then extracted with dichloromethane (2 x 20 mL). The organic layer was dried over $MgSO_4$, filtered and evaporated under reduced pressure. The crude material was purified by preparative HPLC (elution gradient starting from 100% H_2O and linearly increasing to 40% MeCN over 30 minutes); the desired product **11a** was yielded as a white powder (84 mg, 30%). R_t = 10.1 min.

^{1}H-NMR (400 MHz, CDCl3): δ_H 5.56 (br s, 1H, NH), 5.19 (d, *J* 46.4 Hz, 1H, HCF), 3.79 (s, 3H, OCH_3), 3.14 (apt q, *J* 6.8 Hz, 2H, CH_2), 2.75-2.59 (m, 2H, CH_2), 1.89 (s, 3H, CH_3), 1.78 (quint, *J* 6.8 Hz, 2H, CH_2); **^{13}C-NMR** (101 MHz, $CDCl_3$): δ_C 201.1 (R_2CO), 170.5 (RCONH), 164.5 (RCOOR), 92.2-90.21 (HCF *J* 197.33Hz), 53.3 (OCH_3), 38.6 (CH_2), 35.8 (CH_3), 22.7 (CH_2), 23.2 (CH_3); **HR-ESI-MS**: $[M+Na]^+$ calculated: 242.0799, found: 242.0796.

Methyl 6-(d_3-acetamido)-2-fluoro-3-oxohexanoate (11b)

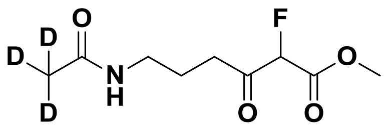

This compound was prepared from methyl 6-(d_3-acetamido)-3-oxohexanoate (**3b**)[2] according to the procedure above reported.

^{1}H-NMR (400 MHz, $CDCl_3$): δ_H 5.56 (br s, 1H, NH), 5.18 (d, *J* 46.4 Hz, 1H, HCF), 3.81 (s, 3H, OCH_3), 3.19 (apt q, *J* 6.78 Hz, 2H, CH_2), 2.75-2.55 (m, 2H, CH_2), 1.76 (quint, *J* 6.8 Hz, 2H, CH_2); **HR-ESI-MS**: $[M+H]^+$ calculated: 245.0987, found: 245.0990.

2.7 Synthesis of *tert*-butyl 5-azidopentylcarbamate (44)

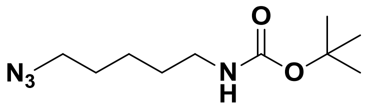

Sodium azide (2.03 g, 31.2 mmol) was dissolved in a solvent mixture of water (9 mL) and dichloromethane (18 mL).[7] Trifluoromethansulphonic anhydride (1.05 mL, 6.24 mmol) was added to the mixture at 0 °C and stirred at room temperature for 2 hours. The aqueous layer was separated and extracted with dichloromethane (2 x 5 mL). The pooled organic layers were washed with brine (1 x 5 mL) and then the trifluoromethansulphonic azide solution was added to a solution of *tert*-butyl (5-aminopentyl)carbamate (0.65 mL, 3.12 mmol), potassium carbonate (0.98 g, 7.10 mmol) and copper sulphate pentahydrate (5 mg) in methanol (22.5 mL) and water (15 mL). The reaction mixture was stirred overnight. The organic layer was washed with water (2 x 5 mL) and the aqueous layers were extracted with dichloromethane (2 x 10 mL). The pooled organic layers were dried over Na_2SO_4, filtered and the solvent was removed *in vacuo.* **44** was yielded as colorless oil (0.69 g, 91%). Data correspond to literature.[8]

^{1}H-NMR (400 MHz, $CDCl_3$): δ_H 4.55 (br s, 1H, NH), 3.25 (t, *J* 7.2 Hz, 2H, CH_2), 3.11 (td, *J* 6.8 Hz, 7.2 Hz, 2H, CH_2), 1.60 (tt, *J* 7.2 Hz, 7.2 Hz, 2H, CH_2), 1.47 (tt, *J* 7.2 Hz, 7.2 Hz, 2H, CH_2), 1.42 (9H, s, $C(CH_3)_3$), 1.37 (tt, *J* 7.2, 7.2 Hz, 2H, CH_2); **^{13}C-NMR** (101 MHz, $CDCl_3$): δ_C 156.0 (RCONH), 77.4 ($RC(CH_3)_3$), 51.3 (N_3CH_2R), 40.3 (CH_2NHR), 28.4 (CH_3), 30.0 (CH_2), 28.5 (CH_2), 23.9 (CH_2); **IR** (thin film): 3350 (N-H), 2935 (C-H), 2093 (N=N=N), 1688 (C=O), 1514, 1455, 1391, 1365, 1247, 1190, 1011, 867, 780 cm^{-1}; **HR-ESI-MS**: $[M+H]^+$ calculated: 229.1665 found: 229.1662.

2.8 Synthesis of 1,3,4,6-tetra-*O*-acetyl-*N*-4-pentynoylglucosamine (46)

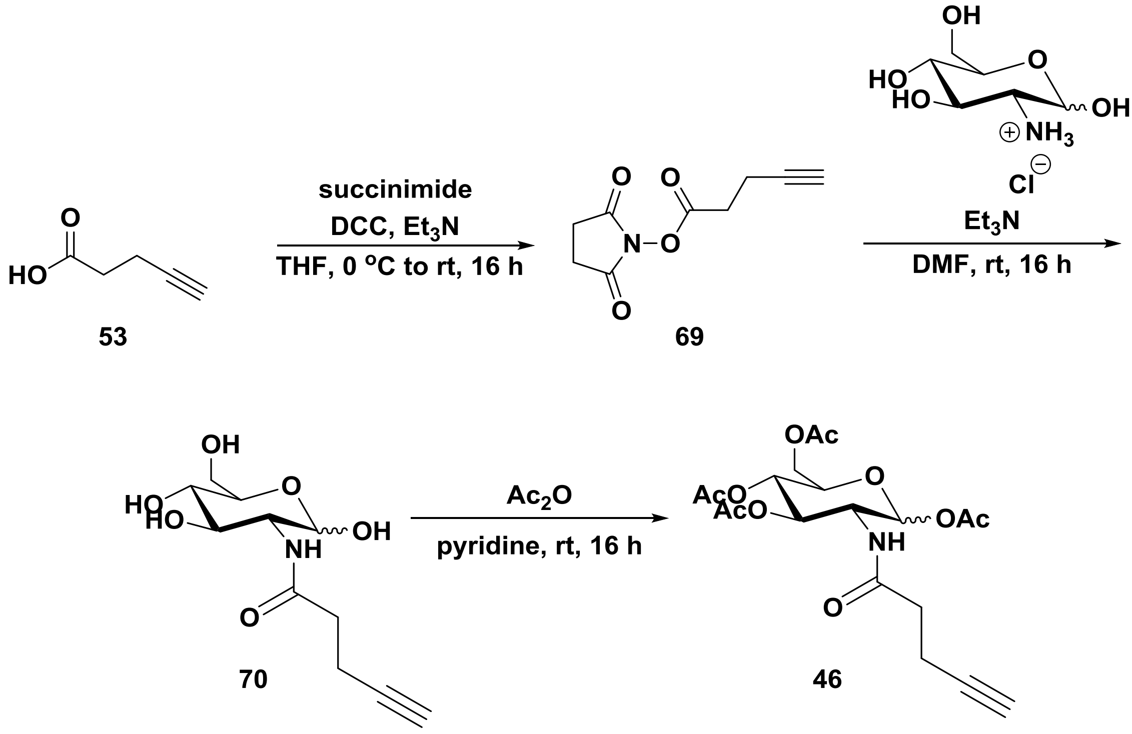

2.8.1 Synthesis of pent-4-ynoic acid 1-oxysuccinimidyl ester (69)

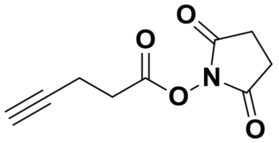

Pent-4-ynoic acid (**53**) (500 mg, 5.00 mmol) and *N*-hydroxysuccinimide (588 mg, 5.00 mmol) were dissolved in tetrahydrofuran (18 mL) and cooled to 0 °C.[9] *N,N'*-Dicyclohexylcarbodiimide (1.05 g, 5.00 mmol) in anhydrous tetrahydrofuran (5 mL) was added dropwise. The mixture was stirred for 45 minutes at 0 °C, slowly warmed to room temperature and stirred for additional 2 h. The *N,N'*-dicyclohexylcarbourea byproduct was filtered off and the solvent was evaporated under reduced pressure. The residue was dissolved in ethyl acetate, filtered again, washed with saturated aqueous $NaHCO_3$ and brine, and dried over $NaSO_4$. Filtration and evaporation of ethyl acetate afforded pent-4-ynoic acid 1-oxysuccinimidyl ester (**69**) as a white powder (883 mg, 89%). Data correspond to literature.[9]

^{1}H-NMR (400 MHz, $CDCl_3$): δ_H 2.86 (t, *J* 7.5 Hz, 2H, CH_2CO), 2.82 (br s, 4H, CH_2CONR_2), 2.59 (td, *J* 7.4, 2.5 Hz, 2H, CH_2), 2.05 (t, *J* 2.6 Hz, 1H, CH); **^{13}C-NMR** (101 MHz, $CDCl_3$): δ_C 169.0 (2 x RCONH), 167.0 (RCOOR), 80.8 (R*C*CH), 70.0 (RC*C*H), 30.2 (*C*H_2CO), 25.5 (2 x *C*H_2CO), 14.0 (CH_2); **HR-ESI-MS**: $[M+Na]^+$ calculated: 218.0424, found: 218.0423.

2.8.2 *N*-4-Pentynoylglucosamine (mixture of α/β anomers) (70)

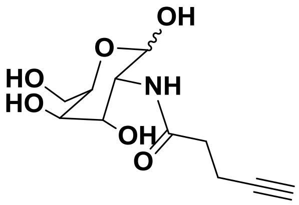

A mixture of D-glucosamine hydrochloride (276 mg, 1.28 mmol), pent-4-ynoic acid 1-oxysuccinimidyl ester (**69**) (250 mg, 1.28 mmol) and triethylamine (535 µL, 3.84 mmol) in *N*,*N*-dimethylformamide (15 mL) was stirred at room temperature for 18 h.[10] The mixture was concentrated *in vacuo* and the residue was purified by column chromatography (CH_3Cl : MeOH 8 : 1) to give **70** (mixture of anomers, α/ β 3:2) as a white solid (193 mg, 69%). Data correspond to literature.[11]

^{1}H-NMR (400 MHz, D_2O): δ_H 5.19 (d, *J* 3.6 Hz, 0.6H, CH), 4.70 (d, *J* 7.9 Hz, 0.4H, CH), 3.93 – 3.66 (m, 4H, CH), 3.57 – 3.30 (m, 2H, CHC*H*$_2$), 2.53 – 2.49 (m, 4H, CH_2), 2.37-2.23 (m, 1H, RCCH); **^{13}C-NMR** (75 MHz, D_2O): δ_C 175.2 (RCONH), 175.0 (RCONH), 94.9 (C1), 90.9 (C1), 83.4 (R*C*CH), 83.2 (R*C*CH), 75.9 (C5), 73.7 (C5), 71.5 (C5), 70.5 (C4), 70.1 (C4), 69.8 (RC*C*H), 60.7 (C6), 60.5 (C6), 56.6 (C2), 54.0 (C2), 34.6 (CH_2), 34.3 (CH_2), 14.4 (CH_2), 14.4 (CH_2); **HR-ESI-MS**: $[M+Na]^+$ calculated: 282.0948, found: 282.0949.

2.8.3 Synthesis of 1,3,4,6-tetra-*O*-acetyl-*N*-4-pentynoylglucosamine (α/β anomers) (46)

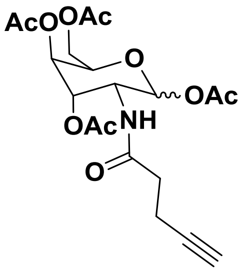

Acetic anhydride (234 µL, 2.45 mmol) was added to a solution of **70** (121 mg, 0.51 mmol) in pyridine. The reaction mixture was stirred at room temperature overnight. The solvent was evaporated under reduced pressure and the residue dissolved in dichloromethane (5 mL). The solution was washed with water (2x 5 mL), dried over $MgSO_4$, filtered and concentrated *in vacuo*.

The resultant residue was purified by flash column chromatography (gradient of 1 : 4 EtOAc : hexane to 1 :1 EtOAc : hexane) to give **46** (mixture of anomers, α : β 4 : 1) as a white solid (150 mg, 68%).[10]

^{1}H-NMR (400 MHz, $CDCl_3$): δ_H δ 6.15 (d, *J* 3.6 Hz, 0.8H, CH), 5.94 (d, *J* 9.5 Hz, 0.2H, NH), 5.82 (d, *J* 9.0 Hz, 0.8H, NH), 5.67 (d, *J* 8.8 Hz, 0.2H, CH), 5.31 – 5.00 (m, 2H, CH), 4.47 (ddd, *J* 10.4, 9.6, 3.5 Hz, 0.8H, CH), 4.37 – 4.24 (m, 0.2H, CH), 4.22 (dd, *J* 12.4, 4.0 Hz, 0.8H, CH), 4.13 – 3.92 (m, 2H, CH), 3.79 (ddd, *J* 9.7, 4.4, 2.1 Hz, 0.2H, CH), 2.50 – 2.40 (m, 2H, CH_2), 2.36 – 2.27 (m, 2H, CH_2), 2.15 (s, 2.4H, CH_3), 2.07 (s, 0.6H, CH_3), 2.05 (s, 2.4H, CH_3), 2.01 (s, 2.4H, CH_3), 2.00 (s, 3H, CH_3), 2.00 (s, 1.2H, CH_3), 1.96–1.92 (m, 1H, RCCH);**^{13}C-NMR** (75 MHz, $CDCl_3$): δ_C 171.7 (RCONH), 171.2 (RCOOR), 171.1 (RCOOR), 170.8 (RCOOR), 169.6 (RCOOR), 169.4 (RCOOR), 169.2 (RCOOR), 168.7 (RCOOR), 92.5 (C1), 90.7 (CH), 82.6 (R*C*CH), 72.8 (CH), 72.5 (CH), 70.6 (CH), 69.8 (CH), 69.7 (RC*C*H), 68.0 (CH), 67.6 (CH), 61.7 (CH), 61.6 (CH), 52.8 (CH), 51.0 (CH), 35.4 (CH_2), 35.2 (CH_2), 21.0 (Ac), 20.9 (Ac), 20.8 (Ac), 20. (Ac), 14.9 (CH_2), 14.8 (CH_2); **HR-ESI-MS**: $[M+Na]^+$ calculated: 450.1371, found: 450.1361.

3 Feeding experiments

3.1 Microbiology methods

All media and glassware were sterilized prior to use by autoclave (Astell). Liquid cultures were grown with shaking in Innova 44 incubator/shaker (New Brunswick scientific).

<u>M79 medium</u>: 2.5 g glucose, 2.5 g peptone, 0.5 g yeast extract, 1.5 g NaCl, 2.5 g casein hydrolysate in 250 ml of tap water adjusted to pH 7.1.

<u>MYM medium</u>: 1.0 g maltose, 1.0 g yeast extract, 2.5 g malt extract in 250 ml of tap water adjusted to pH 7.1.

3.2 Construction of mutant strains of *Streptomyces lasaliensis*

The cultivation of the wild type lasalocid-producing strain *S. lasaliensis* NRRL3382 was carried out as previously described.[12] The construction of *S. lasaliensis* ACP12 (S970A) and ΔlasB ACP12 (S970A) mutant strains was previously reported.[12] The construction of *S. lasaliensis* ACP5 (S3799A) was similarly accomplished by site-directed mutagenesis utilising primer pairs O5_SDMF, oP5R, O5_SDMR and oP5L (Table 1S) by overlap extension PCR and cloning into pYH7 to give plasmid pA5M.[12] The ligated plasmid was transformed into *E. coli* strain DH10B and positive colonies were

tested by restriction mapping and sequencing before a correct clone was transferred to *E. coli* ET12567/pUZ8002. Conjugation was carried out with the *S. lasaliensis* wild type as previously described.[12] Candidate mutants were confirmed by sequencing of the mutant region utilising oligonucleotides oA5SF and oA5SR (Table 1S). The mutant was fermented and LC-MS analysis of the ethyl acetate extracts of each culture showed that lasalocid production was completely abolished.

Table 1S: Primers utilised for the construction and the sequencing of *S. lasaliensis* ACP5 (S3799A).

O5_SDMF	GACCTCGGCTTCGACGCCCTCACCAGCGTCG
O5_SDMR	CGACGCTGGTGAGGGCGTCGAAGCCGAGGTC
oP5L	CCCCGAGTCCATATGTCCGTT
oP5R	GCATGGTGGAAGCTTCGTCCA
oA5SF	TCGGATGCCGTGCTGGT
oA5SR	GATGGCGAGGGGTTCGTC

3.3 Growth of *S. lasaliensis* wild-type (WT), ACP12 (S970A), ACP5 (S3799A) and ΔlasB-ACP12 (S970A) strains and mass spectrometry analysis

All the *S. lasaliensis* strains were grown in M79 medium (10 mL) for 3 days at 30 ºC in 50 mL Erlenmeyer flasks with spring. Seed cultures (100 µL) were used to inoculate MYM liquid cultures (10 mL, in duplicate/triplicate copy, in 50 mL Erlenmeyer flasks with spring). They were incubated at 30°C for 5 days. After the first day of incubation, the different probes (**3-13**, final concentration: 2.5 mM)[1] were added portionwise as follows: addition of 8.3 µmol dissolved in 100 µl of MeOH on day 2 and day 3; addition of 4.1 µmol dissolved in 50 µl of MeOH on day 4 and 5. Control liquid cultures in absence of **3-13** of the strains were also prepared (in duplicate/triplicate copy). After 5 days of fermentation, the liquid cultures were extracted with ethyl acetate (20 mL x 2). The extracts were concentrated and the residues were redissolved in HPLC-grade methanol (1 mL) for mass spectrometry analysis.

Unless otherwise stated, UPLC-HR-ESI-MS analyses of *S. lasaliensis* ACP12 (S970A), ACP5 (S3799A) and ΔlasB-ACP12 (S970A) strains were performed on a MaXis Impact UHR-TOF (Bruker Daltonics).

[1] General protocol unless otherwise specified.

Samples (5 μl) were injected onto an Acquity UPLC HSS T3 (150 mm x 1.0 mm, 1.8 μm) or Agilent Eclipse C18 (1.8um, 100 mm x 2.1 mm). The mobile phase consisted of a gradient of water and acetonitrile (HPLC grade, each with 0.1 % trifluoroacetic acid). The following solvent (A =1% TFA in H_2O, B =1% TFA in MeCN) gradients were applied:

Method 1 (for feeding with probes **4**-**6**, **9**, **11a**, **11b** and analysis of compounds **45** and **47**): 10% B 0-2.7 min; 10-100% B 2.7-42.7 min; 100% B 42.7-52.7 min; 100-10% B 52.7-55.7 min; 10% B 55.7-67.7 min, using an Acquity UPLC HSS T3 column at a flow rate of 0.05 mL/min.

Method 2 (for feeding with probes **7**, **8**, **13**): 10% B 0-2.7 min; 10-100% B 2.7-42.7 min; 100% B 42.7-62.7 min; 100-10% B 62.7-65.7 min; 10% B 65.7-77.7 min, using an Acquity UPLC HSS T3 column at a flow rate of 0.05 mL/min.

Method 3 (for analysis of compounds **48** and **71**): 5% B 0-5.3 min; 5-100% B 5.3-17.3 min; 100% B 17.3-22.3 min; 100-5% B 22.3-25.3 min; 5% B 25.3-35.3 min, using Agilent Eclipse C18 at a flow rate of 0.2 mL/min.

Spectra were recorded in positive ionisation mode, scanning from *m/z* 100 to 3000, with the resolution set at 45K. Selected ion search within 5 ppm was performed, as well as high resolution fragmentation (collision energy set to 15-20%) for the putative biosynthetic intermediates. Off-loading of the putative polyethers **2a**-**b** from *S. lasaliensis* ACP12 (S970A) *via* **1a** and **1b** has been previously reported.[12]

3.4 Off-loading of functionalized polyketides from *S. lasaliensis* ACP12 (S970A) and ΔlasB-ACP12 (S970A) via chemical probes 4-8

3.4.1 LC-HRMS and MS/MS analysis of off-loading of 24, generated from *S. lasaliensis* ACP12 (S970A) and probe 4

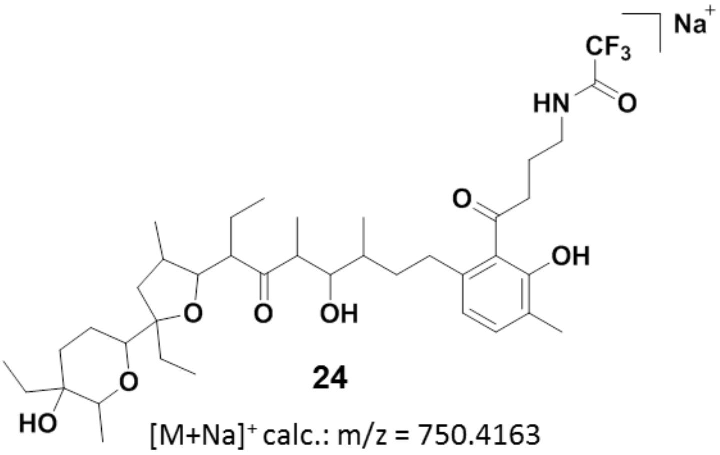

24

$[M+Na]^+$ calc.: m/z = 750.4163

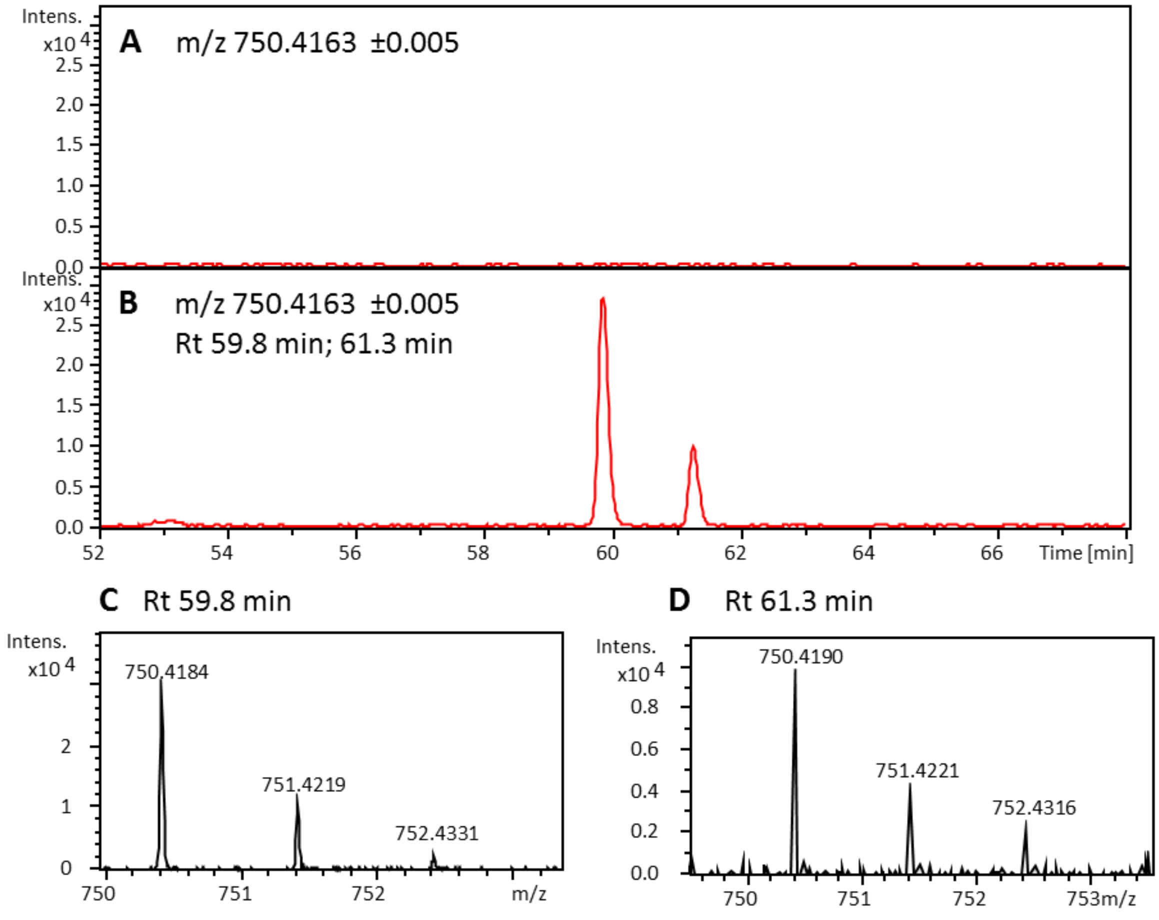

Figure 1: LC-ESI-HRMS analysis of the organic extracts of *S. lasaliensis* ACP12 (S970A) grown in the absence (**A**) and in the presence (**B**) of probe **4** (final concentration 3.0 mM; day 2 and 3: 0.01 mmol, day 4 and 5:

0.005 mmol): $[M+Na]^+$ extracted ion trace for the off-loaded polyketide **24** is shown (**B**). Its stereochemistry is yet to be separately determined; further work is in progress to establish this, as well as the origin of two peaks for **24** (possibly arising from the presence of isomers/conformers). These species have also been detected as ammonium adduct (data not shown) and were absent in all the control samples (e.g. in absence of the probe, **A**, and in extracts obtained from the use of different probes). The high resolution masses and isotopic abundances for **24** are shown (**C** and **D**).

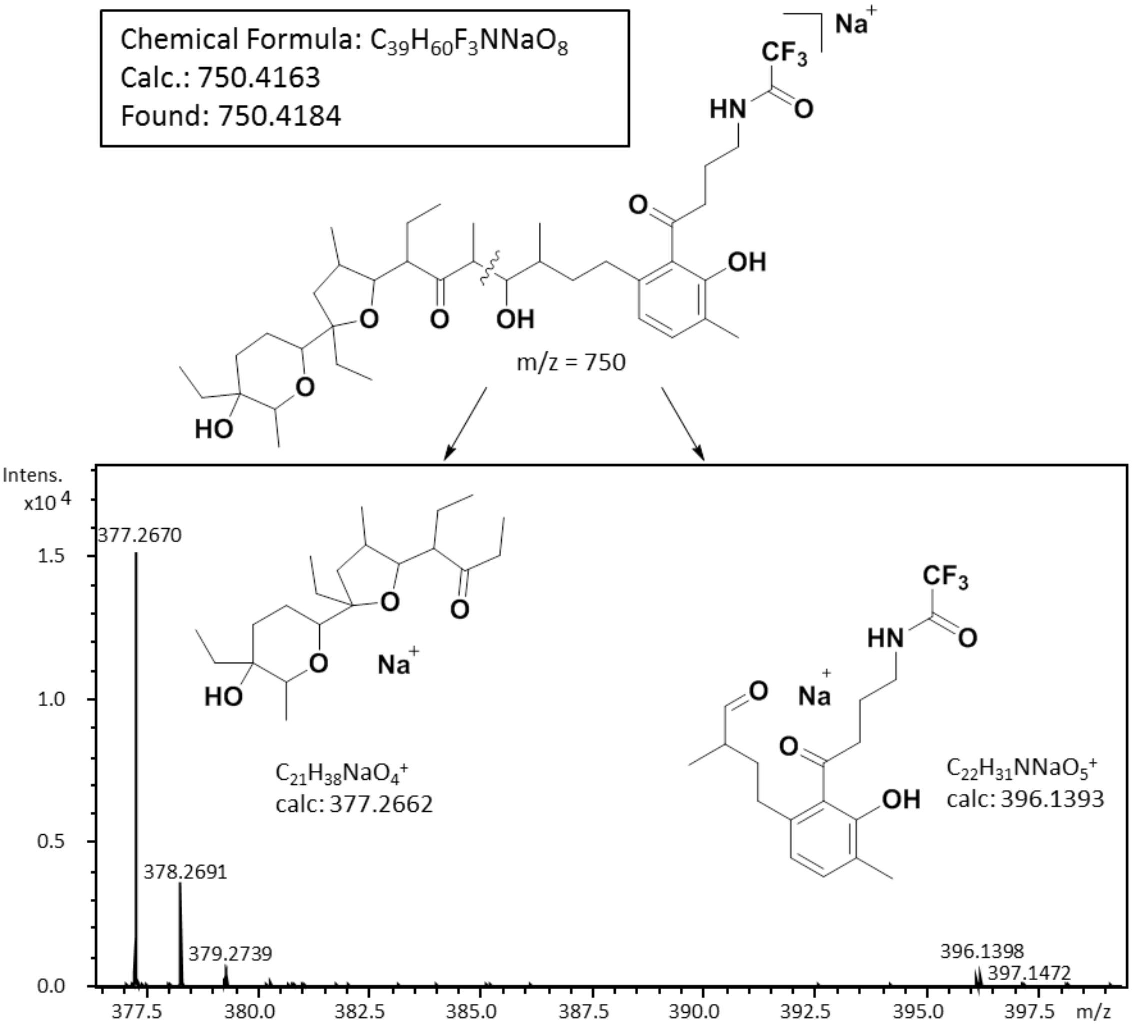

Figure 2: HR-ESI-MS[2] analysis of the off-loaded dodecaketide **24**. McLafferty fragmentation of the $[M+Na]^+$ adduct generates a left–hand fragment of *m/z* 377, characteristic of lasalocid A, as well as a right-hand fragment containing the fluorinated label (*m/z* 396).

3.4.2 LC-HRMS and MS/MS analysis of 25, generated from *S. lasaliensis* ACP12 (S970A) and probe 5

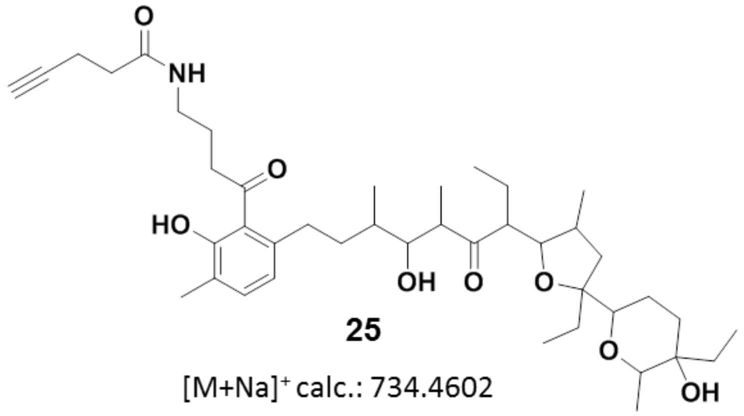

25

$[M+Na]^+$ calc.: 734.4602

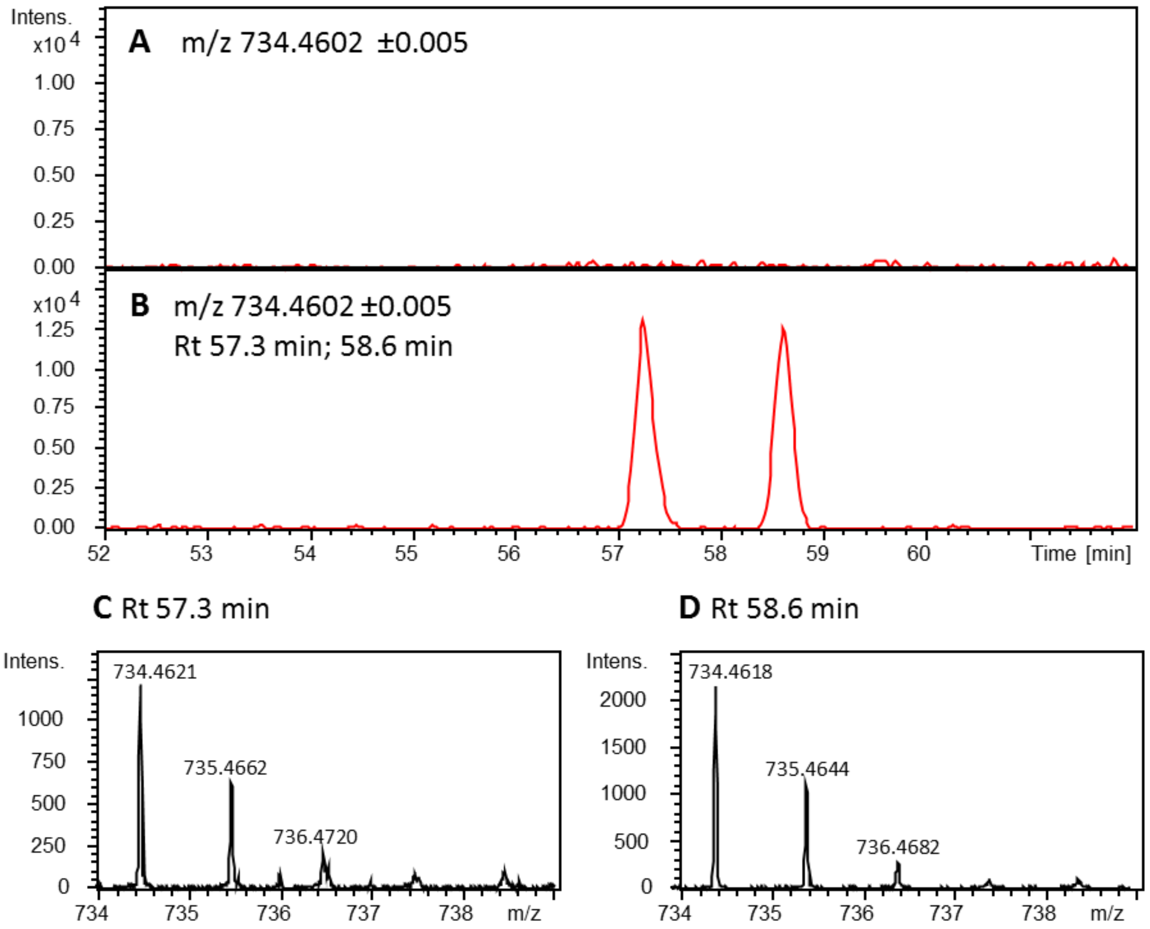

Figure 3: LC-ESI-HRMS analysis of the organic extracts of *S. lasaliensis* ACP12 (S970A) grown in the absence (**A**) and in the presence (**B**) of probe **5** (2.5 mM final concentration): $[M+Na]^+$ extracted ion trace for the off-loaded polyketide **25** is shown (**B**). Its stereochemistry is yet to be separately determined; further work is in progress to establish this, as well as the origin of two peaks for **25** (possibly arising from the presence of isomers/conformers). These species have also been detected as ammonium adducts (data not shown) and were absent in all the control samples (e.g. in the absence of the probe, **A,** and in extracts obtained

from the use of different probes). The high resolution masses and isotopic abundances for **25** are shown (**C** and **D**).

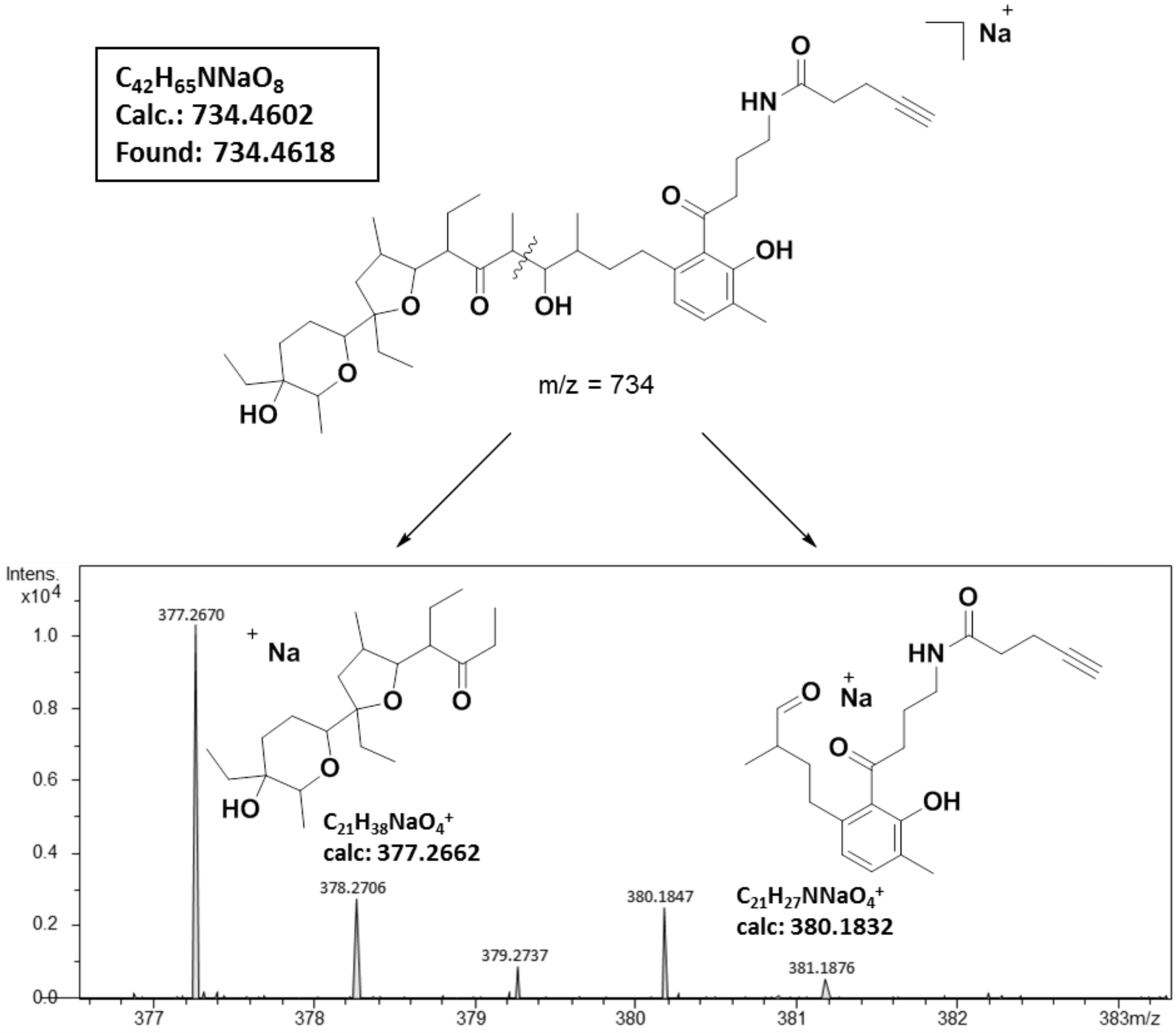

Figure 4: HR-ESI-MS[2] analysis of the dodecaketide **25**. McLafferty fragmentation of the $[M+Na]^+$ adduct generates a left–hand fragment of *m/z* 377, characteristic of lasalocid A, as well as a right-hand fragment containing the alkyne handle (*m/z* 380).

3.4.3 3.3.2 HPLC isolation of 25

Crude extracts containing **25** were purified by semipreparative HPLC (Fig. 3) for further derivatisation by copper-catalyzed azide-alkyne cycloaddition (CuAAC, Figure 33). Isolation was performed on a semi preparative Phenomenex Synergi polar RP column (250 mm x 10.0 mm, 4 µm); a mixture of water and acetonitrile (containing 0.1% TFA) was used with a flow rate of 2.5 mL/min and an elution gradient starting from 100% water and linearly increasing to 100 %

acetonitrile over 45 min, with UV detection set at 254, 280 and 210 nm. The two peaks for **25** were isolated with a Rt of 57.3 and 58.6 min, respectively.

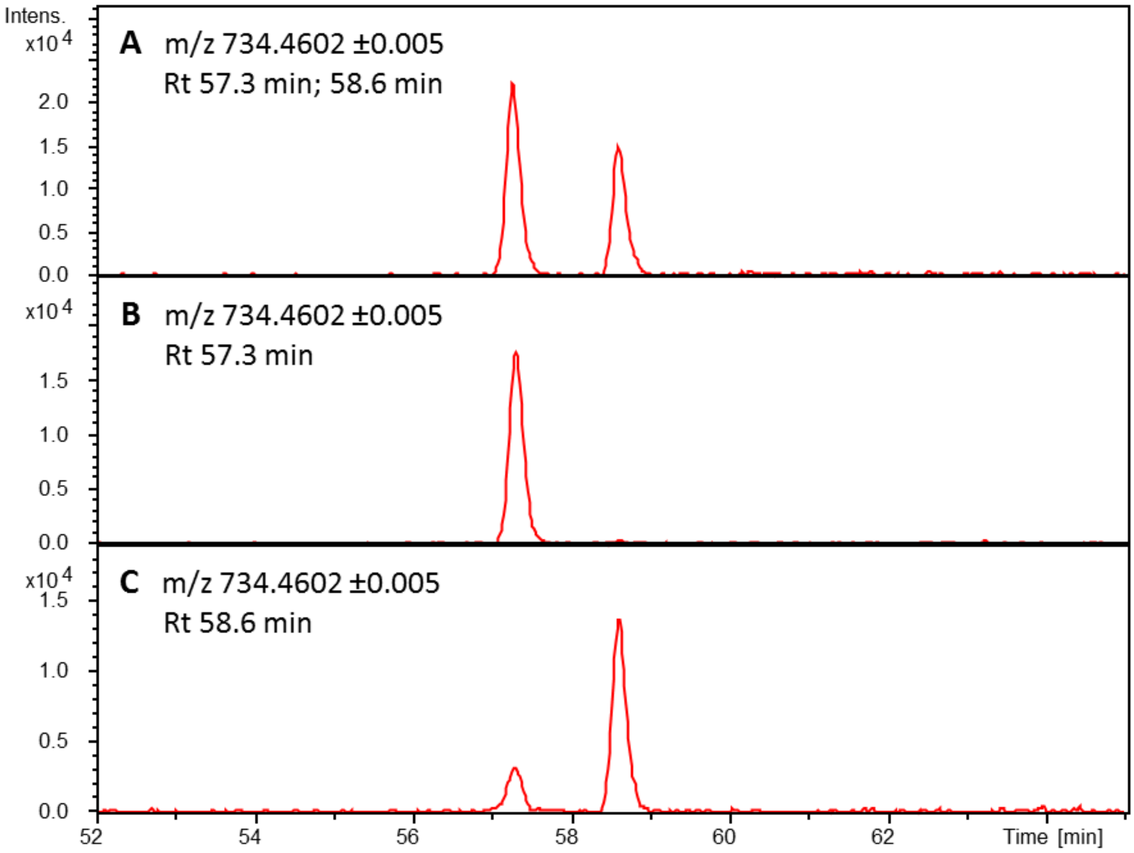

Figure 5: LC-ESI-HRMS analysis of the crude (**A**) and HPLC isolated organic extracts (**B** and **C**) of *S. lasaliensis* ACP12 (S970A) grown in the presence of **5** (2.5 mM final concentration).

3.4.4 LC-HRMS and MS/MS analysis of probe 3 and its decarboxylated form from *S. lasaliensis* ACP12 (S970A) crude extracts

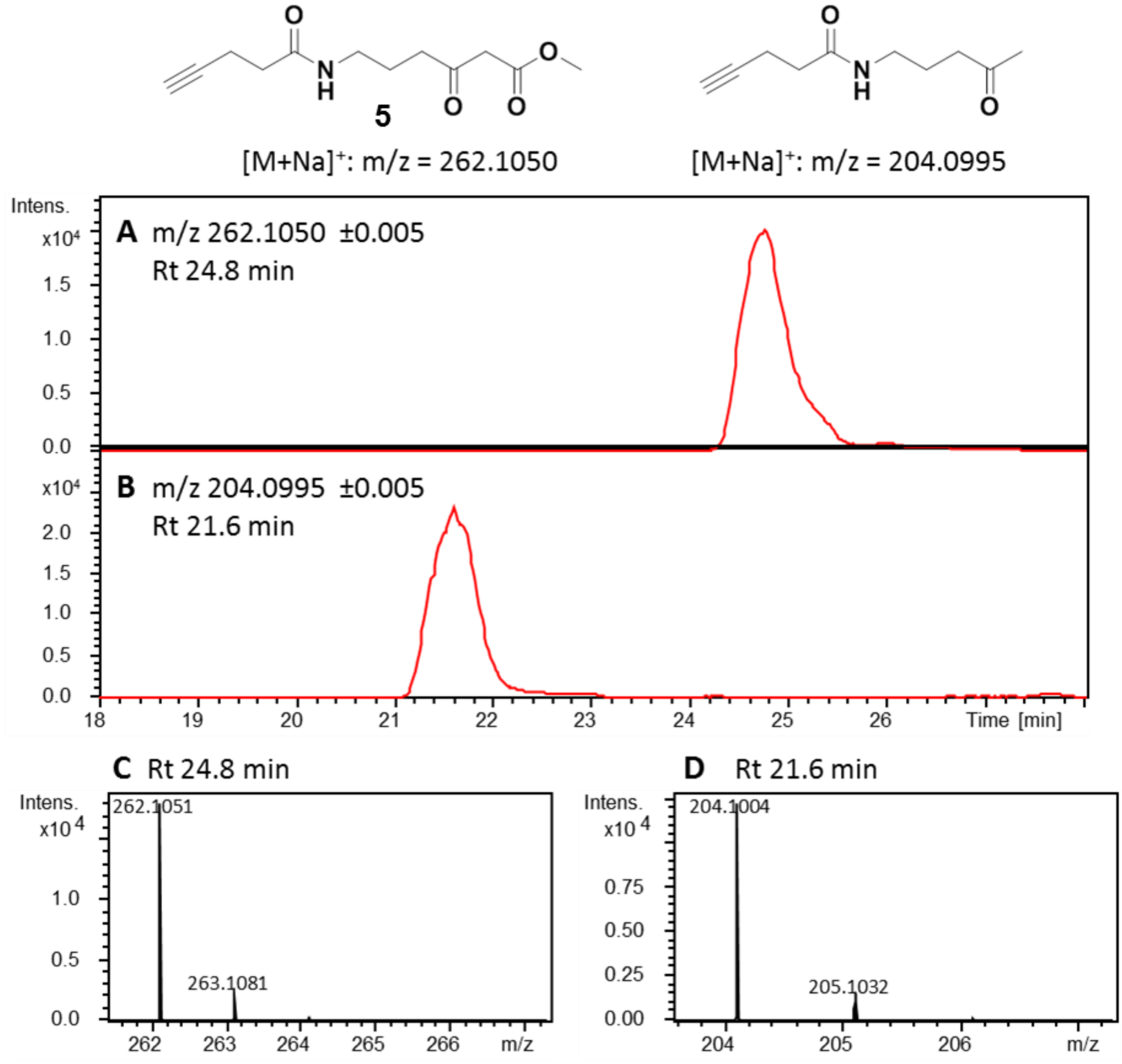

Figure 6: LC-ESI-HRMS analysis* of the probe **5** (**A**) and of the correspondent decarboxylated form following *in vivo* hydrolysis (**B**) in the crude extract of *S. lasaliensis* ACP12 (S970A) after 5 days. The high resolution masses and isotopic abundances for **3** and for the correspondent decarboxylated form are shown in **Figure C** and **Figure D** respectively.

*These data have been collected using a MaXis Bruker instrument.

3.4.5 LC-HRMS and MS/MS analysis of off-loading of 26, generated from *S. lasaliensis* ACP12 (S970A) and probe 6, and of 29, generated from *S. lasaliensis* ΔlasB-ACP12 (S970A) and probe 6

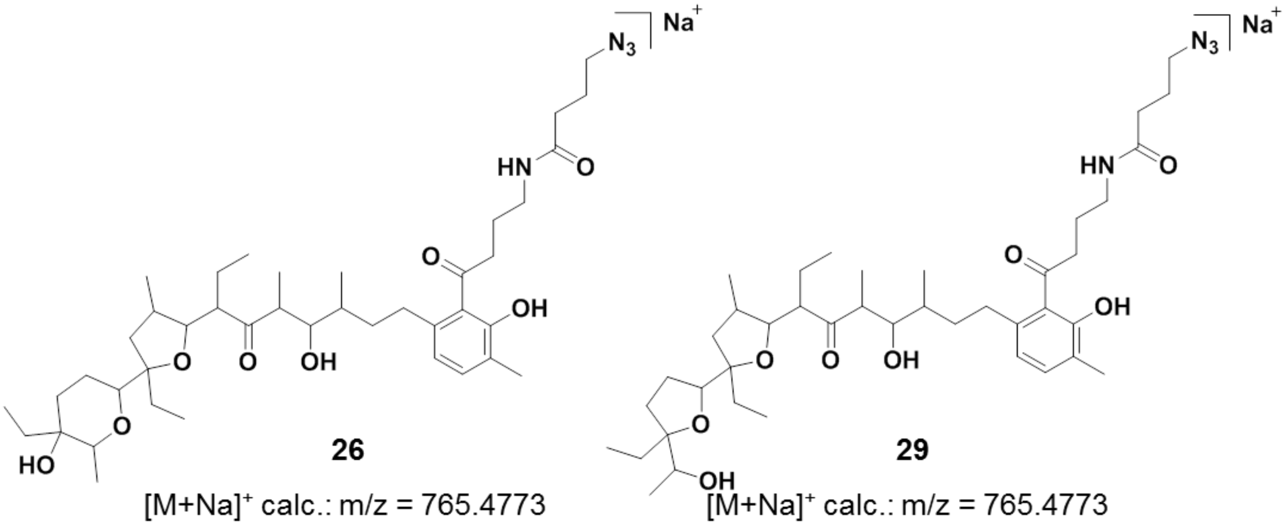

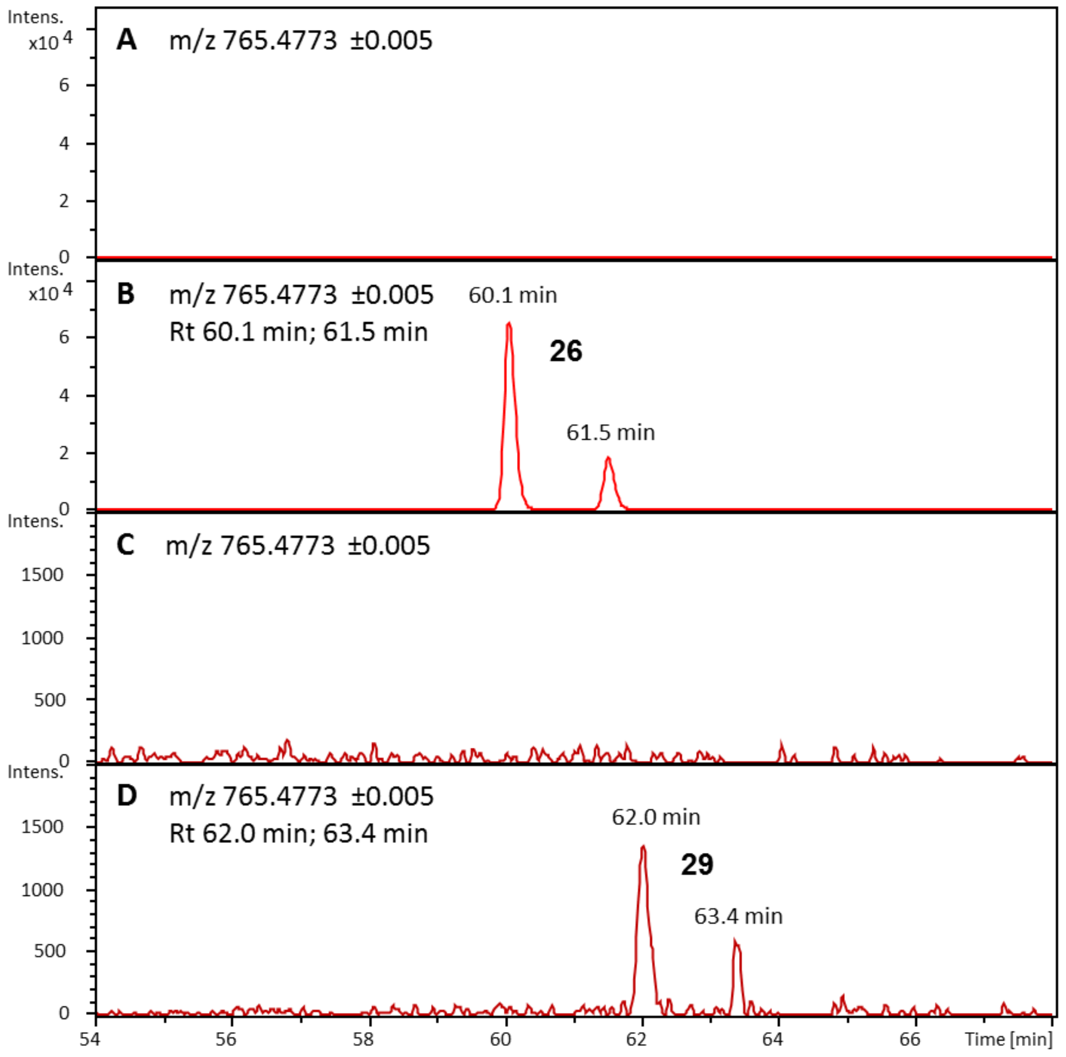

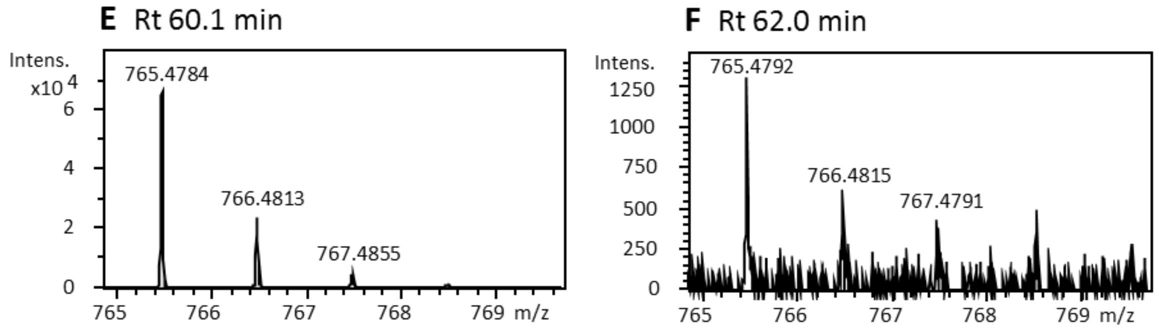

Figure 7: LC-ESI-HRMS analysis of the organic extracts of *S. lasaliensis* ACP12 (S970A) (**A** and **B**) and ΔlasB-ACP12 (S970A) (**C** and **D**) grown in the presence (**B** and **D**) and in absence (**A** and **C**) of probe **6** (final concentration 4.0 mM; 0.01 mmol each day): $[M+Na]^+$ extracted ion traces for the putative off-loaded polyketides **26** and **29** are shown (**B** and **D**, respectively). Their stereochemistry is yet to be separately determined; further work is in progress to establish this, as well as the origin of two peaks for **26** and **29** (possibly arising from the presence of isomers/conformers). These species have been also detected as ammonium adducts (data not shown) and were absent in all the control samples (e.g. in absence of the probe, **A** and **C**, respectively, and in extracts obtained from the use of different probes). The high resolution masses and isotopic abundances for **26** (**E**) and **29** are shown (**F**).

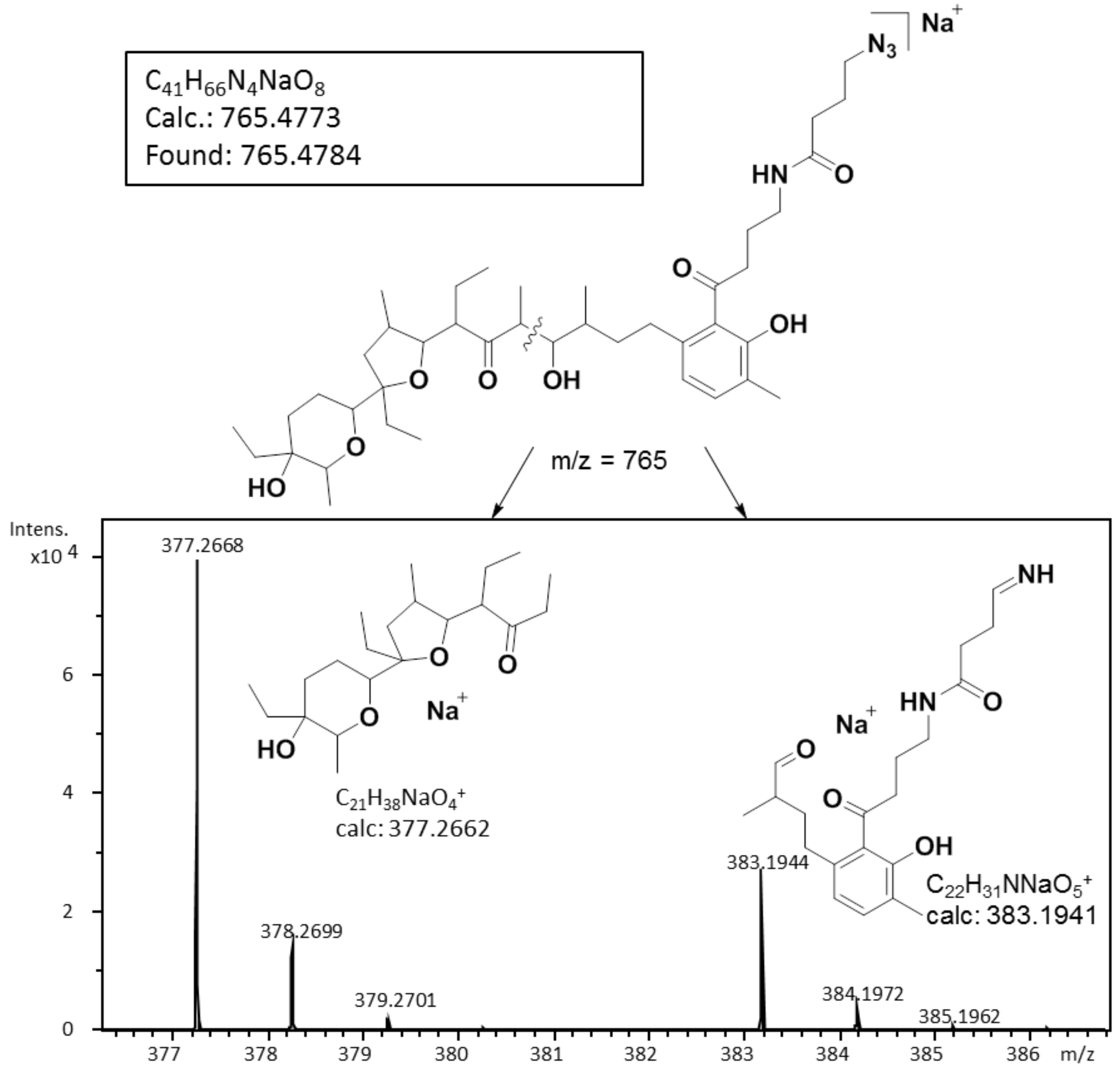

Figure 8: HR-ESI-MS^2 analysis of the off-loaded dodecaketide **26**. McLafferty fragmentation of the $[M+Na]^+$ adduct generates a left–hand fragment of *m/z* 377, characteristic of lasalocid A, as well as a right-hand fragment after spontaneous loss of N_2 (*m/z* 383).

3.4.6 LC-HRMS and MS/MS analysis of off-loading of 27 and 38, generated from *S. lasaliensis* ACP12 (S970A) and probe 7

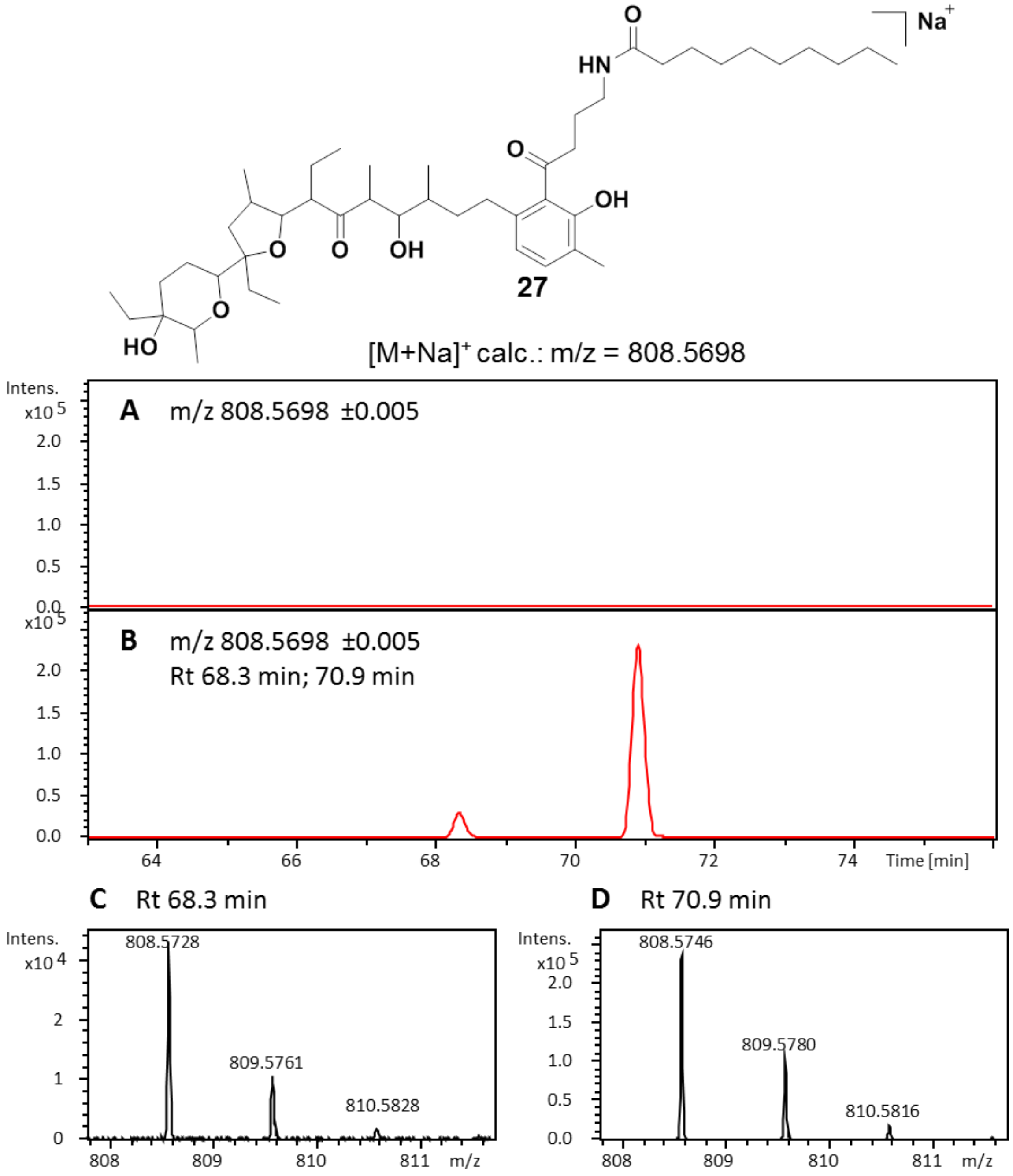

Figure 9: LC-ESI-HRMS analysis of the organic extracts of *S. lasaliensis* ACP12 (S970A) grown in the absence (**A**) and in the presence (**B**) of probe **7** (final concentration 3.0 mM; day 2 and 3: 0.01 mmol, day 4 and 5: 0.005 mmol): $[M+Na]^+$ extracted ion trace for the off-loaded polyketide **27** is shown (**B**). Its stereochemistry is yet to be separately determined; further work is in progress to establish this, as well as the origin of two peaks for **27** (possibly arising from the presence of isomers/conformers). These species have also been detected as ammonium adduct (data not shown) and were absent in all the control samples (e.g. in

absence of the probe, **A**, and in extracts obtained from the use of different probes). The high resolution masses and isotopic abundances for **27** are shown (**C** and **D**).

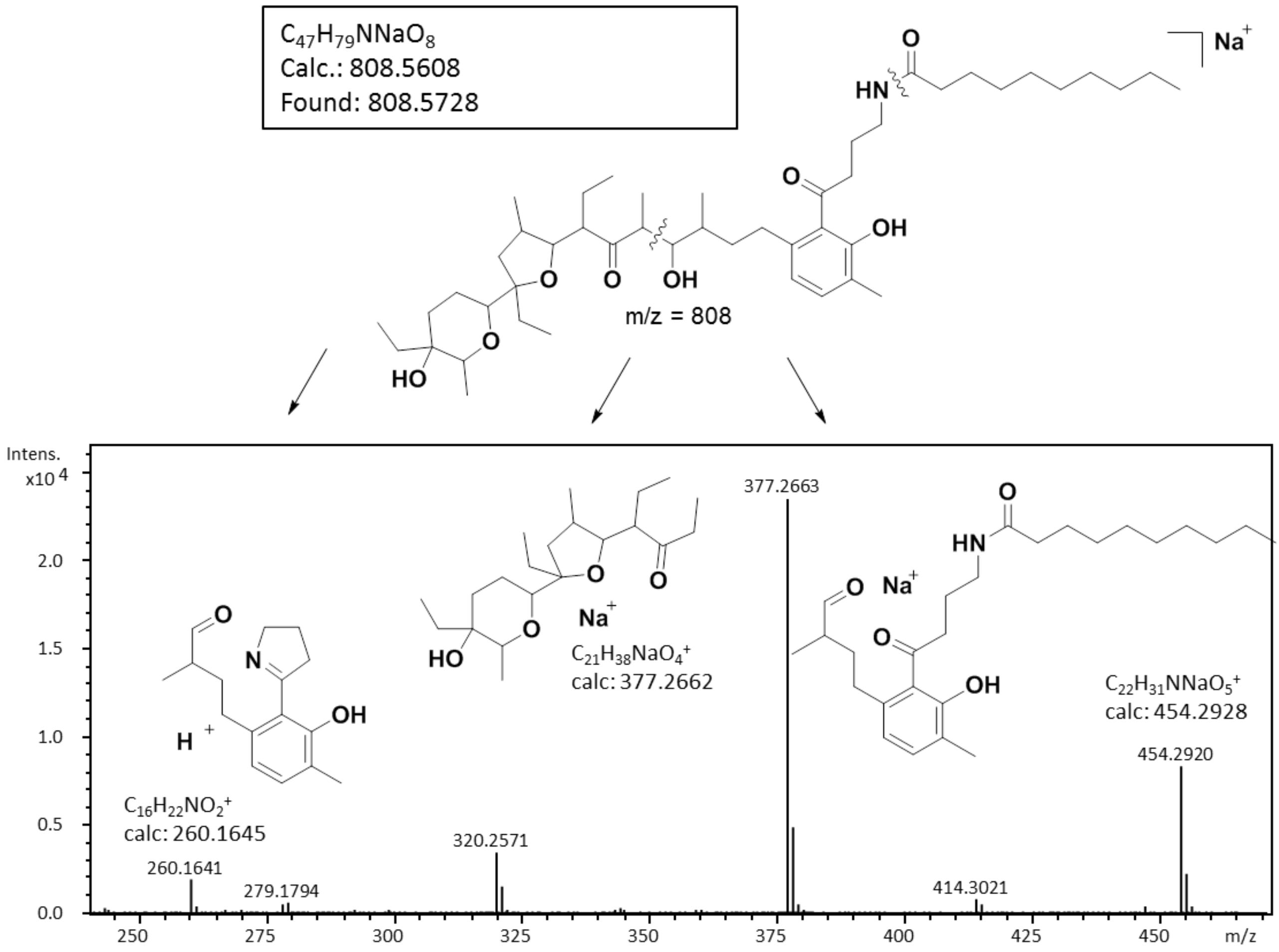

Figure 10: HR-ESI-MS^2 analysis of the off-loaded dodecaketide **27**. McLafferty fragmentation of the $[M+Na]^+$ adduct generates a left–hand fragment of *m/z* 377, characteristic of lasalocid A, as well as a right-hand fragment containing the long chain moiety (*m/z* 454). The distinctive peak at *m/z* 260 corresponds to the protonated imine generated by the cyclization of the aromatic ketone and the amine derived from loss of the decanoyl chain.

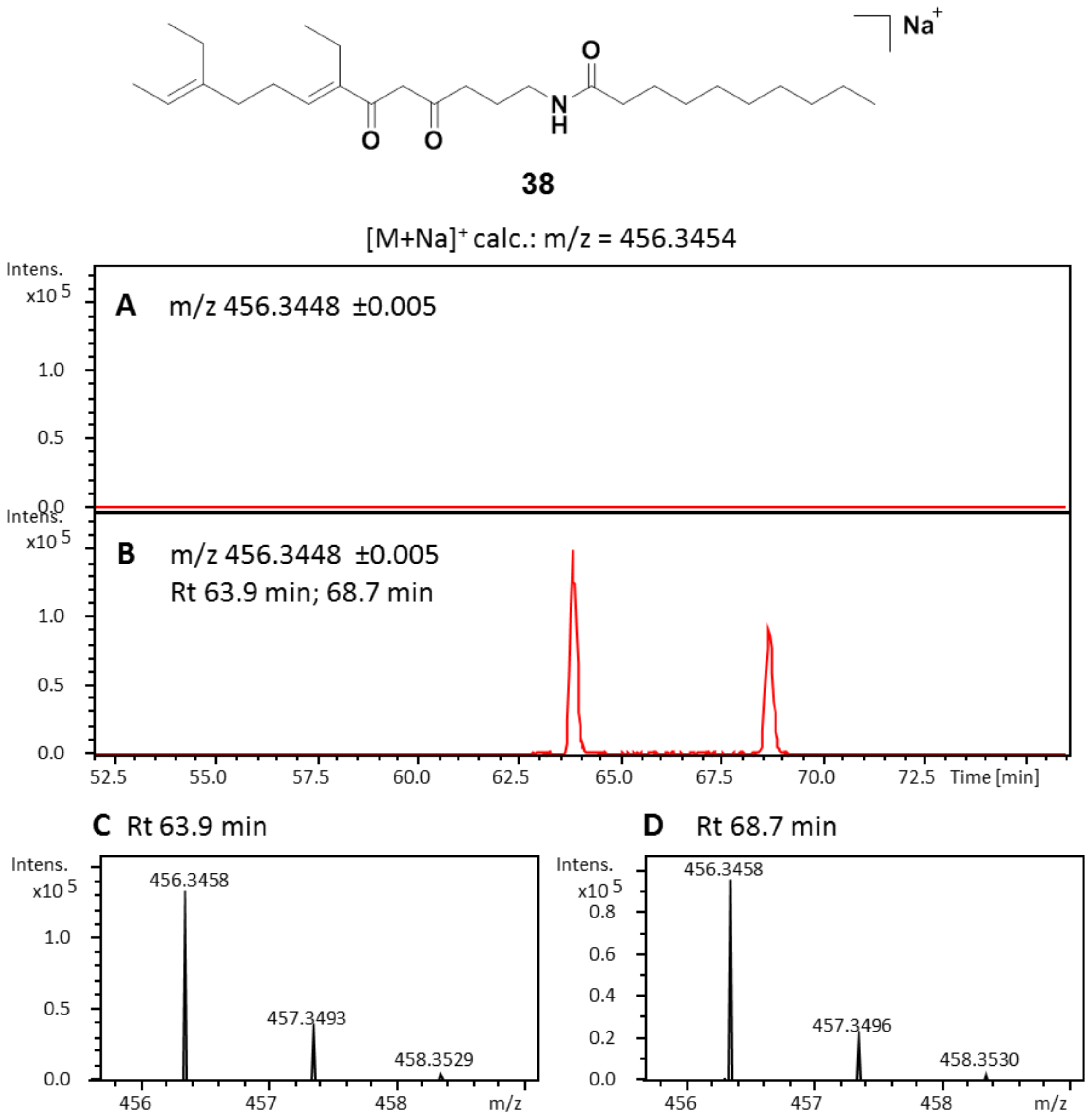

Figure 11: LC-ESI-HRMS analysis of the organic extracts of *S. lasaliensis* ACP12 (S970A) grown in the absence (**A**) and in the presence (**B**) of probe **7** (final concentration 3.0 mM; day 2 and 3: 0.01 mmol, day 4 and 5: 0.005 mmol): $[M+Na]^+$ extracted ion trace for the off-loaded polyketide **38** is shown (**B**). Further work is in progress to establish the origin of two peaks for **38** (possibly arising from the presence of isomers). These species were absent in all the control samples (e.g. in absence of the probe, **A**, and in extracts obtained from the use of different probes). The high resolution masses and isotopic abundances for **38** are shown (**C** and **D**).

3.4.7 LC-HRMS and MS/MS analysis of off-loading of 28 and 39, generated from *S. lasaliensis* ACP12 (S970A) and probe 8 and 30, generated from *S. lasaliensis* ΔlasB-ACP12 (S970A) and probe 8

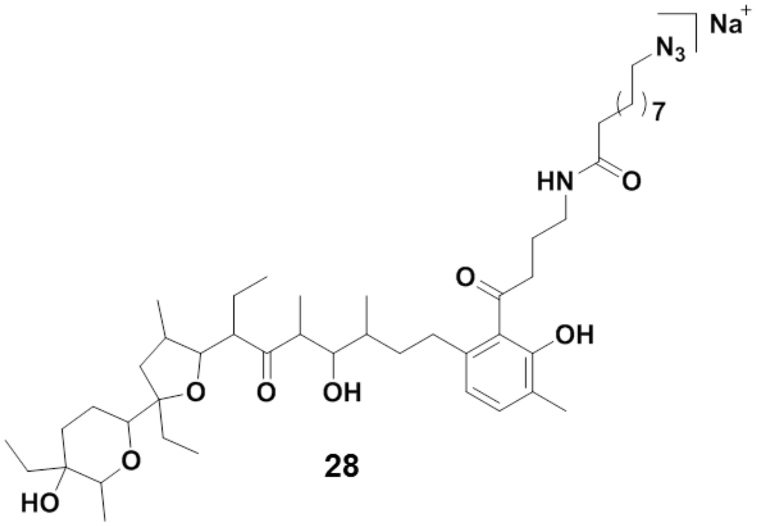

28

$[M+Na]^+$ calc.: m/z = 849.5712

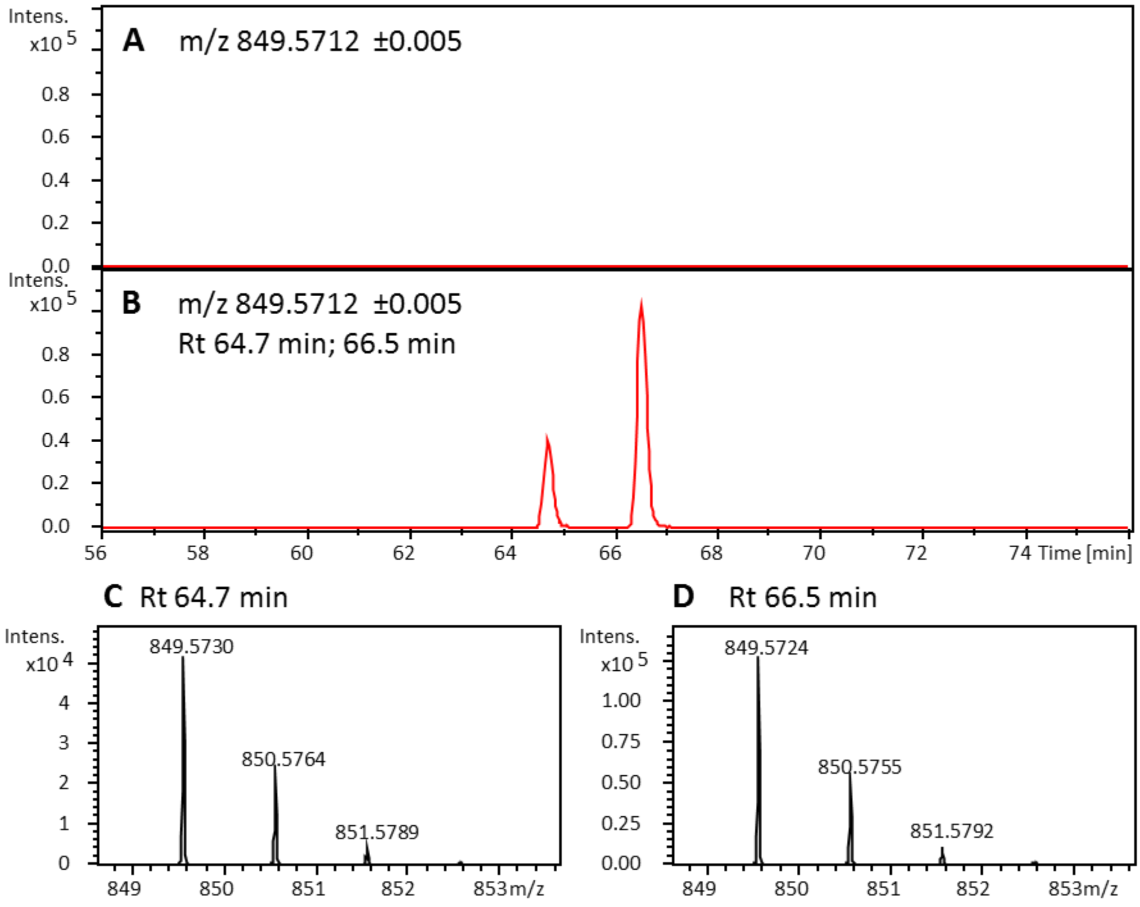

Figure 12: LC-ESI-HRMS analysis of the organic extracts of *S. lasaliensis* ACP12 (S970A) grown in the absence (**A**) and in the presence (**B**) of probe **8** (final concentration 4.0 mM; 0.1 mmol each day): $[M+Na]^+$

extracted ion trace for the off-loaded polyketide **28** is shown (**B**). Its stereochemistry is yet to be separately determined; further work is in progress to establish this, as well as the origin of two peaks for **28** (possibly arising from the presence of isomers/conformers). These species have also been detected as ammonium adduct (data not shown) and were absent in all the control samples (e.g. in absence of the probe, **A**, and in extracts obtained from the use of different probes). The high resolution masses and isotopic abundances for **28** are shown (**C** and **D**).

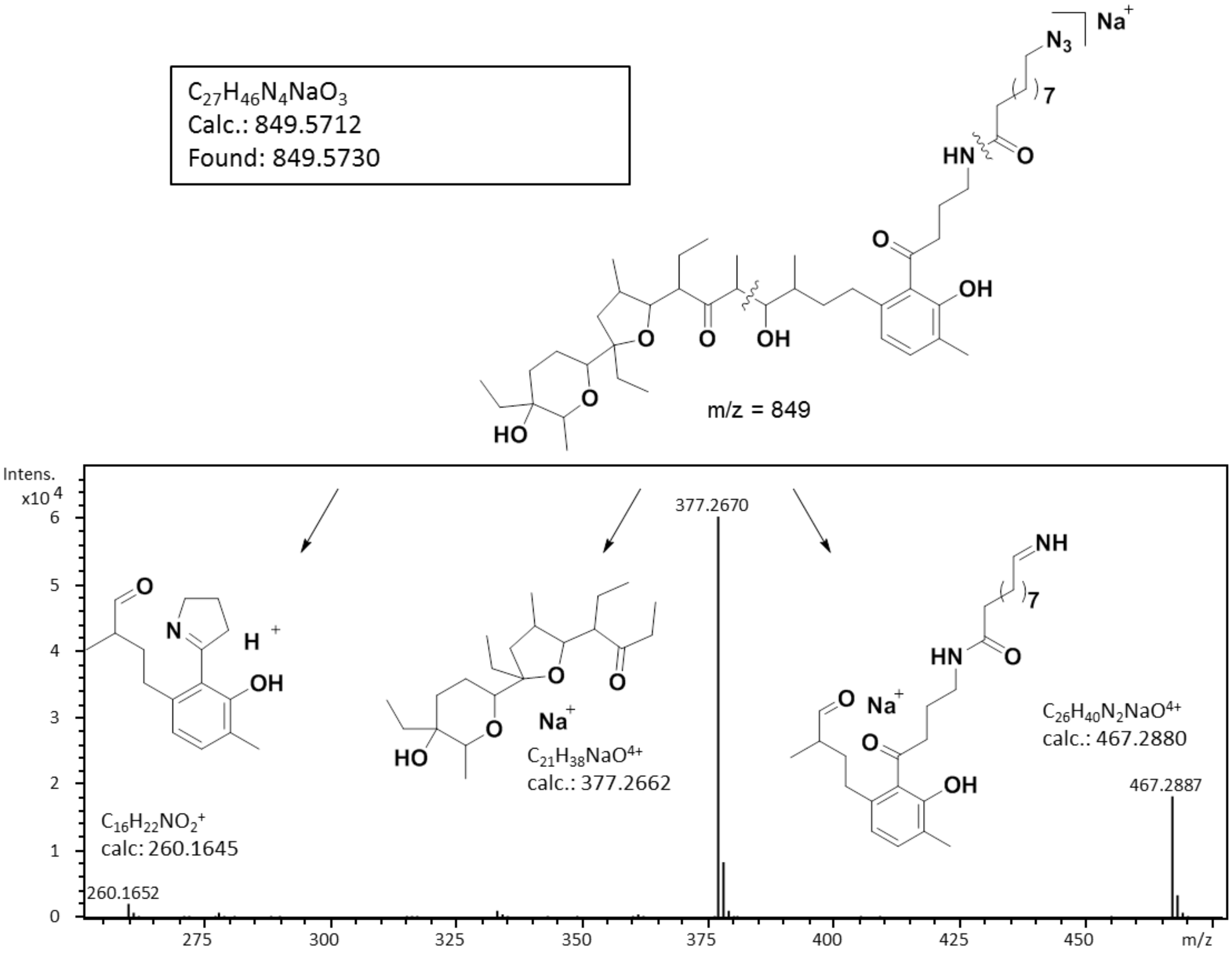

Figure 13: HR-ESI-MS[2] analysis of the off-loaded dodecaketide **28**. Fragmentation of the $[M+Na]^+$ adduct generates a left–hand fragment of *m/z* 377, characteristic of lasalocid A, as well as a right-hand fragment after spontaneous loss of N_2 (*m/z* 467). The distinctive peak at *m/z* 260 corresponds to the protonated imine generated by cyclization of the aromatic ketone and the amine resulting from loss of the decanoyl chain.

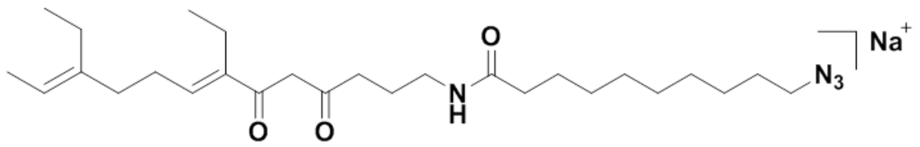

39

$[M+Na]^+$ calc.: m/z = 497.3462

$[M+H]^+$ calc.: m/z = 475.3643

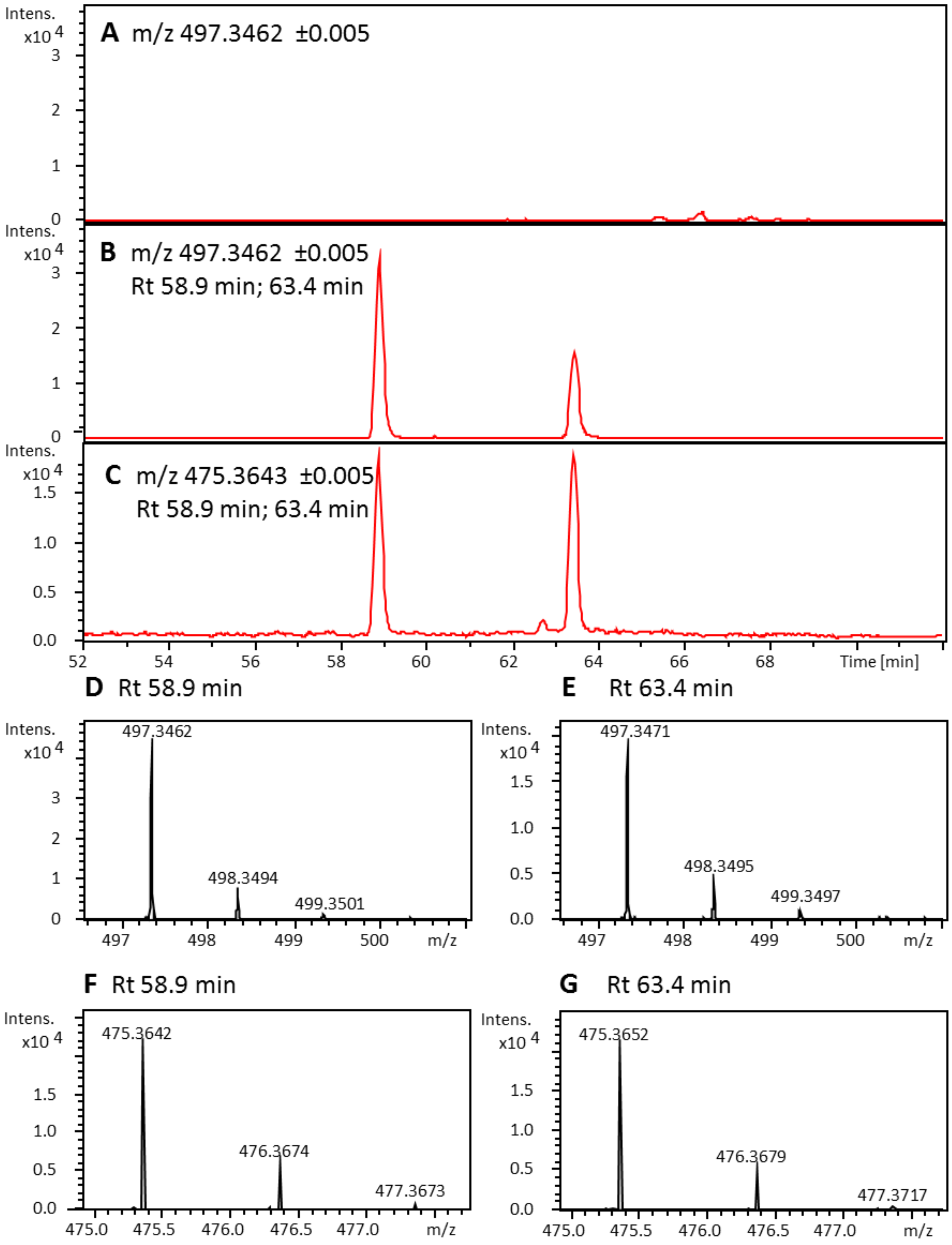

Figure 14: LC-ESI-HRMS analysis of the organic extracts of *S. lasaliensis* ACP12 (S970A) grown in the absence (**A**) and in the presence (**B** and **C**) of probe **8** (final concentration 4.0 mM; 0.1 mmol each day): $[M+Na]^+$ and $[M+H]^+$ extracted ion traces for the off-loaded polyketide **39** are shown (**B** and **C**, respectively). Further work is in progress to establish the origin of two peaks for **39** (possibly arising from the presence of isomers). This specie was absent in all the control samples (e.g. in absence of the probe, **A**, and in extracts obtained from the use of different probes). The high resolution masses and isotopic abundances for **39** are shown (**D** to **G**).

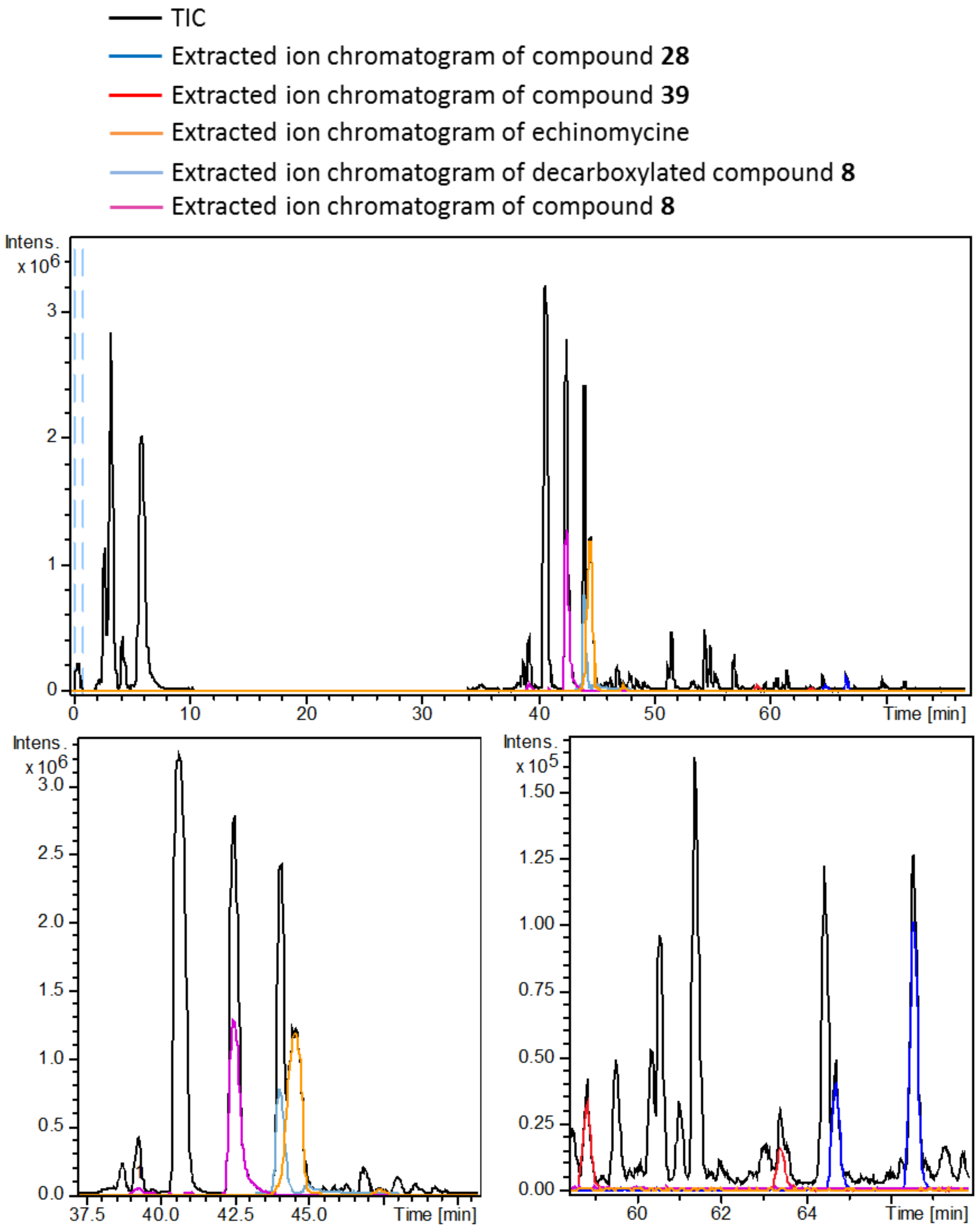

Figure 15: The comparison between the total ion chromatogram (TIC) of the organic extracts of *S. lasaliensis* ACP12 (S970A) grown in presence of probe **8** and the extracted ion chromatogram of polyketide **28** (blue) and the pentaketide **39** (red) is shown. Both polyketides **28** and **39** are clearly detectable also in the TIC.

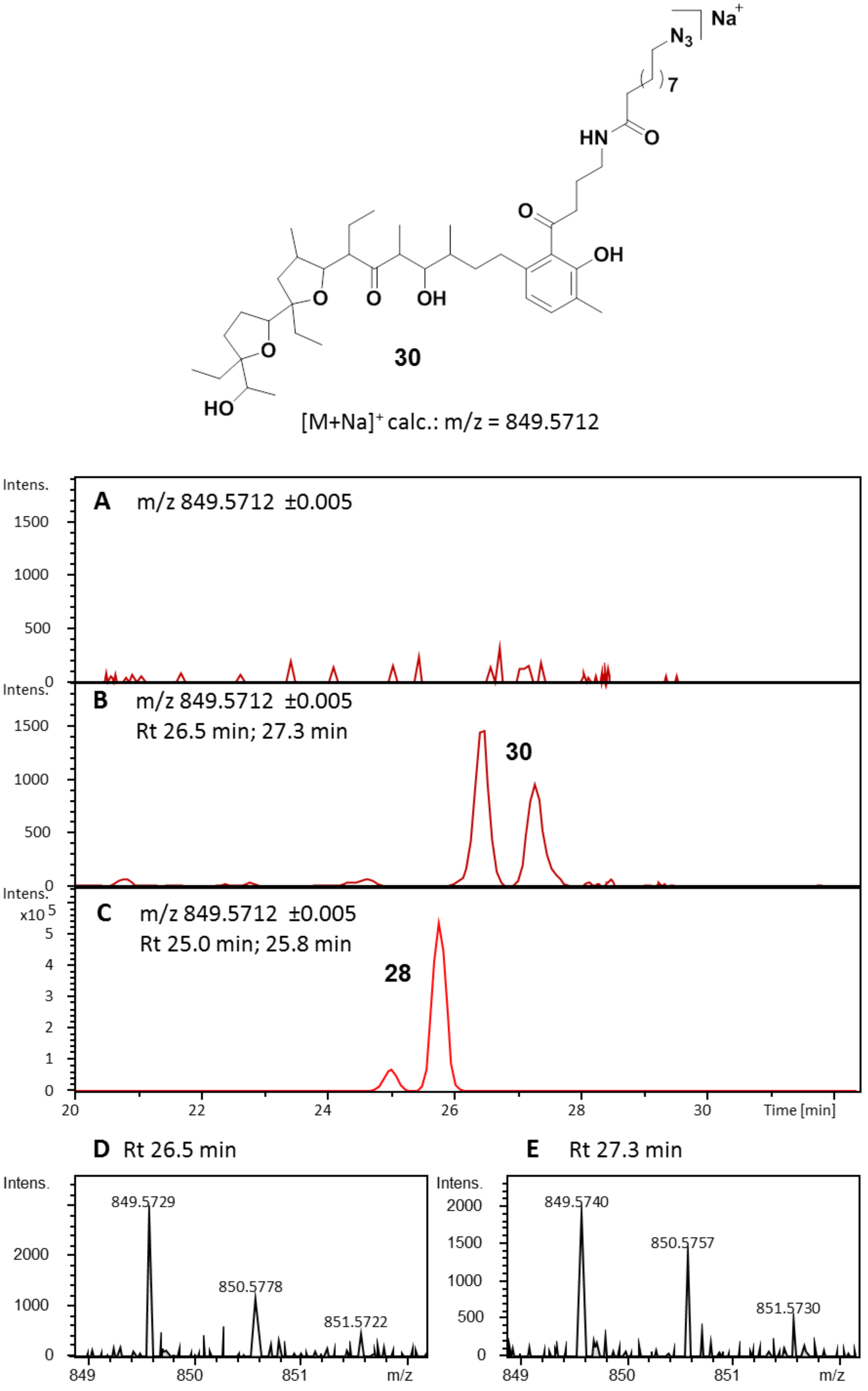
Na+
N3
7
HN
O
O
OH
O
O
OH
O
HO
30
[M+Na]+ calc.: m/z = 849.5712
Intens.
A m/z 849.5712 ±0.005
1500
1000
500
0
B m/z 849.5712 ±0.005
Rt 26.5 min; 27.3 min
30
C m/z 849.5712 ±0.005
Rt 25.0 min; 25.8 min
28
x10 5
20
22
24
26
28
30
Time [min]
D Rt 26.5 min
849.5729
850.5778
851.5722
2000
849
850
851
m/z
E Rt 27.3 min
849.5740
850.5757
851.5730

Figure 16: LC-ESI-HRMS analysis of the organic extracts of ΔlasB-ACP12 (S970A) (**A** and **B**) and *S. lasaliensis* ACP12 (S970A) (**C**) grown in the presence (**B** and **C**) and in absence (**A**) of probe **8** (final concentration 4.0 mM; 0.01 mmol each day): $[M+Na]^+$ extracted ion traces for the putative off-loaded polyketides **30** and **28** are shown (**B** and **C**, respectively, Analysis Method 3). Their stereochemistry is yet to be separately determined; further work is in progress to establish this, as well as the origin of two peaks for **28** and **30** (possibly arising from the presence of isomers/conformers). These species have been also detected as ammonium adducts (data not shown) and were absent in all the control samples (e.g. in absence of the probe, **A**, and in extracts obtained from the use of different probes). The high resolution masses and isotopic abundances for **30** (**D** and **E**) are shown.

3.4.8 LC-HRMS and MS/MS analysis of off-loading of probe 8 and its decarboxylated form from *S. lasaliensis* ACP12 (S970A) crude extracts

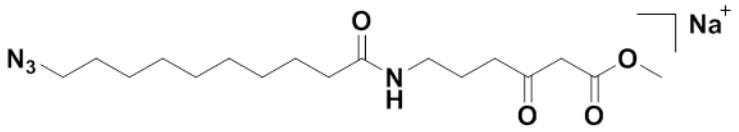

8

$[M+Na]^+$ calc.: m/z = 377.2159

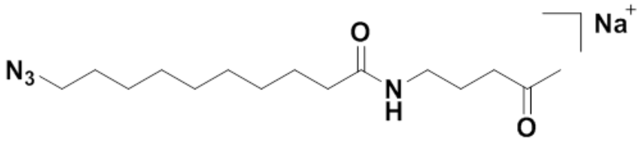

$[M+Na]^+$ calc.: m/z = 319.2104

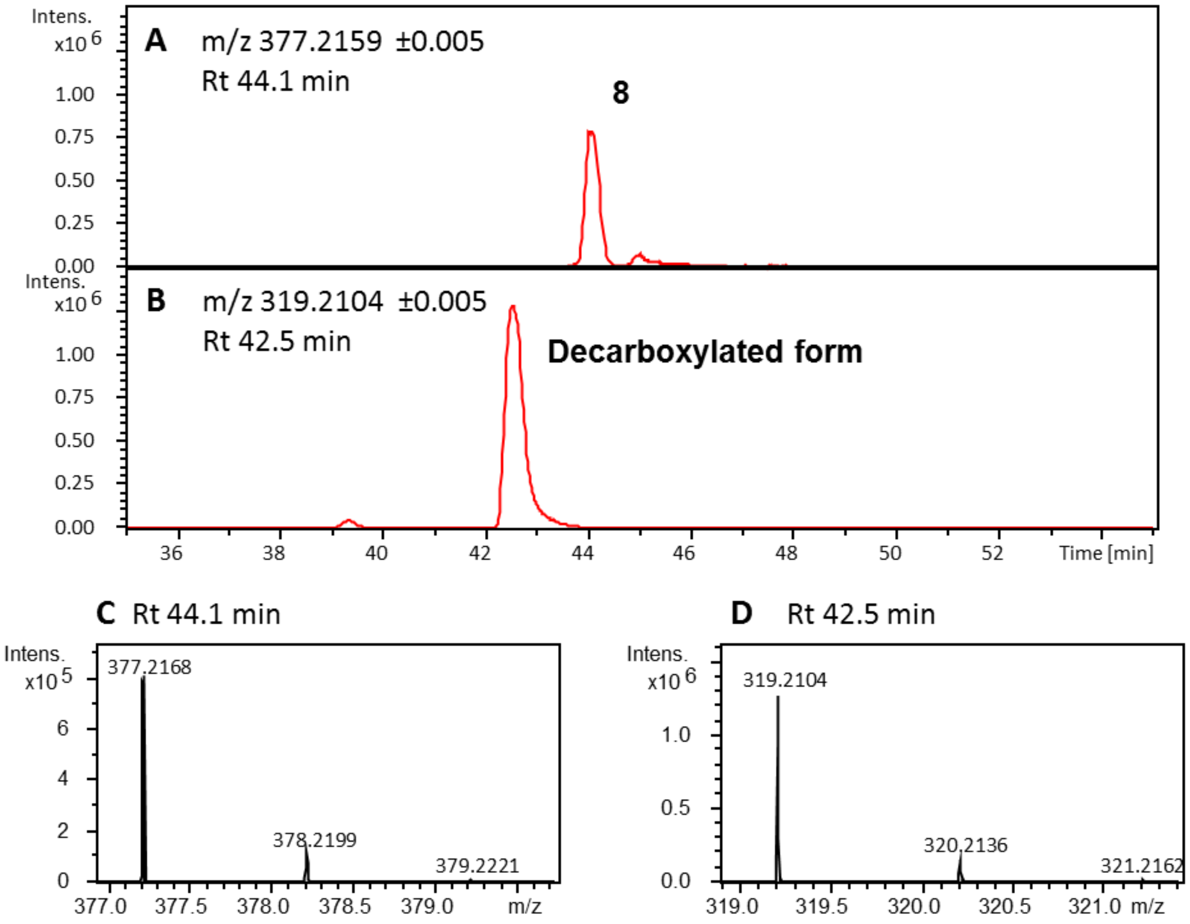

Figure 17: LC-ESI-HRMS analysis of the probe **8** (**A**) and of the correspondent decarboxylated form following *in vivo* hydrolysis (**B**) in the crude extract of *S. lasaliensis* ACP12 (S970A) after 5 days. The high resolution masses and isotopic abundances for **8** and for the correspondent decarboxylated form are shown in (**C**) and (**D**), respectively.

3.5 LC-HRMS and MS/MS analysis of off-loaded polyketides generated from *S. lasaliensis* ACP5 (S3799A)

3.5.1 LC-HRMS and MS/MS analysis of off-loading of 38, generated from *S. lasaliensis* ACP5 (S3799A) and probe 7

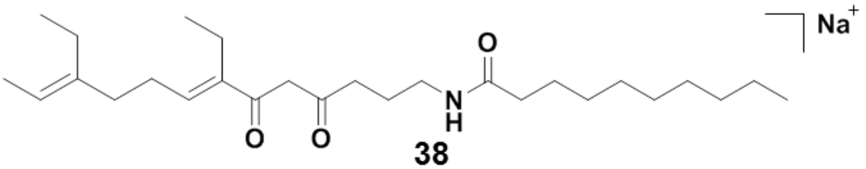

38

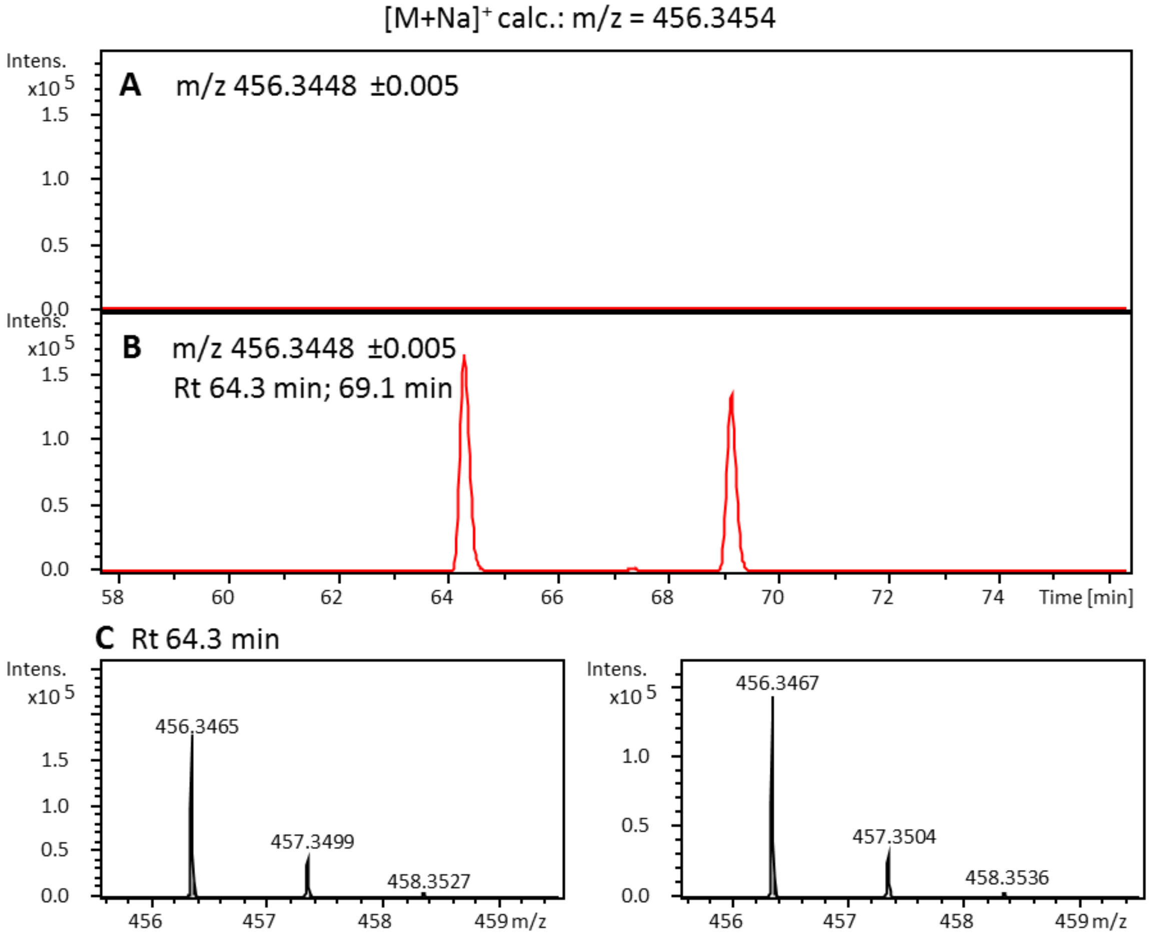

Figure 18: HR-ESI-HRMS analysis of the organic extracts of *S. lasaliensis* ACP5 (S3799A) grown in the absence (**A**) and in the presence (**B**) of probe **7** (final concentration 3.0 mM; day 2 and 3: 0.01 mmol, day 4 and 5: 0.005 mmol): $[M+Na]^+$ extracted ion trace for the off-loaded polyketide **38** is shown (**B**). Further work is in progress to establish the origin of two peaks for **38**. These species are identical to those identified from the growth of *S. lasaliensis* ACP12 (S970A) in the presence of **7** (Figure 16). The high resolution masses and isotopic abundances for **38** are shown (**C** and **D**).

3.5.2 LC-HRMS and MS/MS analysis of off-loading of 39, generated from *S. lasaliensis* ACP5 (S3799A) and probe 8

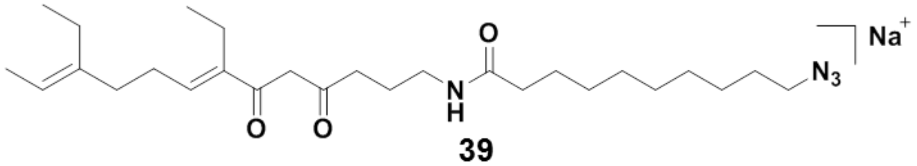

39

$[M+Na]^+$ calc.: m/z = 497.3462

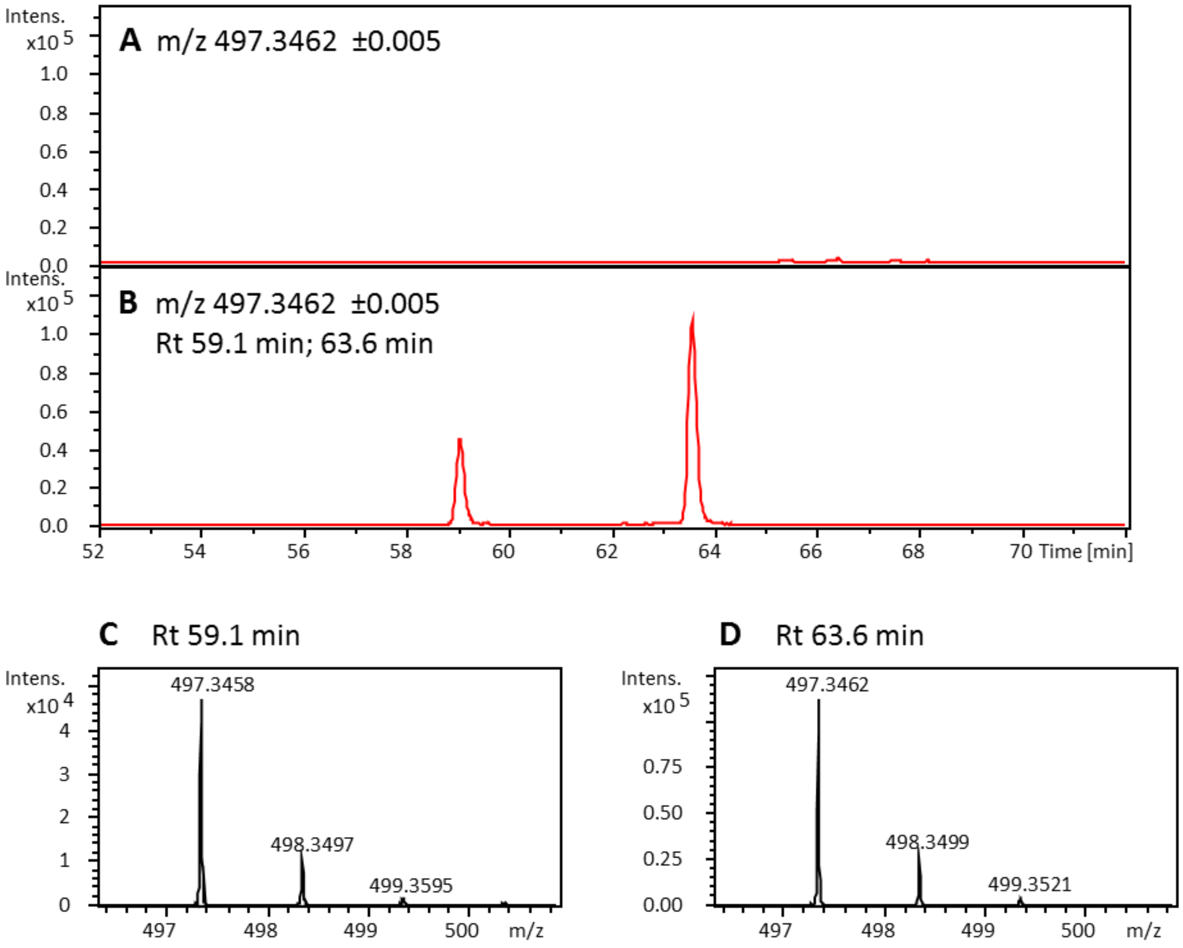

Figure 19: HR-ESI-HRMS analysis of the organic extracts of *S. lasaliensis* ACP5 (S3799A) grown in the absence (**A**) and in the presence (**B**) of probe **8** (final concentration 4.0 mM; 0.1 mmol each day): $[M+Na]^+$ extracted ion trace for the off-loaded polyketide **39** is shown (**B**). Further work is in progress to establish the origin of two peaks for **39**. These species are identical to those identified from the growth of *S. lasaliensis* ACP12 (S970A) in the presence of **8** (Figure 26). The high resolution masses and isotopic abundances for **39** are shown (**C** and **D**).

3.6 Off-loading of functionalized polyketides from *S. lasaliensis* WT and ACP12 (S970A) via chemical probes 9

3.6.1 LC-HRMS and MS/MS analysis of 31, generated from *S. lasaliensis* ACP12 (S970A) and probe 9

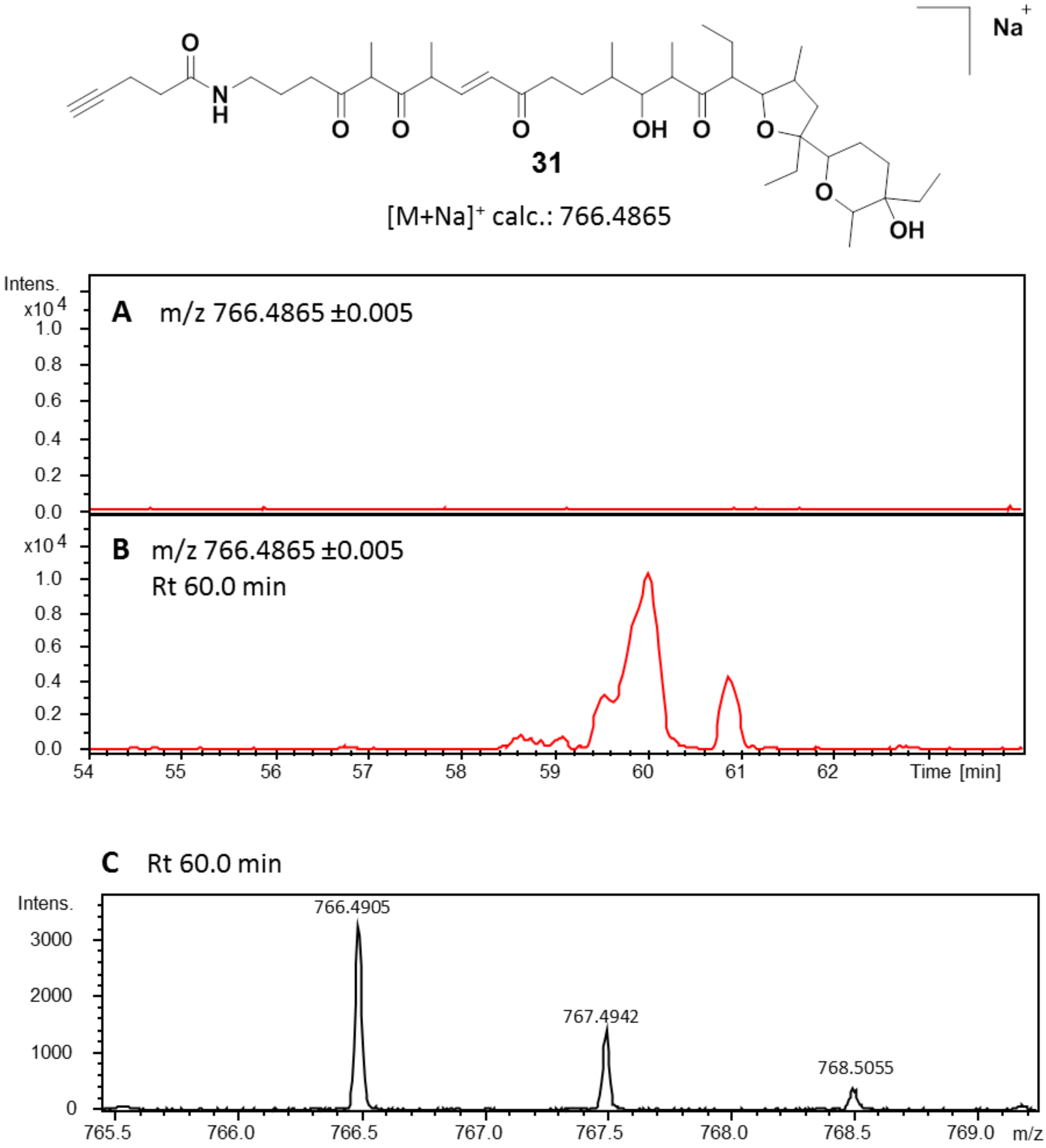

Figure 20: LC-ESI-HRMS analysis of the organic extracts of *S. lasaliensis* ACP12 (S970A) grown in the absence (**A**) and in the presence (**B**) of probe **9** (2.5 mM final concentration): $[M+Na]^+$ extracted ion trace for the off-loaded polyketide **31** is shown (**B**). These species have also been detected as ammonium adduct (data not shown) and were absent in all the control samples (e.g. in the absence of the probe, **A**, and in

extracts obtained from the use of different probes). The high resolution mass and isotopic abundance for **31** are shown (**C**).

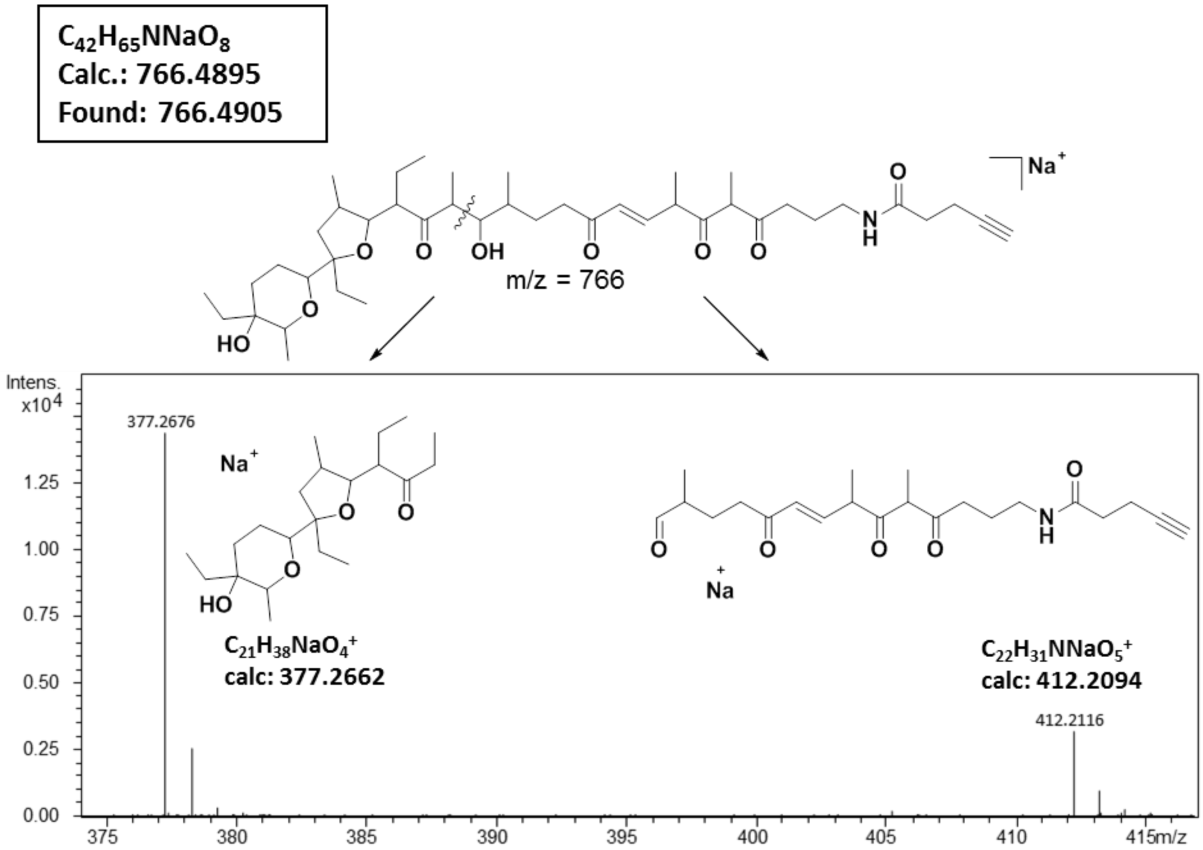

Figure 21: HR-ESI-MS^2 analysis of the undecaketide **31**. Fragmentation of the $[M+Na]^+$ adduct generates a left–hand fragment of *m/z* 377, characteristic of lasalocid A, as well as a right-hand fragment containing the alkyne handle (*m/z* 412).

3.6.2 LC-HRMS of 31 and 35, generated from wild type *S. lasaliensis* and probe 9

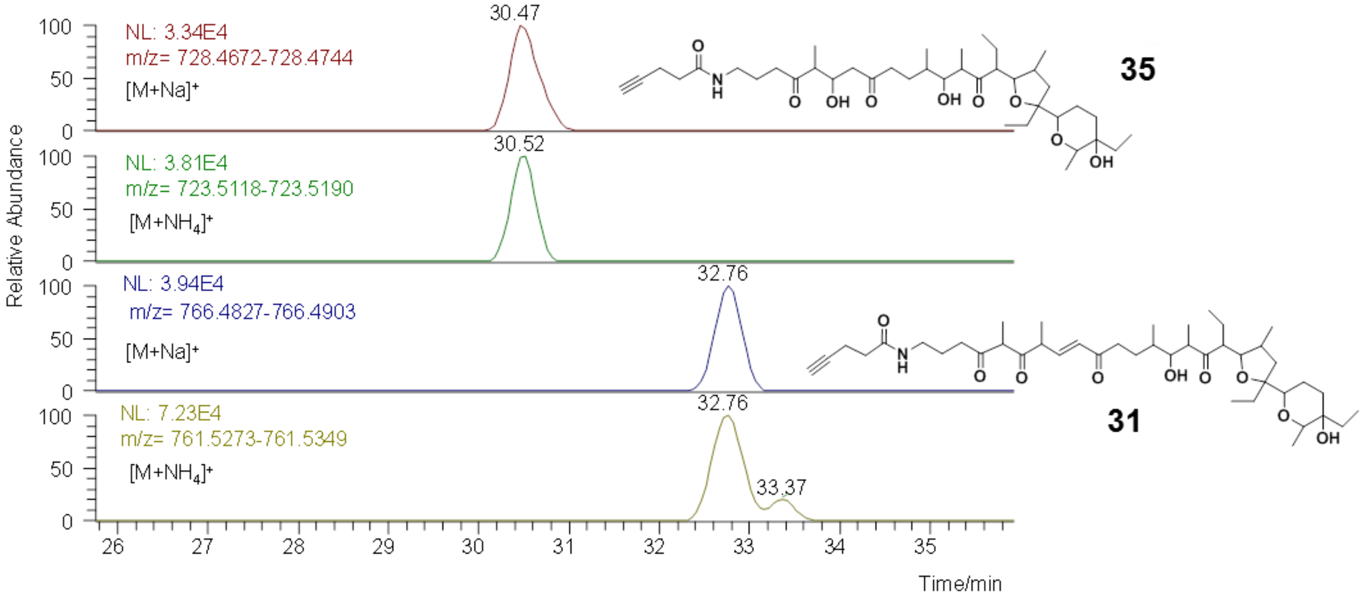

Figure 22: Micro LC-ESI-HRMS analysis (LTQ- Orbitrap, Cambridge)[12] of the organic extracts of wild type *S. lasaliensis* grown in the in the presence of probe **9** (10 mM) on MYM-agar plates for 5 days as reported in literature.[12] The $[M+Na]^+$ and $[M+NH_4]^+$ extracted ion trace (5 ppm mass accuracy) for the polyketides **31** and **35** are shown. These species were absent in all control samples (data not shown). **35** mirrors its *N*-acetyl counterpart previously isolated and characterised by HR-MS^n in detail (see reference 12 and LC-MS traces below).

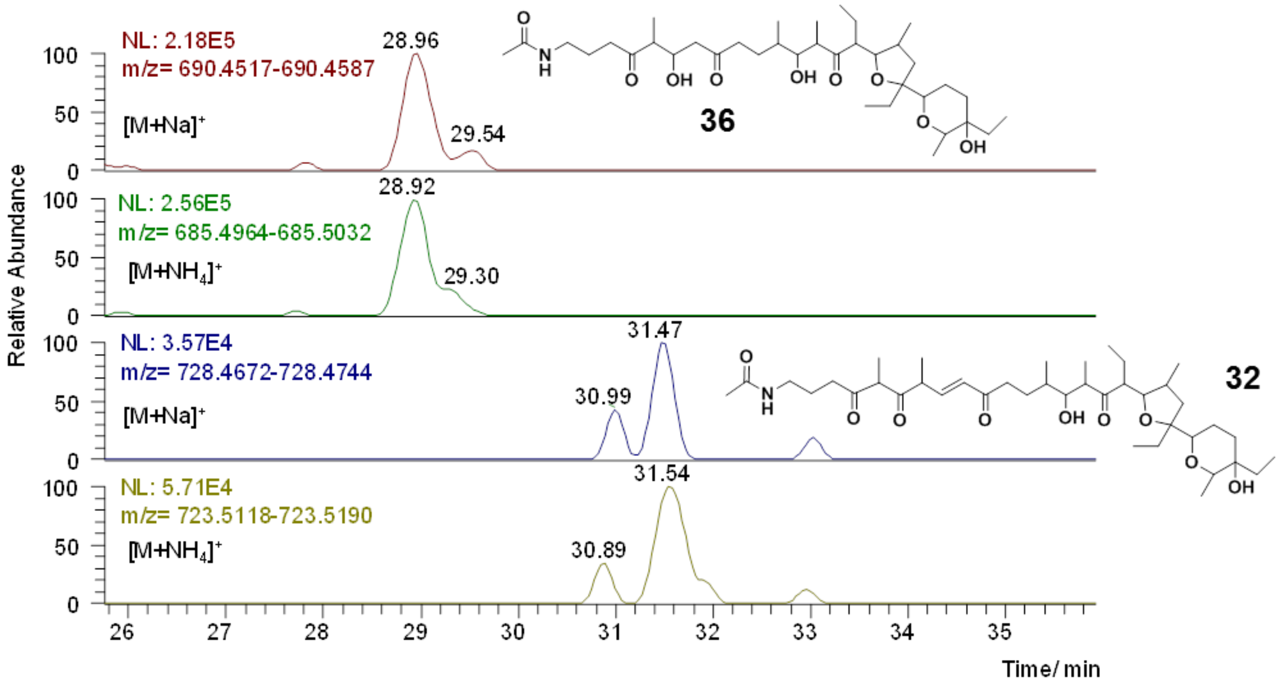

3.7 Off-loading of functionalized polyketides from *S. lasaliensis* ACP12 (S970A) via chemical probes 11a, 11b and 13

3.7.1 LC-HRMS and MS/MS analysis of polyketides 37a and 37b, generated from *S. lasaliensis* ACP12 (S970A) and probes 11a and 11b

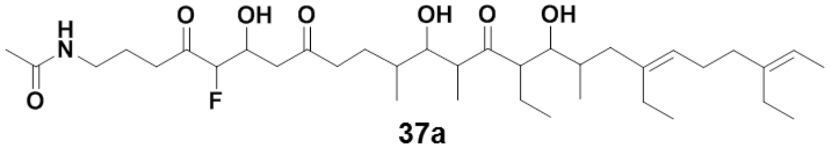

37a

$[M+Na]^+$ calc: 662.4403

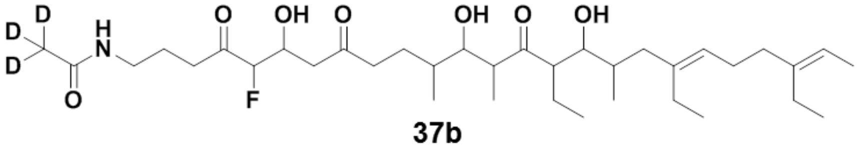

37b

$[M+Na]^+$ calc: 665.4591

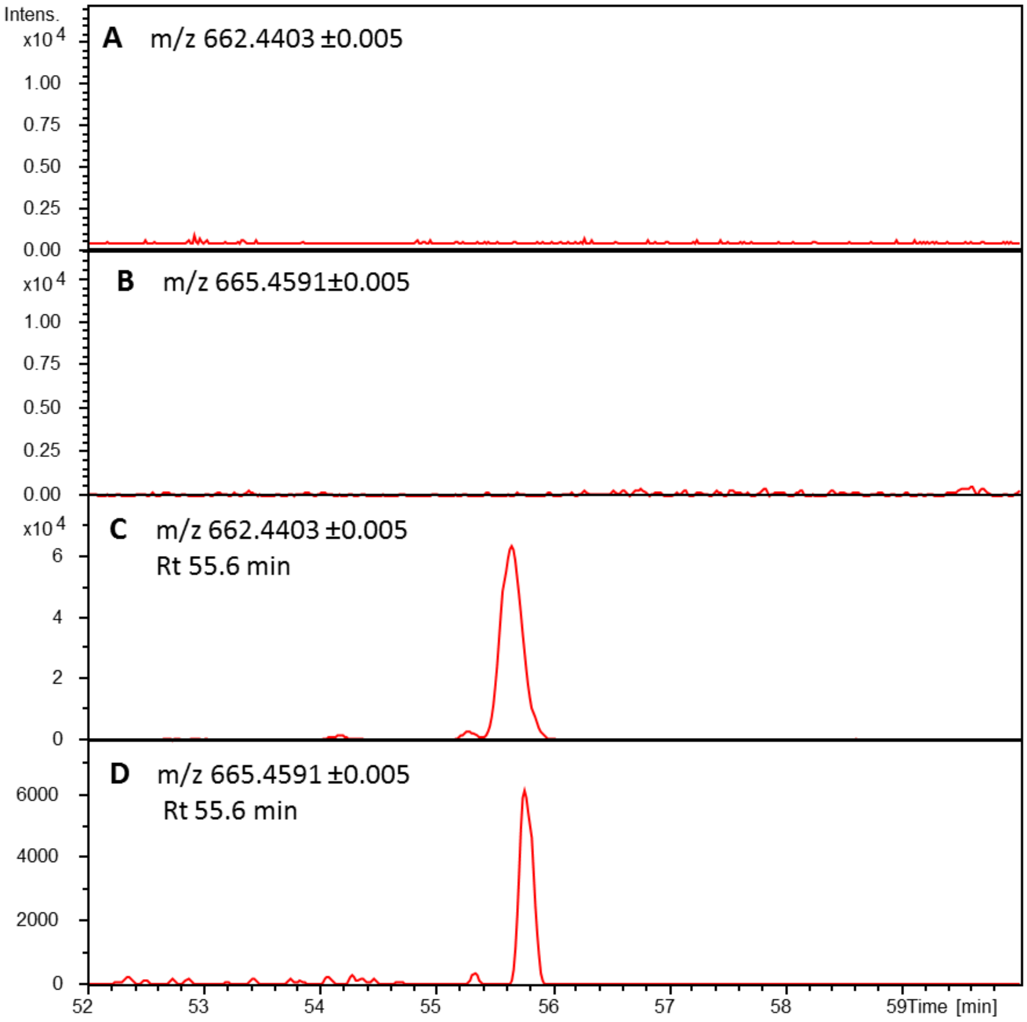

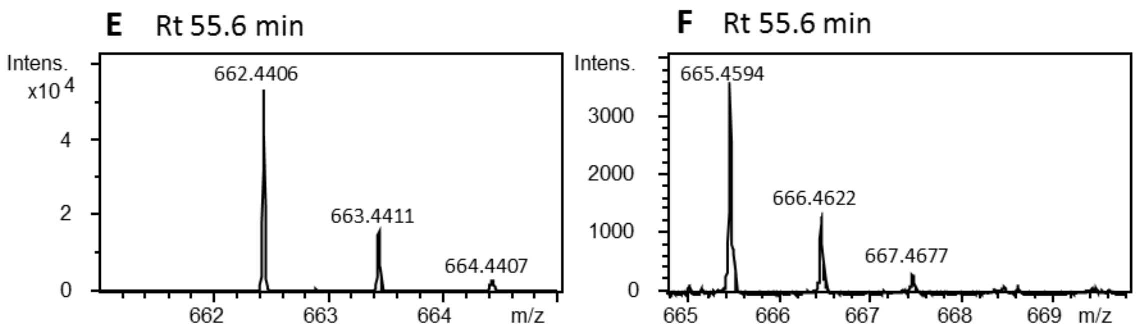

Figure 23: LC-ESI-HRMS analysis of the organic extracts of ACP12 *S. lasaliensis* (S970A) grown in the absence (**A, B**) and in the presence (**C, D**) of probe **11a** and **11b** respectively (2.5 mM final concentration): $[M+Na]^+$ extracted ion traces for the off-loaded polyketides **37a** and **37b** are shown (**C** and **D,** respectively). These species were absent in all the control samples (e.g. in absence of the probes, **A** and **B**, and in extracts obtained from the use of different probes). The high resolution masses and isotopic abundances for **37a** and **37b** are shown (**E** and **F**, respectively).

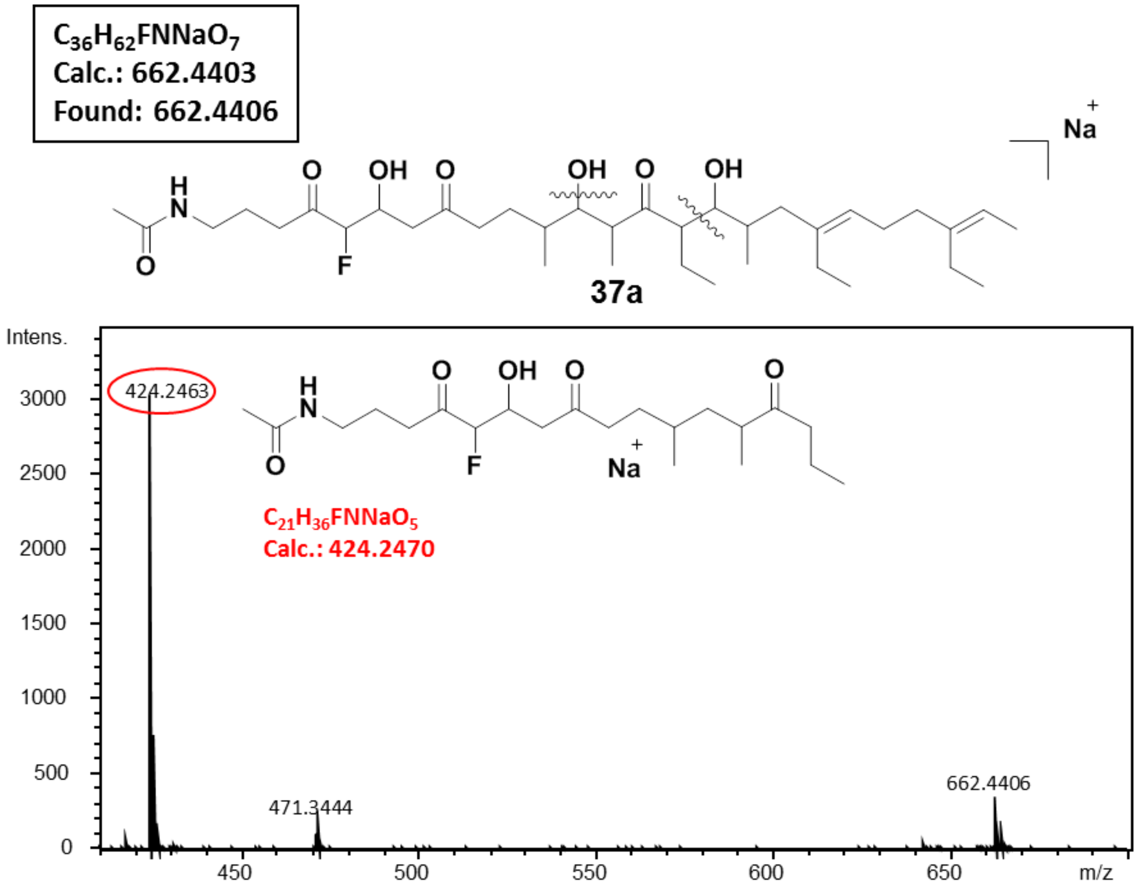

Figure 24: HR-ESI-MS[2] analysis of the off-loaded intermediate **37a**. The fragmentation pattern (the mechanisms of which are currently under detailed investigation) is identical to that of the analogous compounds reported previously in literature.[12]

3.7.2 LC-HRMS and MS/MS analysis of off-loading of 34, 40-43, generated from *S. lasaliensis* ACP12 (S970A) and probe 13

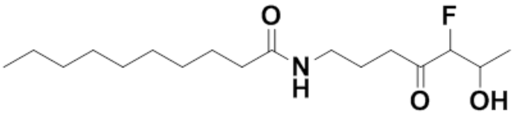

43

$[M+H]^+$ calc.: 318.2439

$[M+Na]^+$ calc.: 340.2258

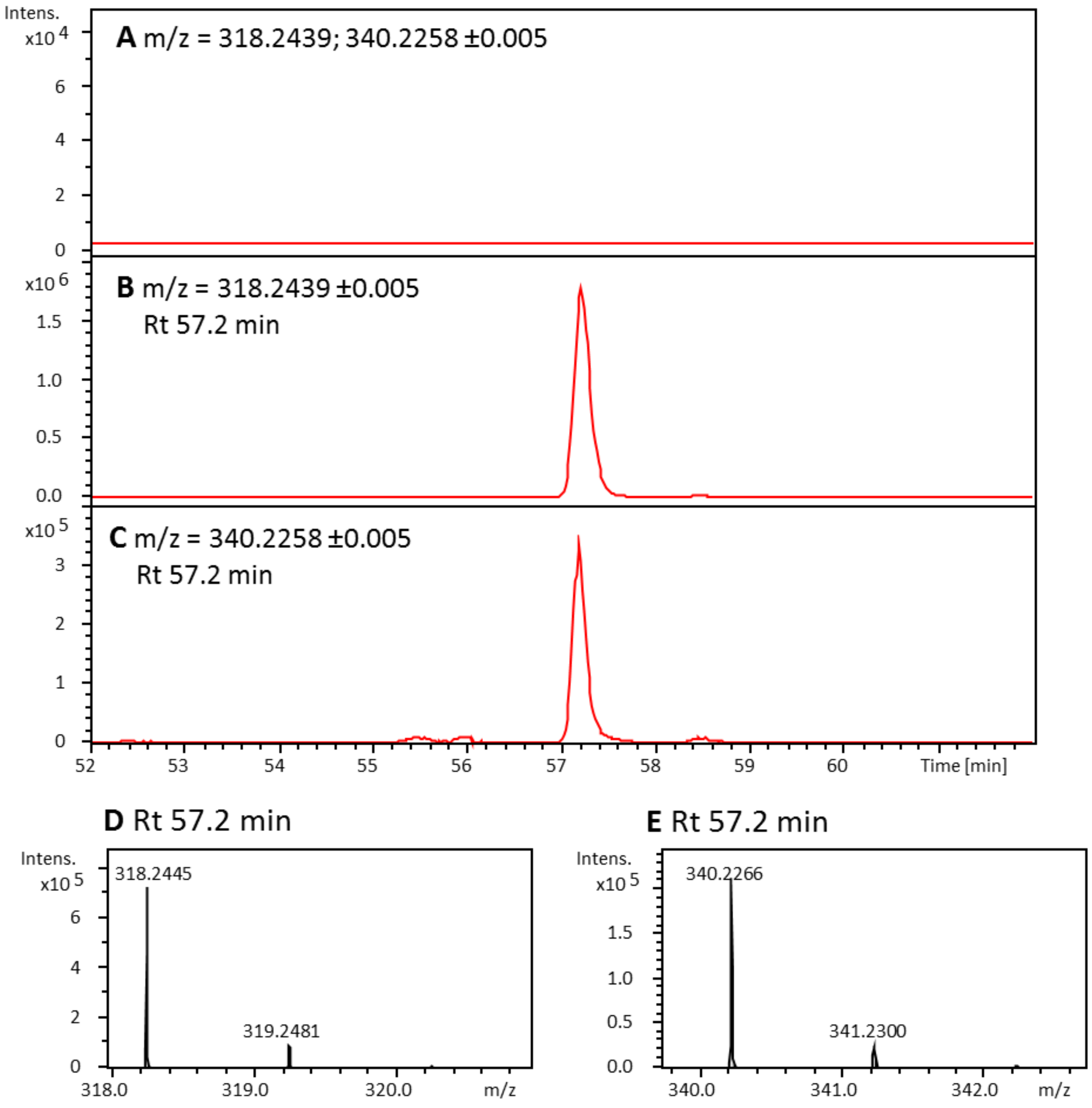

Figure 25: LC-ESI-HRMS analysis of the organic extracts of *S. lasaliensis* ACP12 (S970A) grown in the absence (**A**) and in the presence (**B** and **C**) of probe **13** (final concentration 3.0 mM; day 2 and 3: 0.01 mmol, day 4 and 5: 0.005 mmol): $[M+H]^+$ and $[M+Na]^+$ extracted ion traces for the off-loaded polyketide **43** are

shown (**B** and **C**, respectively). This specie was absent in all the control samples (e.g. in absence of the probe, **A**, and in extracts obtained from the use of different probes). The high resolution masses and isotopic abundances for **43** are shown (**D** and **E**).

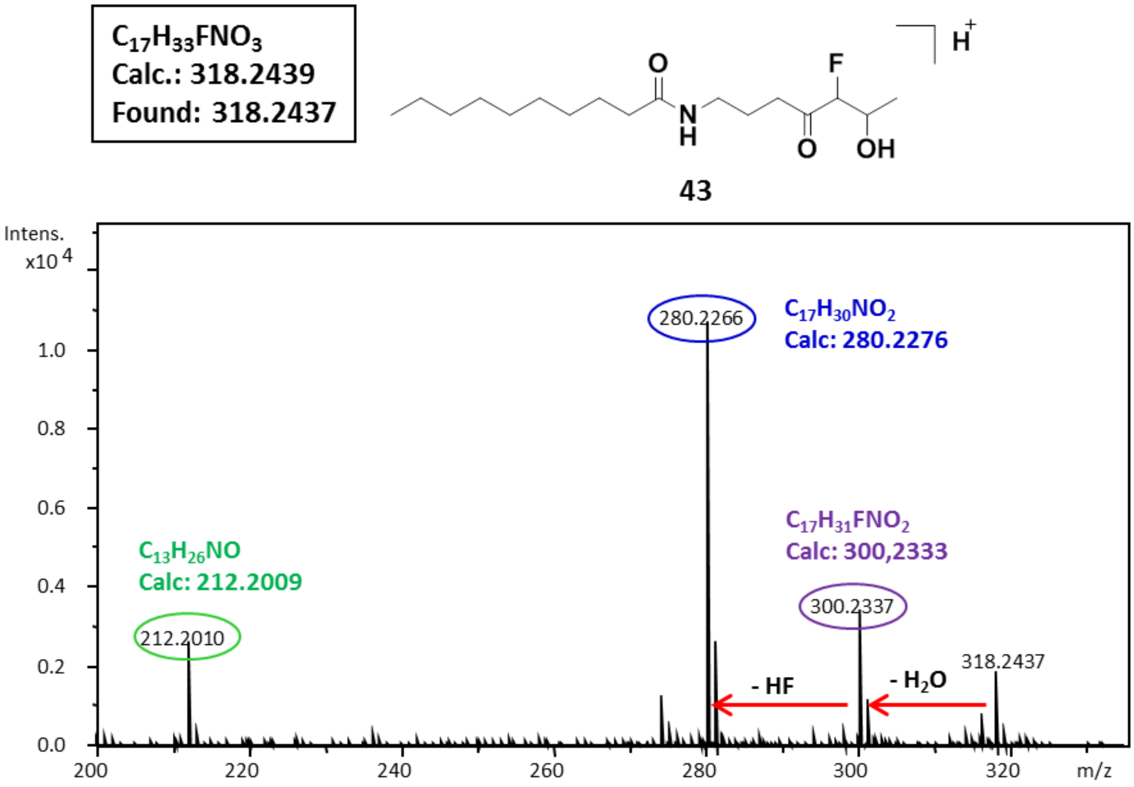

Figure 26: HR-ESI-MS^2 analysis of the off-loaded intermediate **43**. The fragmentation pathway leading to m/z 212 is currently under detailed investigation.

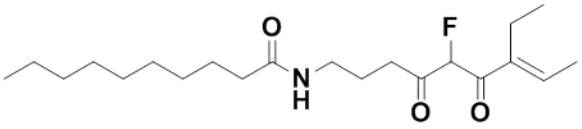

42

[M+H]$^+$ calc.: 370.2752

[M+Na]$^+$ calc.: 392.2571

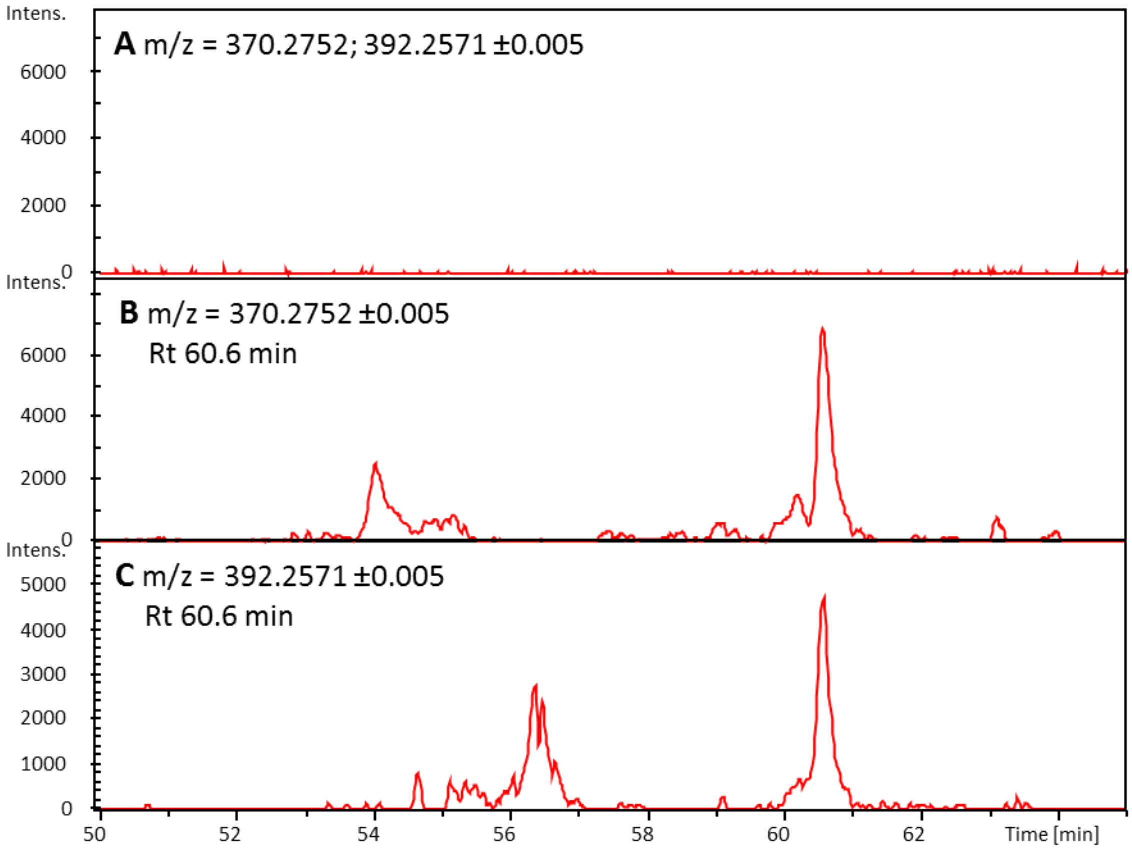

Figure 27: LC-ESI-HRMS analysis of the organic extracts of *S. lasaliensis* ACP12 (S970A) grown in the absence (**A**) and in the presence (**B** and **C**) of probe **13** (final concentration 3.0 mM; day 2 and 3: 0.01 mmol, day 4 and 5: 0.005 mmol): [M+H]$^+$ and [M+Na]$^+$ extracted ion traces for the off-loaded polyketide **42** are shown (**B** and **C**, respectively). This specie was absent in all the control samples (e.g. in absence of the probe, **A**, and in extracts obtained from the use of different probes).

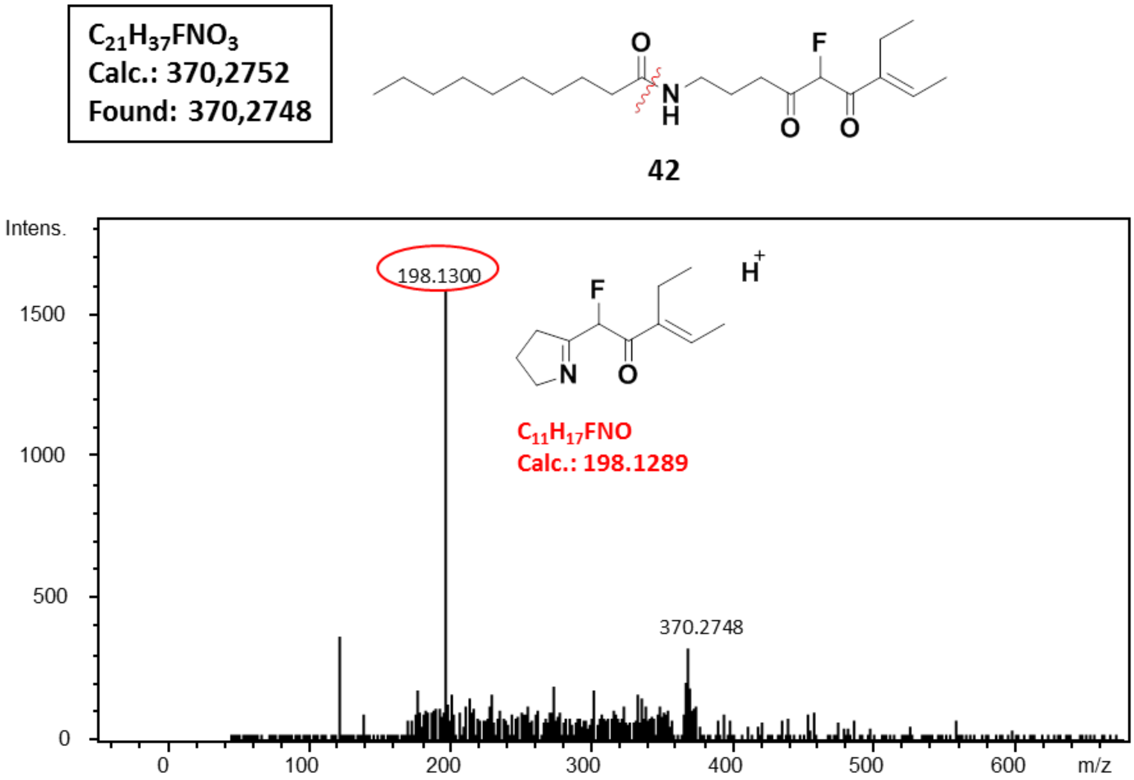

Figura 19: HR-ESI-MS^2 analysis of the off-loaded intermediate **42** eluting at Rt 60.6 min. The fragment *m/z* 198 derives from the cyclization of the ketone and the amine generated from loss of the decanoyl chain.

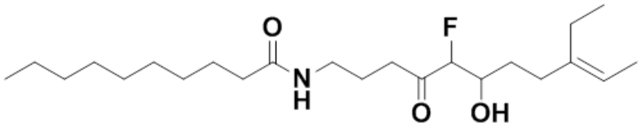

41

$[M+H]^+$ calc.: 400.3221

$[M+Na]^+$ calc.: 422.3041

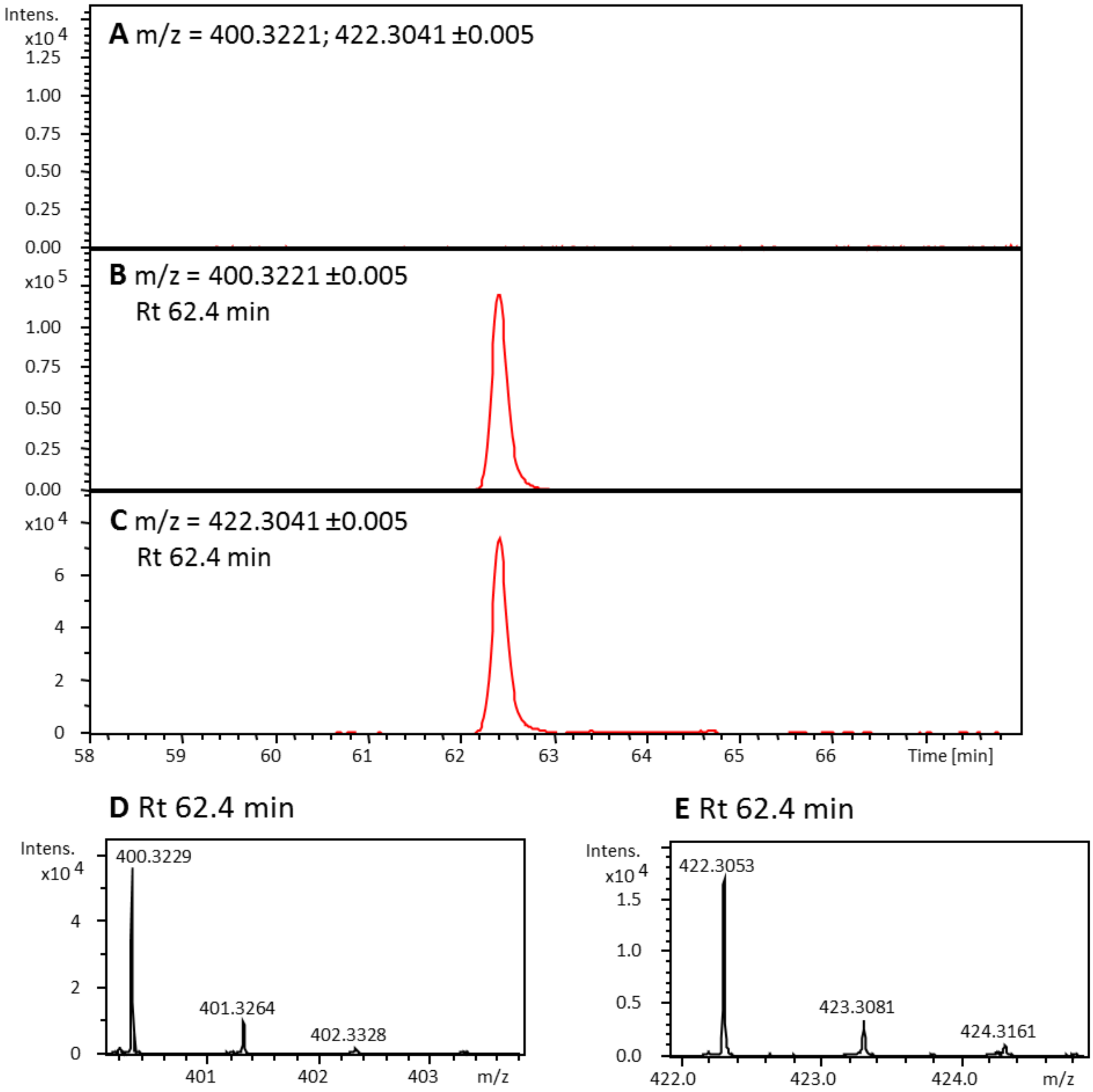

Figure 28: LC-ESI-HRMS analysis of the organic extracts of *S. lasaliensis* ACP12 (S970A) grown in the absence (**A**) and in the presence (**B** and **C**) of probe **13** (final concentration 3.0 mM; day 2 and 3: 0.01 mmol, day 4 and 5: 0.005 mmol): $[M+H]^+$ and $[M+Na]^+$ extracted ion traces for the off-loaded polyketide **41** are shown (**B** and **C**, respectively). This specie was absent in all the control samples (e.g. in absence of the probe, **A**, and in extracts obtained from the use of different probes). The high resolution masses and isotopic abundances for **41** are shown (**D** and **E**).

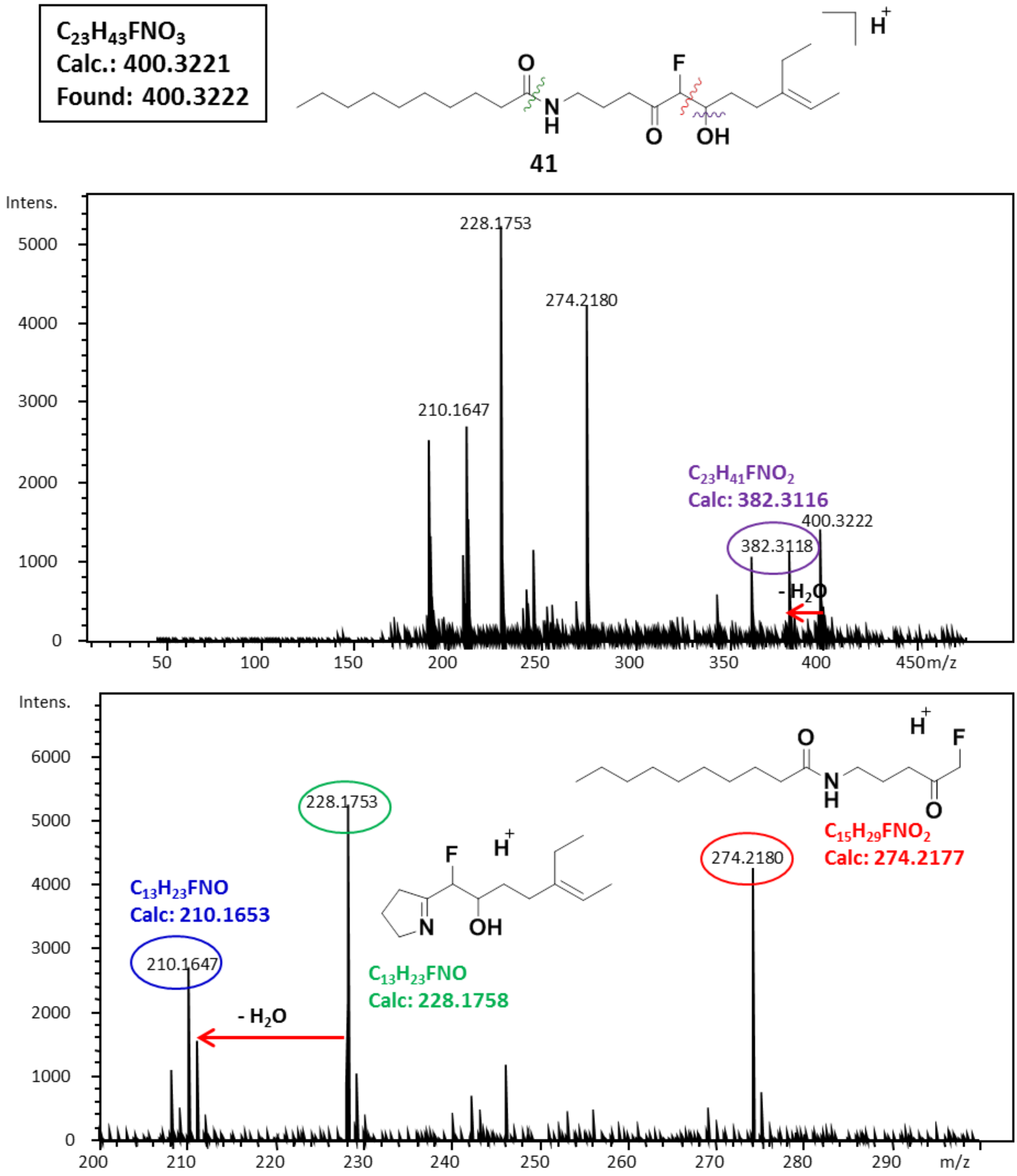

Figure 29: HR-ESI-MS^2 analysis of the off-loaded intermediate **41**. Loss of water from of the $[M+H]^+$ adduct generates the *m/z* 382 fragment, while McLafferty fragmentation generates the *m/z* 274 fragment. *m/z* 228 derives from the cyclization of the ketone and the amine resulting from loss of the decanoyl chain; further loss of water gives origin to *m/z* 210.

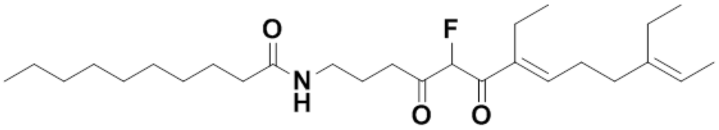

40

$[M+H]^+$ calc.: 452.3534

$[M+Na]^+$ calc.: 474.3354

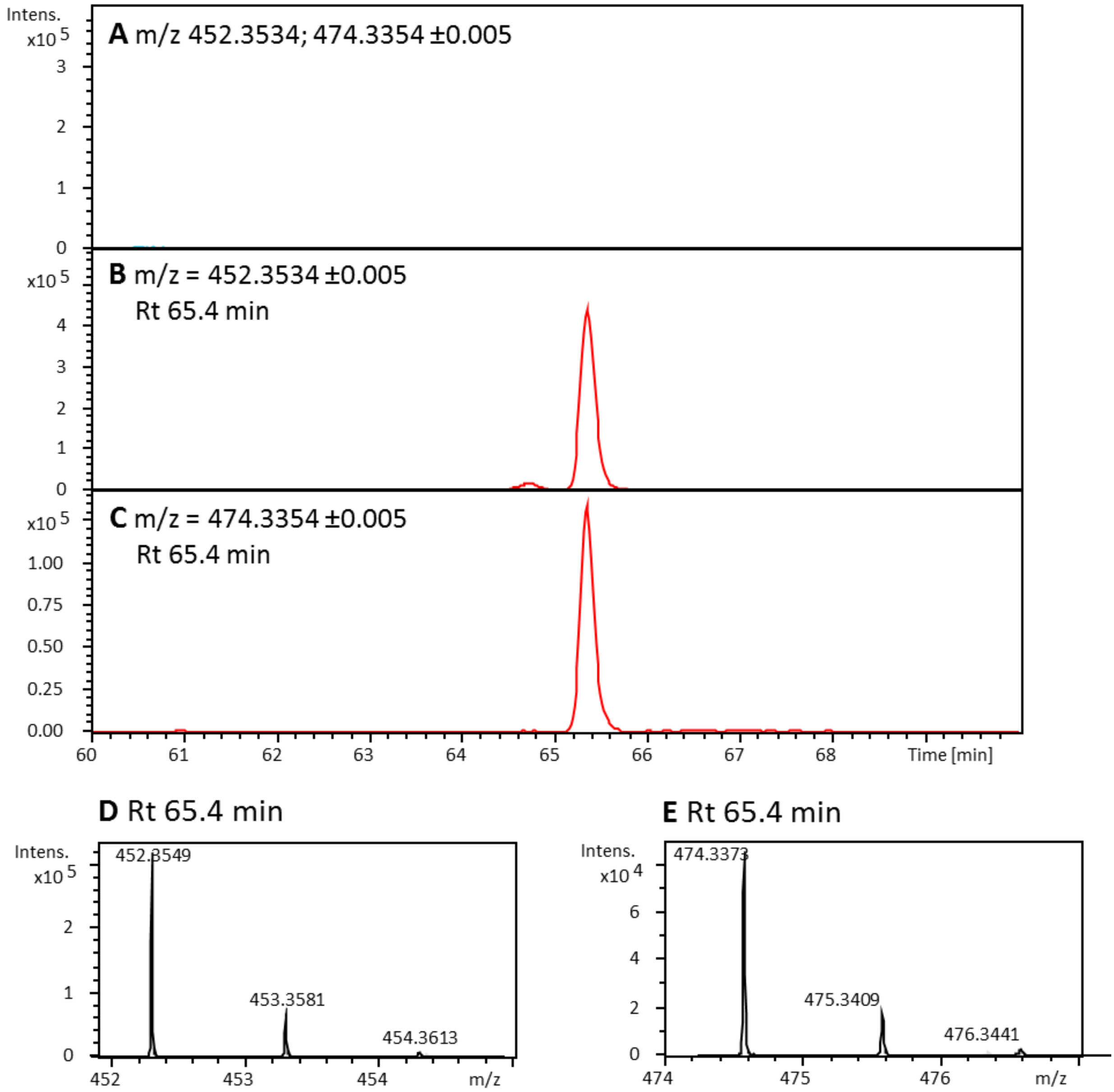

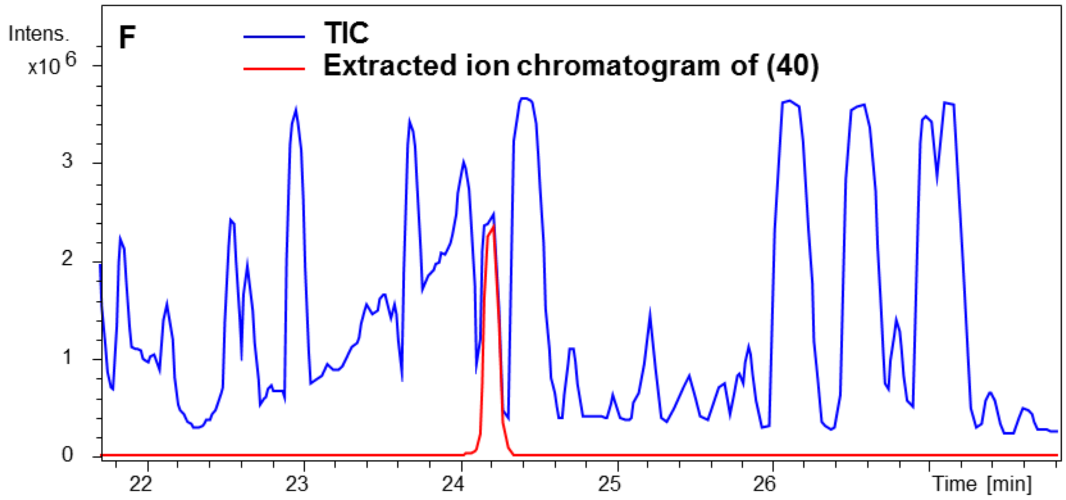

Figure 30: LC-ESI-HRMS analysis of the organic extracts of *S. lasaliensis* ACP12 (S970A) grown in the absence (**A**) and in the presence (**B** and **C**) of probe **13** (final concentration 3.0 mM; day 2 and 3: 0.01 mmol, day 4 and 5: 0.005 mmol): $[M+H]^+$ and $[M+Na]^+$ extracted ion traces for the off-loaded polyketide **40** are shown (**B** and **C**, respectively). This specie was absent in all the control samples (e.g. in absence of the probe, **A**, and in extracts obtained from the use of different probes). The high resolution masses and isotopic abundances for **40** are shown (**D** and **E**). The comparison between the total ion chromatogram (TIC) of the organic extracts of *S. lasaliensis* ACP12 (S970A) grown in presence of probe **13** and the extracted ion chromatogram of polyketide **40** is shown (**F**; spectra obtained using Method 3). Polyketide **40** is clearly detectable also in the TIC.

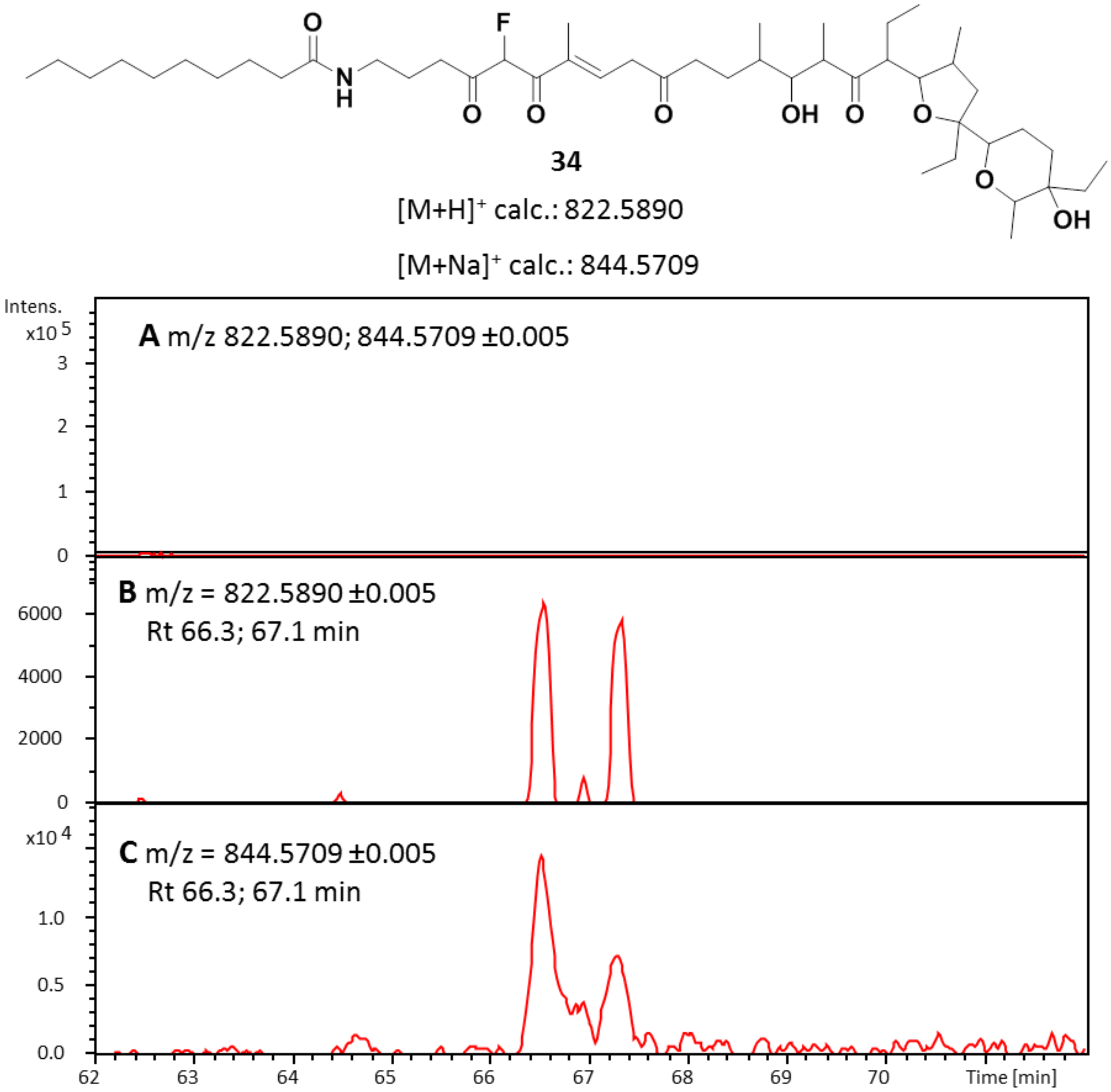

Figure 21: LC-ESI-HRMS analysis of the organic extracts of *S. lasaliensis* ACP12 (S970A) grown in the absence (**A**) and in the presence (**B** and **C**) of probe **13** (final concentration 3.0 mM; day 2 and 3: 0.01 mmol, day 4 and 5: 0.005 mmol): $[M+H]^+$ and $[M+Na]^+$ extracted ion traces for the off-loaded polyketide **34** - detected in low amount- are shown (**B** and **C**, respectively). This specie was absent in all the control samples (e.g. in absence of the probe, **A**, and in extracts obtained from the use of different probes).

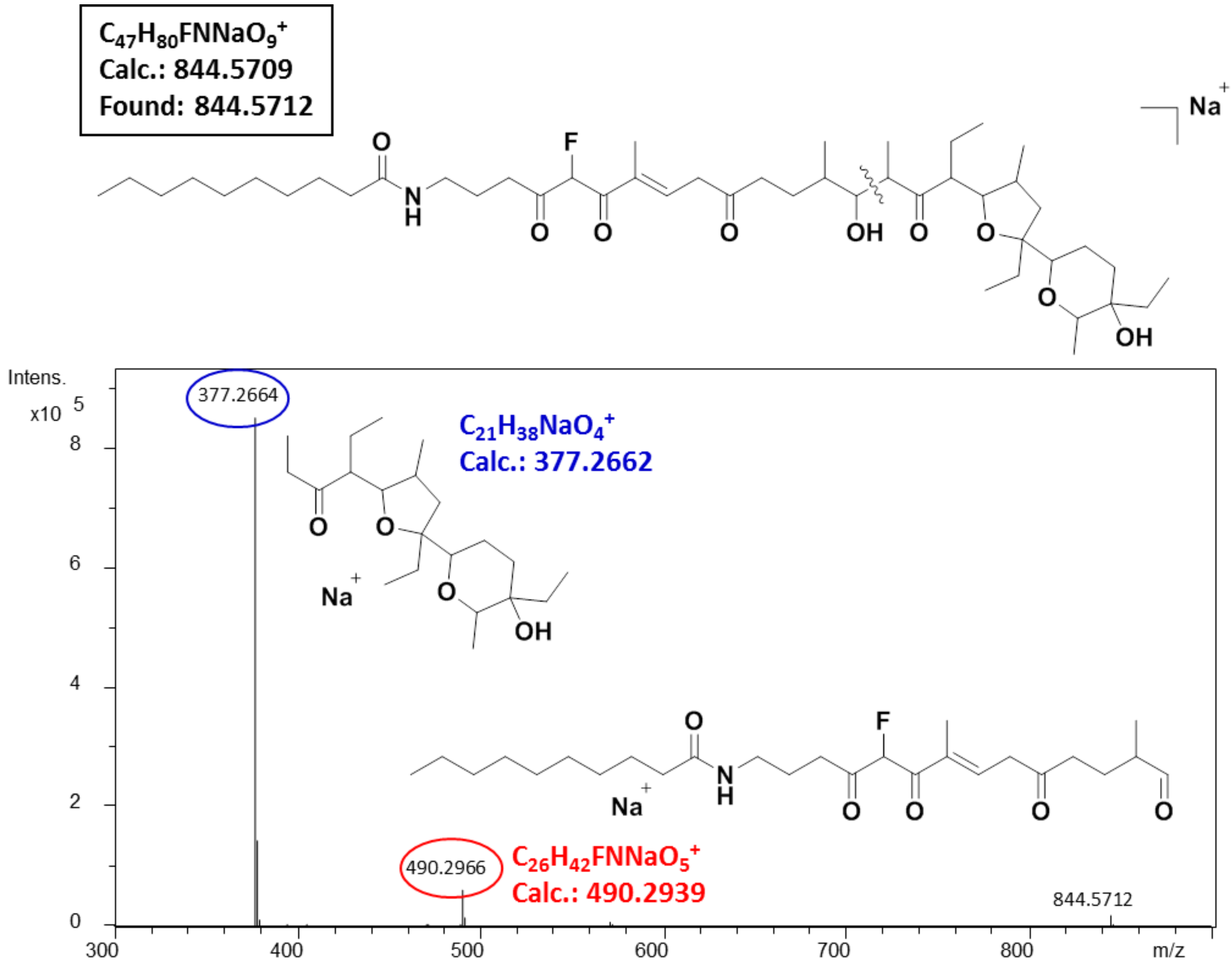

Figure 31: HR-ESI-MS^2 analysis of the intermediate **34**. Fragmentation of the $[M+Na]^+$ adduct generates a left–hand fragment of m/z 377, characteristic of lasalocid A, as well as a right-hand fragment containing the fluorinated portion (m/z 490).

4 Copper-catalyzed azide-alkyne cycloaddition (CuAAC) of alkyne and azide intermediates 25 and 26

General Procedure: The reaction was performed on 100 µL of the organic extracts previously dissolved in 1 mL HPLC-grade methanol. The extract solution was further diluted to 1 mL with HPLC-grade MeOH and then compounds **13** or **15** (1 eq), sodium ascorbate (10 mol%), copper sulfate pentahydrate (10 mol%) and TBTA (5 mol%) were added (equivalents were calculated based on fed amount of probe **3** or **9**). The reaction mixture was stirred at room temperature for 24 hours. Afterwards the reaction mixture was analyzed by LC-HRMS.

4.1 Copper-catalyzed azide-alkyne cycloaddition (CuAAC) of alkyne intermediates 25 with azide 44

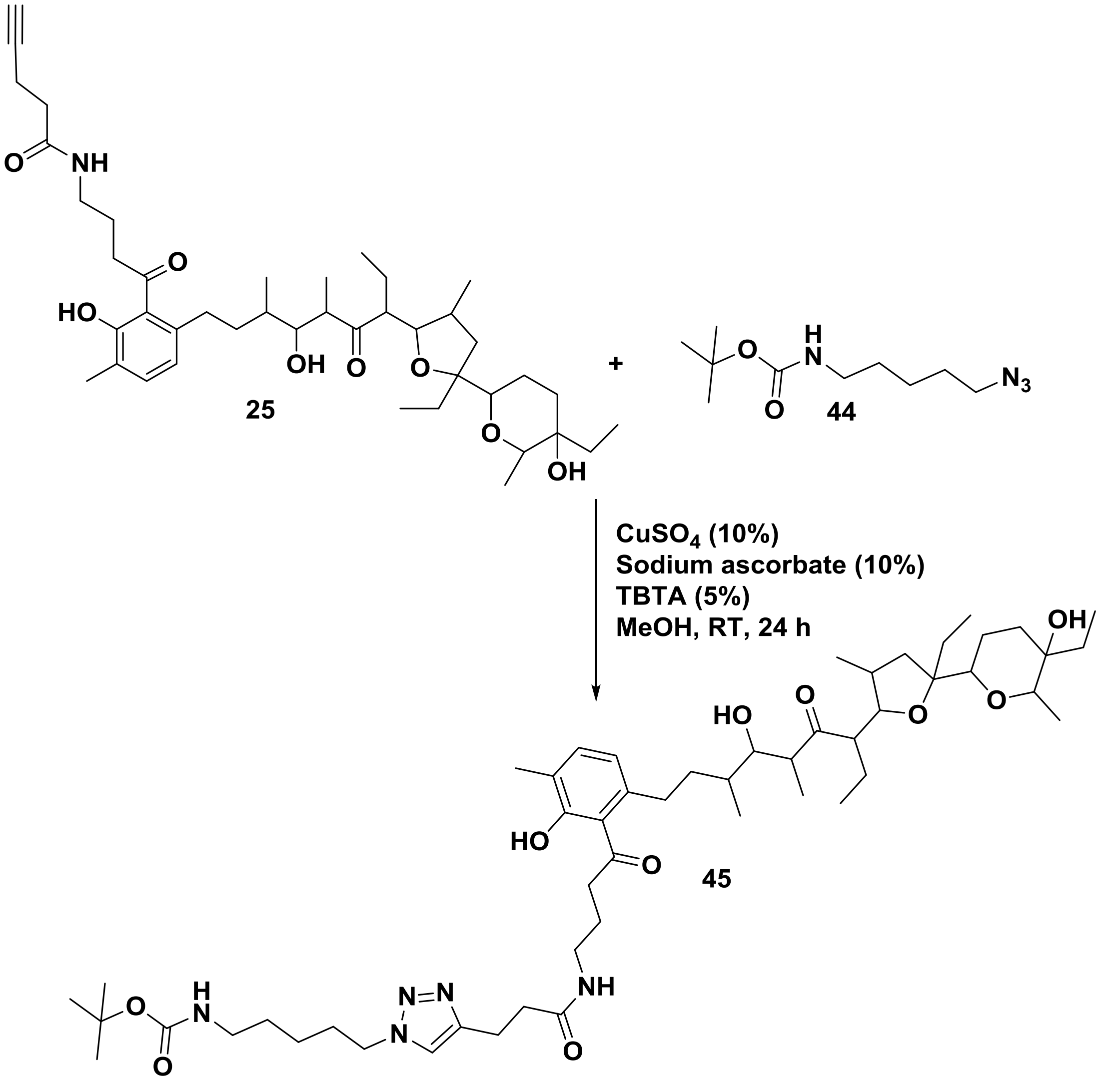

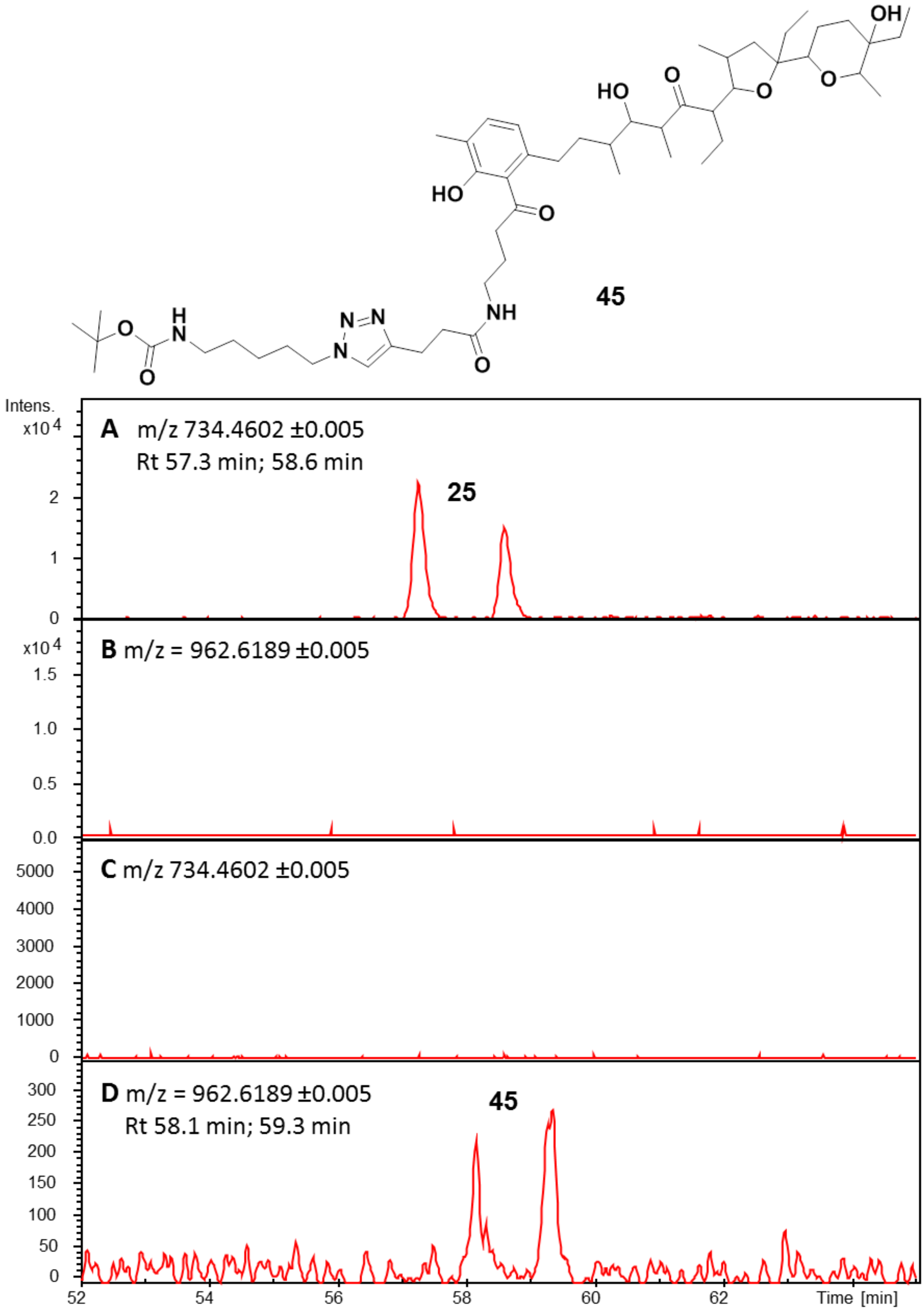

Figure 32: LC-ESI-HRMS analysis of the CuAAC of organic extracts containing alkyne intermediate **25** and azide **44** (Analysis Method 1). $[M+Na]^+$ extracted ion traces are shown. **A**) EIC of alkyne intermediates **25** used as starting material for the CuAAC. **D**) EIC of the triazole compound **45** derived from the CuAAC (not

present in the extracts containing the starting material **25**, **B**). No starting material could be detected in the reaction mixture after 24 h (**C**).

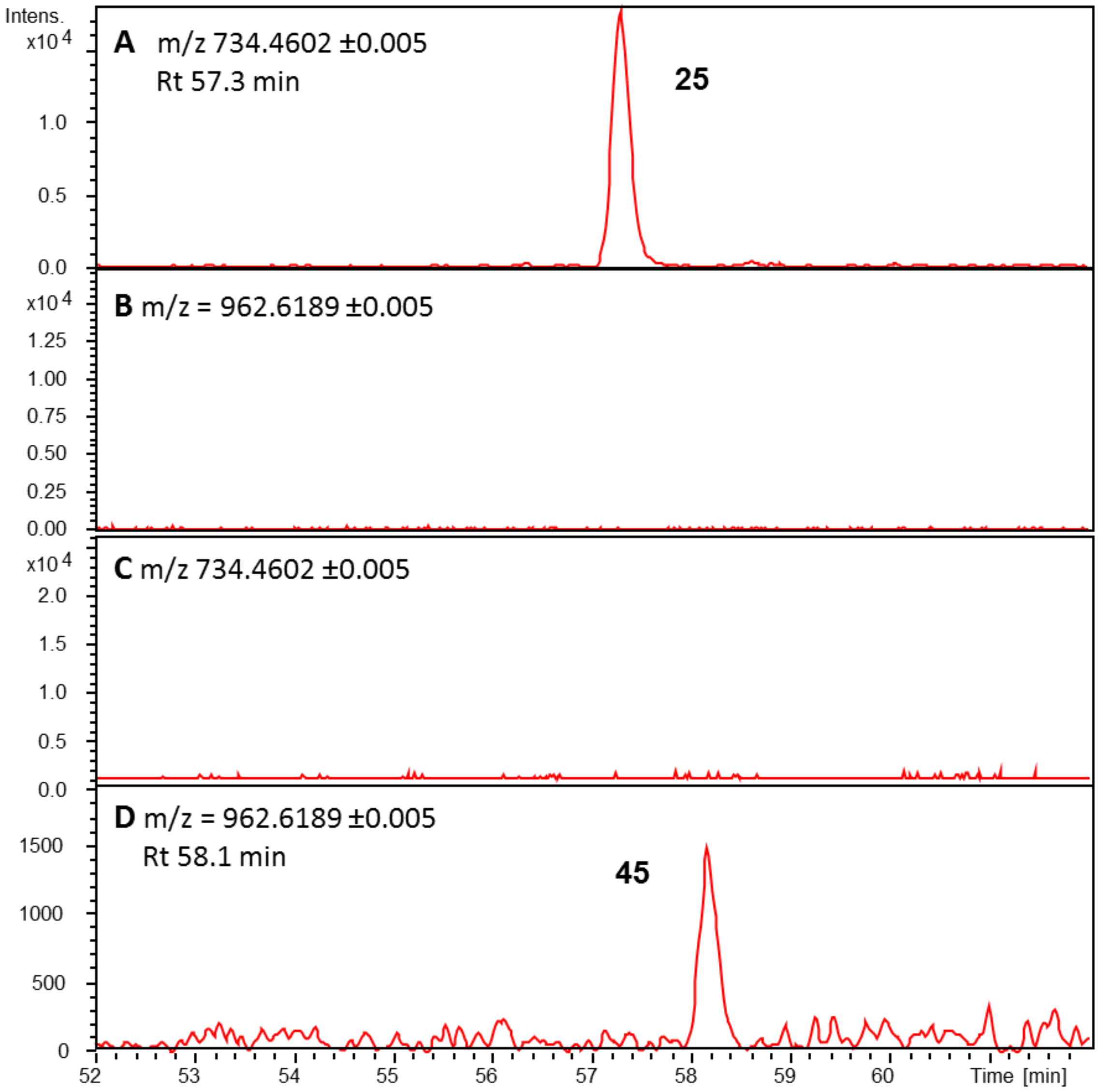

Figure 33: LC-ESI-HRMS analysis of the CuAAC of HPLC isolated organic extracts containing alkyne intermediate **25** (Fig. 3) and azide **44** (Analysis Method 1). $[M+Na]^+$ extracted ion traces are shown. **A**) EIC of HPLC purified alkyne intermediate **25** used as starting material for the CuAAC. **D**) EIC of the triazole compound **45** derived from the CuAAC (not present in the extracts containing the starting material **25**, **B**). No starting material could be detected in the reaction mixture after 24 h (**C**).

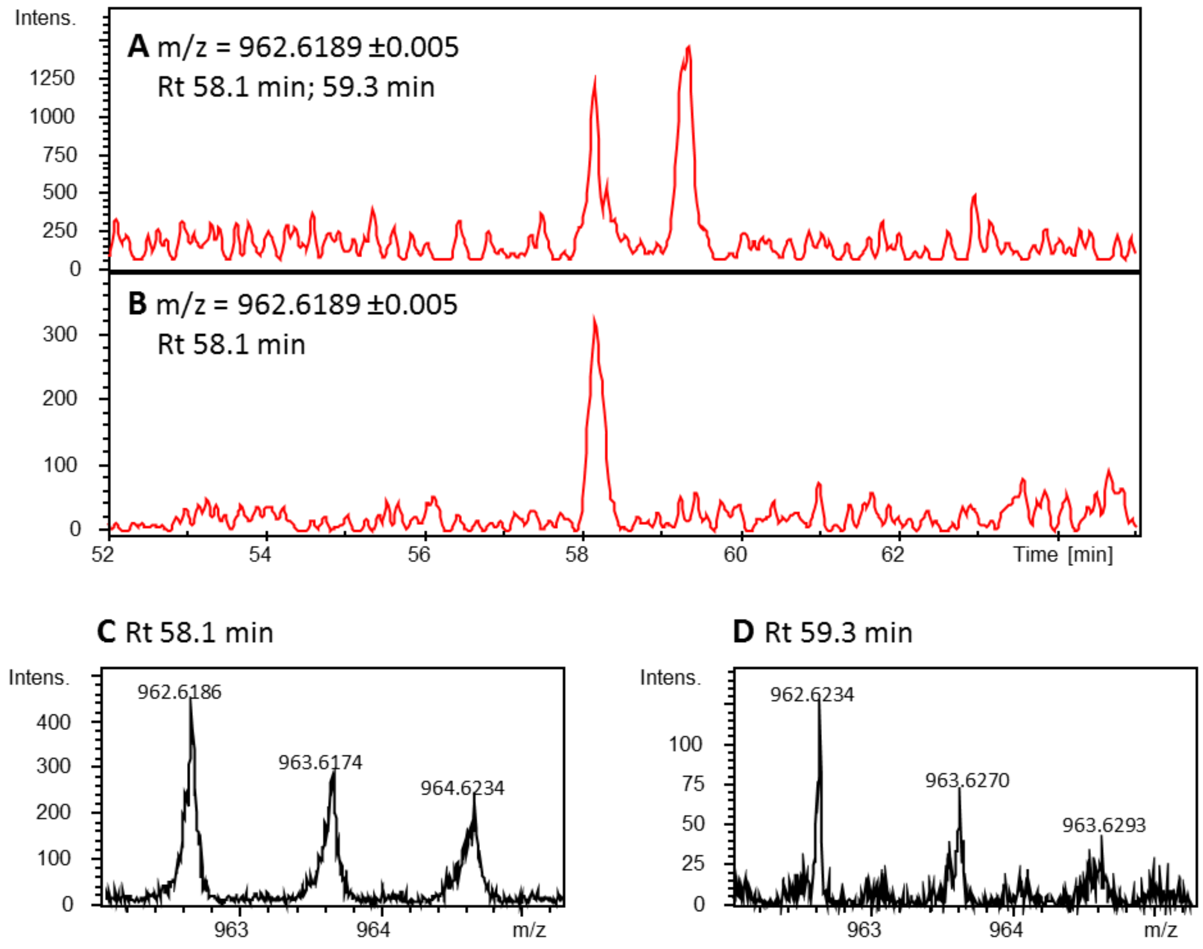

Figure 34: Comparison between EIC traces of products derived from CuAAC performed on crude and purified extracts containing intermediates **25** (**A** and **B**, respectively; Analysis Method 1). The high resolution masses and isotopic abundances for product **45** are reported in **C** and **D**.

4.2 Copper-catalyzed azide-alkyne cycloaddition (CuAAC) of alkyne intermediates 26 with alkyne 46

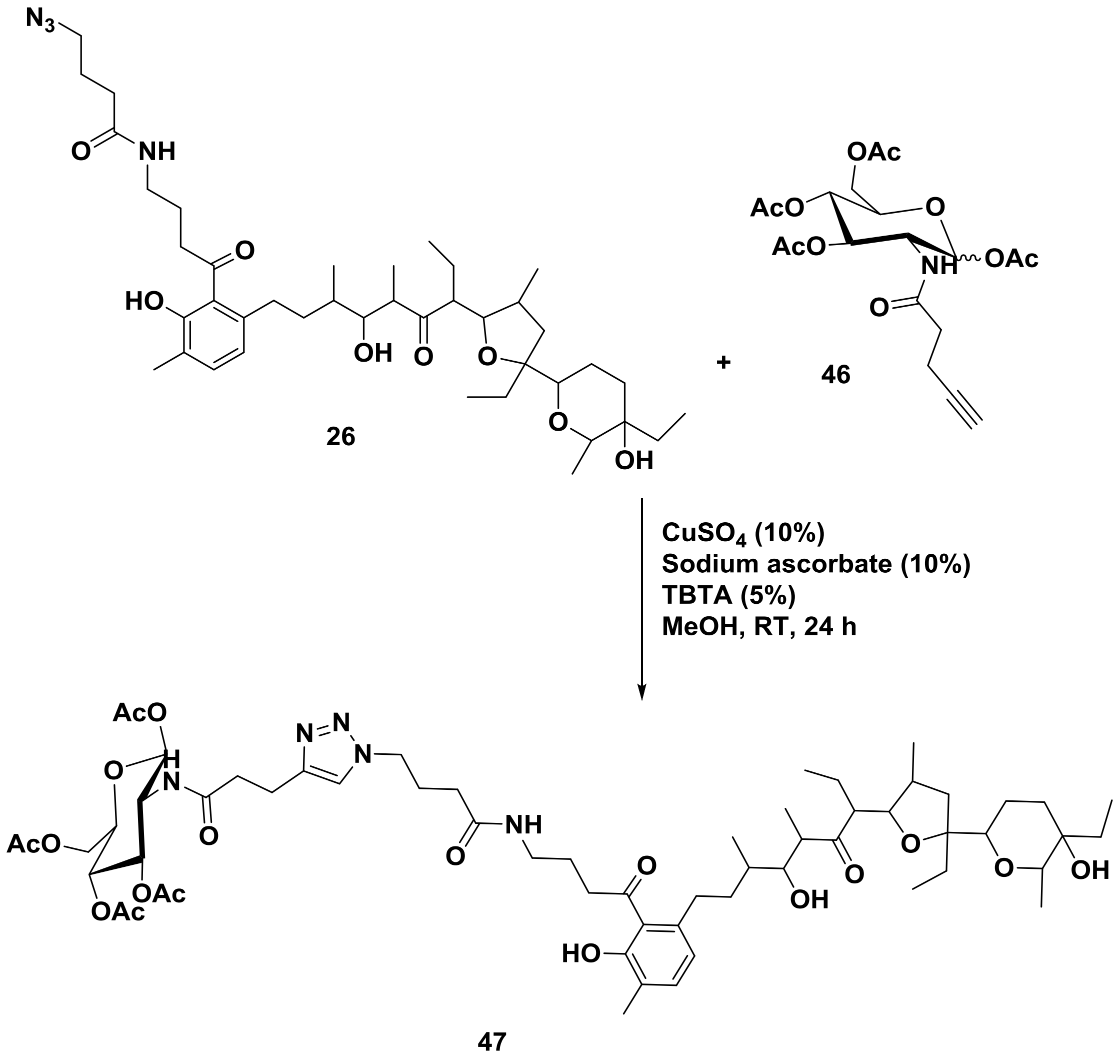

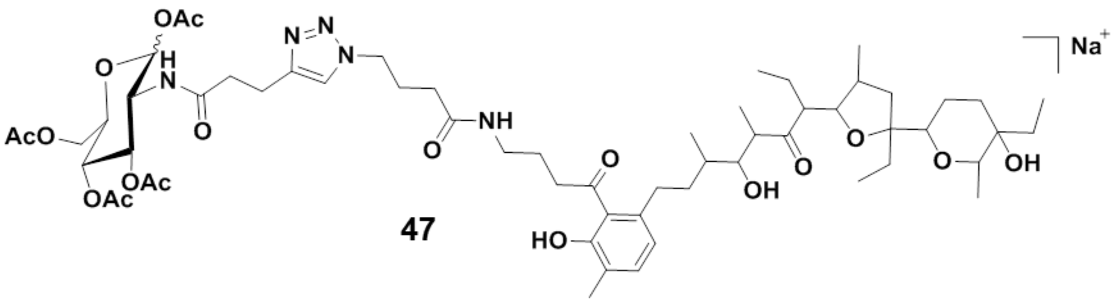

[M+Na]$^+$ calc.: m/z = 1192.6251

[M+H]$^+$ calc.: m/z = 1170.6432

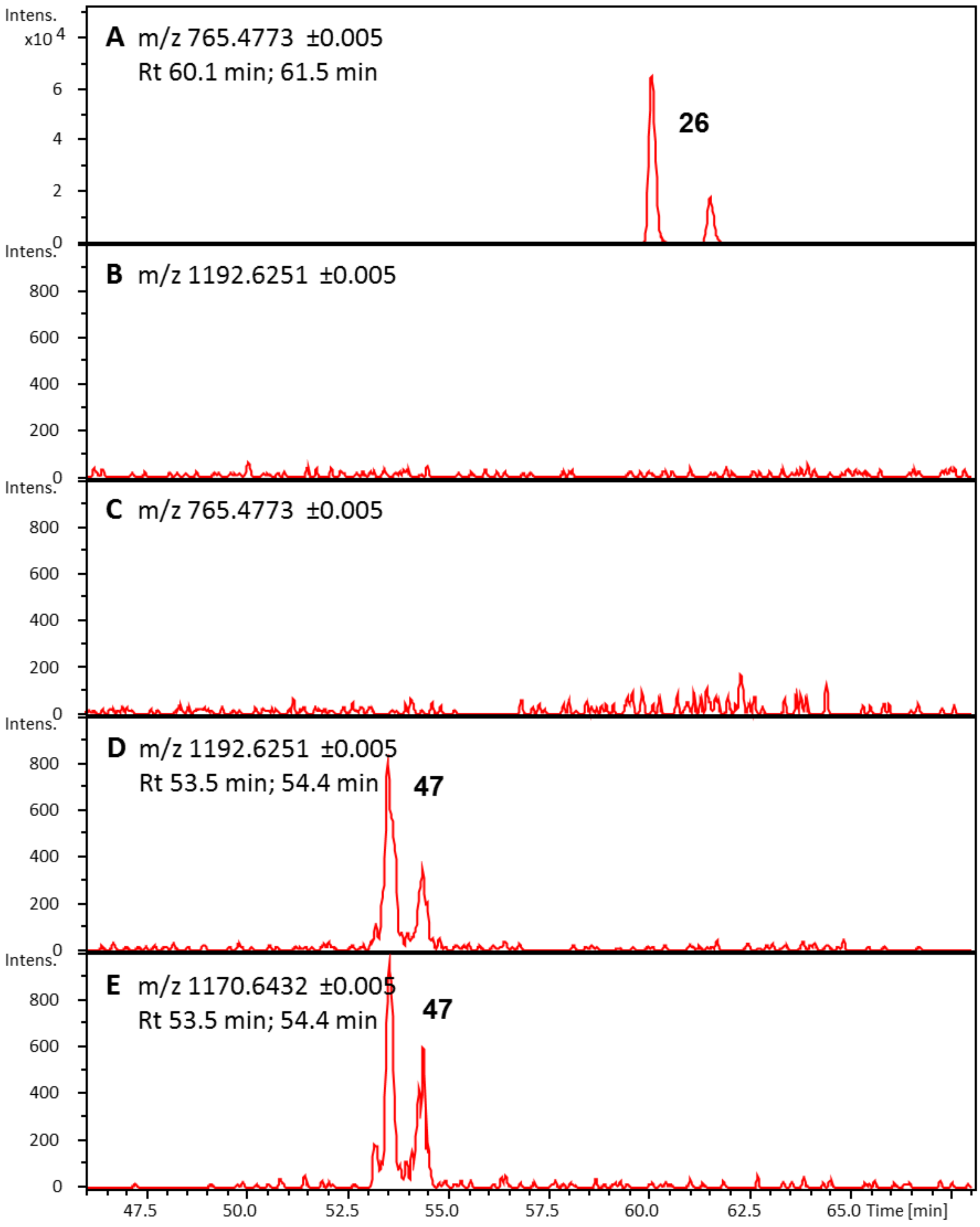

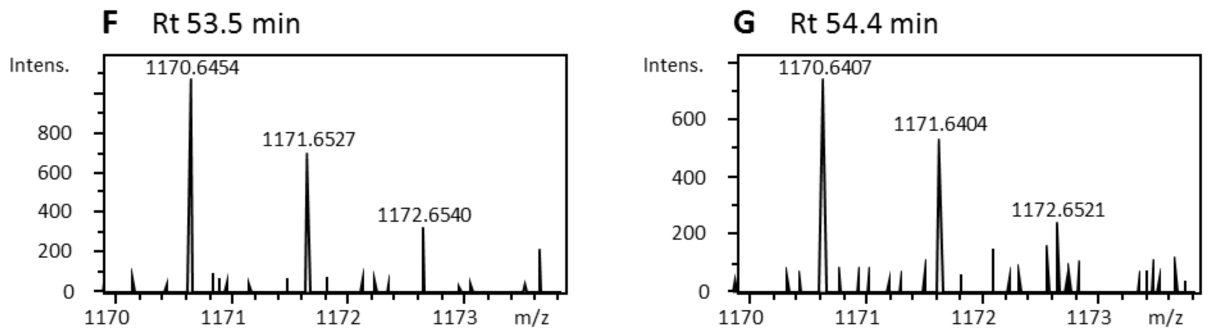

Figure 35: LC-ESI-HRMS analysis of the CuAAC of organic extracts containing azide intermediate **26** and alkyne **46** (Analysis Method 1). $[M+Na]^+$ and $[M+H]^+$ extracted ion traces are shown. **A**) EIC of azide intermediates **9b** used as starting material for the CuAAC. **D** and **E**) EIC ($[M+Na]^+$ and $[M+H]^+$) of the triazole compound **47** derived from the CuAAC (not present in the extracts containing the starting material **26**, **B**). No starting material could be detected in the reaction mixture after 24 h (**C**). The high resolution masses and isotopic abundances for product **47** are reported in **F** and **G**.

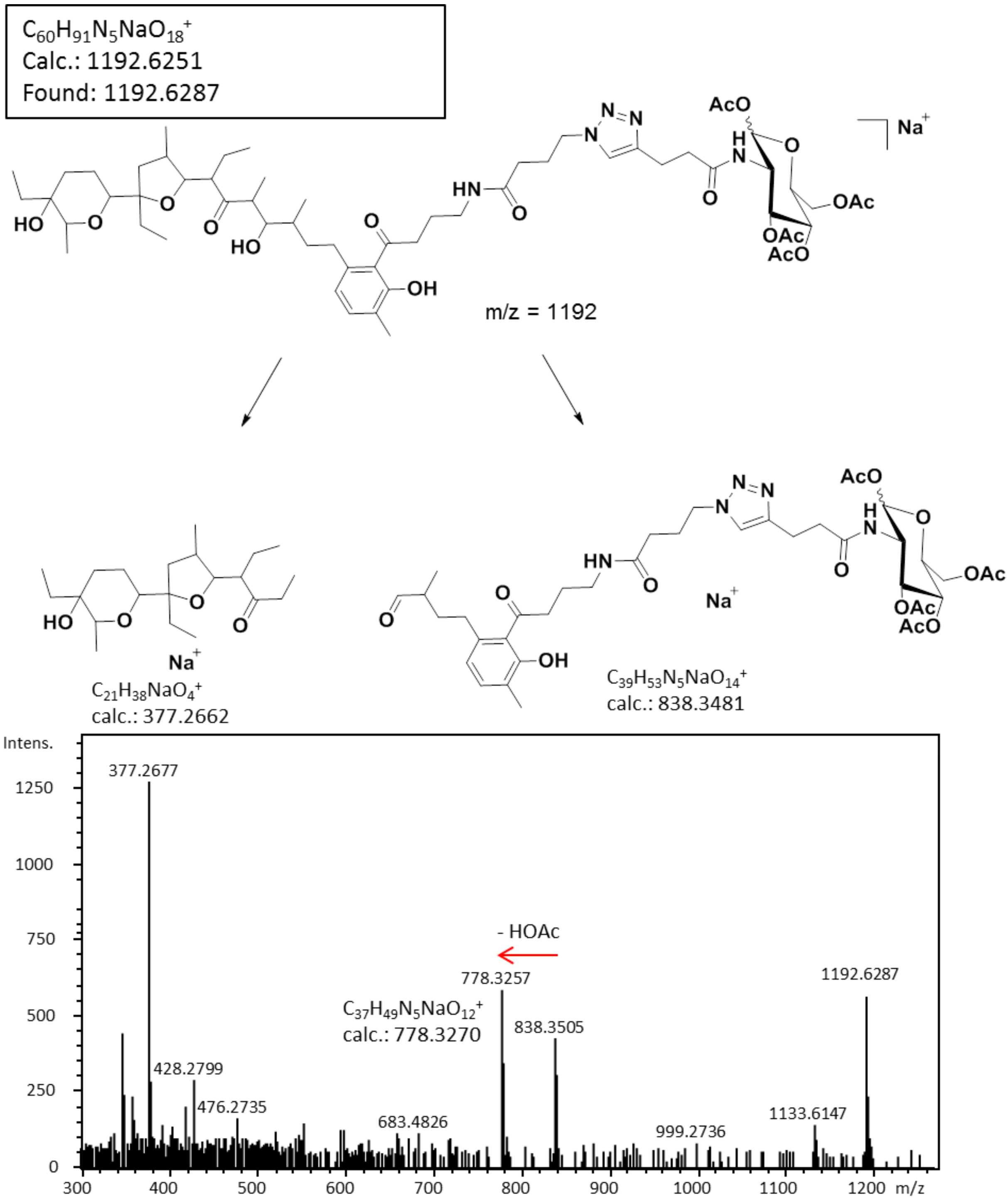

Figure 36: HR-ESI-MS^2 analysis of the phosphoramidate **47** (Analysis Method 3). McLafferty fragmentation of the $[M+Na]^+$ adduct generates a left–hand fragment of *m/z* 377, characteristic of lasalocid A, as well as a right-hand fragment containing the phosphoramidate moiety (*m/z* 838); further loss of acetic acid gives origin to *m/z* 778.

5 Staudinger-Phosphite reaction with intermediate 26

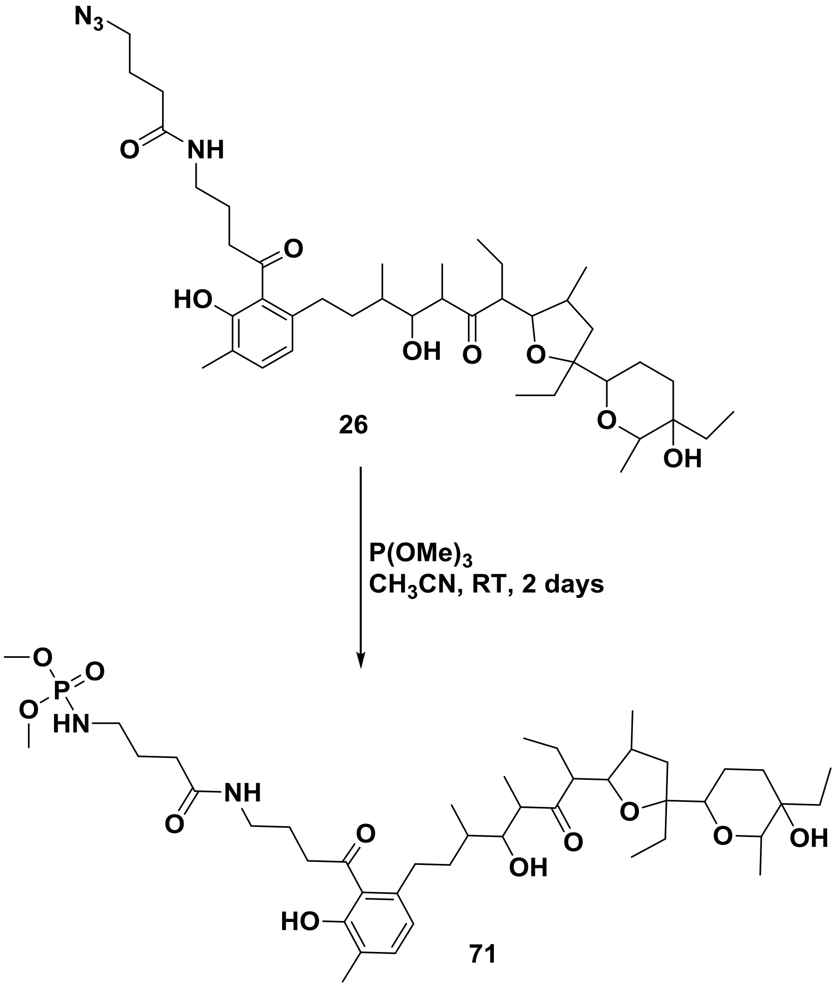

100 µL of the organic extracts containing intermediate **26** previously dissolved in 1 mL HPLC-grade MeOH were concentrated under reduced pressure and the residue was dissolved in 200 µL acetonitrile. Trimethyl phosphite (0.30 mmol, 37.2 mg, 100 eq, equivalents were calculated based on fed amount of probe **6**). The reaction mixture was stirred for two days at room. Afterwards the reaction mixture was concentrated *in vacuo* and the residue was dissolved in 100 µL MeOH and analyzed by LC-HRMS.

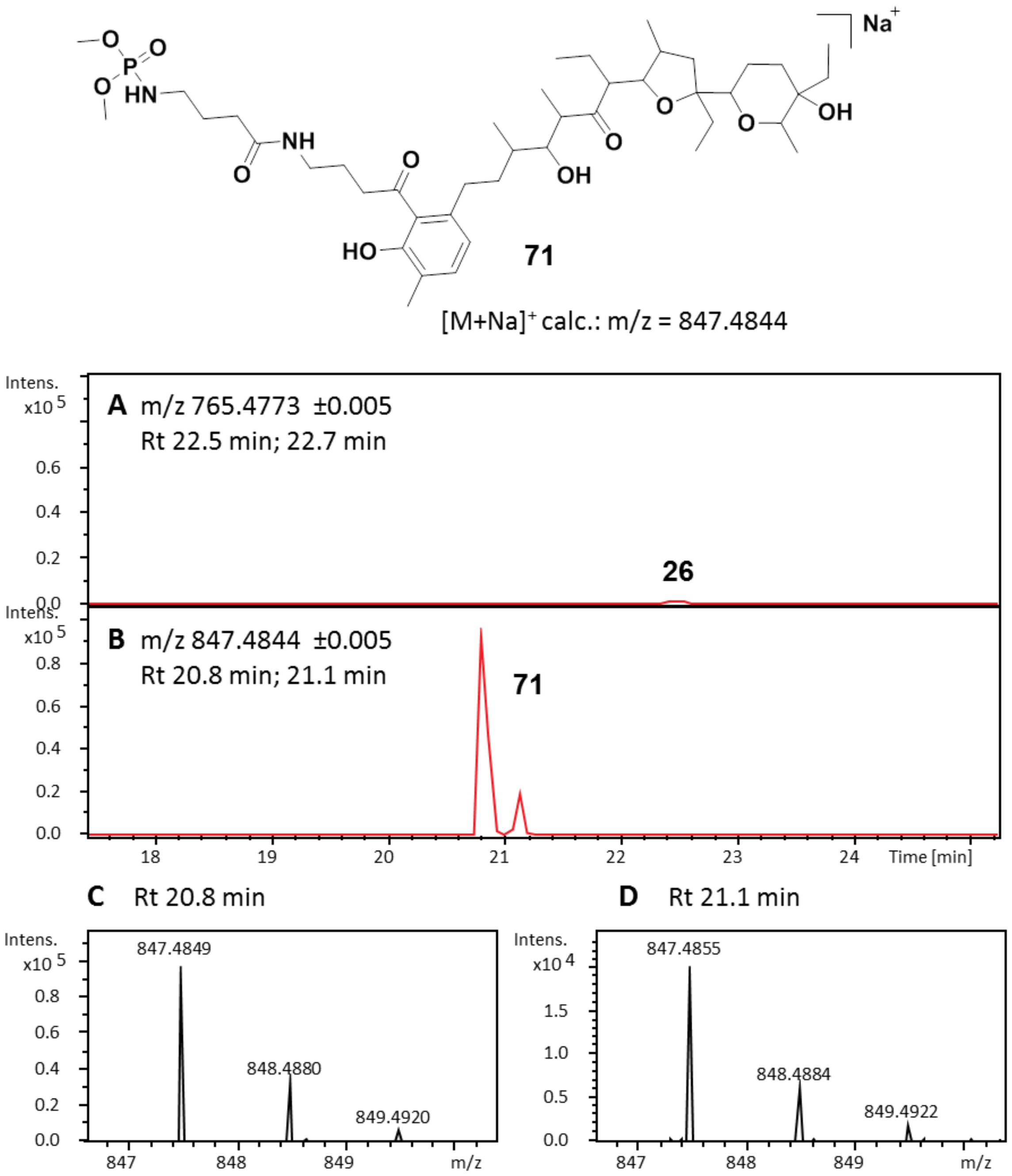

Figure 37: LC-ESI-HRMS analysis of the Staudinger-phosphite reaction of organic extracts containing azide intermediate **26** and trimethyl phosphite. $[M+Na]^+$ extracted ion traces are shown (Analysis Method 3). **B**) EIC ($[M+Na]^+$ of the phosphonamidate compound **71** derived from the Staudinger-phosphite reaction (not present in the extracts containing the starting material **26**. Traces of unreacted starting material **26** could still be detected after the reaction (**B**). The high resolution masses and isotopic abundances for product **71** are reported in **C** and **D**.

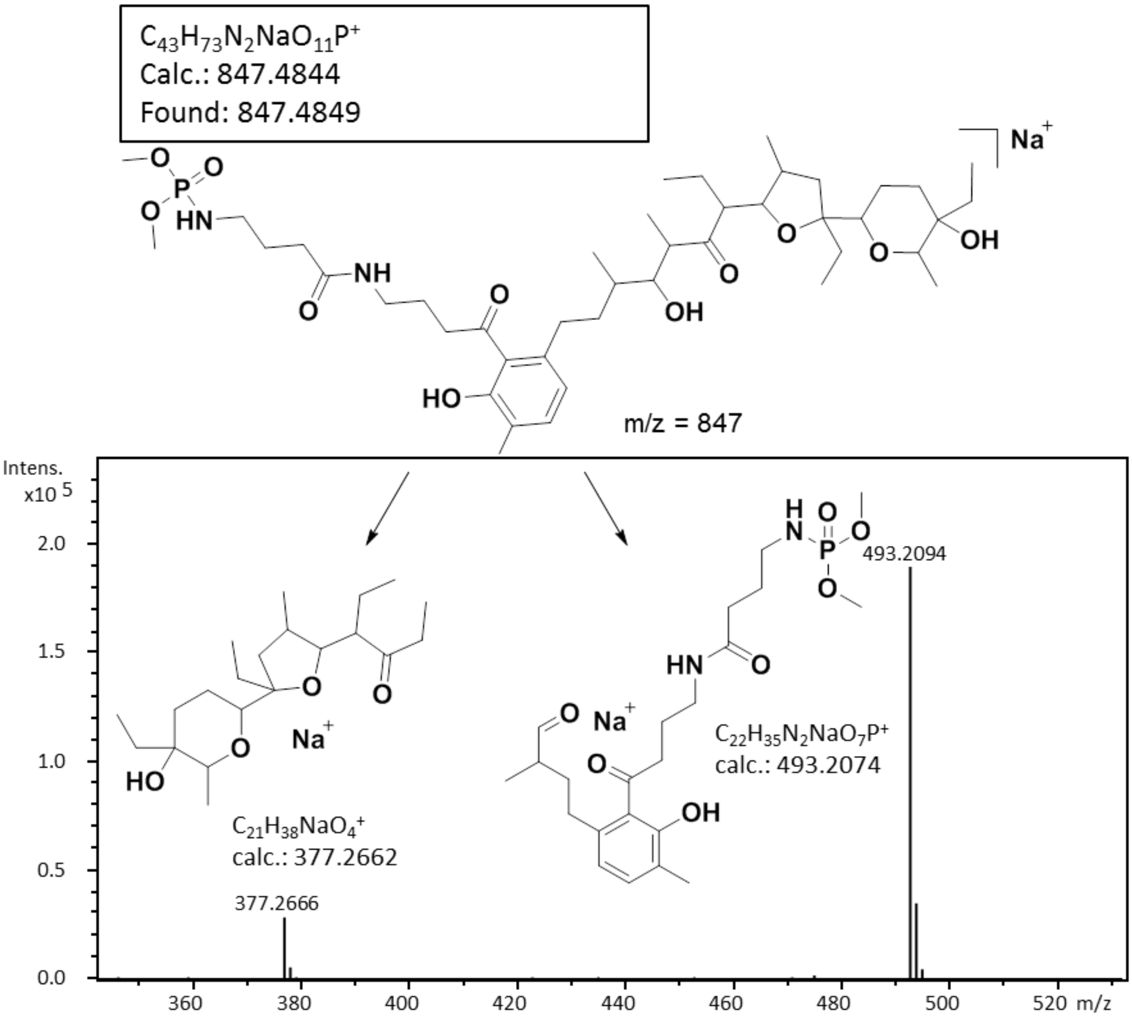

Figure 38: HR-ESI-MS[2] analysis of the phosphoramidate **71** (Analysis Method 3). McLafferty fragmentation of the $[M+Na]^+$ adduct generates a left–hand fragment of *m/z* 377, characteristic of lasalocid A, as well as a right-hand fragment containing the phosphoramidate moiety (*m/z* 493).

6 Staudinger-Phosphite reaction with intermediate 28

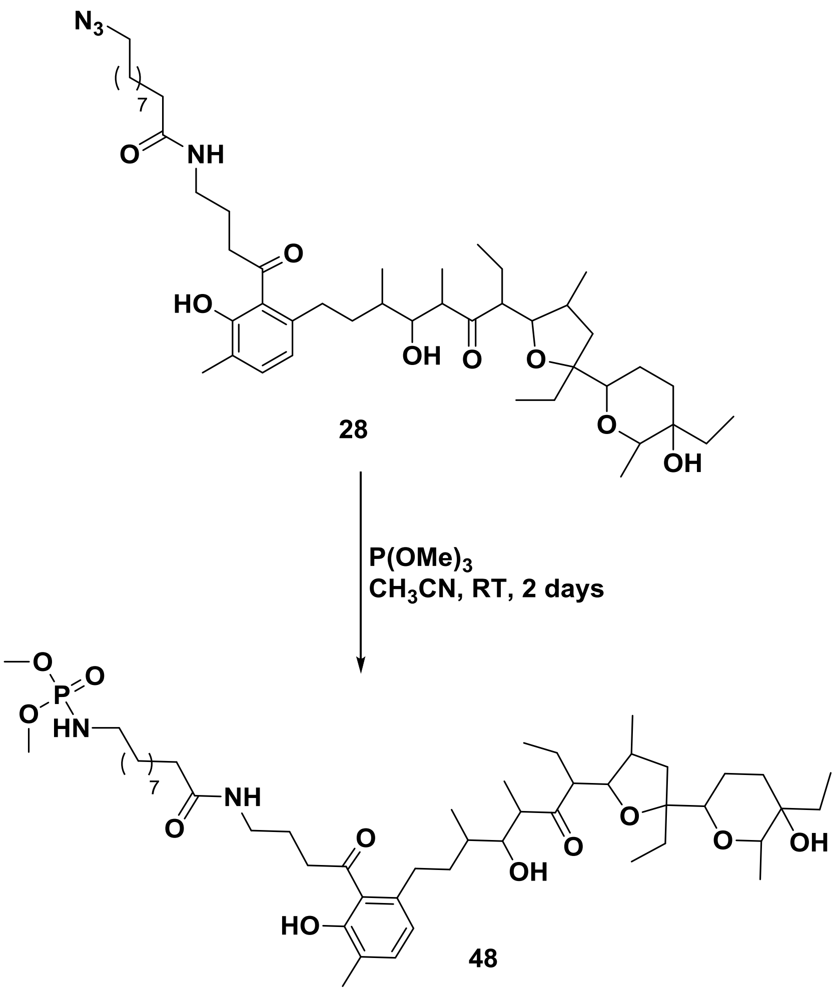

100 µL of the organic extracts containing intermediate **28** previously dissolved in 1 mL HPLC-grade MeOH were concentrated under reduced pressure and the residue was dissolved in 200 µL acetonitrile. Trimethyl phosphite (0.30 mmol, 37.2 mg, 100 eq, equivalents were calculated based on fed amount of probe **8**). The reaction mixture was stirred for two days at room. Afterwards the reaction mixture was concentrated *in vacuo* and the residue was dissolved in 100 µL MeOH and analyzed by LC-HRMS.

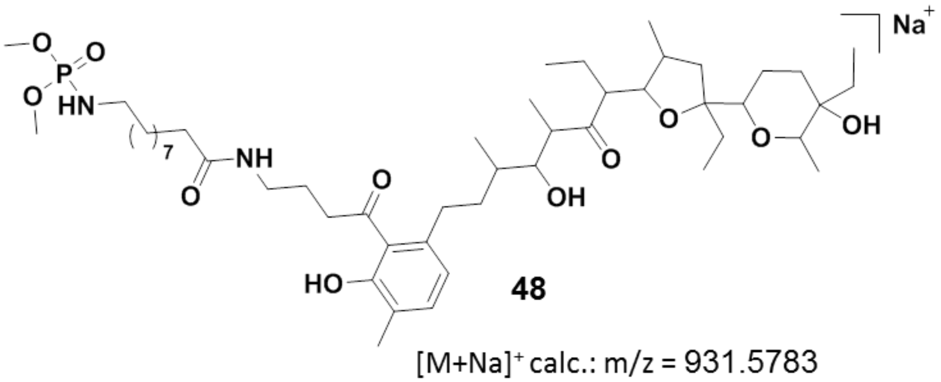

[M+Na]$^+$ calc.: m/z = 931.5783

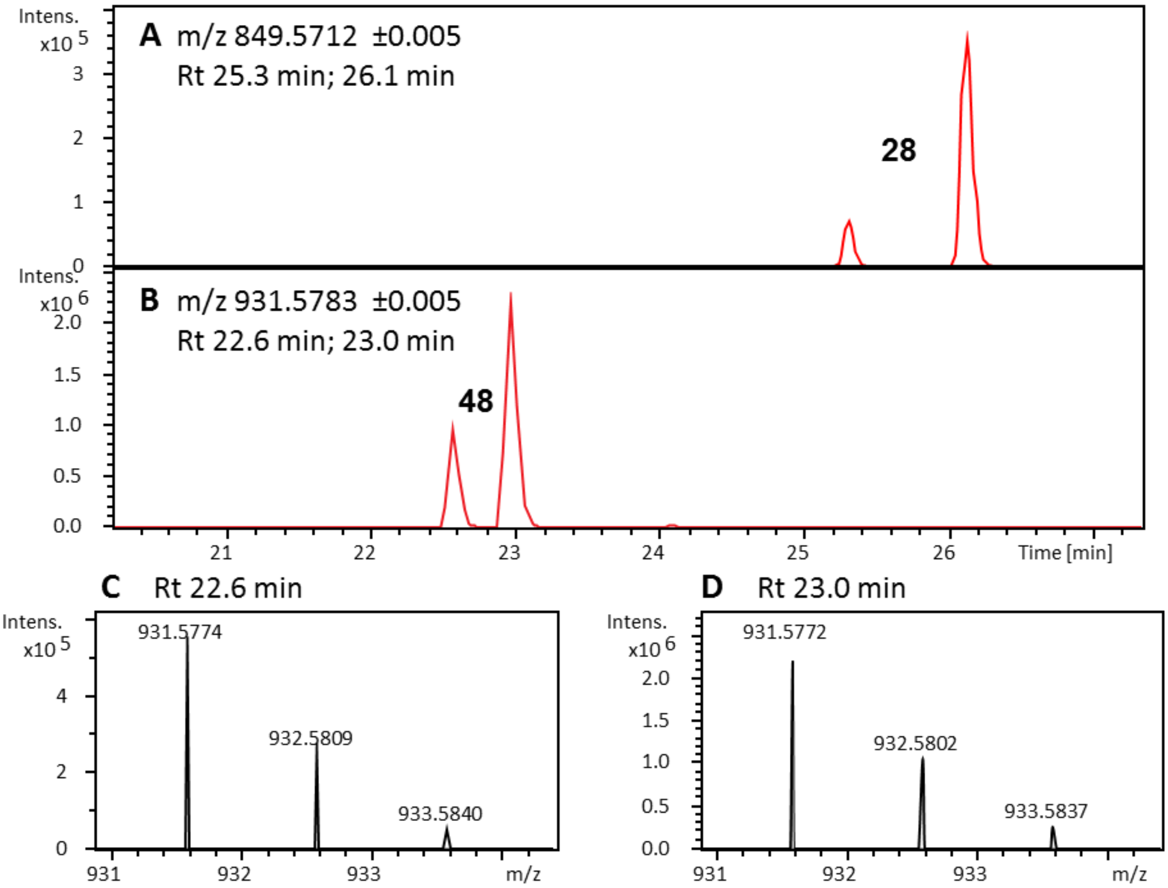

Figure 39: LC-ESI-HRMS analysis of the Staudinger-phosphite reaction of organic extracts containing azide intermediate **28** and trimethyl phosphite. [M+Na]$^+$ extracted ion traces is shown (Analysis Method 3). **B**) EIC ([M+Na]$^+$ of the phosphonamidate compound **48** derived from the Staudinger-phosphite reaction (not present in the extracts containing the starting material **28**. Traces of unreacted starting material **28** could still be detected after the reaction (**B**). The high resolution masses and isotopic abundances for product **48** are reported in **C** and **D**.

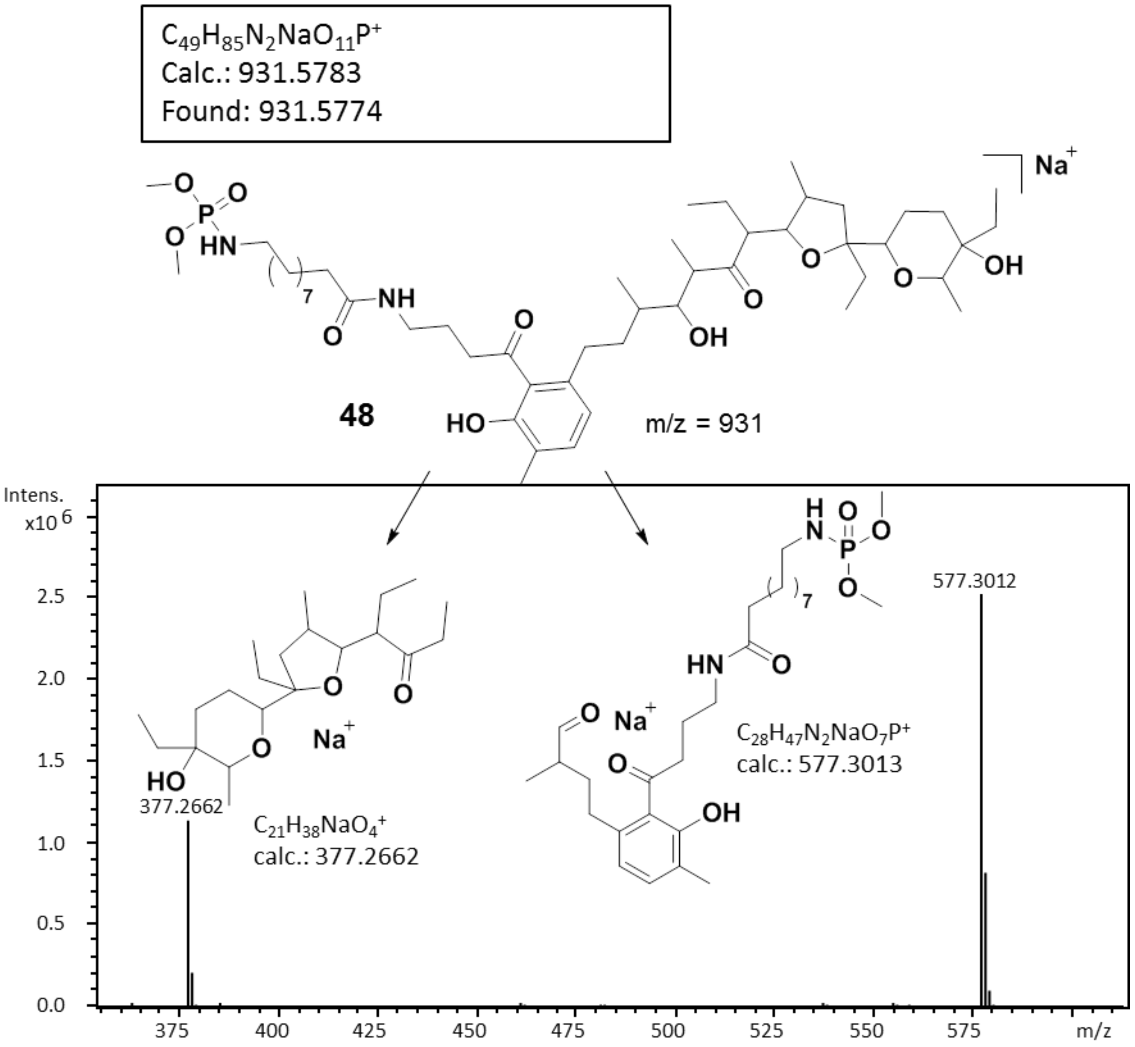

Figure 40 HR-ESI-MS^2 analysis of the phosphoramidate **48** (Analysis Method 3). McLafferty fragmentation of the $[M+Na]^+$ adduct generates a left–hand fragment of *m/z* 377, characteristic of lasalocid A, as well as a right-hand fragment containing the phosphoramidate moiety (*m/z* 577).

Literature:

[1] M. Tosin, L. Betancor, E. Stephens, W. M. Li, J. B. Spencer, P. F. Leadlay, *ChemBioChem* **2010**, *11*, 539-546.

[2] M. Tosin, Y. Demydchuk, J. S. Parascandolo, C. B. Per, F. J. Leeper, P. F. Leadlay, *Chem. Commun.* **2011**, *47*, 3460-3462.

[3] J. Deblander, S. Van Aeken, J. Jacobs, N. De Kimpe, K. Abbaspour Tehrani, *Eur. J. Org. Chem.* **2009**, *2009*, 4882-4892.

[4] R. Frantz, L. Hintermann, M. Perseghini, D. Broggini, A. Togni, *Org. Lett.* **2003**, *5*, 1709-1712.

[5] S. G. Alvarez, M. T. Alvarez, *Synthesis* **1997**, *1997*, 413-414.

[6] C. A. DeForest, K. S. Anseth, *Nat. Chem.* **2011**, *3*, 925-931.

[7] T. L. Mindt, C. Muller, M. Melis, M. de Jong, R. Schibli, *Bioconjugate Chem.* **2008**, *19*, 1689-1695.

[8] F. Kaschani, J. Clerc, D. Krahn, D. Bier, T. N. Hong, C. Ottmann, S. Niessen, T. Colby, R. A. L. van der Hoorn, M. Kaiser, *Angew. Chem. Int. Ed.* **2012**, *51*, 5230-5233.
[9] A. Horatscheck, S. Wagner, J. Ortwein, B. G. Kim, M. Lisurek, S. Beligny, A. Schütz, J. Rademann, *Angew. Chem. Int. Ed.* **2012**, *51*, 9441-9447.
[10] D. C. Kennedy, C. S. McKay, M. C. B. Legault, D. C. Danielson, J. A. Blake, A. F. Pegoraro, A. Stolow, Z. Mester, J. P. Pezacki, *J. Am. Chem. Soc.* **2011**, *133*, 17993-18001.
[11] M. A. Breidenbach, J. E. G. Gallagher, D. S. King, B. P. Smart, P. Wu, C. R. Bertozzi, *Proc. Natl. Acad. Sci.* **2010**, *107*, 3988-3993.
[12] M. Tosin, L. Smith, P. F. Leadlay, *Angew. Chem. Int. Ed. Engl.* **2011**, *50*, 11930-11933.

7 NMR Spectra

7.1 ^{1}H-, ^{13}C- and ^{19}F-NMR of compound 51

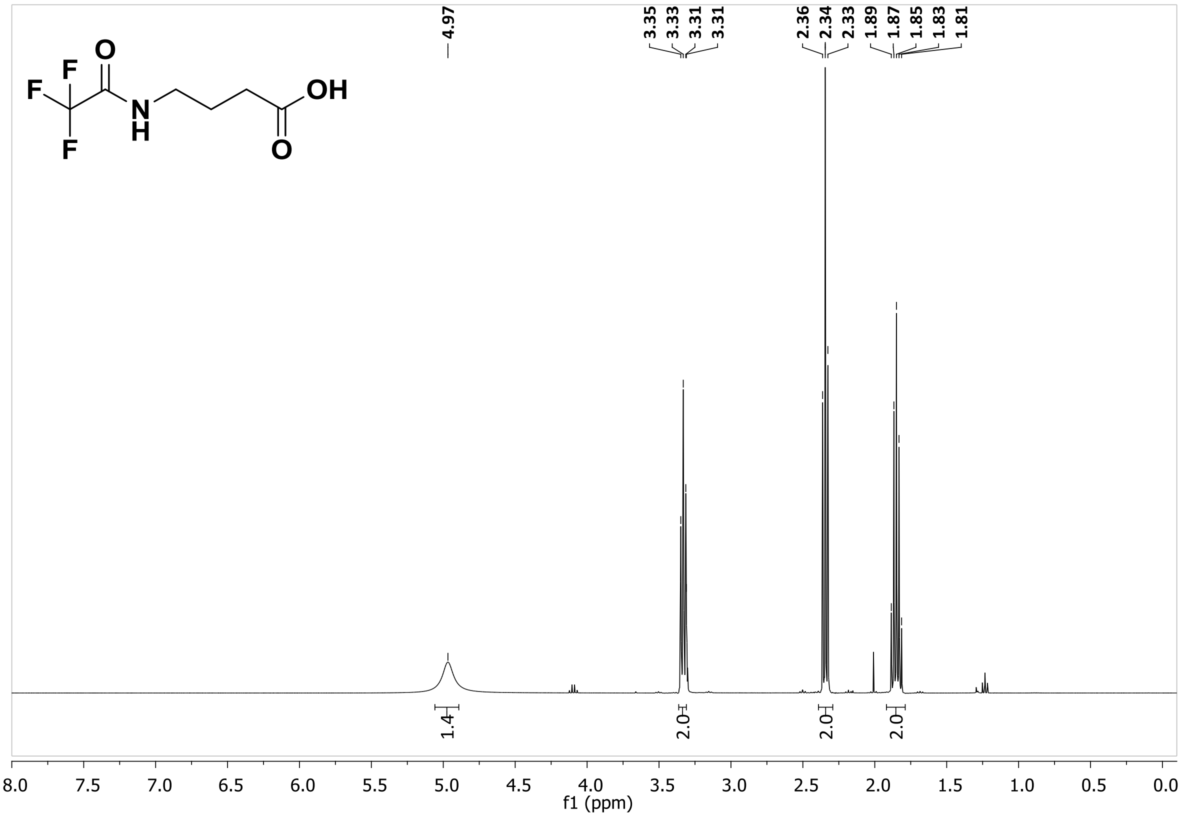

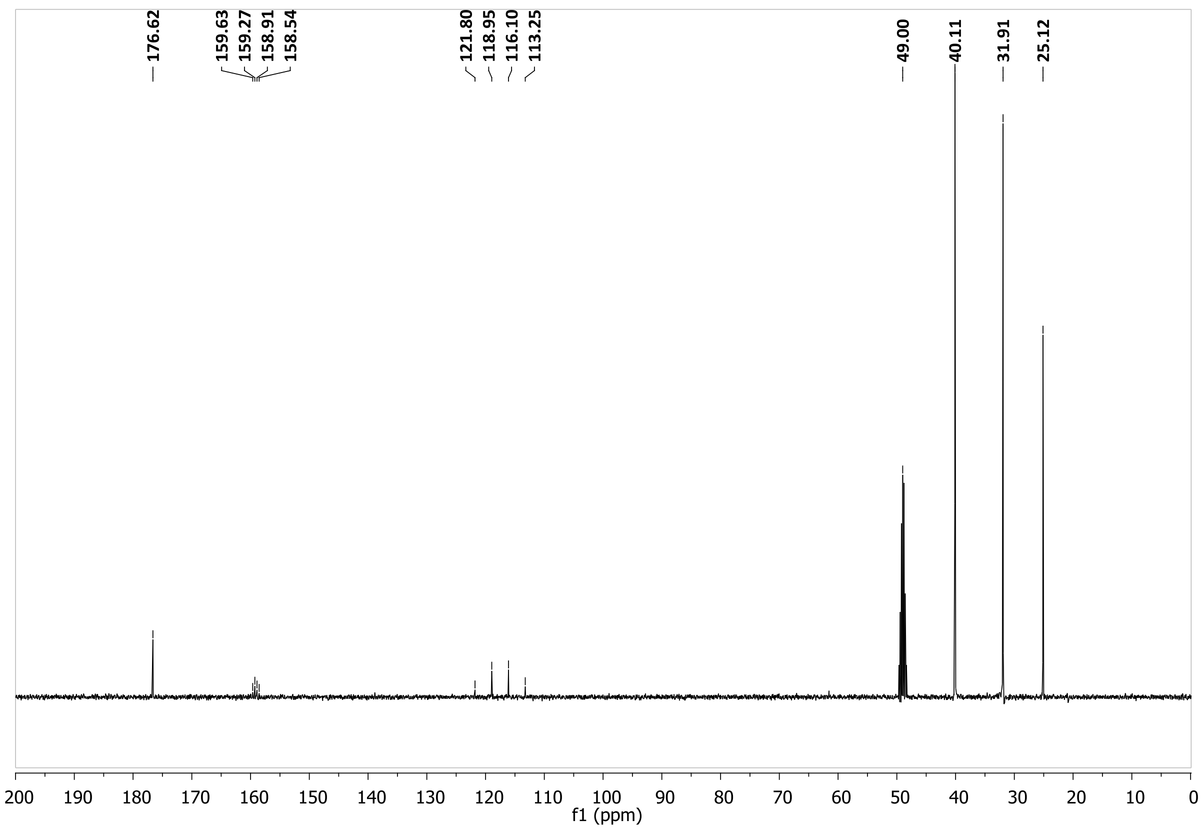
176.62
159.63
159.27
158.91
158.54
121.80
118.95
116.10
113.25
49.00
40.11
31.91
25.12
f1 (ppm)

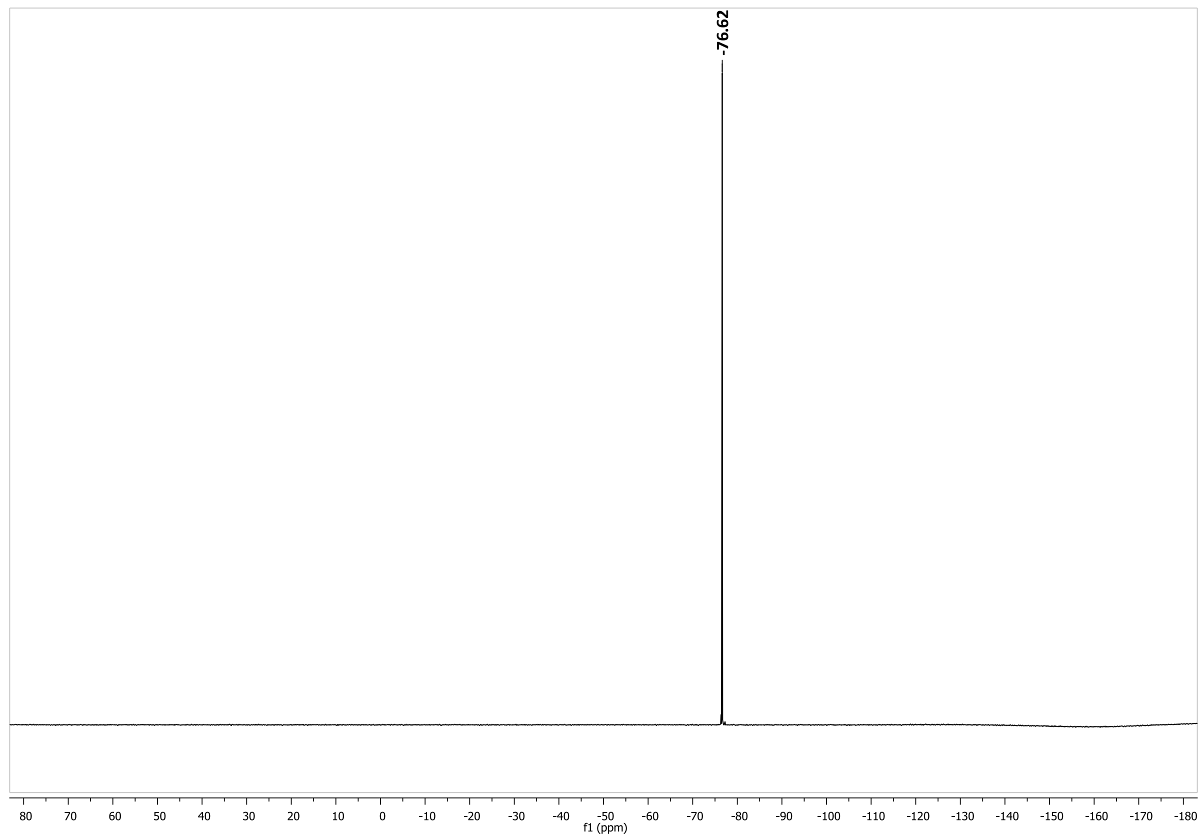
-76.62
f1 (ppm)

7.2 ^{1}H-, ^{13}C- and ^{19}F-NMR of compound 52

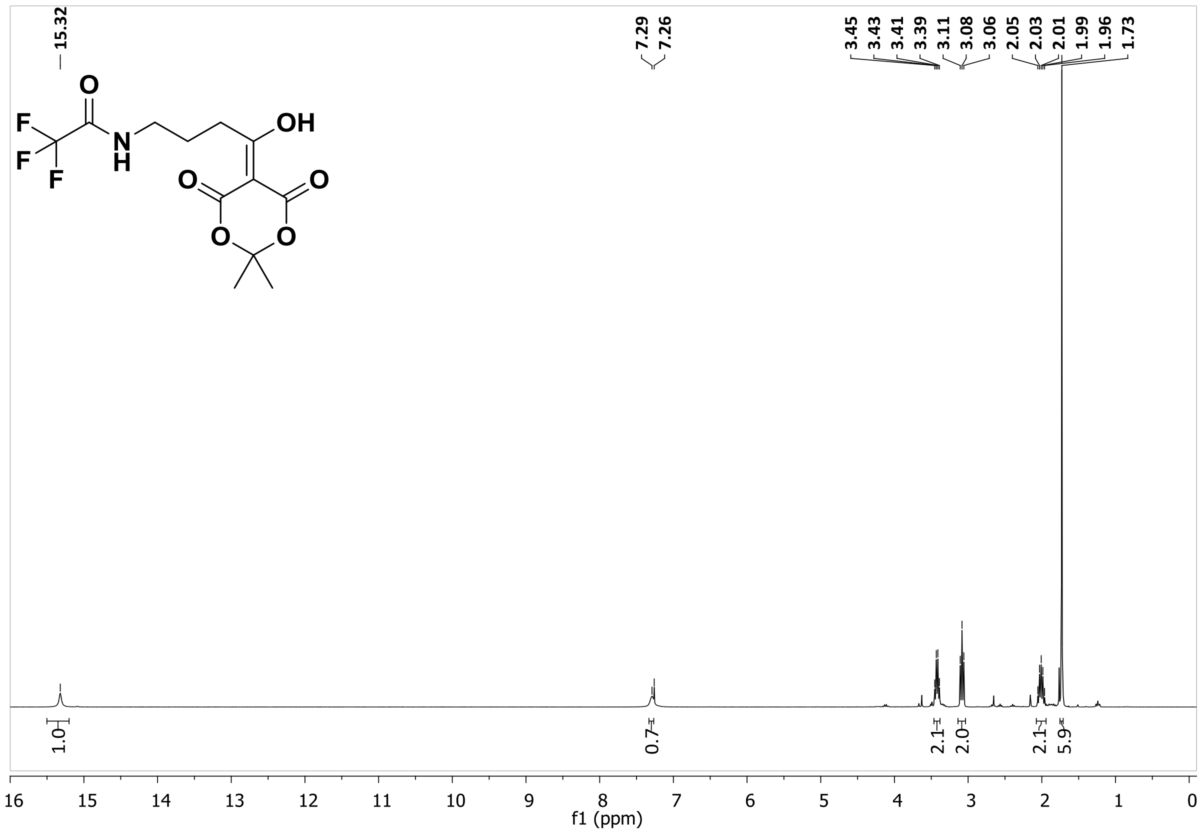

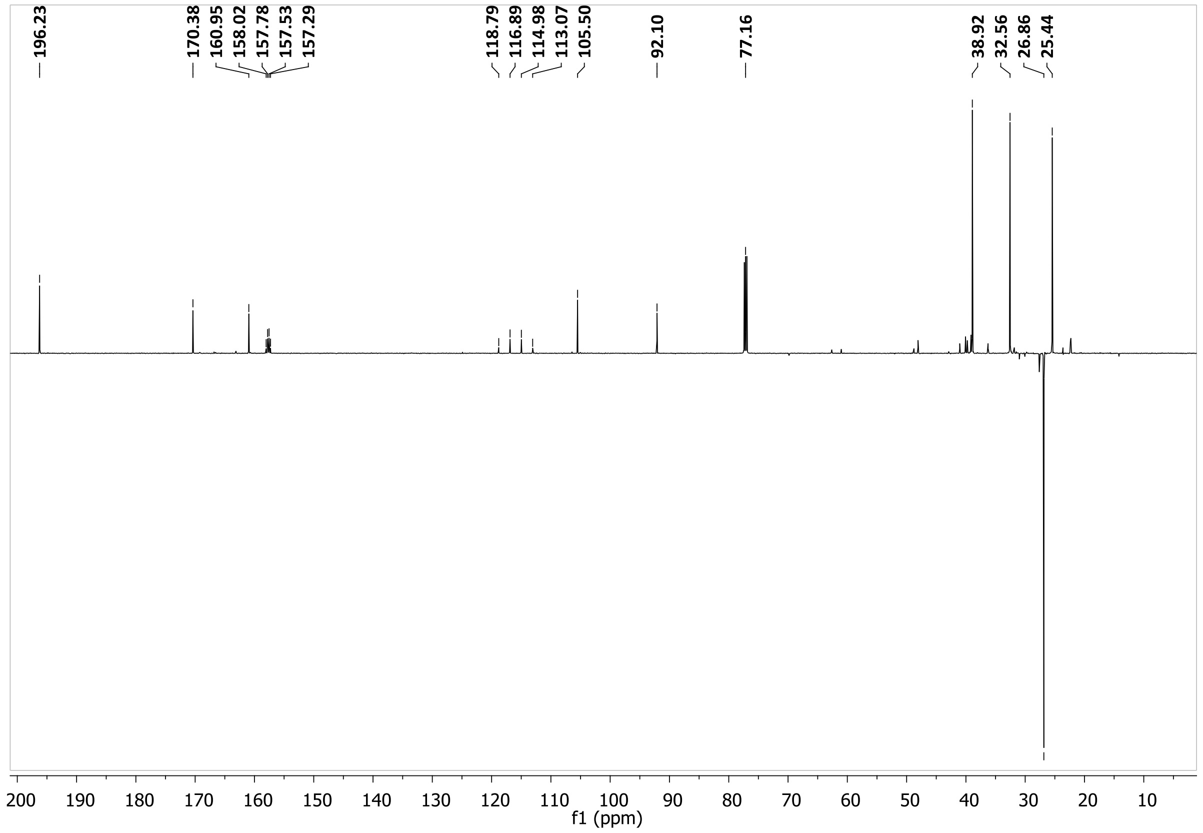

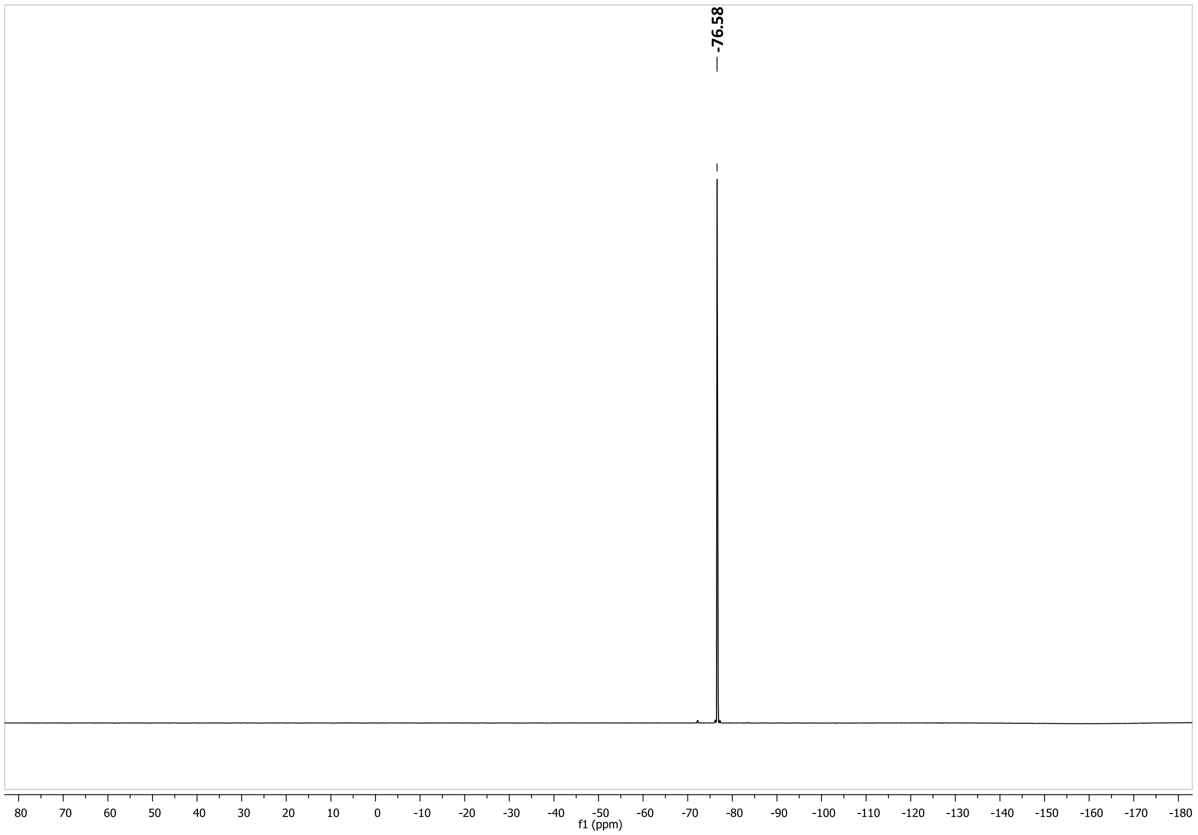

7.3 ^{1}H-, ^{13}C- and ^{19}F-NMR of compound 4

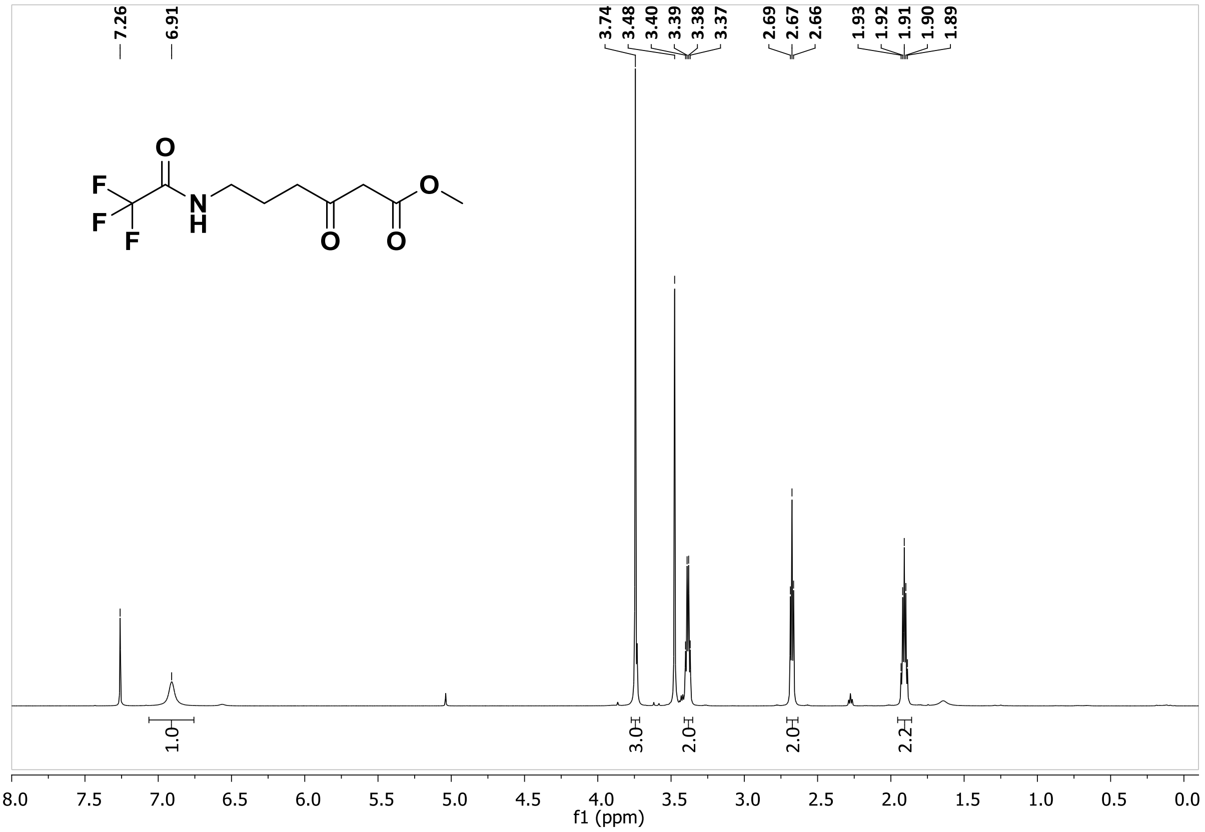

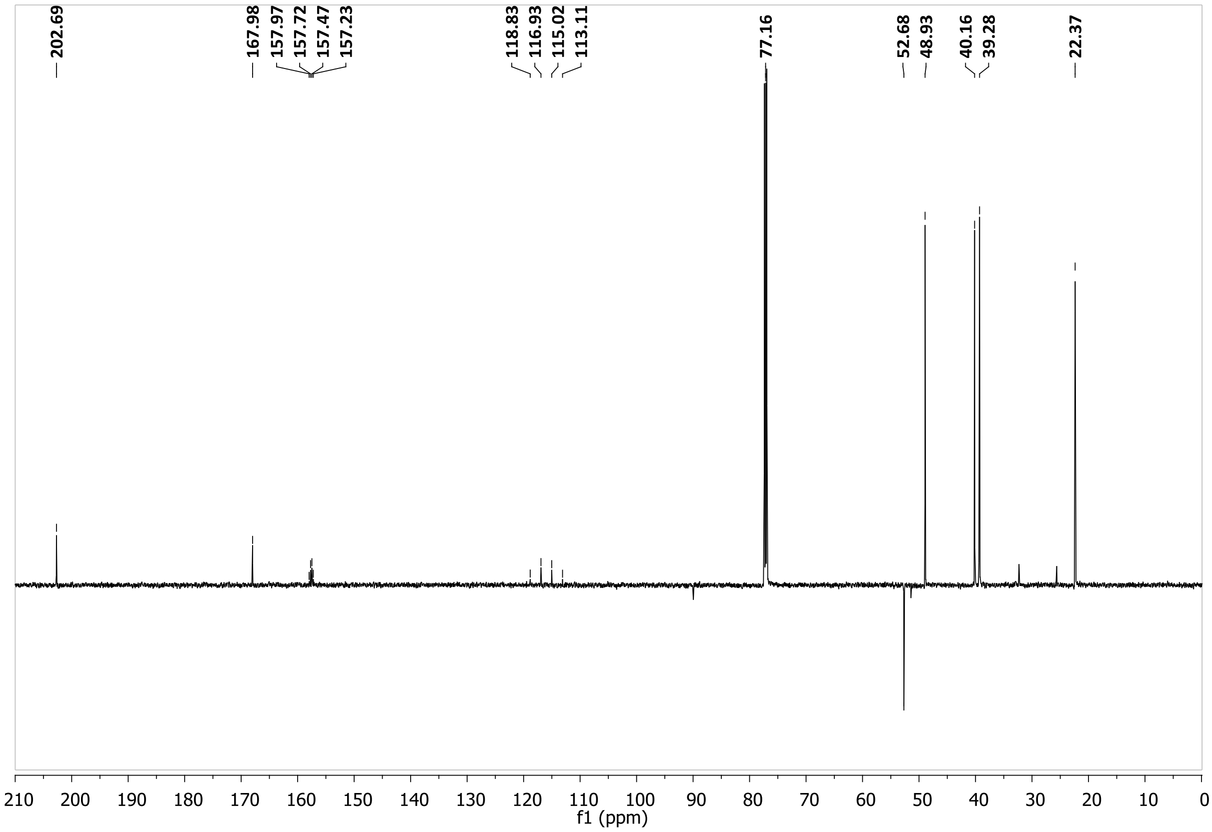
202.69
167.98
157.97
157.72
157.47
157.23
118.83
116.93
115.02
113.11
77.16
52.68
48.93
40.16
39.28
22.37
210 200 190 180 170 160 150 140 130 120 110 100 90 80 70 60 50 40 30 20 10 0
f1 (ppm)

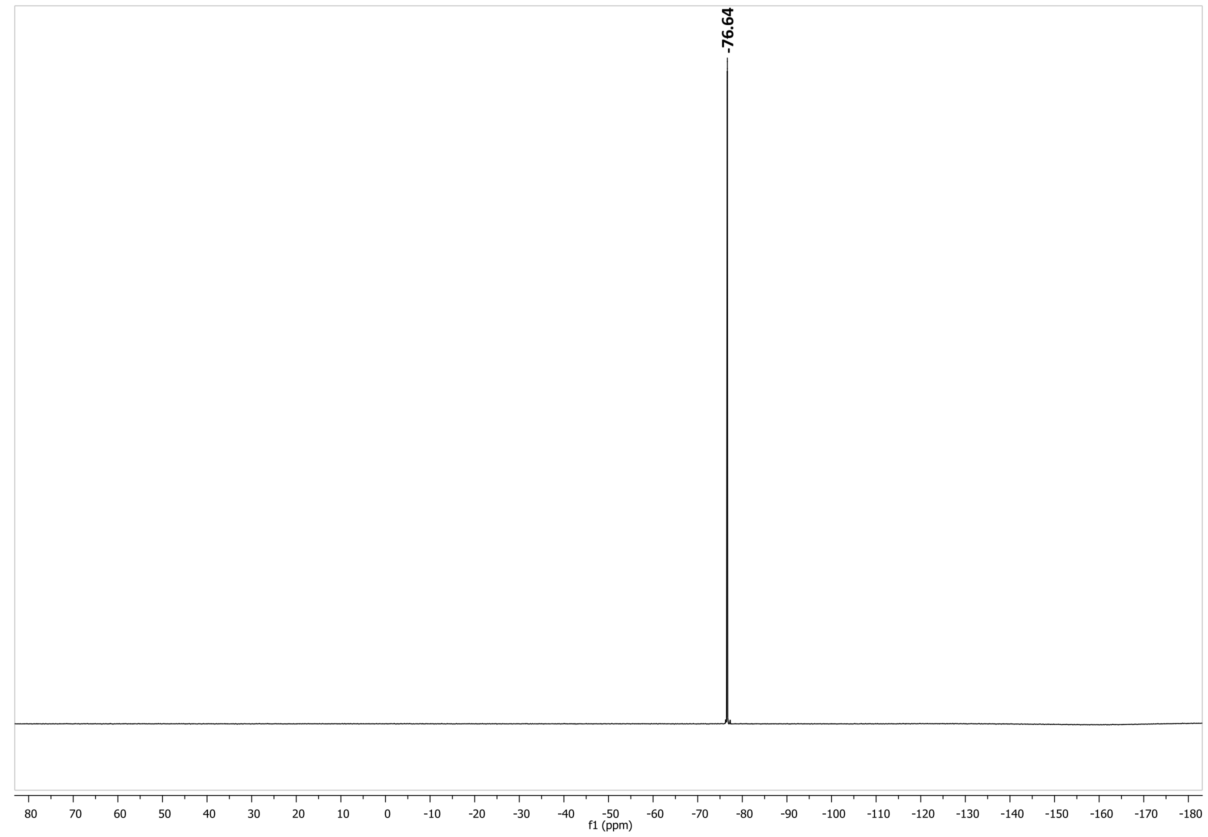
-76.64
80 70 60 50 40 30 20 10 0 -10 -20 -30 -40 -50 -60 -70 -80 -90 -100 -110 -120 -130 -140 -150 -160 -170 -180
f1 (ppm)

7.4 ^{1}H- and ^{13}C-NMR of compound 55

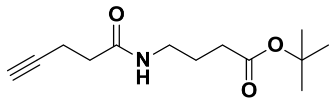

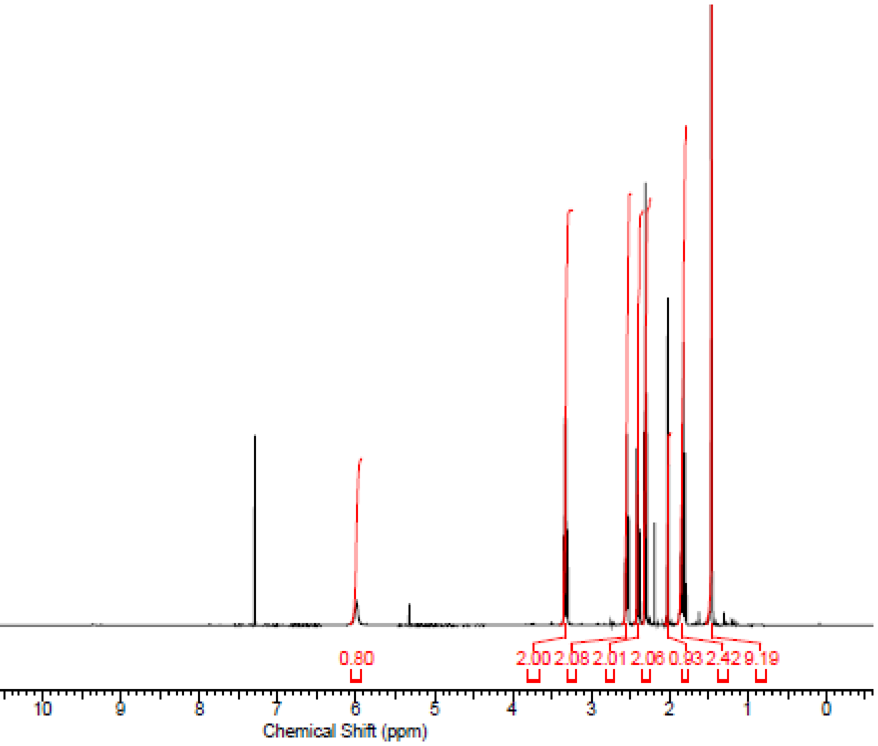

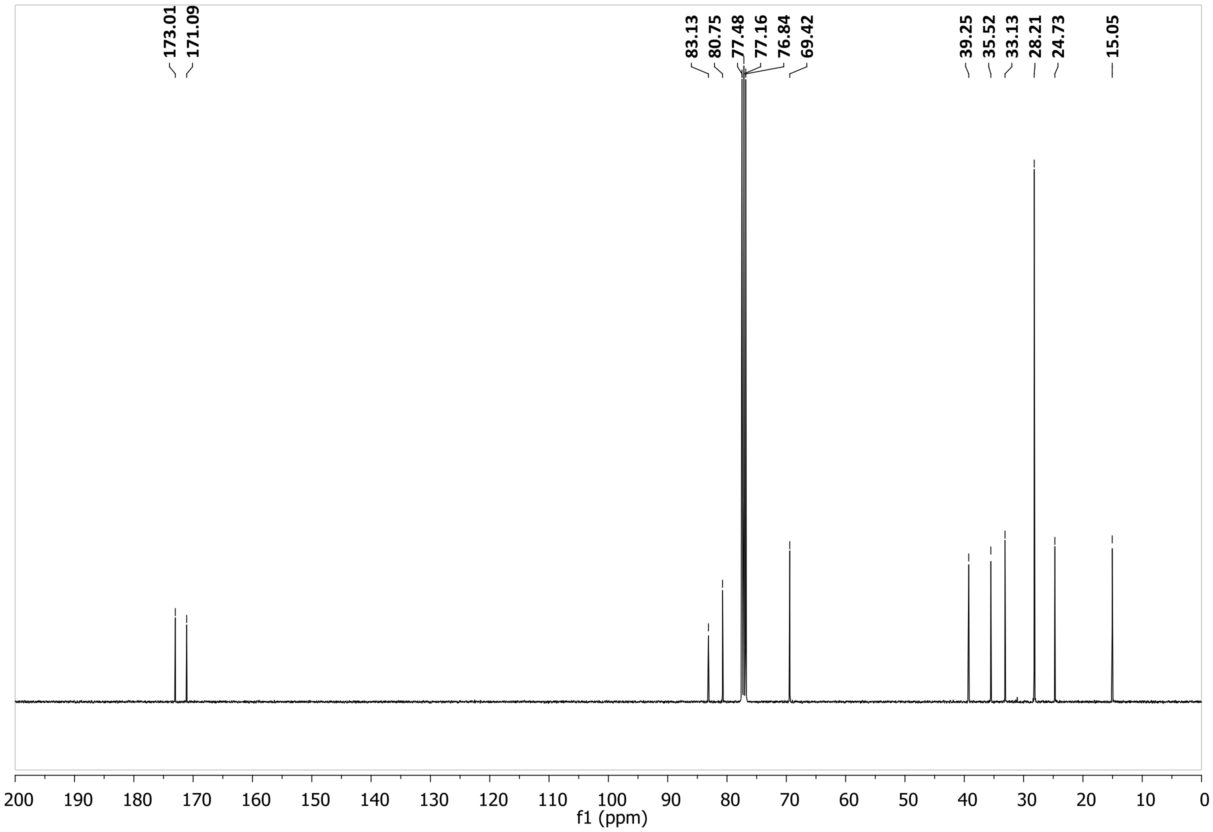

7.5 ^{1}H- and ^{13}C-NMR of compound 56

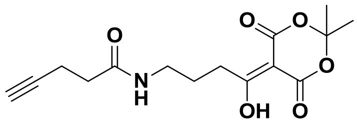

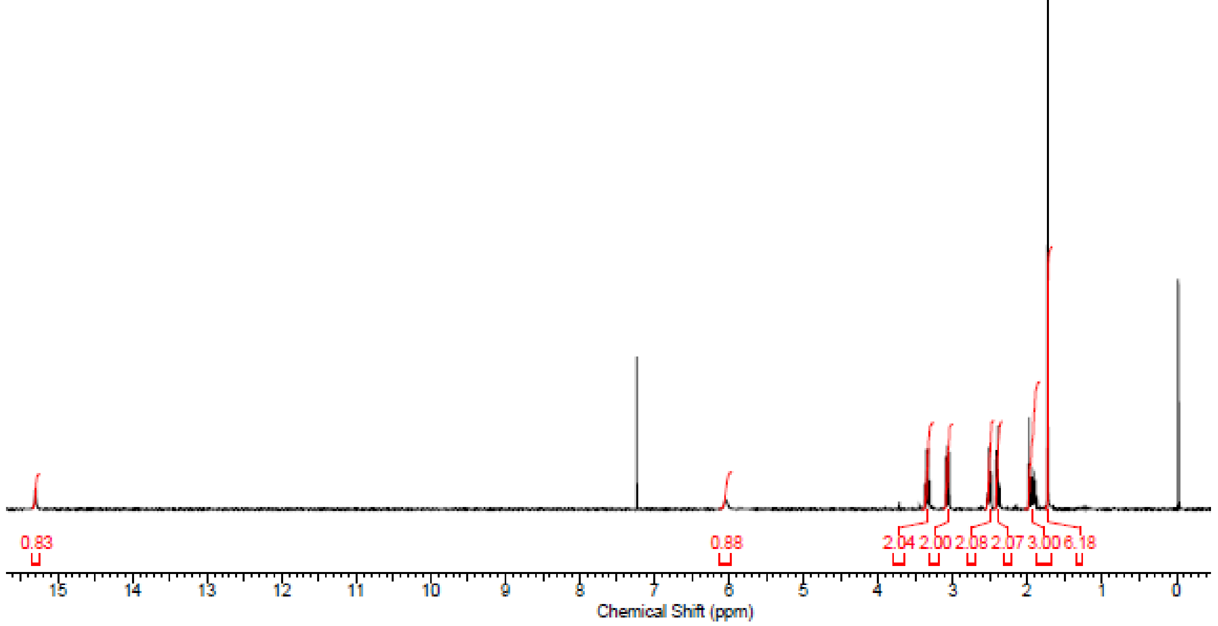

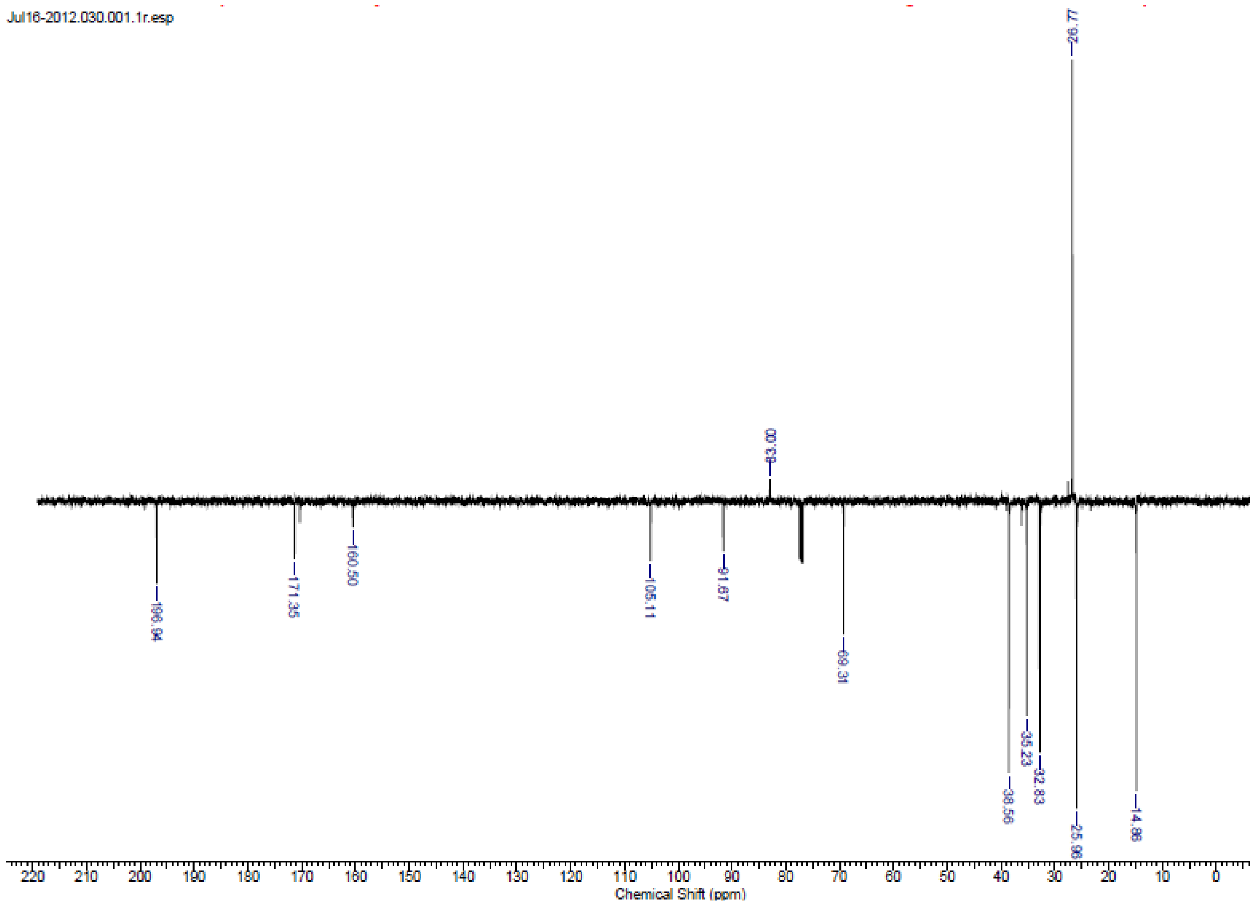

7.6 ^{1}H- and ^{13}C-NMR of compound 5

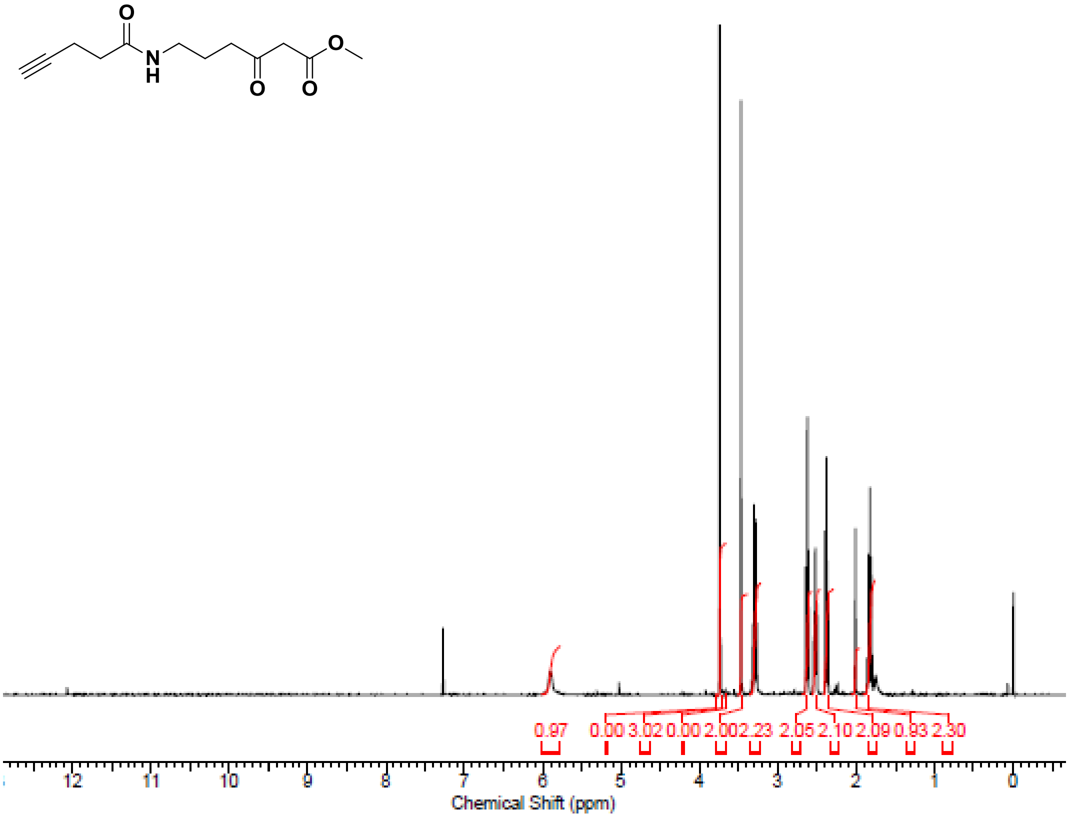

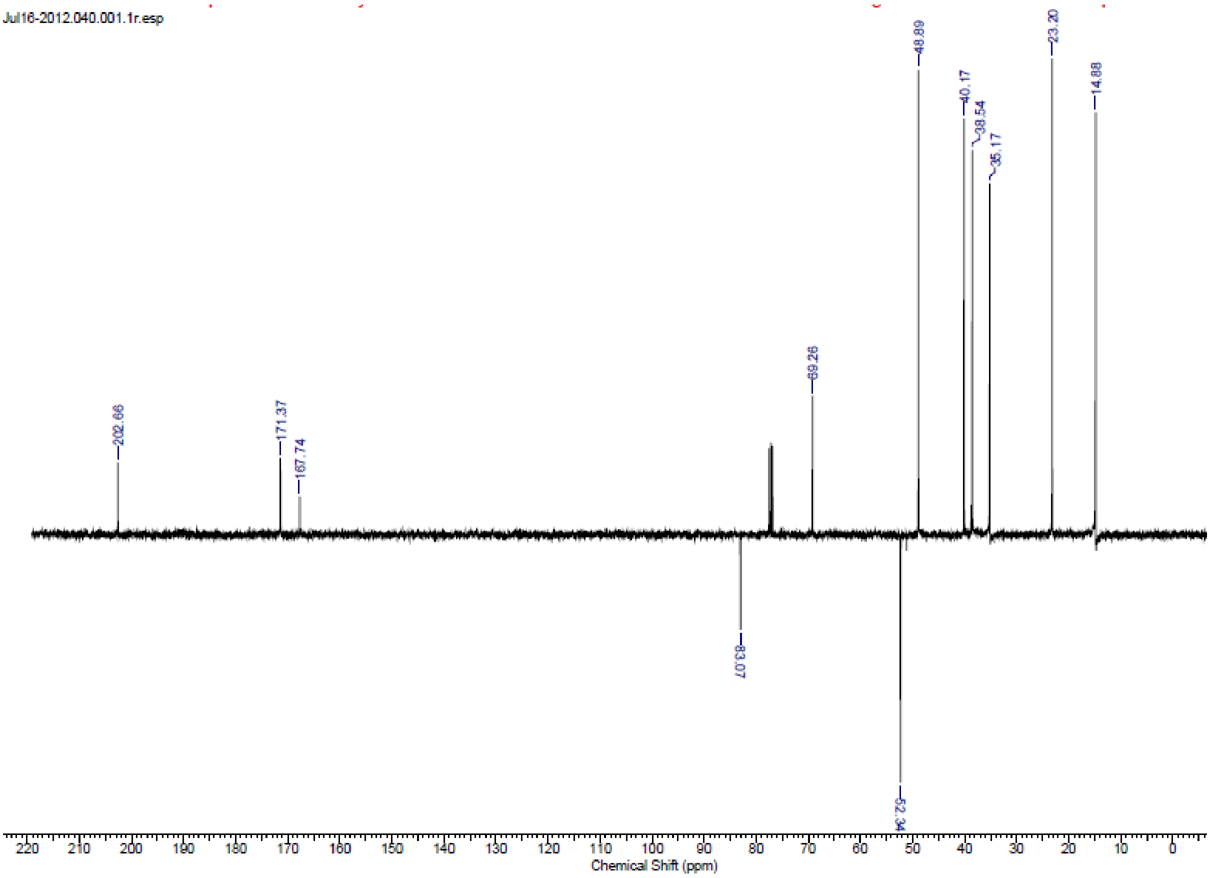

7.7 ¹H- and ¹³C-NMR of compound 9

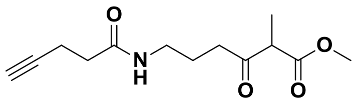

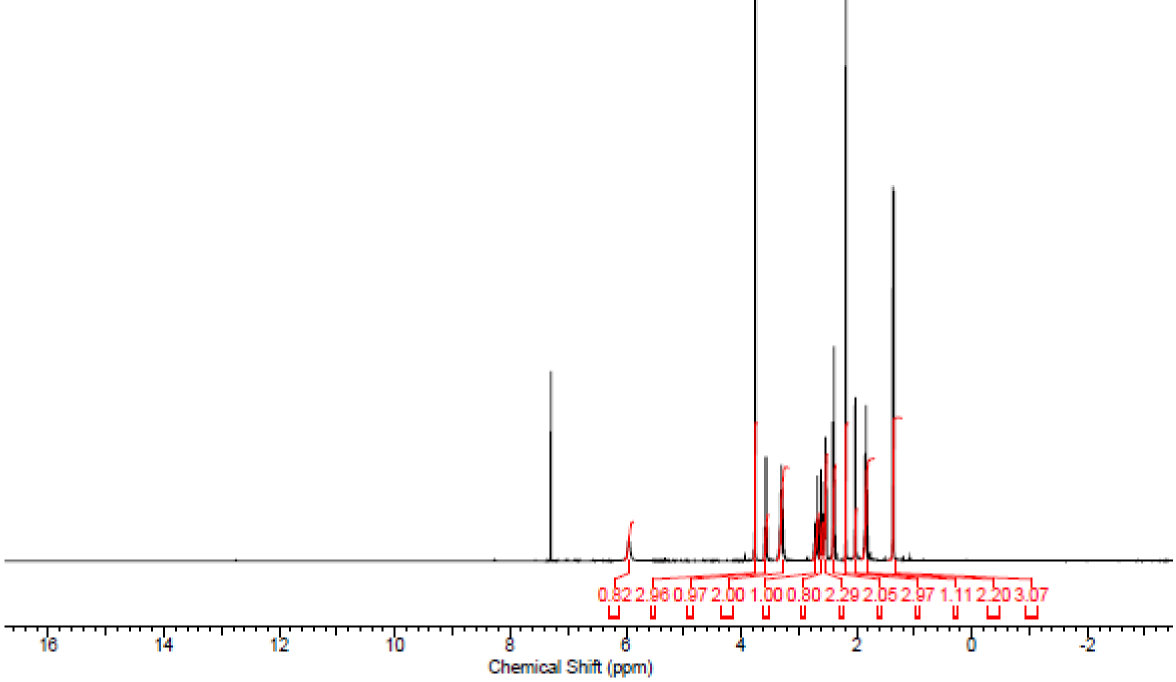

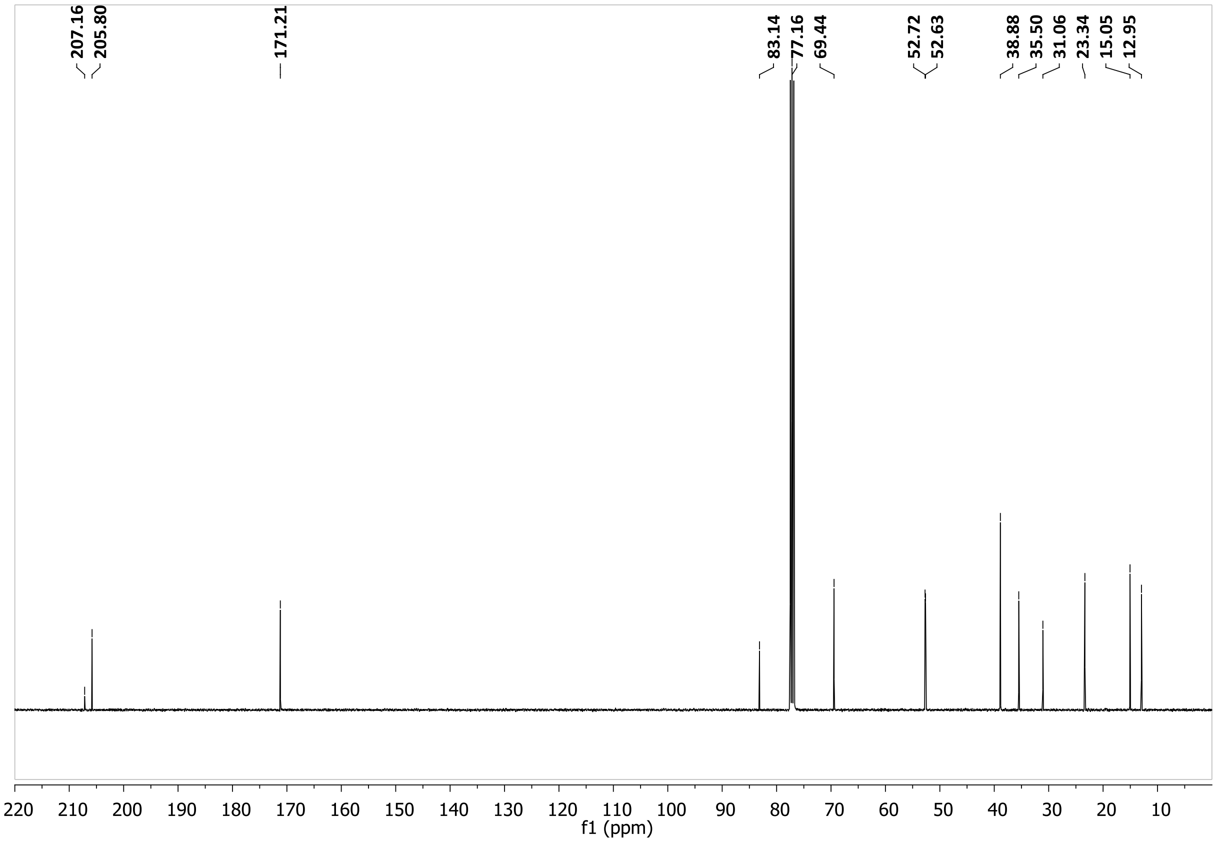

7.8 ¹H- and ¹³C-NMR of compound 12

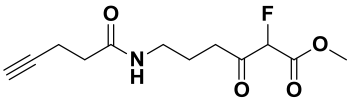

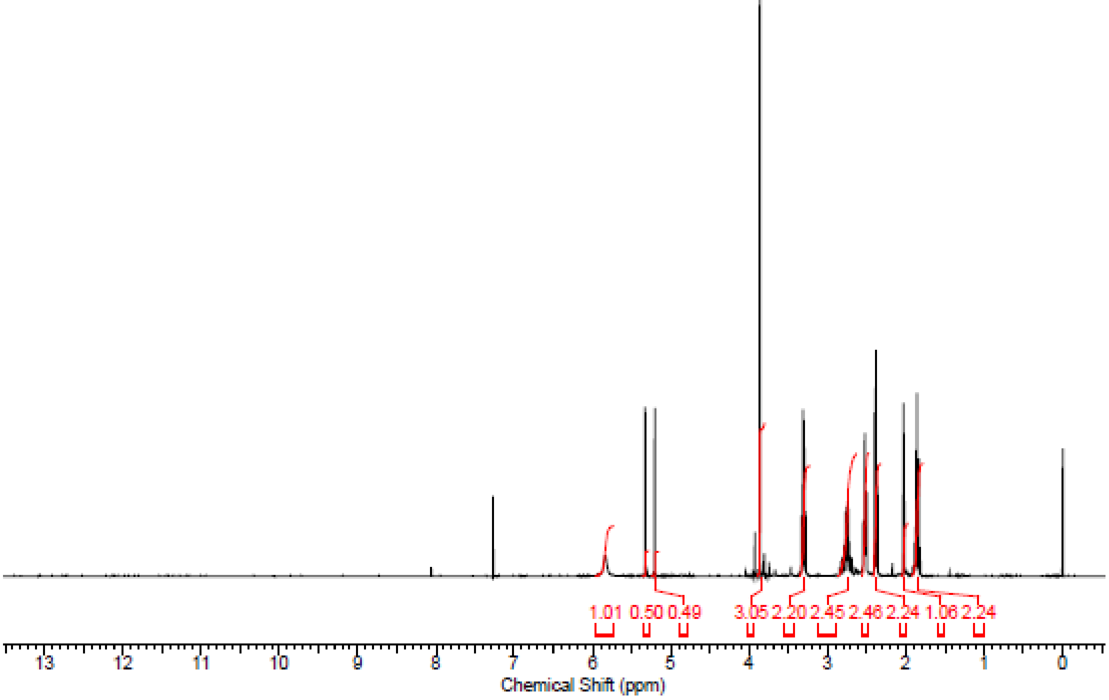

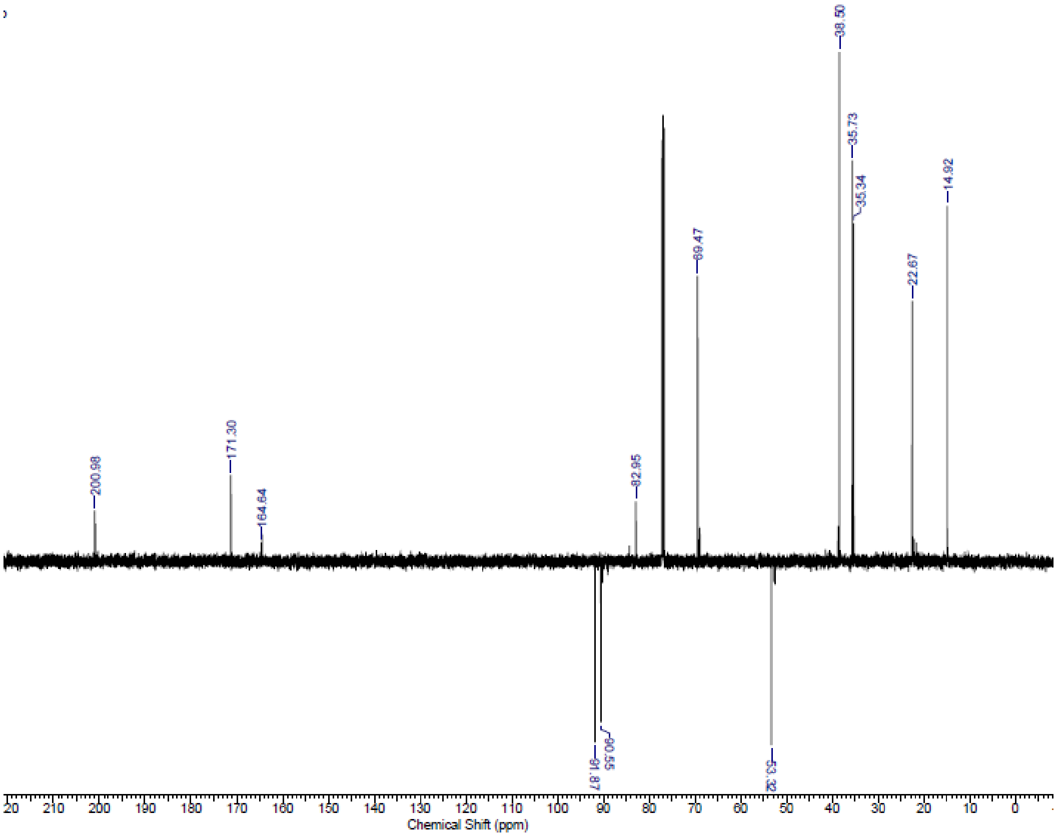

7.9 ^{1}H- and ^{13}C-NMR of compound 59

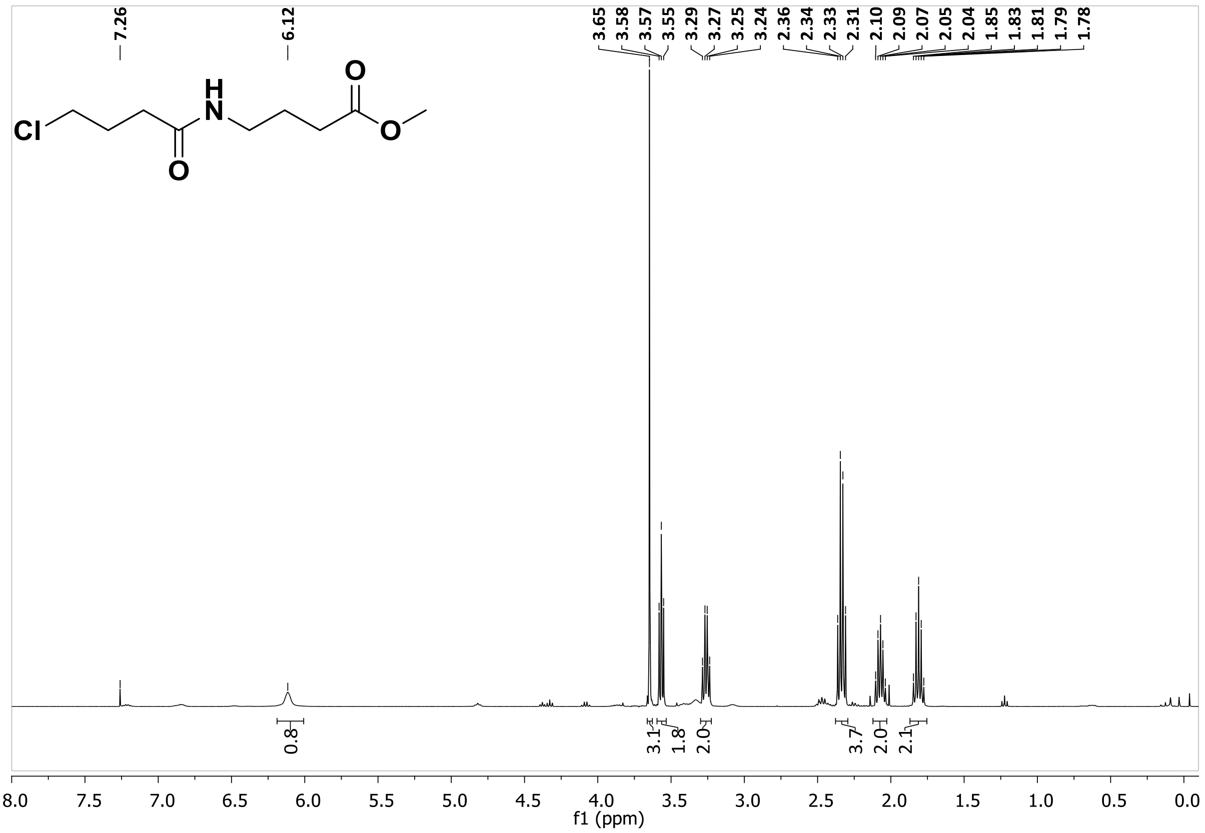

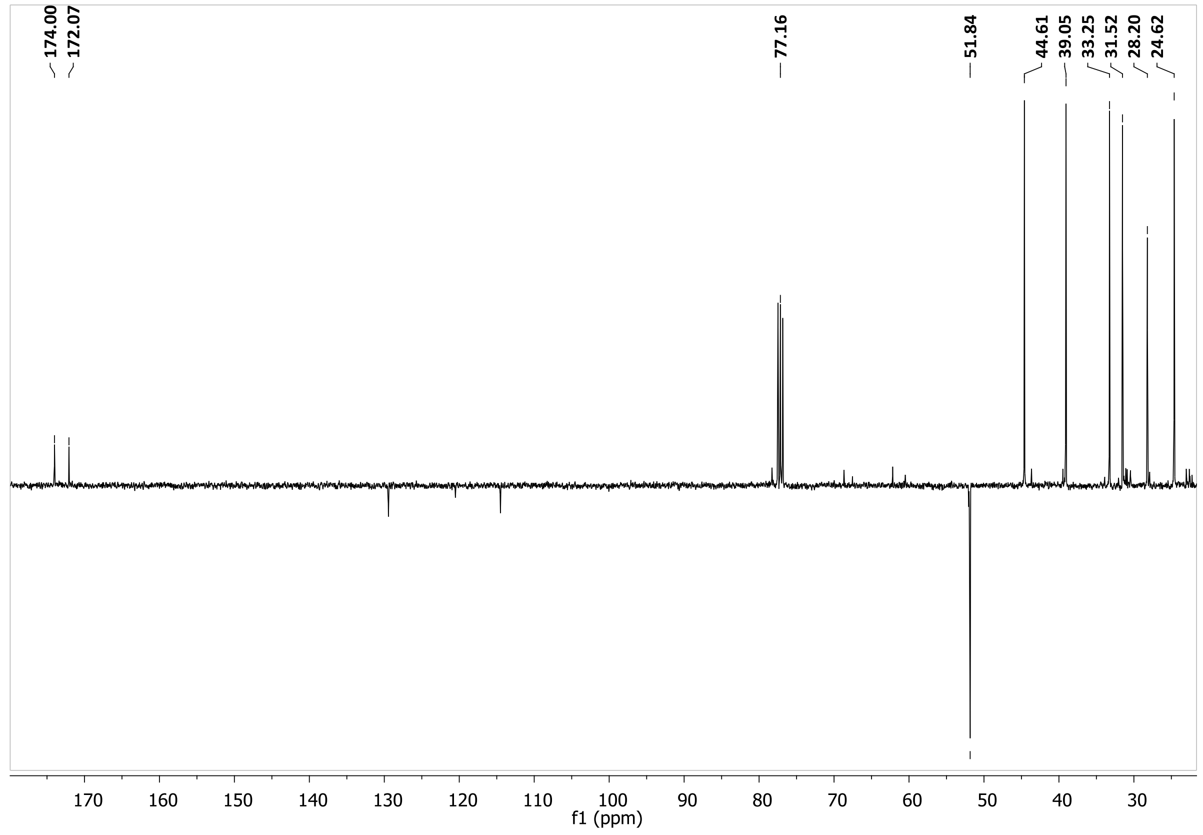

7.10 ^{1}H- and ^{13}C-NMR of compound 60

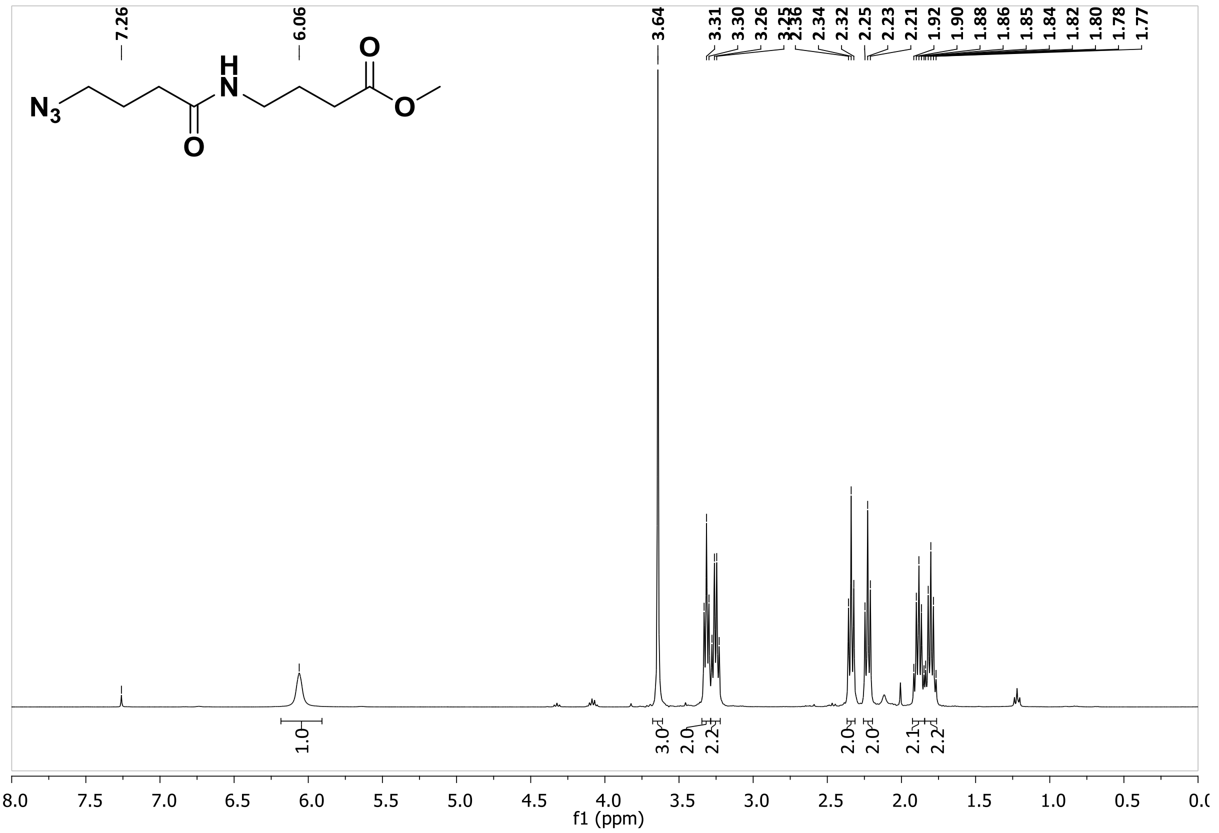

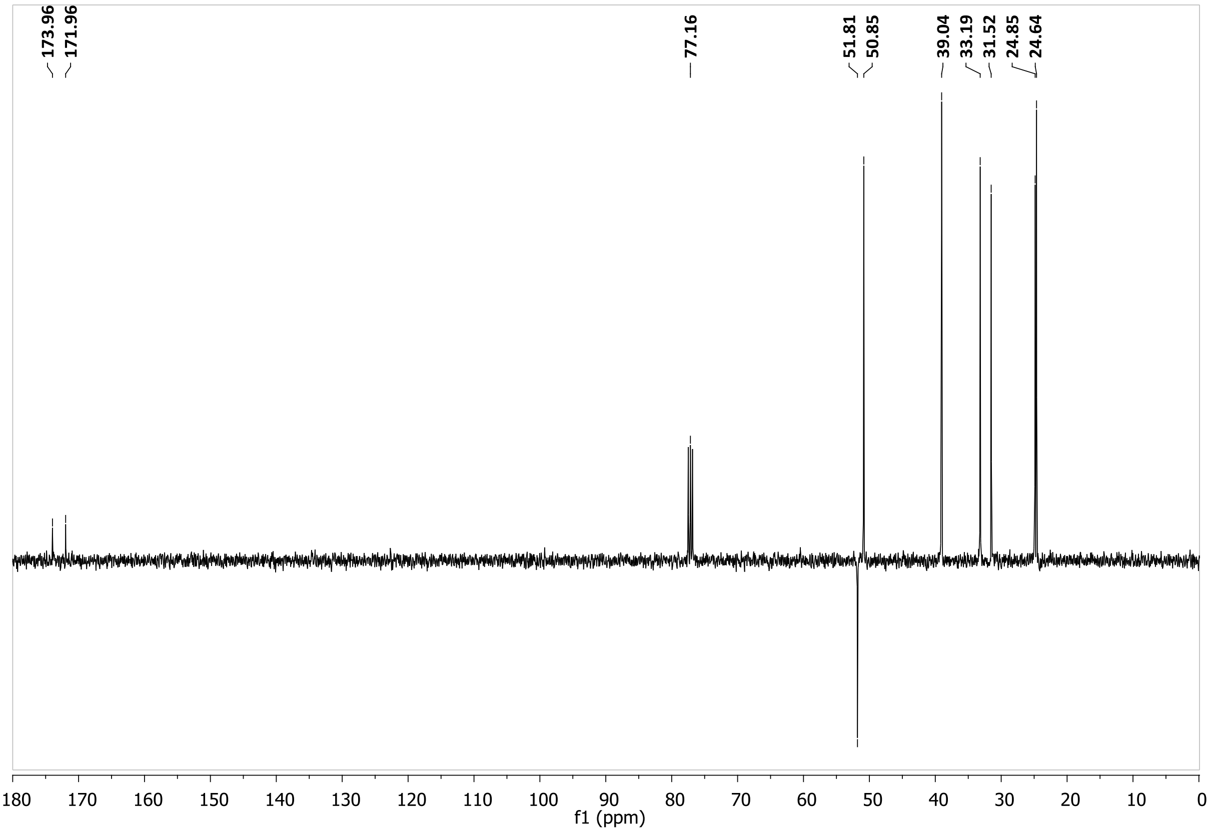

7.11 ^{1}H- and ^{13}C-NMR of compound 61

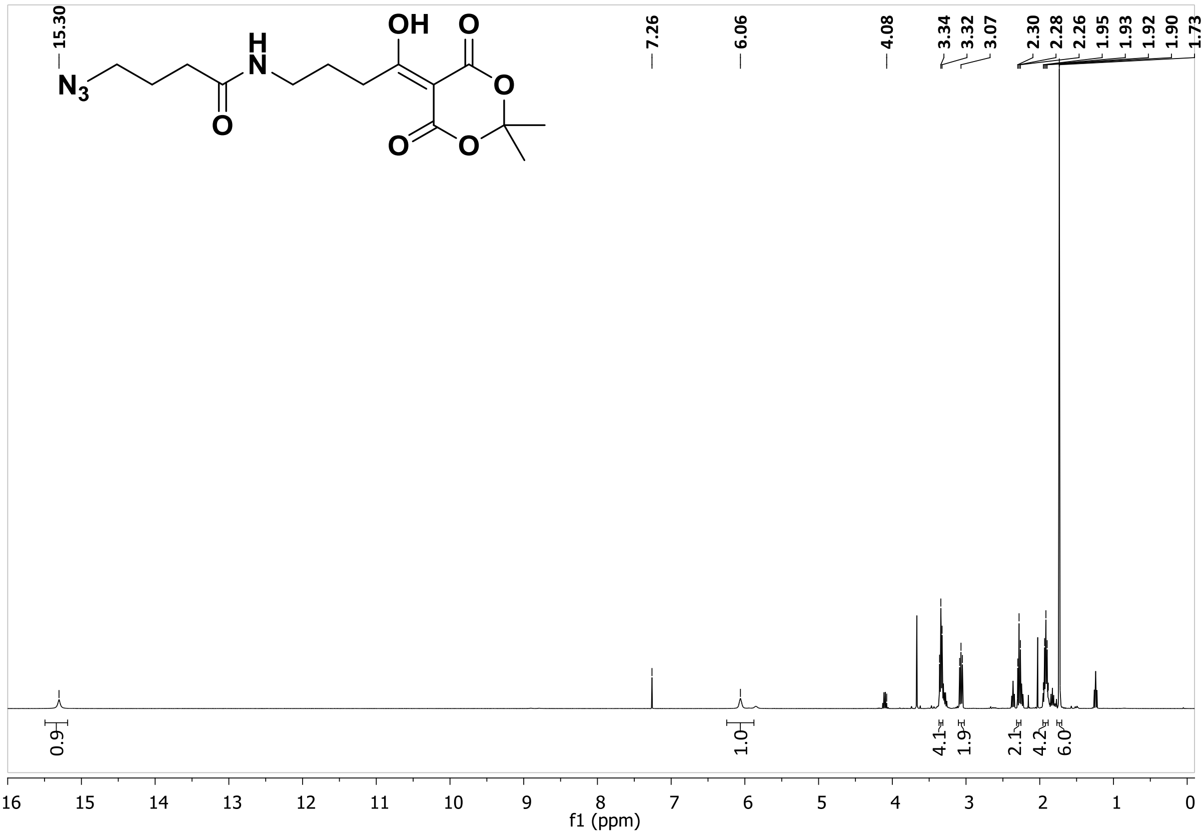

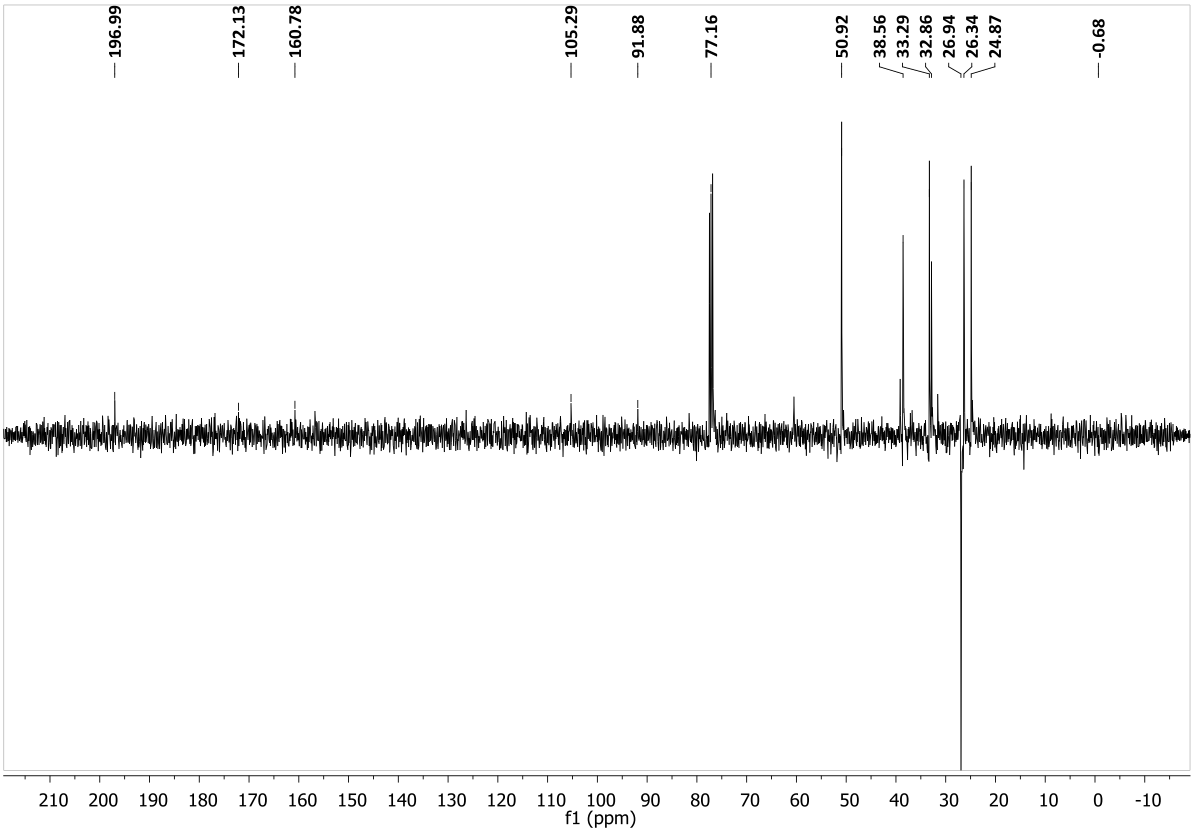

7.12 ¹H- and ¹³C-NMR of compound 6

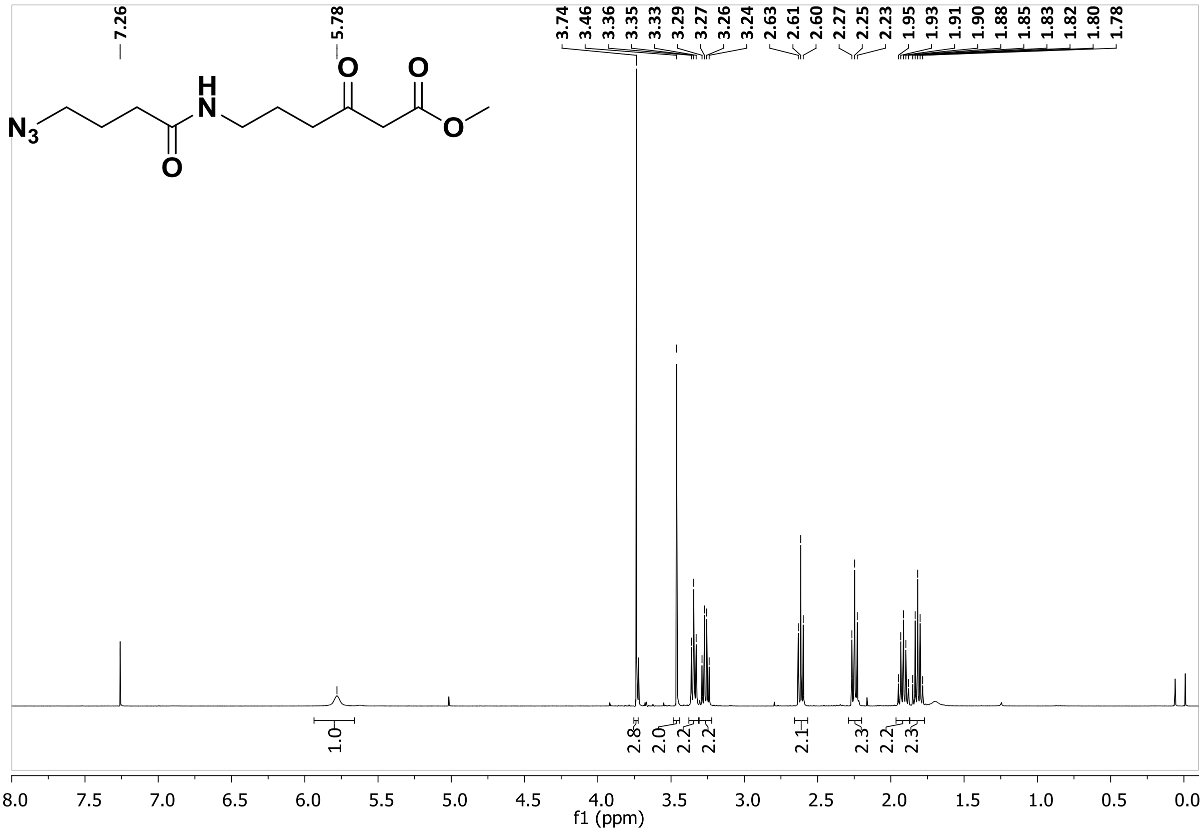

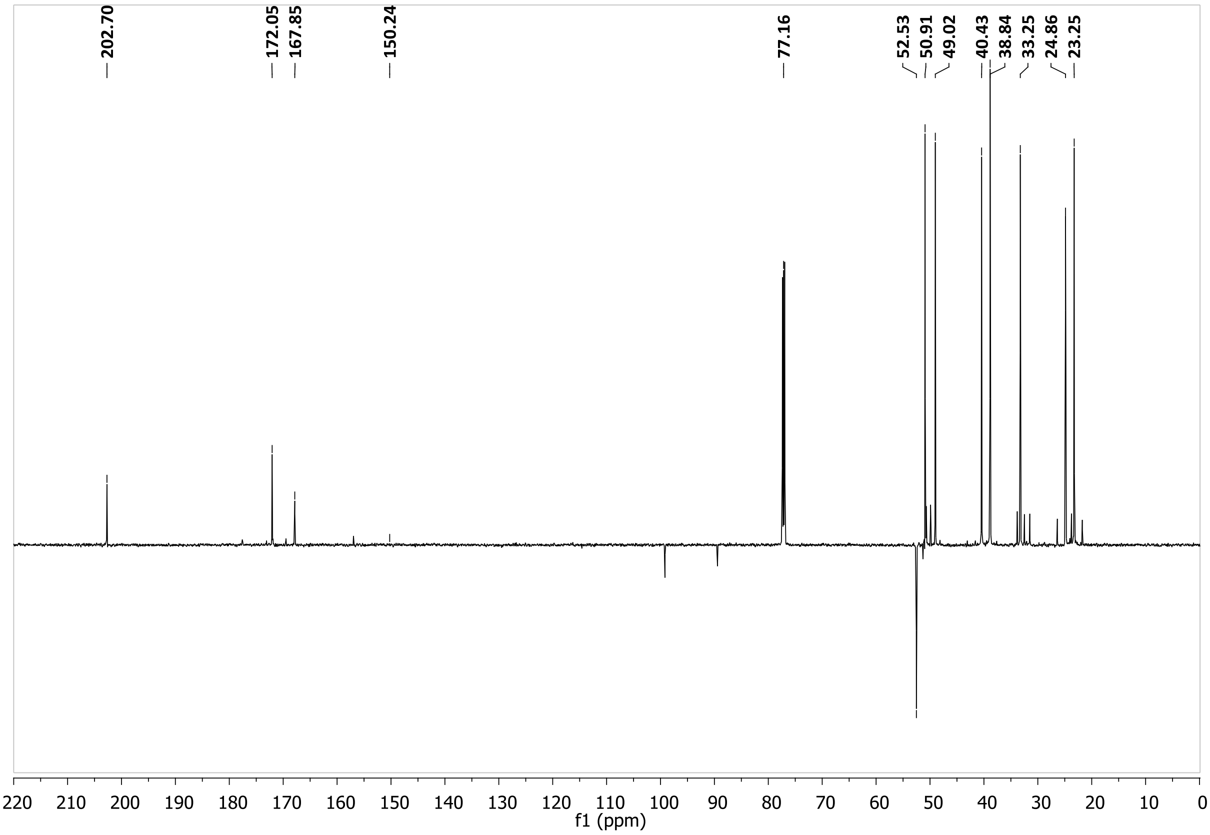

7.13 ^{1}H- and ^{13}C-NMR of compound 63

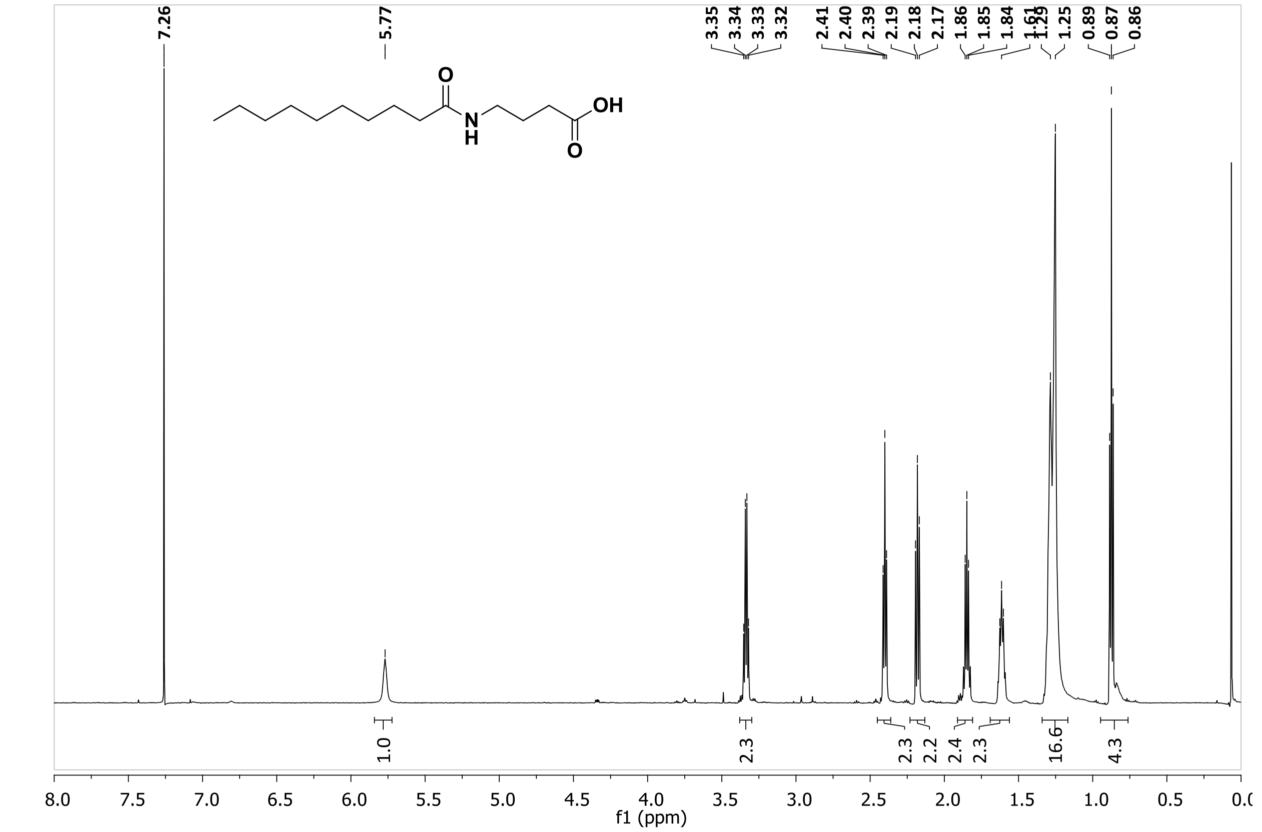

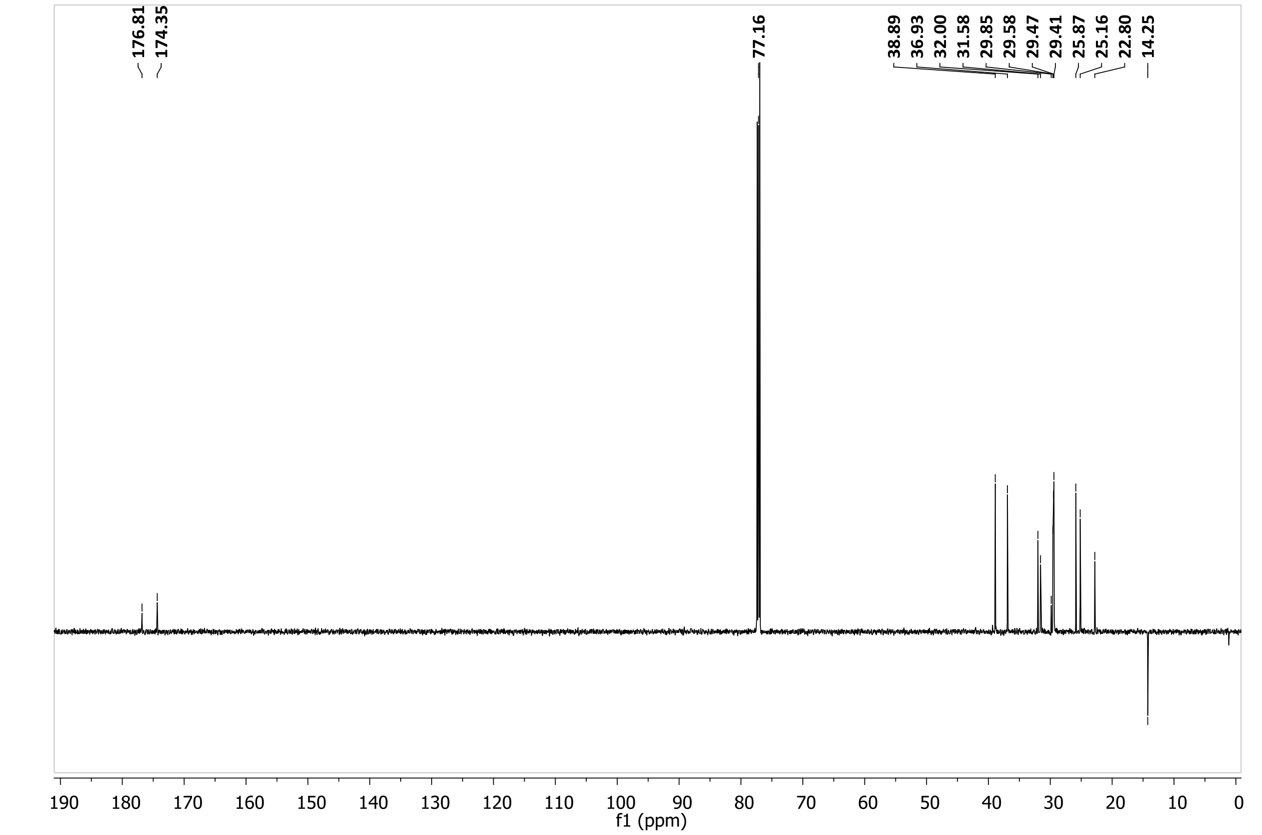

7.14 ^{1}H- and ^{13}C-NMR of compound 64

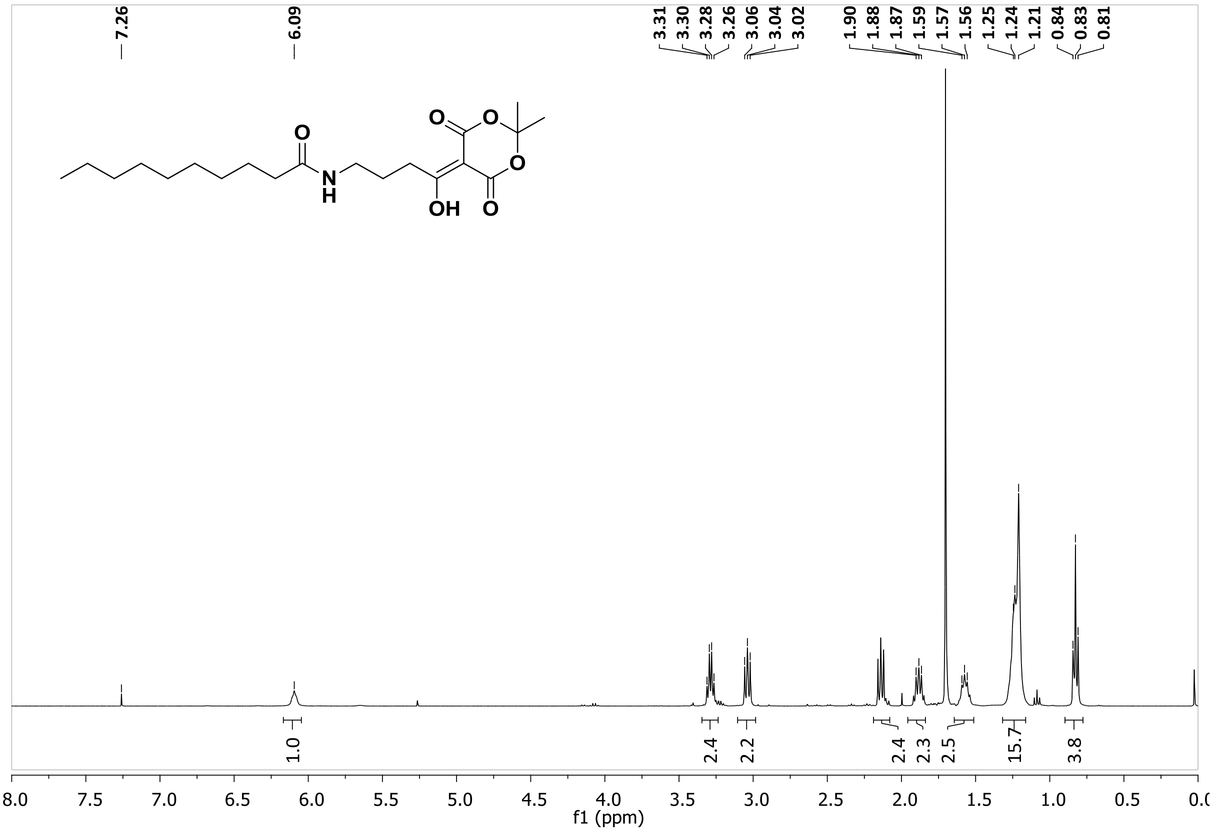

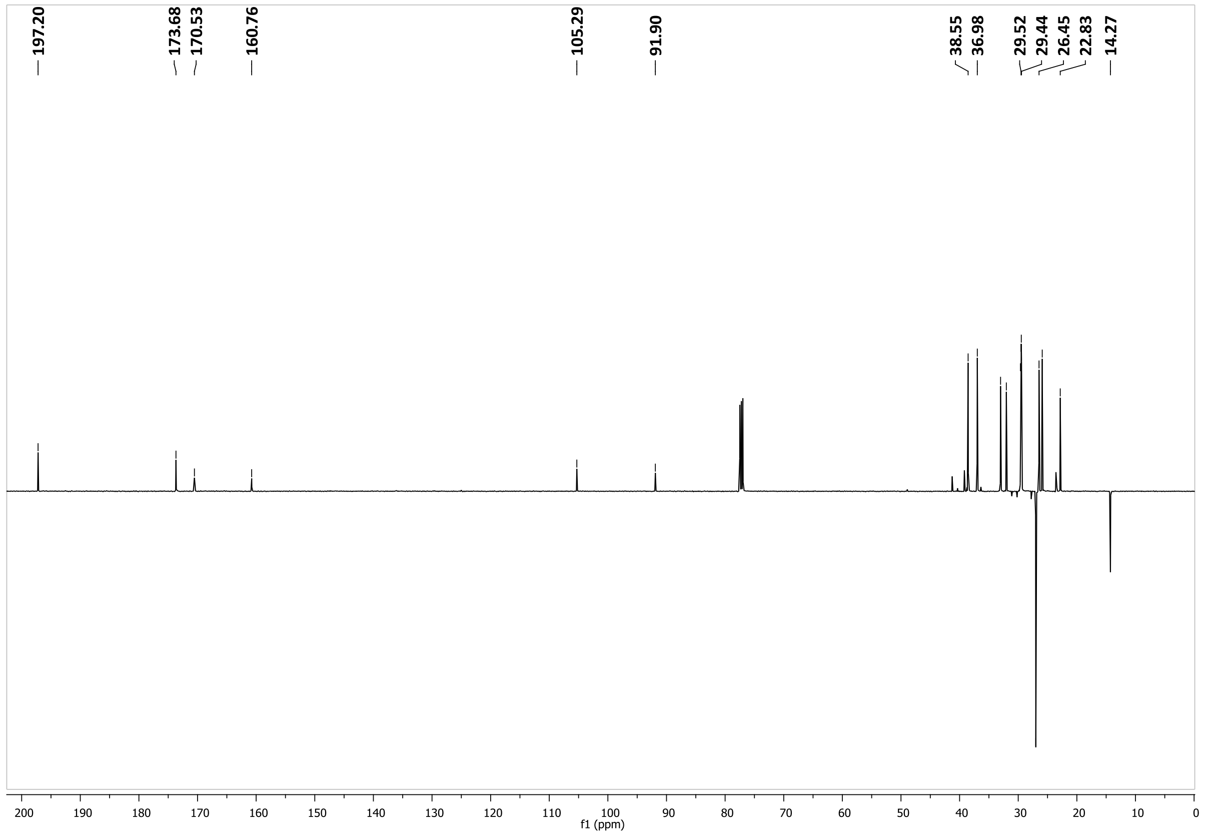

7.15 1H- and 13C-NMR of compound 7

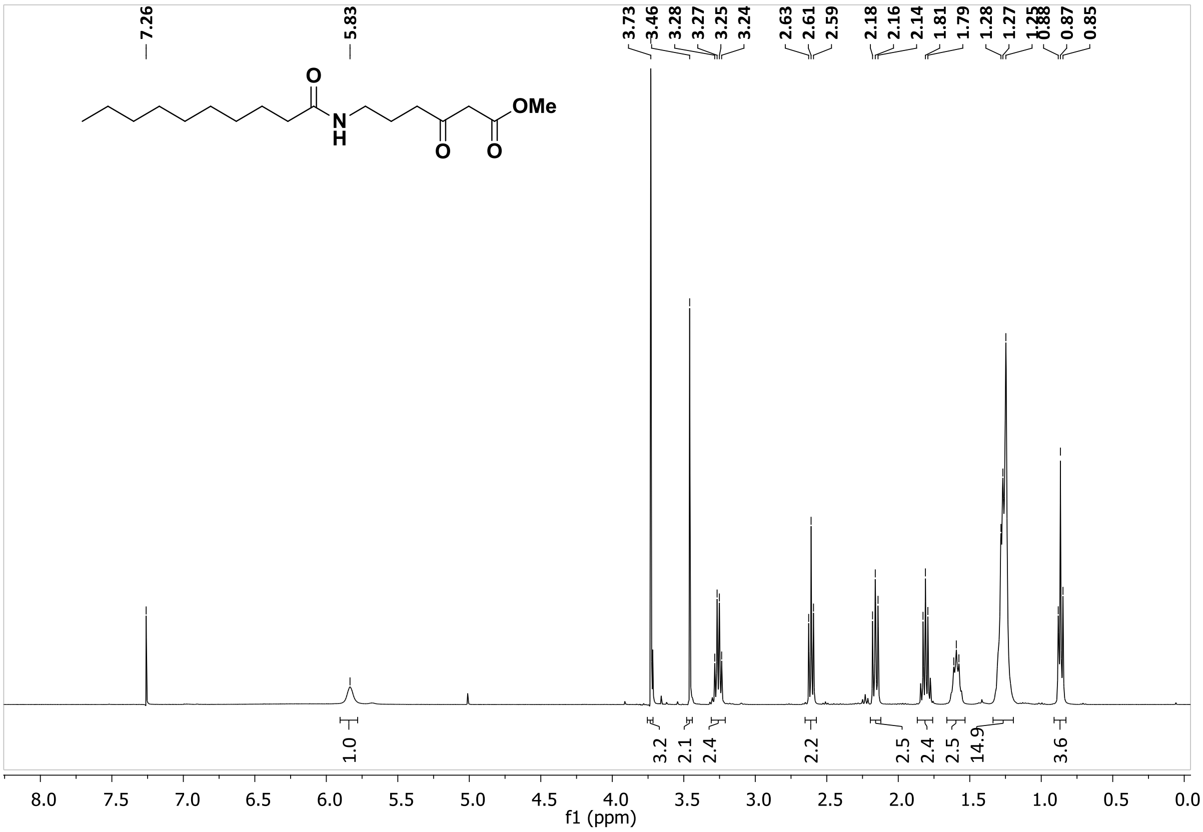

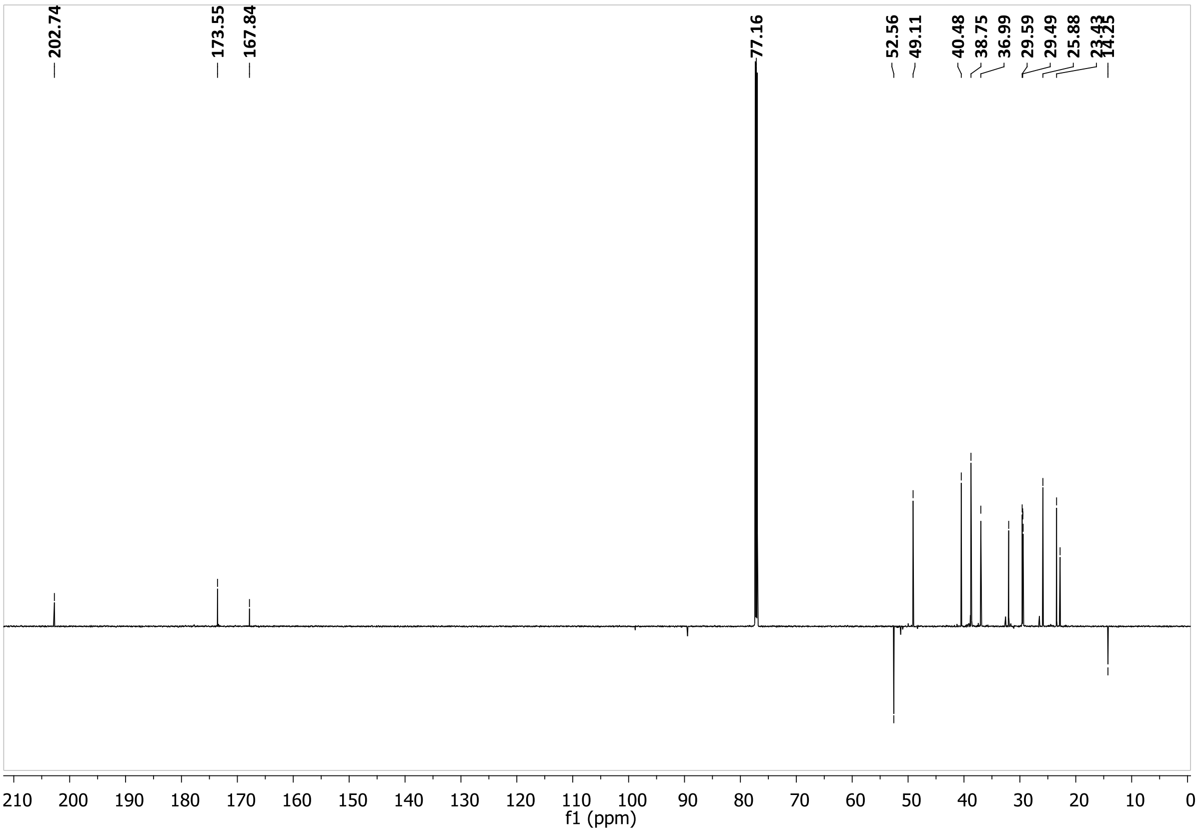

7.16 ^{1}H- and ^{13}C-NMR of compound 13

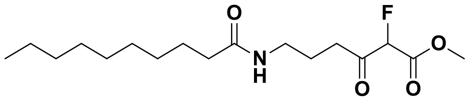

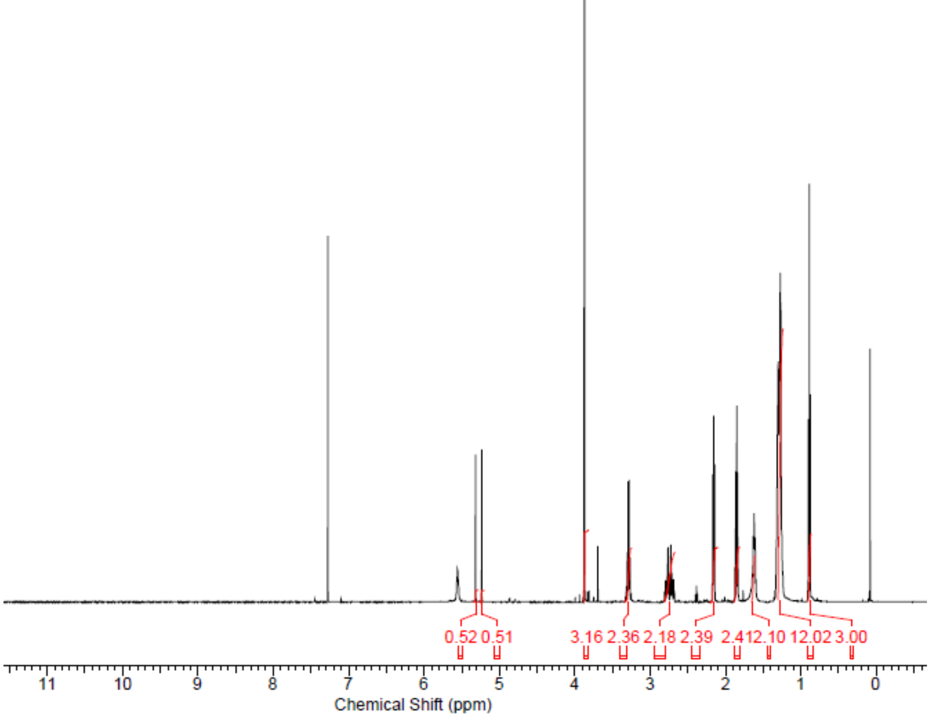

7.17 ^{1}H- and ^{13}C-NMR of compound 66

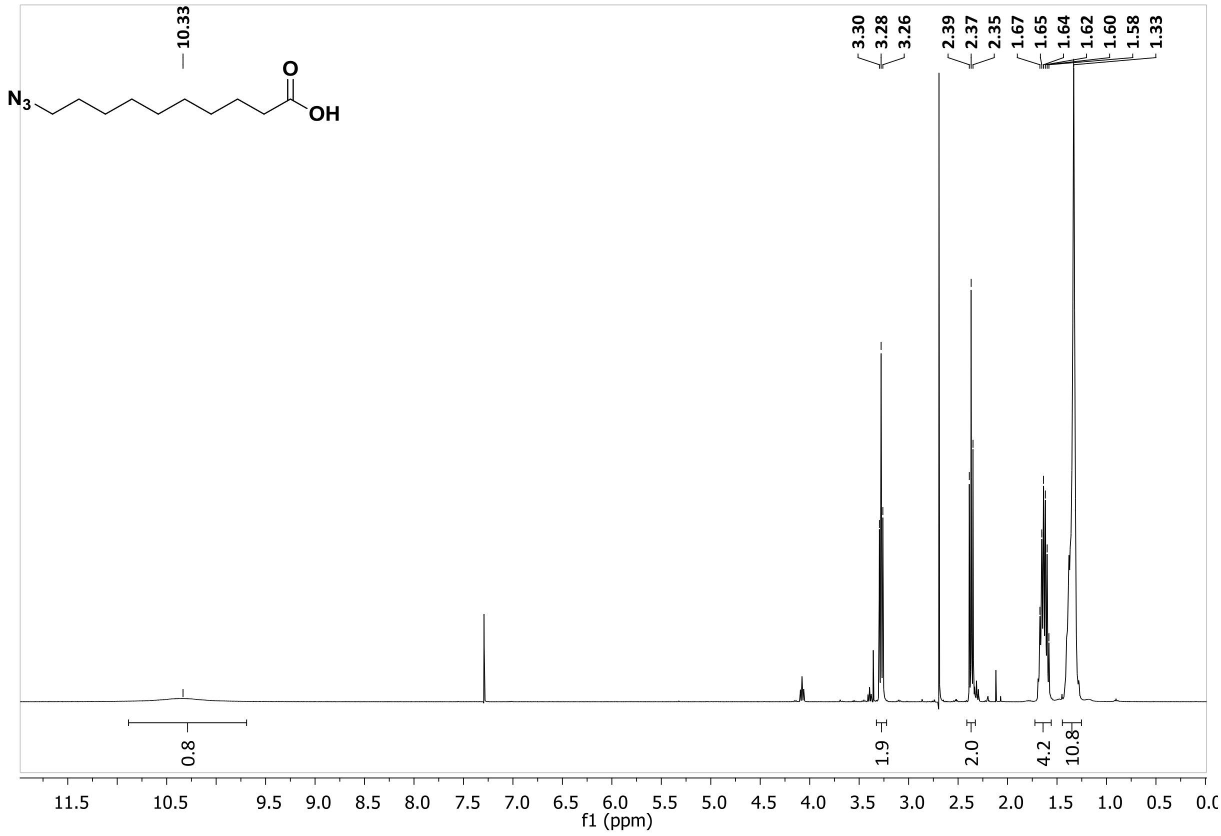

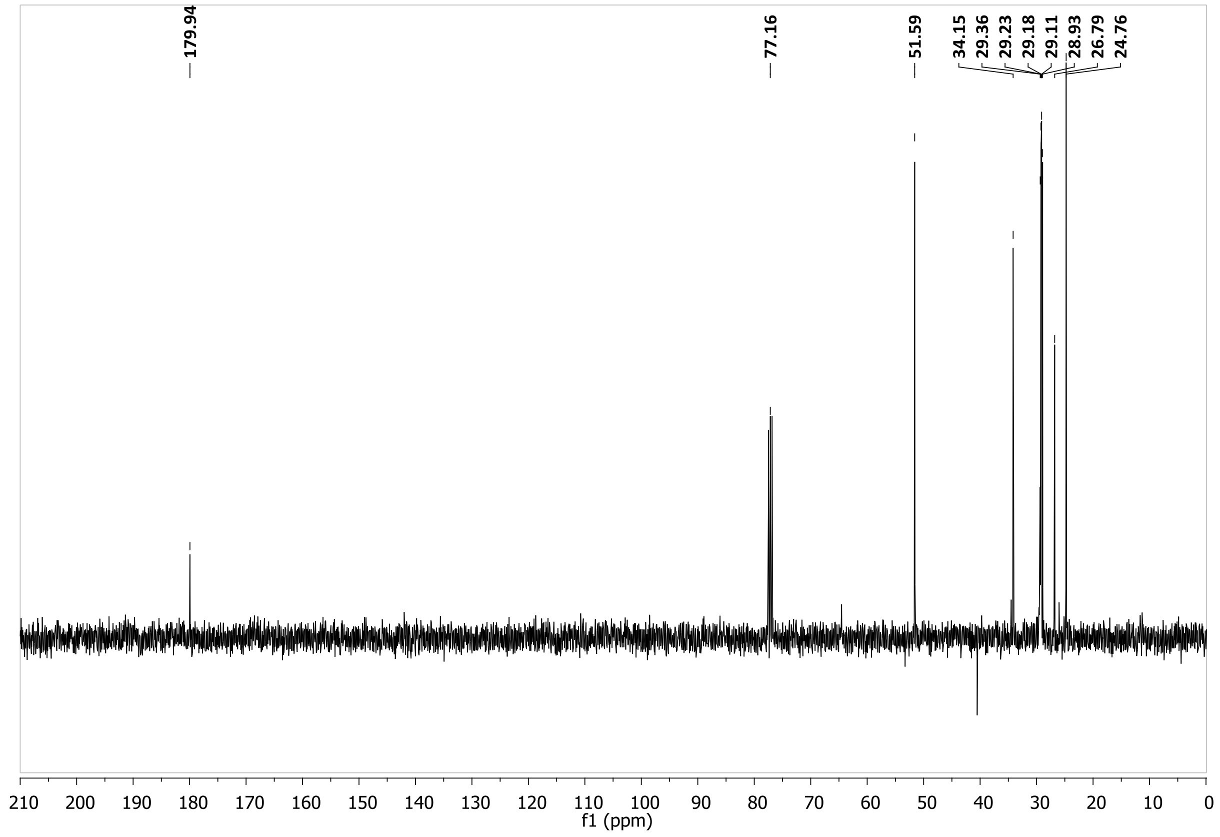

7.18 ^{1}H- and ^{13}C-NMR of compound 67

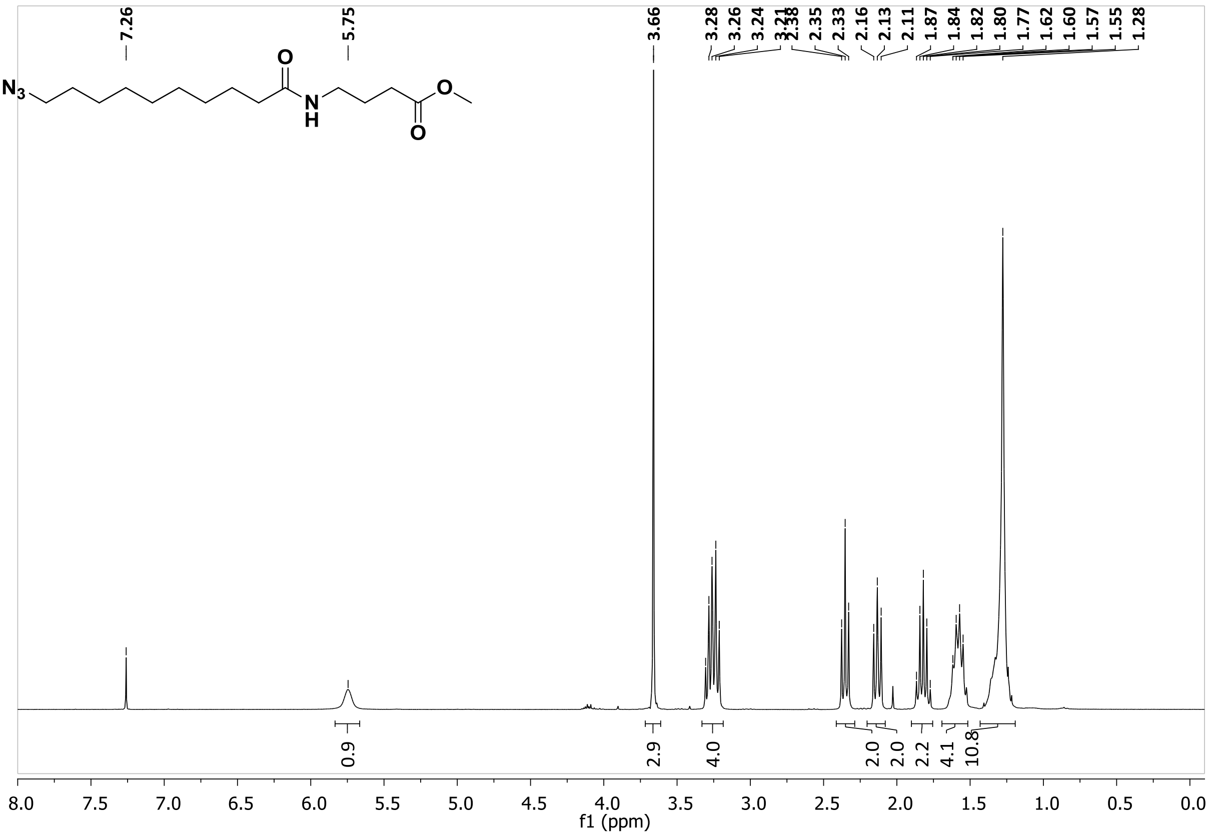

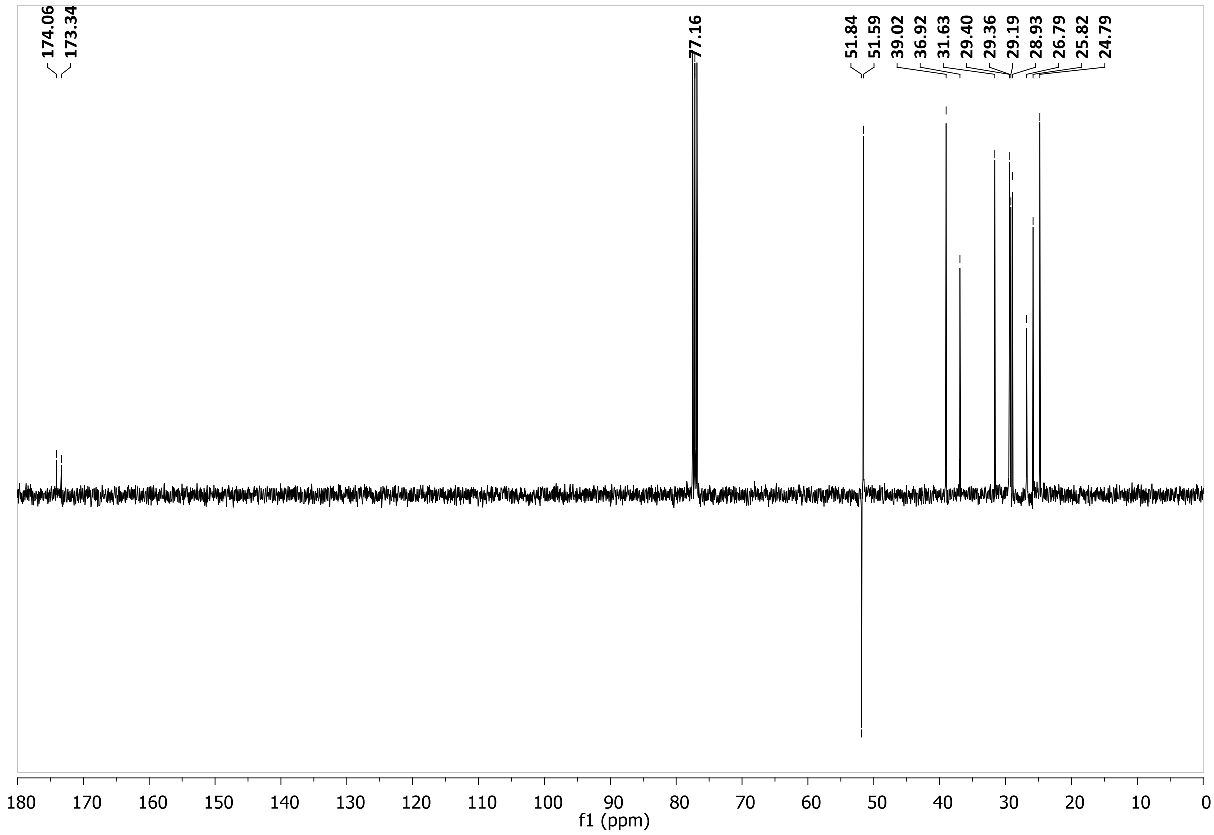

7.19 ^{1}H- and ^{13}C-NMR of compound 68

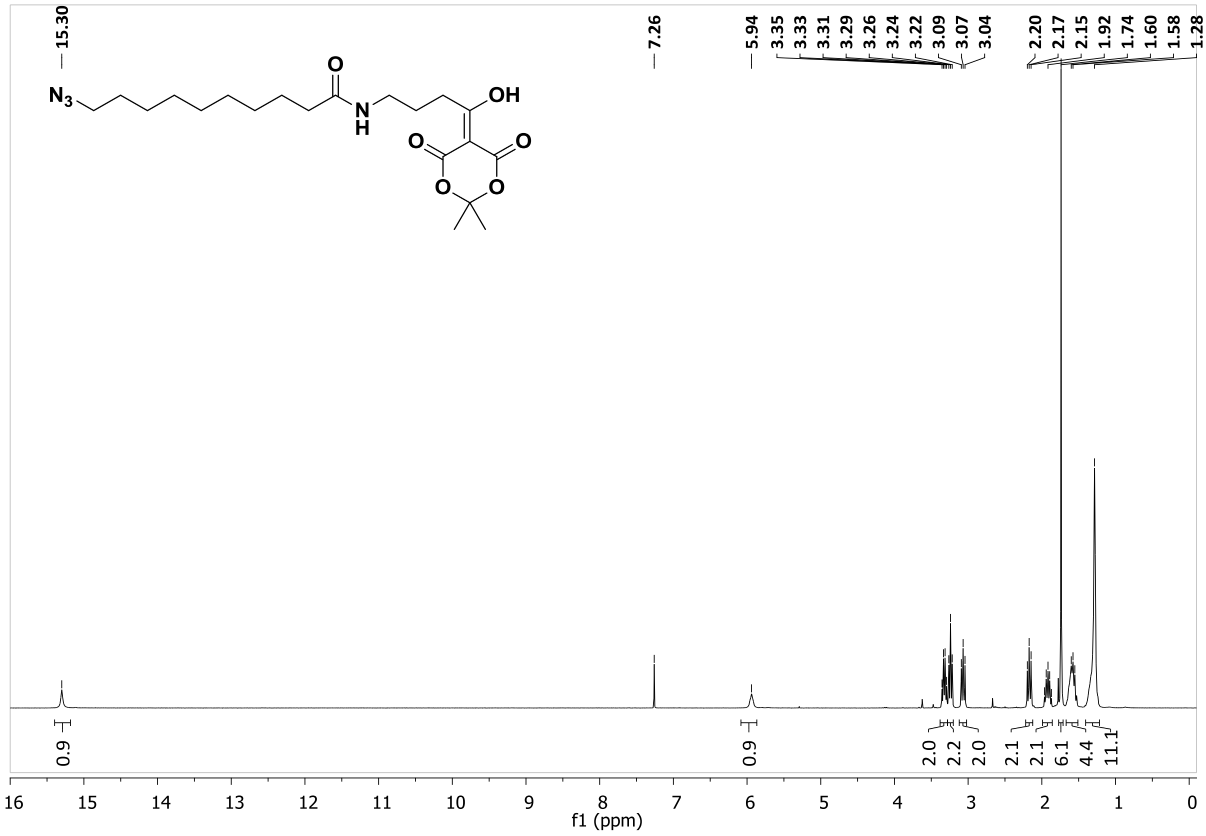

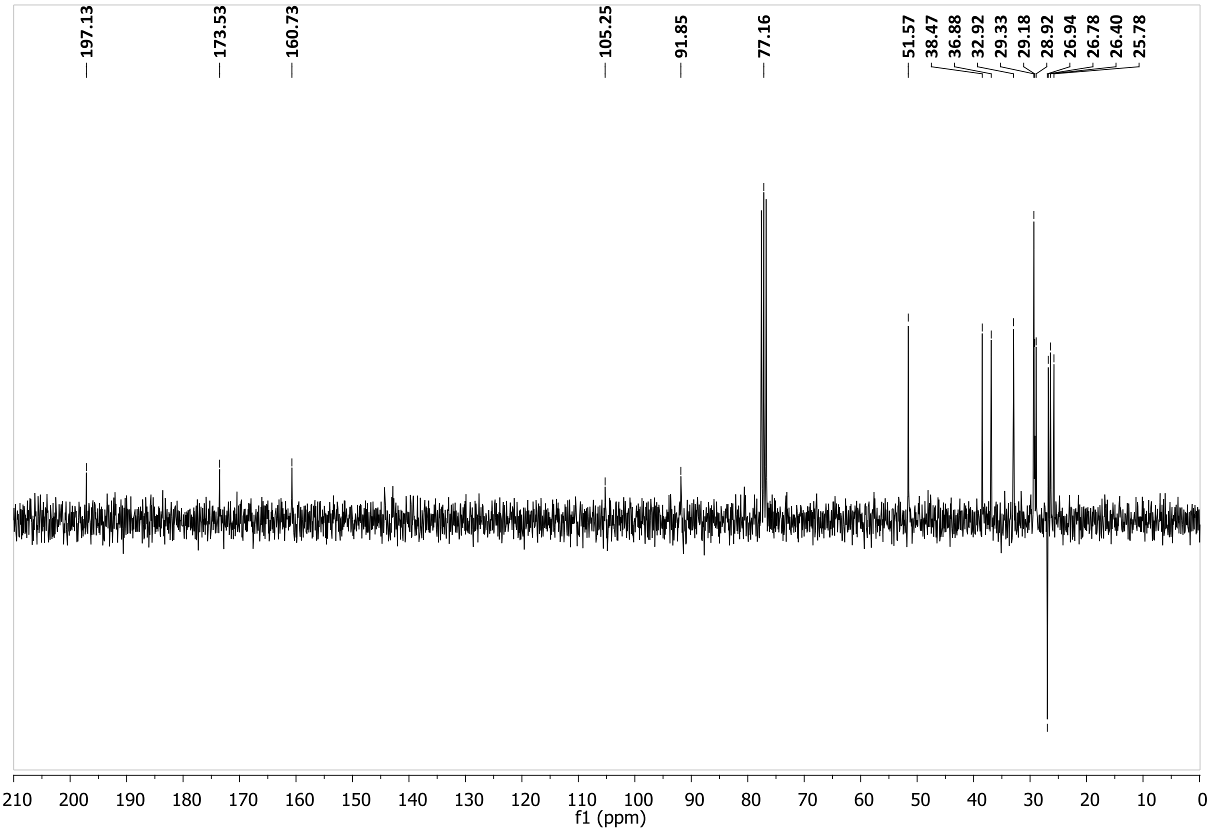

7.20 ^{1}H- and ^{13}C-NMR of compound 8

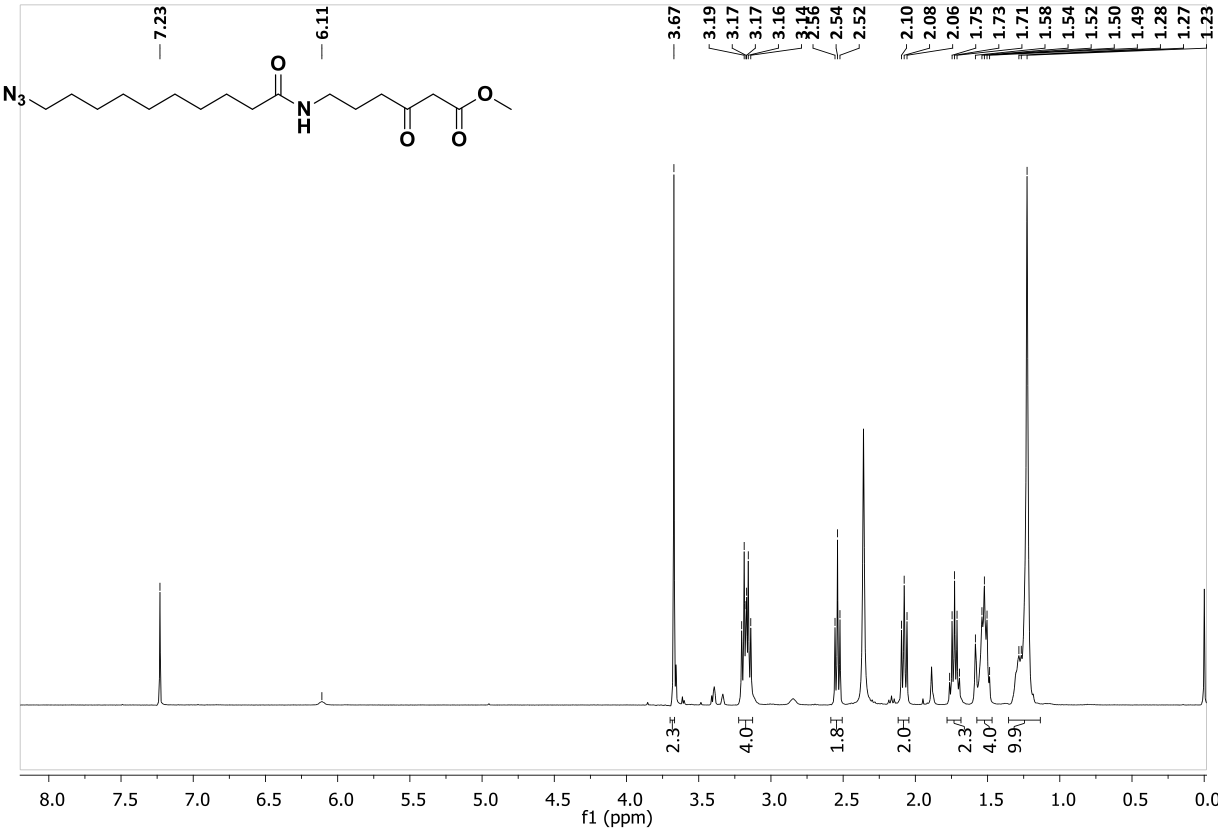

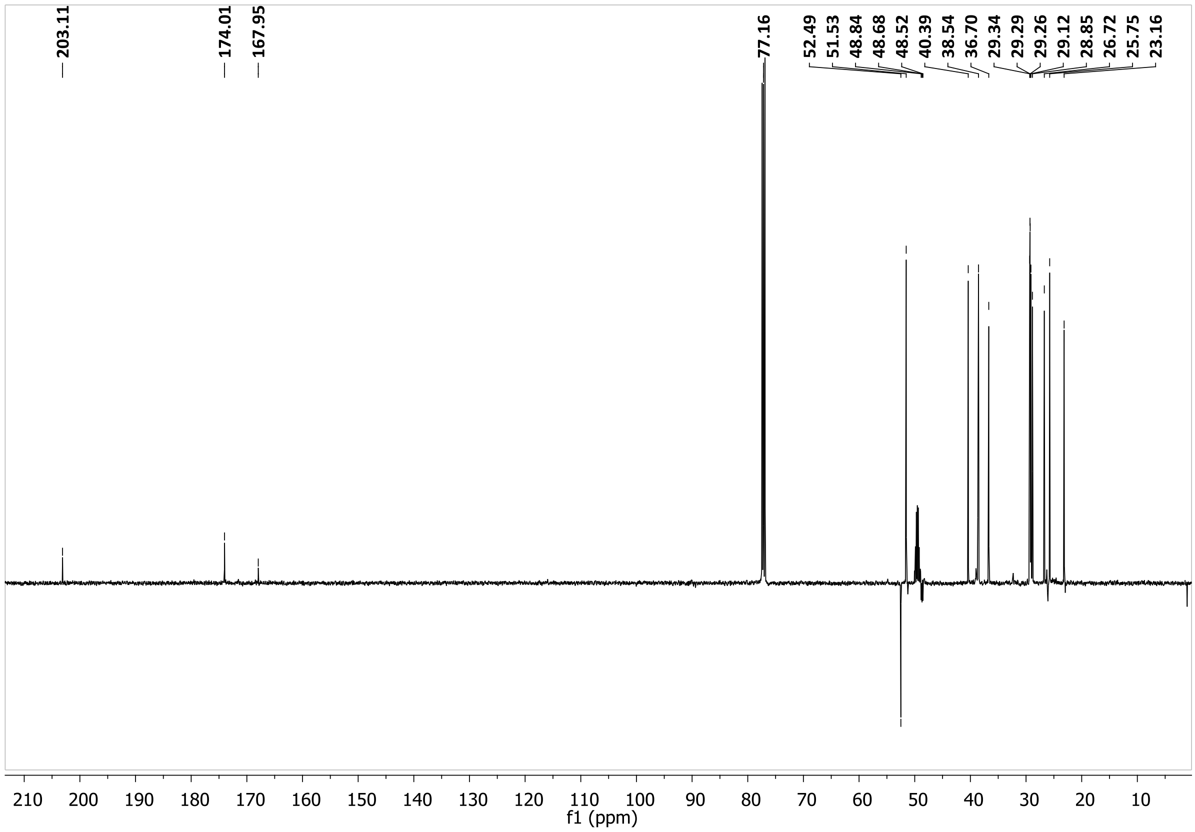

7.21 ^{1}H- and ^{13}C-NMR of compound 11a

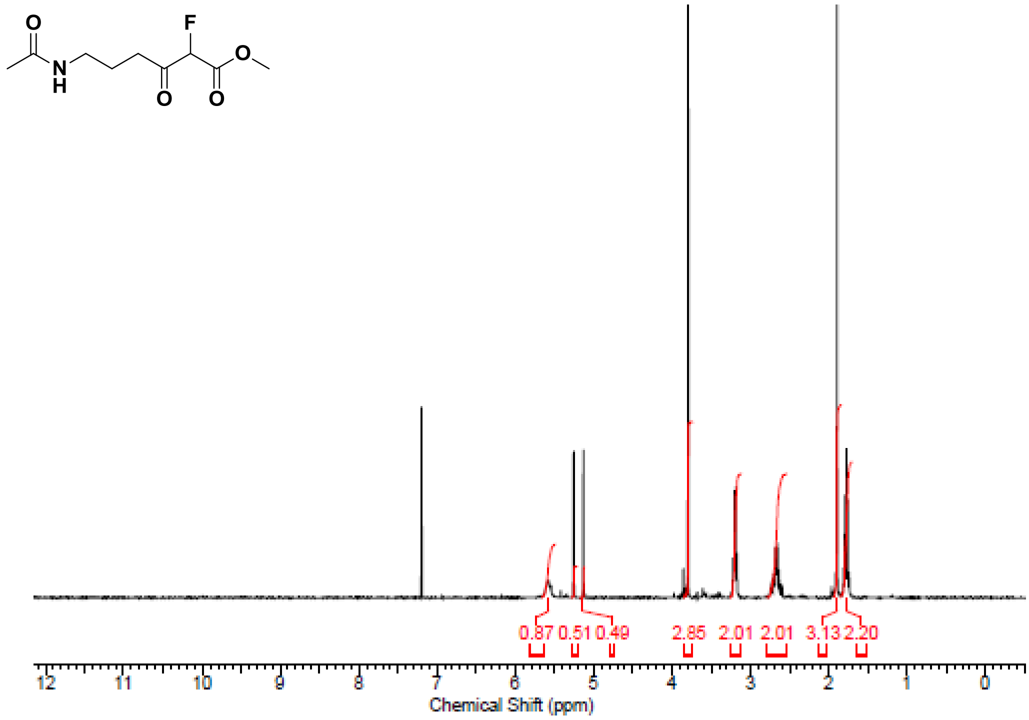

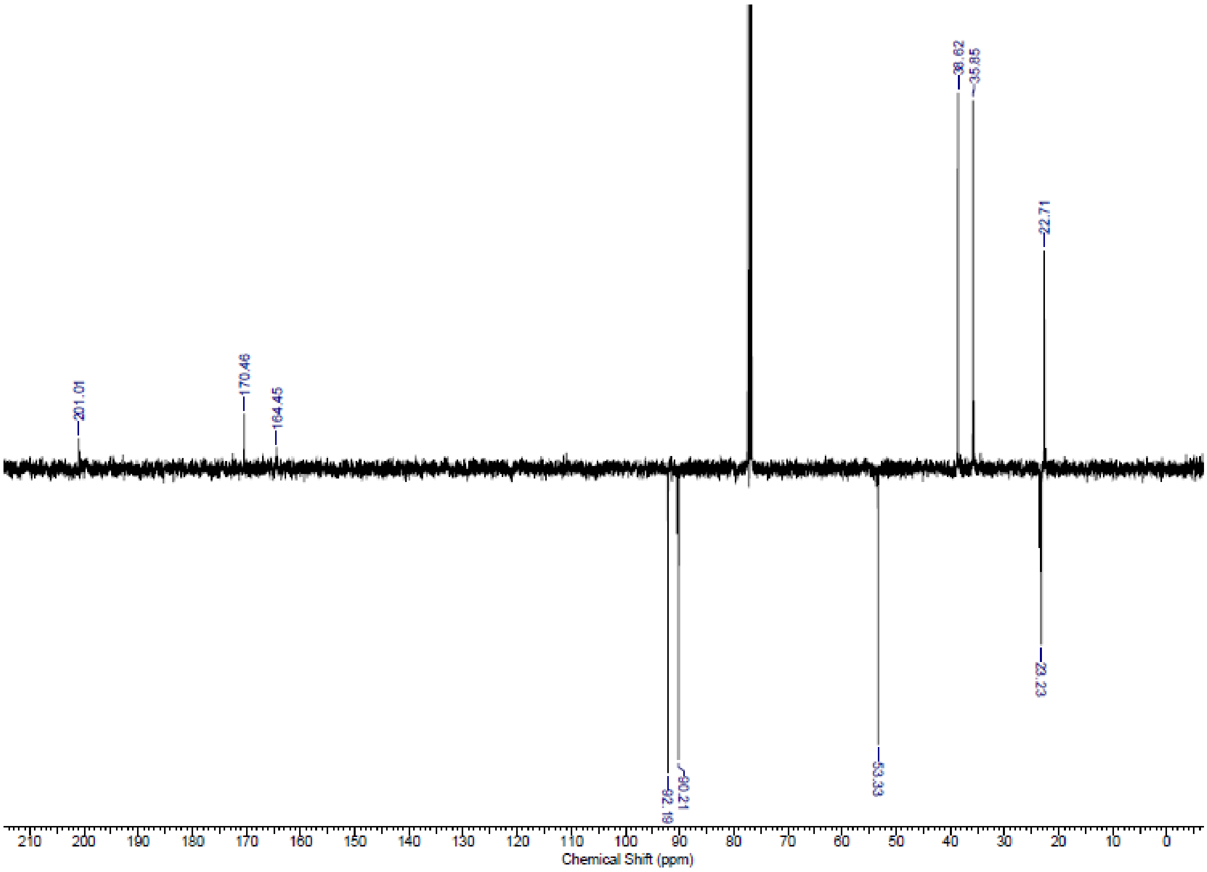

7.22 ¹H- and ¹³C-NMR of compound 11b

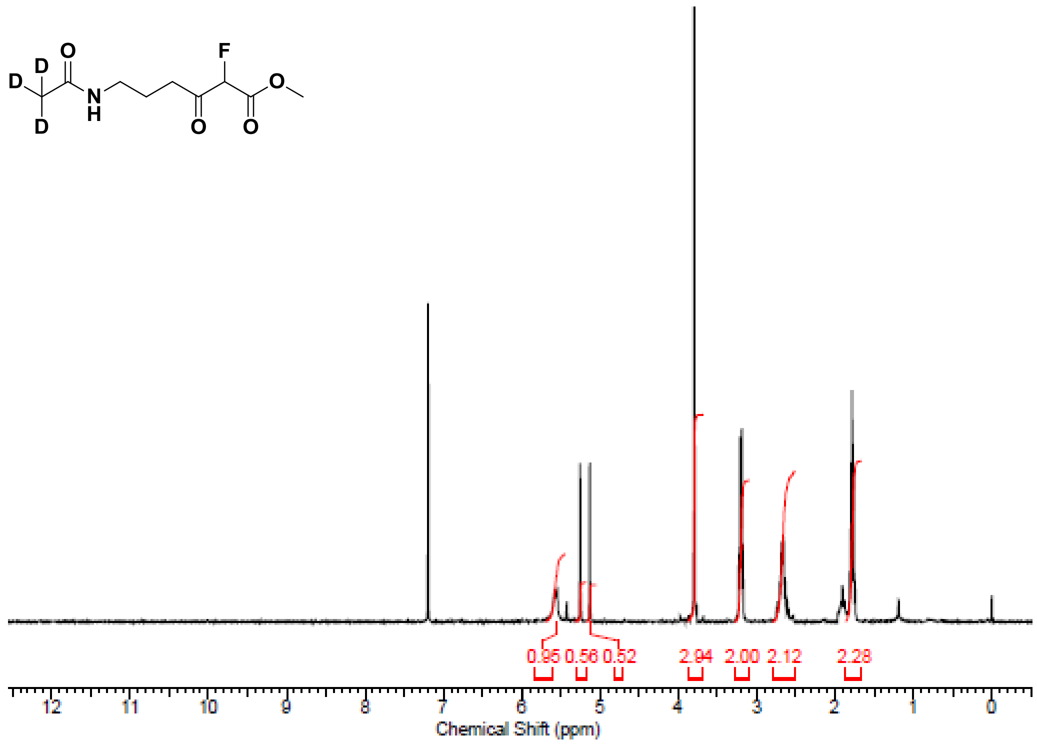

7.23 ¹H- and ¹³C-NMR of compound 69

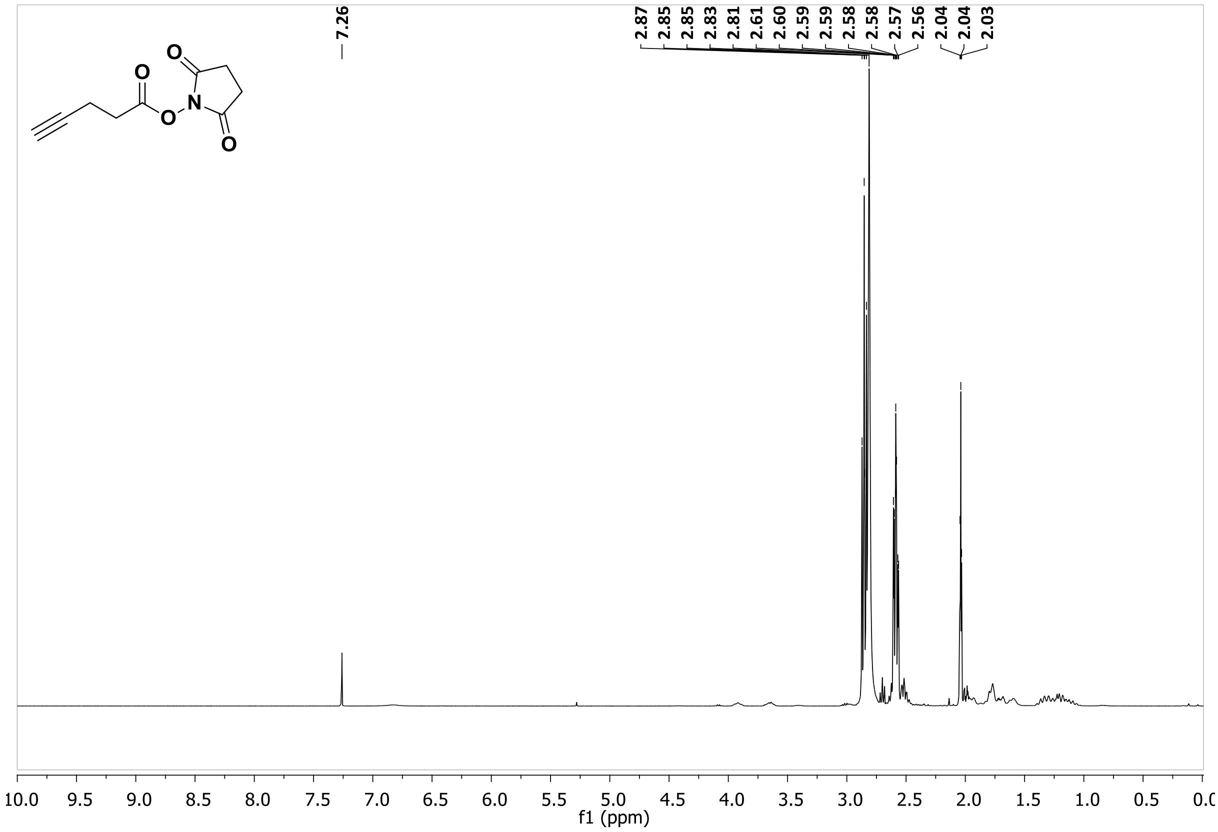

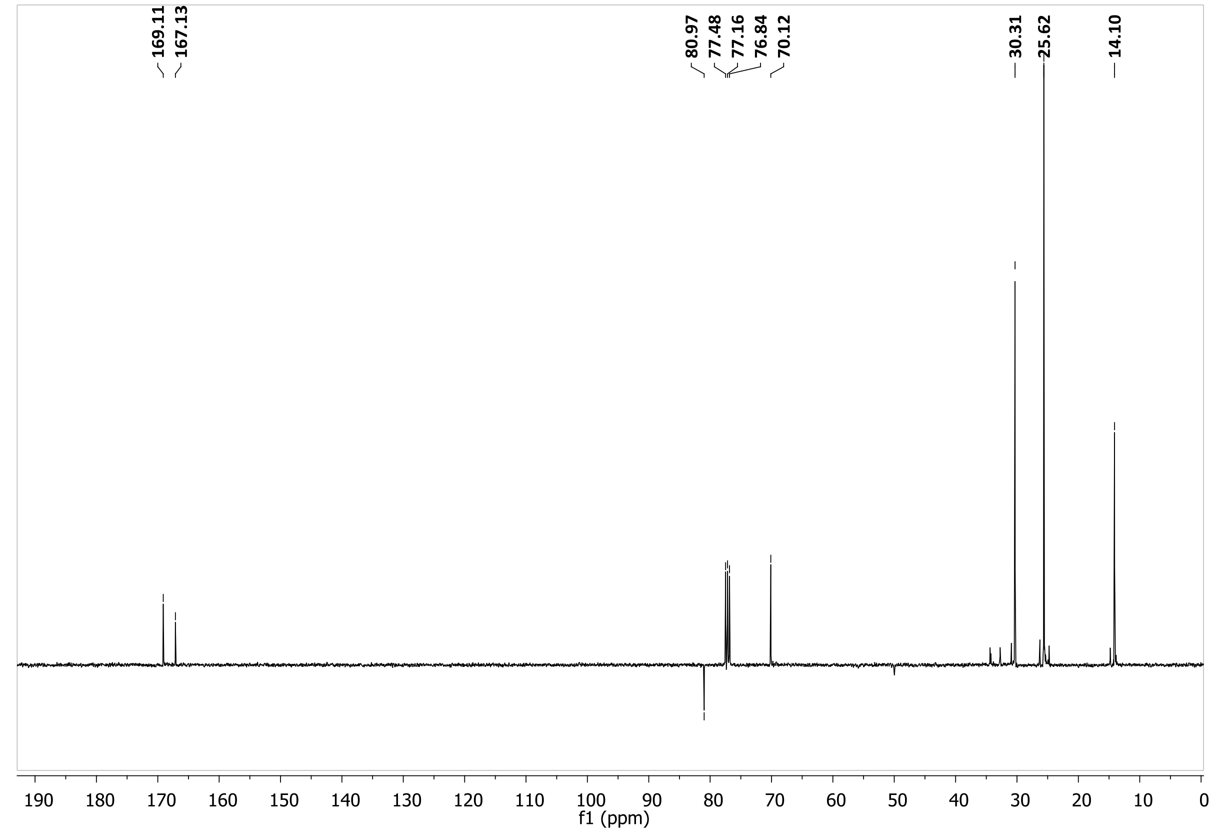

7.24 1H- and 13C-NMR of compound 70

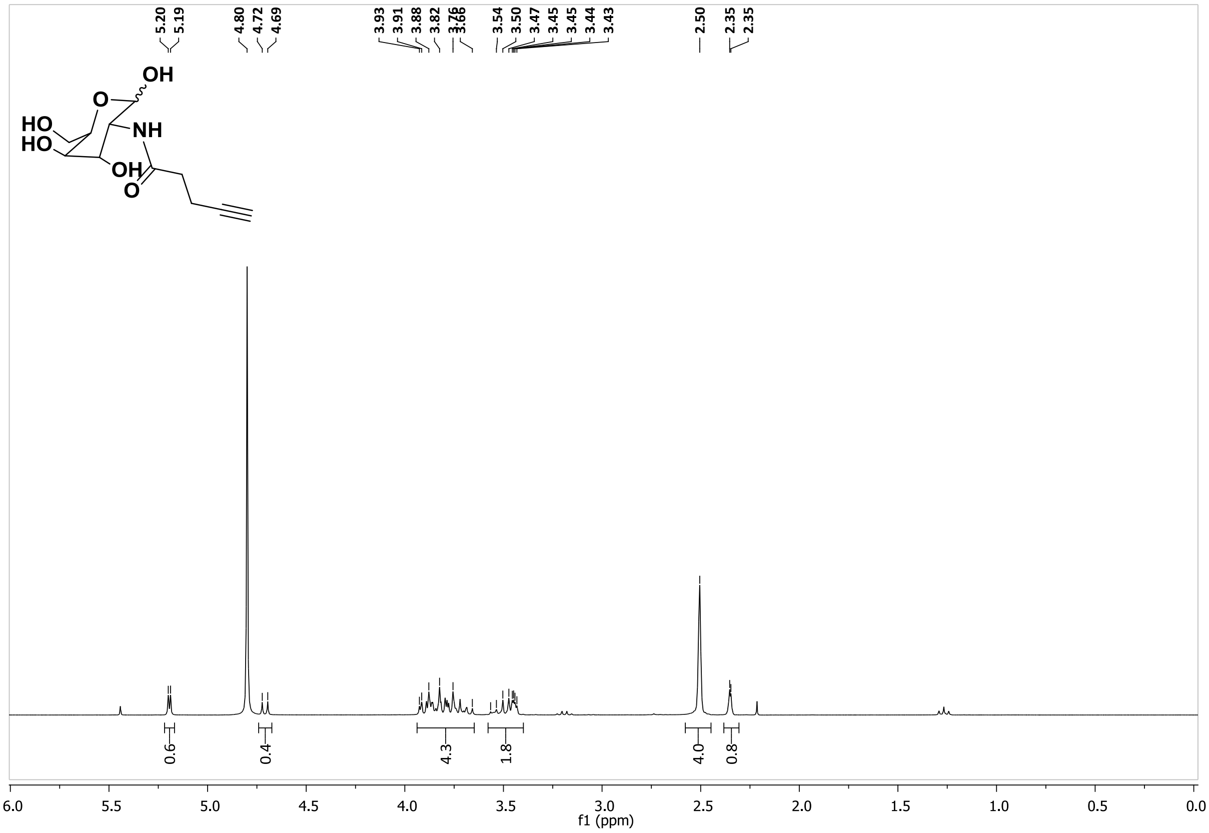

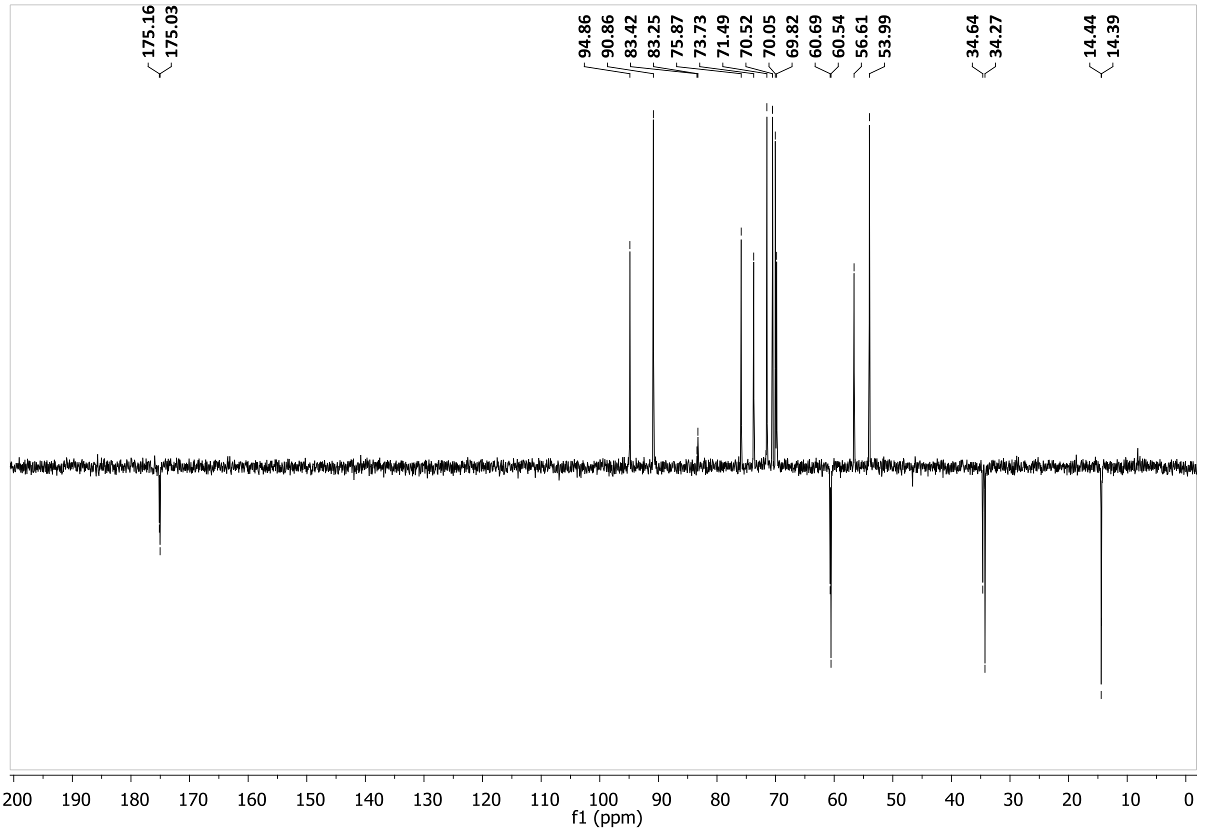

7.25 ^{1}H- and ^{13}C-NMR of compound 46

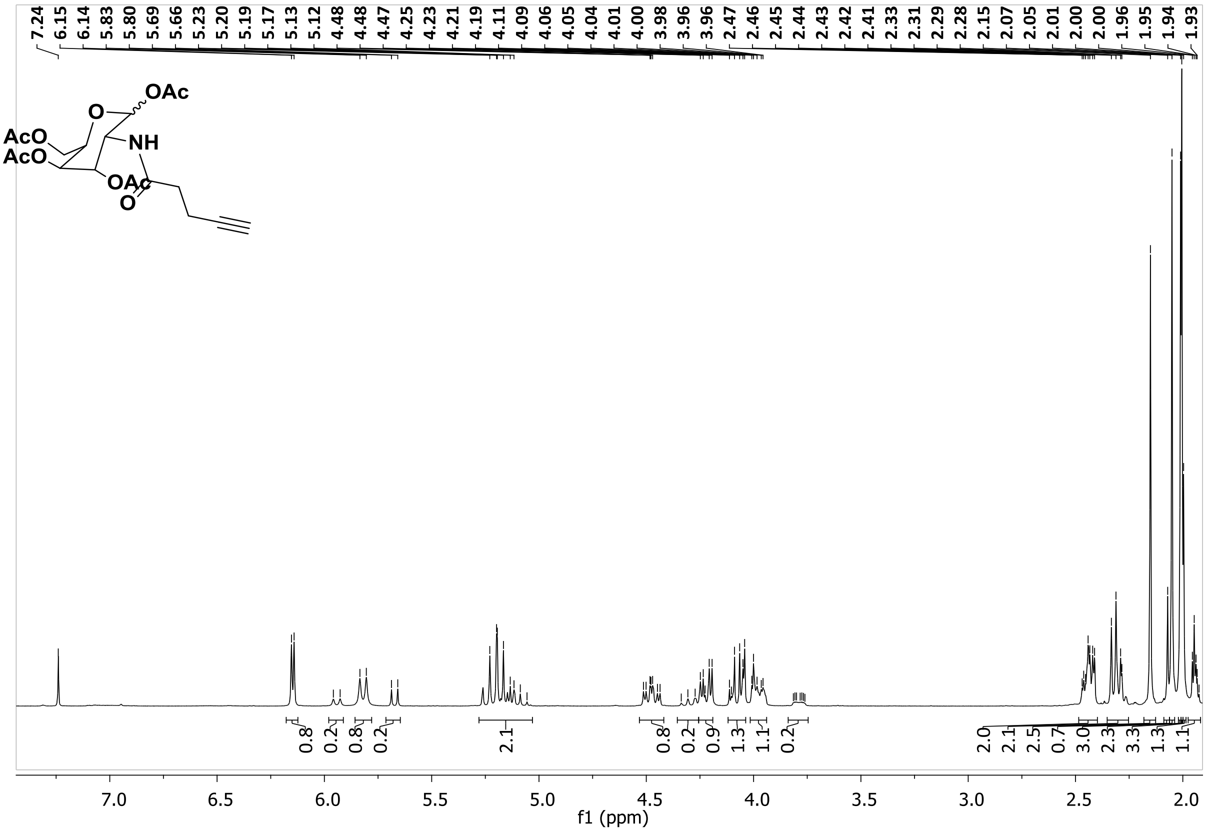

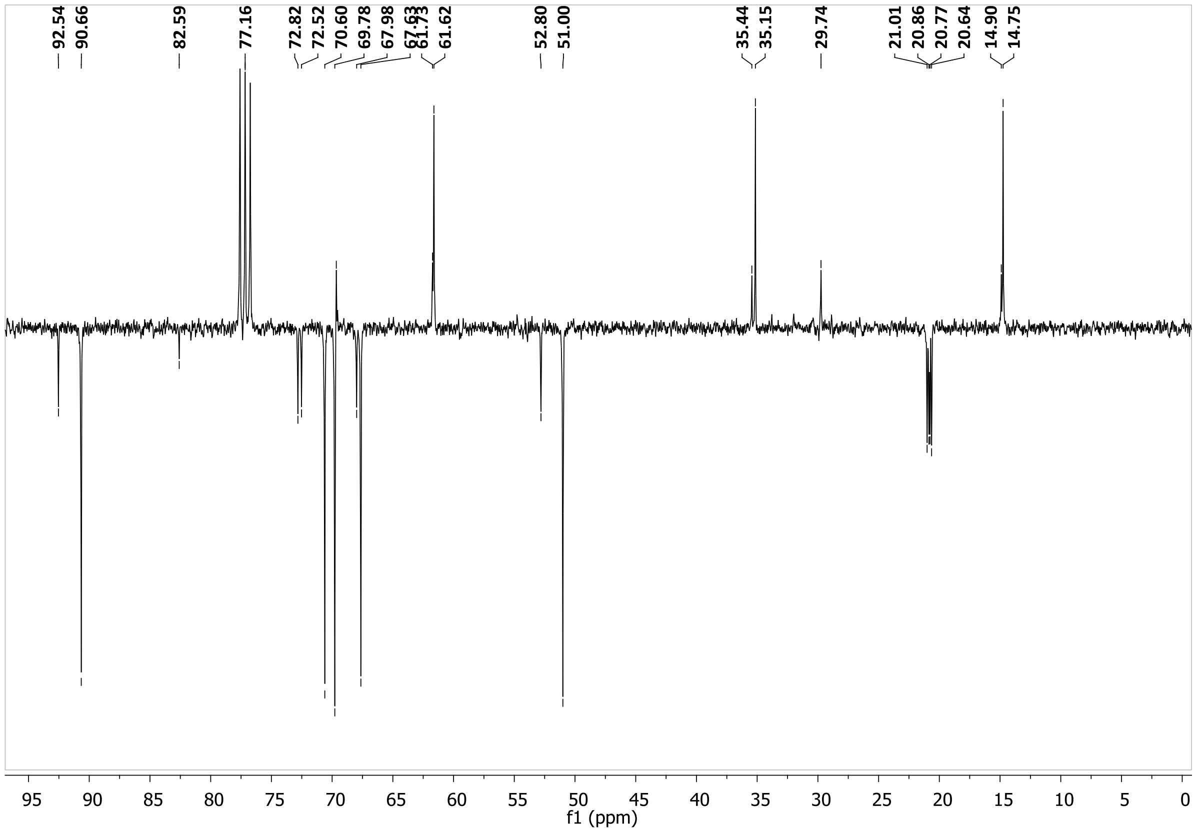

Supplement: Supplementary file 1 [file anie0053-11944-sd1.pdf]
